# Supplementary material for: Identifying gaps in global evidence for nurse staffing and patient care outcomes research in low/middle-income countries: an umbrella review
Source: BMJ Open. 2022 Oct 12;12(10):e064050. doi: 10.1136/bmjopen-2022-064050 (PMC9562716; doi:10.1136/bmjopen-2022-064050)
Supplement: Supplementary data [file bmjopen-2022-064050supp001.pdf]

Online supplemental file 1 : Medline and Embase search strategy

1.	Nursing Staff, Hospital/
2.	Nurse's Role/
3.	Specialties, Nursing/
4.	Nurse-Patient Relations/
5.	((nurse* or nursing) adj3 (staff* or ratio or ratios or workforce or workload or sensitive or mix or practitioner* or assistant* or practice* or performance)).ab,kw,ti.
6.	1 or 2 or 3 or 4 or 5
7.	"Personnel Staffing and Scheduling"/
8.	Health Care Rationing/
9.	"Quality of Health Care"/
10.	Quality Indicators, Health Care/
11.	Patient Safety/
12.	Outcome Assessment, Health Care/
13.	(outcome* or impact* or safety or quality or evidence or indicator* or advers* or mortality or death* or omission* or missed or missing or unmet or undone or rationed or rationing).ti.
14.	"missed nursing".ab,kw,ti.
15.	"missed care".ab,kw,ti.
16.	7 or 8 or 9 or 10 or 11 or 12 or 13 or 14 or 15
17.	(systematic and review).ti.
18.	("meta analysis" or metaanalysis).ti.
19.	"Systematic Review"/

20. Meta-Analysis/
21. 17 or 18 or 19 or 20
22. 6 and 16 and 21

Search strategy in CINAHL

S1	MH "NURSING STAFF, HOSPITAL"/ OR MH "NURSE'S ROLE"/ OR MH "SPECIALTIES, NURSING"/ OR MH "NURSE-PATIENT RELATIONS"/ OR (TI "nurse*" N3 "STAFF*") OR (AB "nurse*" N3 "STAFF*") OR (TI "nurse*" N3 "RATIO") OR (AB "nurse*" N3 "RATIO") OR (TI "nurse*" N3 "RATIOS") OR (AB "nurse*" N3 "RATIOS") OR (TI "nurse*" N3 "WORKFORCE") OR (AB "nurse*" N3 "WORKFORCE") OR (TI "nurse*" N3 "WORKLOAD") OR (AB "nurse*" N3 "WORKLOAD") OR (TI "nurse*" N3 "SENSITIVE") OR (AB "nurse*" N3 "SENSITIVE") OR (TI "nurse*" N3 "MIX") OR (AB "nurse*" N3 "MIX") OR (TI "nurse*" N3 "PRACTITIONER*") OR (AB "nurse*" N3 "PRACTITIONER*") OR (TI "nurse*" N3 "ASSISTANT*") OR (AB "nurse*" N3 "ASSISTANT*") OR (TI "nurse*" N3 "PRACTICE*") OR (AB "nurse*" N3 "PRACTICE*") OR (TI "nurse*" N3 "PERFORMANCE") OR (AB "nurse*" N3 "PERFORMANCE") OR (TI "nursing" N3 "STAFF*") OR (AB "nursing" N3 "STAFF*") OR (TI "nursing" N3 "RATIO") OR (AB "nursing" N3 "RATIO") OR (TI "nursing" N3 "RATIOS") OR (AB "nursing" N3 "RATIOS") OR (TI "nursing" N3 "WORKFORCE") OR (AB "nursing" N3 "WORKFORCE") OR (TI "nursing" N3 "WORKLOAD") OR (AB "nursing" N3 "WORKLOAD") OR (TI "nursing" N3 "SENSITIVE") OR (AB "nursing" N3 "SENSITIVE") OR (TI "nursing" N3 "MIX") OR (AB "nursing" N3 "MIX") OR (TI "nursing" N3 "PRACTITIONER*") OR (AB "nursing" N3 "PRACTITIONER*") OR (TI "nursing" N3 "ASSISTANT*") OR (AB "nursing" N3 "ASSISTANT*") OR (TI "nursing" N3 "PRACTICE*") OR (AB "nursing" N3 "PRACTICE*") OR (TI "nursing" N3 "PERFORMANCE") OR (AB "nursing" N3 "PERFORMANCE")
S2	MH "Personnel Staffing and Scheduling"/ OR MH "Health Care Rationing"/ OR MH "Quality of Health Care"/ OR MH "Quality Indicators, Health Care"/ OR MH "Patient Safety"/ OR MH "Outcome Assessment, Health Care"/ OR TI(outcome* OR impact* OR safety OR quality OR evidence OR indicator* OR advers* OR mortality OR death* OR omission* OR missed OR missing OR unmet OR undone OR rationed OR rationing) OR TI ("missed nursing") OR AB ("missed nursing") OR TI ("missed care") OR AB ("missed care")
S3	TI (systematic AND review) OR TI ("meta analysis" OR metaanalysis) OR MH "Systematic Review"/ OR MH "Meta-Analysis/")
S4	S1 AND S2 AND S3
