# Supplementary material for: Identifying gaps in global evidence for nurse staffing and patient care outcomes research in low/middle-income countries: an umbrella review
Source: BMJ Open. 2022 Oct 12;12(10):e064050. doi: 10.1136/bmjopen-2022-064050 (PMC9562716; doi:10.1136/bmjopen-2022-064050)
Supplement: Supplementary data [file bmjopen-2022-064050supp002.pdf]

Online supplemental file 2 – List of Excluded systematic reviews after full text screening

First Author	Year	Citation	Reason for exclusion	Explanation
Bae et al	2014	Bae SH, Fabry D. Assessing the relationships between nurse work hours/overtime and nurse and patient outcomes: systematic literature review. Nursing outlook. 2014 Mar 1;62(2):138-56.	Wrong intervention	Intervention was nurse working hours which is not a metric of nurse staffing level
Butler et al	2019	Butler M, Schultz TJ, Halligan P, Sheridan A, Kinsman L, Rotter T, Beaumier J, Kelly RG, Drennan J. Hospital nurse-staffing models and patient-and staff-related outcomes. Cochrane Database of Systematic Reviews. 2019(4).	Wrong intervention	Intervention was nurse staffing models not ratio change or change in other metrics of nurse staffing
Wilson et al	2010	Wilson S, Bremner A, Hauck Y. Association between nurse staffing and hospitalised children's health outcomes: A systematic review. JBI Evidence Synthesis. 2010 Jan 1;8(8):1-5.	Protocol paper	
Recio-Saucedo et al	2018	Recio-Saucedo A, Dall'Ora C, Maruotti A, Ball J, Briggs J, Meredith P, Redfern OC, Kovacs C, Prytherch D, Smith GB, Griffiths P. What impact does nursing care left undone have on patient outcomes? Review of the literature. Journal of clinical nursing. 2018 Jun;27(11-12):2248-59.	Wrong intervention	Impact of missed nursing care on patient care outcomes and not the impact of staffing
Shekelle et al	2013	Shekelle PG. Nurse–patient ratios as a patient safety strategy:	Literature review	Narrative literature review

		a systematic review. Annals of internal medicine. 2013 Mar 5;158(5_Part_2):404-9.		
Papastavrou et al	2014	Papastavrou E, Andreou P, Efstathiou G. Rationing of nursing care and nurse–patient outcomes: a systematic review of quantitative studies. The International journal of health planning and management. 2014 Jan;29(1):3-25.	Wrong intervention	Impact of missed nursing care on patient care outcomes and not the impact of nurse staffing
Driscoll et al	2018	Driscoll A, Grant MJ, Carroll D, Dalton S, Deaton C, Jones I, Lehwaldt D, McKee G, Munyombwe T, Astin F. The effect of nurse-to-patient ratios on nurse-sensitive patient outcomes in acute specialist units: a systematic review and meta-analysis. European Journal of Cardiovascular Nursing. 2018 Jan 1;17(1):6-22.	Wrong study setting (non-ward setting)	All papers from intensive care unit
Garcia et al	2010	García F, Sampietro-Colom L. Nursing staff and patient results: systematic review about the existing relationship. Revista de enfermeria (Barcelona, Spain). 2010 Jan 1;33(1):14-26.	Non-English paper	Spanish
Al-ghraiybah et al	2021	Al-ghraiybah T, Sim J, Lago L. The relationship between the nursing practice environment and five nursing-sensitive patient outcomes in acute care hospitals: A systematic review. Nursing Open. 2021 Mar 4.	Wrong intervention	Intervention is nurse practice environment and not nurse staffing

dit Dariel et al	2015	dit Dariel OP, Regnaux JP. Do Magnet®-accredited hospitals show improvements in nurse and patient outcomes compared to non-Magnet hospitals: a systematic review. JBI Evidence Synthesis. 2015 Jun 1;13(6):168-219.	Wrong intervention	Intervention is magnet accreditation and not nurse staffing
Kazanijan et al	2005	Kazanjian A, Green C, Wong J, Reid R. Effect of the hospital nursing environment on patient mortality: a systematic review. Journal of Health Services Research & Policy. 2005 Apr 1;10(2):111-7A.	Wrong intervention	Intervention is nurse practice environment and not nurse staffing
Johnston et al	2015	Johnston MJ, Arora S, King D, Bouras G, Almoudaris AM, Davis R, Darzi A. A systematic review to identify the factors that affect failure to rescue and escalation of care in surgery. Surgery. 2015 Apr 1;157(4):752-63.	Wrong intervention	Focused on factors that affect failure to rescue and not nurse staffing
Kushemererwa et al	2020	Kushemererwa D, Davis J, Moyo N, Gilbert S, Gray R. The Association between Nursing Skill Mix and Mortality for Adult Medical and Surgical Patients: Protocol for a Systematic Review. International Journal of Environmental Research and Public Health. 2020 Jan;17(22):8604.	Protocol paper	Protocol paper and not original article
Olley et al	2018	Olley R, Edwards I, Avery M, Cooper H. Systematic review of the evidence related to mandated nurse staffing ratios in	Wrong outcome	Outcome is on general evidence and not patient care outcomes

		acute hospitals. Australian Health Review. 2018 Apr 17;43(3):288-93.		
Copanitsanou et al	2017	Copanitsanou P, Fotos N, Brokalaki H. Effects of work environment on patient and nurse outcomes. British Journal of Nursing. 2017 Feb 9;26(3):172-6.	original paper	Original paper and not a systematic review
Myers et al	2018	Myers H, Pugh JD, Twigg DE. Identifying nurse-sensitive indicators for stand-alone high acuity areas: A systematic review. Collegian. 2018 Aug 1;25(4):447-56.	Wrong study setting (non-ward setting)	Intensive care unit
Bae et al	2021	Bae SH. Relationships between comprehensive characteristics of nurse work schedules and adverse patient outcomes: A systematic literature review. Journal of Clinical Nursing. 2021 Feb 22.	Wrong intervention	Intervention is nurses' working schedule and not nurse staffing
Butler et al	2011	Butler M, Collins R, Drennan J, Halligan P, O'Mathuna D, Schultz T, Sheridan A, Vilis E. Hospital nurse staffing models and patient and staff-related outcomes-an epic EPOC systematic review. JBI Evidence Implementation. 2011 Sep 1;9(3):326.	Wrong intervention	Intervention is nurse staffing models not a change in nurse staffing numbers
Bae et al	2011	BAE SH. Assessing the relationships between nurse working conditions and patient outcomes: systematic literature review. Journal of nursing management. 2011 Sep;19(6):700-13.	Wrong intervention	Intervention is nurse working conditions and not nurse staffing
