# Supplementary material for: Identifying gaps in global evidence for nurse staffing and patient care outcomes research in low/middle-income countries: an umbrella review
Source: BMJ Open. 2022 Oct 12;12(10):e064050. doi: 10.1136/bmjopen-2022-064050 (PMC9562716; doi:10.1136/bmjopen-2022-064050)
Supplement: Supplementary data [file bmjopen-2022-064050supp003.pdf]

Online supplemental file 3: Excluded primary studies and reasons for exclusion.

systematic review	Title	reason for exclusion
Griffith et al		
	Zander, B., Dobler, L., Bäumler, M. and Busse, R., 2014. Implicit rationing of care services in German acute care hospitals – Results of the international nursing study Rn4cast. Health Care, 76(11), pp.727-734.	German
Assaye et al		
	Soares M, Bozza FA, Angus DC, Japiassu AM, Viana WN, Costa R, et al. Organizational characteristics, outcomes, and resource use in 78 Brazilian intensive care units: the ORCHESTRA study. Intensive Care Med 2015;41(12):2149– 60.	ICU setting
	Silva MCMd, Sousa RMCd, Padilha KG. Factors associated with death and readmission into the Intensive Care Unit. Rev Lat Am Enfermagem 2011;19:911–9.	ICU setting
	Chittawatanarat K, Sataworn D, Thongchai C. Thai Society of Critical Care Medicine Study G. Effects of ICU characters, human resources and workload to outcome indicators in Thai ICUs: the results of ICU-RESOURCE I study. J Med Assoc Thai 2014;97(Suppl 1):S22–30.	ICU setting
	Cremasco MF, Wenzel F, Zanei SSV, Whitaker IY. Pressure ulcers in the intensive care unit: the relationship between nursing workload, illness severity and pressure ulcer risk. J Clin Nurs 2013;22(15/16):2183–91.	ICU setting
	Al Tehewy M, Fahim H, Gad NI, El Gafary M, Rahman SA. Medication administration errors in a university hospital. J Patient Saf 2016;12(1):34–9.	Wrong intervention (number and type of shift)

	Daud-Gallotti RM, Costa SF, Guimara˜es T, Padilha KG, Inoue EN, Vasconcelos TN, et al. Nursing workload as a risk factor for health care associated infections in ICU: a prospective study. PLoS One 2012;7(12):e52342.	ICU setting
	Celen MK, Tamam Y, Hosoglu S, Ayaz C, Geyik MF, Apak I. Multiresistant bacterial colonization due to increased nurse workload in a neurology intensive care unit. Neurosciences 2006;11(4):265–70.	ICU setting
	Aycan IO, Celen MK, Yilmaz A, Almaz MS, Dal T, Celik Y, et al. Bacterial colonization due to increased nurse workload in an intensive care unit. Rev Brasil Anestesiol 2015;65:180–5	ICU setting
	. Cremasco MF, Wenzel F, Zanei SSV, Whitaker IY. Pressure ulcers in the intensive care unit: the relationship between nursing workload, illness severity and pressure ulcer risk. J Clin Nurs 2013;22(15/16):2183–91	ICU setting
	Bjorklund de Lima L, Rejane Rabelo E. Nursing workload in the post-anesthesia care unit. Acta Paul Enferm 2013;26(2): 116–22	Wrong intervention (Nursing workload measure)
	Day SW. Evaluating the impact of the Guatemalan Nursing Program on staff, organizational, and clinical outcomes. University of Tennessee Health Science Center; 2010; p. 93	Wrong intervention (Addition of a nurse educator)
	Ilhan MN, Durukan E, Taner E, Maral I, Bumin MA. Burnout and its correlates among nursing staff: questionnaire survey. J Adv Nurs 2008;61(1):100–6	Nurse outcome (Burn out)
	Karakoc A, Yilmaz M, Alcalar N, Esen B, Kayabasi H, Sit D. Burnout syndrome among hemodialysis and peritoneal dialysis nurses. Iran J Kidney Dis 2016;10(6):395–404.	Nurse outcome (Burn out)
	Roomaney R, Steenkamp J, Kagee A. Predictors of burnout among HIV	Nurse outcome (Burn out)

	nurses in the Western Cape. Curationis 2017;40(1):1–9	
	Kunaviktikul W, Wichaikhum O, Nantsupawat A, Nantsupawat R, Chontawan R, Klunklin A et al. Nurses' extended work hours: patient, nurse and organizational outcomes. Int Nurs Rev 2015;62(3):386–93.	Wrong intervention (Nursing workload measure)
	De Paiva LC, Gomes Cana´rio AC, Corsino de Paiva China EL, Gonc¸alves AK. Burnout syndrome in health-care professionals in a university hospital. Clinics 2017;72(5):305–9.	Nurse outcome (Burn out)
	Lu MM, Ruan H, Xing WJ, Hu Y. Nurse burnout in China: a questionnaire survey on staffing, job satisfaction, and quality of care. J Nurs Manag 2015;23(4):4	Nurse outcome (Burn out)
	Mohammadpoorasl A, Maleki A, Sahebihagh MH. Prevalence of professional burnout and its related factors among nurses in Tabriz in 2010. IJNMR 2012;17(7):524–9	Nurse outcome (Burn out)
	Naz S, Hashmi AM, Asif A. Burnout and quality of life in nurses of a tertiary care hospital in Pakistan. J Pak Med Assoc 2016;66(5):532–6	Nurse outcome (Burn out)
	. Negi Y, Bagga R. Burnout among nursing professionals in tertiary care hospitals of Delhi. J Health Manag 2015;17(2):163–77	Nurse outcome (Burn out)
	Nantsupawat A, Srisuphan W, Kunaviktikul W, Wichaikhum, Aungsuroch Y, Aiken LH. Impact of nurse work environment and staffing on hospital nurse and quality of care in Thailand. J Nurs Scholarsh 2011;43(4):426–32	Nurse outcome (Burn out)
	Nantsupawat A, Nantsupawat R, Kulnaviktikul W, McHugh MD. Relationship between nurse staffing levels and nurse outcomes in community hospitals, Thailand. Nurs Health Sci 2015;17(1):112–8	Nurse outcome (Emotional exhaustion and Needle stick injuries)

Thungjaroenkul P, Cummings GG, Embleton A. The impact of nurse staffing on hospital costs and patient length of stay: a systematic review. Nursing Economics. 2007 Sep 1;25(5):255.		
	Amaravadi, R.K., Dimick, J.B., Pronovost, P.J., & Lipsett, P.A. (2000). ICU nurseto-patient ratio is associated with complications and resource use after esophagectomy. Intensive Care Medicine, 26, 1857-1862.	ICU setting
	Pronovost PJ, Jenckes MW, Dorman T, Garrett E, Breslow MJ, Rosenfeld BA, Lipsett PA, Bass E. Organizational characteristics of intensive care units related to outcomes of abdominal aortic surgery. Jama. 1999 Apr 14;281(14):1310-7.	ICU setting
	Lassnigg A, Hiesmayr M, Bauer P, Haisjackl M. Effect of centre-, patient-and procedure-related factors on intensive care resource utilisation after cardiac surgery. Intensive care medicine. 2002 Oct;28(10):1453-61.	ICU setting
	Dimick, Justin B., et al. "Effect of nurse-to-patient ratio in the intensive care unit on pulmonary complications and resource use after hepatectomy." American Journal of Critical Care 10.6 (2001): 376.	ICU setting

	Bloom JR, Alexander JA, Nuchols BA. Nurse staffing patterns and hospital efficiency in the United States. Social science & medicine. 1997 Jan 1;44(2):147-55.	Wrong outcome (cost of care)
	Lee TY, Yeh ML, Chen HH, Lien GH. The skill mix practice model for nursing: measuring outcome. Journal of advanced nursing. 2005 Aug;51(4):406-13.	Wrong outcome (cost of care)
	Hall LM, Doran D, Pink GH. Nurse staffing models, nursing hours, and patient safety outcomes. JONA: The Journal of Nursing Administration. 2004 Jan 1;34(1):41-5.	Wrong outcome (cost of care)
	Pratt RA, Burr G, Leelarthaepin B, Blizard P, Walsh S. The effects of All-RN and RN-EN staffing on the quality and cost of patient care. The Australian journal of advanced nursing: a quarterly publication of the Royal Australian Nursing Federation. 1993 Mar 1;10(3):27-39.	Wrong intervention (skill mix)
	Newhouse RP, Johantgen M, Pronovist PJ, Johnson E. Perioperative nurses and patient outcomes: mortality, complications, and length of stay. The Journal of nursing administration. 2010 Oct 1;40(10 Suppl):S54-67.	Wrong intervention (skill mix)
	Needleman J, Buerhaus PI, Stewart M, Zelevinsky K, Mattke S. Nurse staffing in hospitals: is there a business case for quality?. Health Affairs. 2006 Jan;25(1):204-11.	Wrong intervention (costs)
	Barkell NP, Killinger KA, Schultz SD. The relationship between nurse staffing models and patient outcomes: a descriptive study. Outcomes Management. 2002 Jan 1;6(1):27-33.	Wrong intervention (skill mix)
	McCue M, Mark BA, Harless DW. Nurse staffing, quality, and financial performance. Journal of health care finance. 2003 Jan 1;29(4):54-76.	Wrong outcome (cost of care)

Twigg DE, Whitehead L, Doleman G, El-Zaemey S. The impact of nurse staffing methodologies on nurse and patient outcomes: A systematic review. Journal of Advanced Nursing. 2021 Jun 3.		
	Bowblis, J. R., & Ghattas, A. (2017). The impact of minimum quality standard regulations on nursing home staffing, quality, and exit decisions. Review of Industrial Organization, 50(1), 43–68. https://doi. org/10.1007/s11151-016-9528-x	nursing home
	Chan, T. C., Killeen, J. P., Vilke, G. M., Marshall, J. B., & Castillo, E. M. (2010). Effect of mandated nurse–patient ratios on patient wait time and care time in the emergency department. Academic Emergency Medicine, 17(5), 545–552. https://doi. org/10.1111/j.1553-2712.2010.00727.x	Emergency department
	Chen, M. M., & Grabowski, D. C. (2015). Intended and unintended consequences of minimum staffing standards for nursing homes. Health Economics, 24(7), 822–839. https://doi.org/10.1002/hec.3063	nursing home
	Cox, K. S., Anderson, S. C., Teasley, S. L., Sexton, K. A., & Carroll, C. A. (2005). Nurses' work environment perceptions when employed in states with and without mandatory staffing ratios and/or mandatory staffing plans. Policy, Politics, &	Nurse outcomes

	Nursing Practice, 6(3), 191–197. https://doi.org/10.1177/1527154405279091	
	Hodgson, A., Morgan, D., & Peterson, R. (2016). Does better nurse staffing improve detection of depression and anxiety as secondary conditions in hospitalized patients with pneumonia? Nursing Economics, 34(3), 134	Mental health outcome
	Law, A. C., Stevens, J. P., Hohmann, S., & Walkey, A. J. (2018). Patient outcomes after the introduction of statewide intensive care unit nurse staffing regulations. Critical Care Medicine, 46(10), 1563.	ICU setting
	Leigh, J. P., Markis, C. A., Iosif, A.-M., & Romano, P. S. (2015). California's nurse-to-patient ratio law and occupational injury. International Archives of Occupational and Environmental Health, 88(4), 477–484.	Nurse outcome
	Park, J., & Stearns, S. C. (2009). Effects of state minimum staffing standards on nursing home staffing and quality of care. Health Services Research, 44(1), 56–78. https://doi.org/10.1111/j.1475-6773.2008.00906.x	nursing home
	Spetz J, Herrera C. Changes in nurse satisfaction in California, 2004 to 2008. Journal of Nursing Management. 2010 Jul;18(5):564-72.	nurse outcome
	Spetz, J. (2008). Nurse satisfaction and the implementation of minimum nurse staffing regulations. Policy, Politics, & Nursing Practice, 9(1), 15–21. https://doi.org/10.1177/1527154408316950	Nurse outcome
	Tellez, M. (2012). Work satisfaction among California registered nurses: A longitudinal comparative analysis. Nursing economic$, 30(2), 73–81	Nurse outcome

	Tellez, M., & Seago, J. A. (2013). California nurse staffing law and RN workforce changes. Nursing Economics, 31(1), 18	Nurse outcome
	Matsudaira, J. D. (2014). Government regulation and the quality of healthcare evidence from minimum staffing legislation for nursing homes. Journal of Human Resources, 49(1), 32–72. https://doi.org/10.1353/jhr.2014.0003	nursing home
	Weichenthal, L., & Hendey, G. W. (2011). The effect of mandatory nurse ratios on patient care in an emergency department. The Journal of Emergency Medicine, 40(1), 76–81. https://doi.org/10.1016/j.jemermed.2009.02.037	Emergency department
	Zhang, X., & Grabowski, D. C. (2004). Nursing home staffing and quality under the nursing home reform act. The Gerontologist, 44(1), 13–23. https://doi.org/10.1093/geront/44.1.13	nursing home
Shin et al 2019		
	UK Neonatal Staffing Study Group. Patient volume, staffing, and workload in relation to risk-adjusted outcomes in a random stratified sample of UK neonatal intensive care units: a prospective evaluation. The Lancet. 2002 Jan 12;359(9301):99-107.	ICU setting
	Brooks-Carthon JM, Kutney-Lee A, Sloane DM, Cimiotti JP, Aiken LH. Quality of care and patient satisfaction in hospitals with high concentrations of black patients. Journal of Nursing Scholarship. 2011 Sep;43(3):301-10.	wrong intervention (practice environment)
Lang 2004		
	Kustaborder MJ, Rigney M. Interventions for safety.	wrong intervention (nurse staffing mix)

	Bostrom J, Zimmerman J. Restructuring nursing for a competitive health care environment. Nursing economic$. 1993 Jan 1;11(1):35-41.	wrong intervention (nurse restructuring/ skill mix)
	Needleman J, Buerhaus P. Nurse staffing and patient safety: current knowledge and implications for action. International Journal for Quality in Health Care. 2003 Aug 1;15(4):275-7.	editorial
	Behner KG, Fogg LF, Fournier LC, Frankenbach JT, Robertson SB. Nursing resource management: analyzing the relationship between costs and quality in staffing decisions. Health Care Management Review. 1990 Jan 1;15(4):63-71.	wrong intervention (labour efficiency)
	Shukla RK. All-RN model of nursing care delivery: A cost-benefit evaluation. Inquiry. 1983 Jul 1:173-84.	wrong intervention
	Arndt M, Crane S. Influences on nursing care volume. Journal of the Society for Health Systems. 1998 Jan 1;5(4):38-49.	nurse outcome
	Hinshaw AS, Scofield R, Atwood JR. Staff, patient, and cost outcomes of all-registered nurse staffing. JONA: The Journal of Nursing Administration. 1981 Nov 1;11(11):30-6.	Wrong intervention (skill mix)
	Grillo-Peck AM, Risner PB. The effect of a partnership model on quality and length of stay. Nursing economic$. 1995 Nov 1;13(6):367-72.	wrong intervention (nurse partnership model)
	Ceria CD. Nursing absenteeism and its effects on the quality of patient care. The Journal of nursing administration. 1992 Dec;22(12):11-38.	wrong intervention (absenteeism)
	Bloom JR, Alexander JA, Nuchols BA. The effect of the social organization of work on the voluntary turnover rate of hospital nurses in the United	nurse outcome

	States. Soc Sci Med. 1992;34:1413-1424.	
	Lanza ML, Kayne HL, Gulliford D, et al. Staffing of inpatient psychiatric units and assault by patients. J Am Psychiatr Nurses Assoc. 1997;3:42-4	nurse outcome
	Shortell SM, Hughes EF. The effects of regulation, competition, and ownership on mortality rates among hospital inpatients. New England Journal of Medicine. 1988 Apr 28;318(17):1100-7.	wrong intervention
	Clarke SP, Sloane DM, Aiken LH. Effects of hospital staffing and organizational climate on needlestick injuries to nurses. American journal of public health. 2002 Jul;92(7):1115-9.	nurse outcome
	GLANDON GL, COLBERT KW, Thomasma M. Nursing delivery models and RN mix: cost implications. Nursing Management. 1989 May 1;20(5):30-3.	nurse outcome
	Halloran EJ. RN staffing: more care—less cost. Nursing Management. 1983 Sep 1;14(9):18-23.	nurse outcome
	Osinski EG, Powals JG. The cost of all RN staffed primary nursing. Supervisor nurse. 1980 Jan;11(1):16-21.	nurse outcome
	Bradbury RC, Golec JH, Steen PM. Relating hospital health outcomes and resource expenditures. Inquiry. 1994 Apr 1:56-65.	Wrong intervention (nurse skill mix)
	Krakauer H, Bailey RC, Skellan KJ, Stewart JD, Hartz AJ, Kuhn EM, Rimm AA. Evaluation of the HCFA model for the analysis of mortality following hospitalization. Health services research. 1992 Aug;27(3):317.	Wrong intervention (nurse skill mix)
	Kuhn EM, Hartz A, Gottlieb MS, Rimm AA. The relationship of hospital characteristics and the results of peer review in six large	nurse outcome

	states. Med Care. 1991;29:1028-1038.	
Kane 2007		
	Fridkin SK, Pear SM, Williamson TH, Galgiani JN, Jarvis WR. The role of understaffing in central venous catheter-associated bloodstream infection. Infection Control & Hospital Epidemiology. 1996 Mar;17(3):150-8.	ICU setting
	Amaravadi RK, Dimick JB, Pronovost PJ, et al. ICU nurse-to-patient ratio is associated with complications and resource use after esopha- gectomy. Intensive Care Med. 2000;26: 18	ICU setting
	Dang D, Johantgen ME, Pronovost PJ, et al. Postoperative complica-tions: does intensive care unit staff nursing make a difference? Heart Lung. 2002;3 1:219-	ICU setting
	Dimick JB, Swoboda SM, Pronovost PJ, et al. Effect of nurse-to-patient ratio in the intensive care unit on pulmonary complications and re-source use after hepatectomy. Am J Crit Care. 2001	ICU setting
	Pronovost PJ, Dang D, Dorman T, et al. Intensive care unit nurse staffing and the risk for complications after abdominal aortic surgery. EffClin Pract. 2001 ;4: 19	ICU setting
	Pronovost PJ, Jenckes MW, Dorman T, et al. Organizational charac-teristics of intensive care units related to outcomes of abdominal aortic surgery. JAMA. 1999;281:1310-1	ICU setting
	Shortell SM, Zimmerman JE, Rousseau DM, et al. The performance of intensive care units: does good management make a difference? Med Care. 1994;32:508-525.	ICU setting
	Cimiotti JP, Haas J, Saiman L, et al. Impact of staffing on bloodstream infections in the neonatal intensive	ICU setting

	care unit. Arch Pediatr Adolesc Med. 2006;160:832-836	
	Marcin JP, Rutan E, Rapetti PM, et al. Nurse staffing and unplanned extubation in the pediatric intensive care unit. Pediatr Crit Care Med. 2005;6:254-257.	ICU setting
	Cimiotti JP. Nurse Staffing and Healthcare-Associated Infections in the Neonatal ICU [dissertation]. Ann Arbor, MI: Columbia University; 2004: AAT 3128935.	ICU setting
	Robert J, Fridkin SK, Blumberg HM, Anderson B, White N, Ray SM, Chan J, Jarvis WR. The influence of the composition of the nursing staff on primary bloodstream infection rates in a surgical intensive care unit. Infection Control & Hospital Epidemiology. 2000 Jan;21(1):12-7.	ICU setting
Labelle 2019		
	Dang D, Johantgen ME, Pronovost PJ, Jenckes MW, Bass EB. Postoperative complications: does intensive care unit staff nursing make a difference?. Heart & Lung. 2002 May 1;31(3):219-28.	ICU setting
	Rao AD, Kumar A, McHugh M. Better nurse autonomy decreases the odds of 30-day mortality and failure to rescue. Journal of Nursing Scholarship. 2017 Jan;49(1):73-9.	wrong intervention (nurse work environment)
	Diya L, Van den Heede K, Sermeus W, Lesaffre E. The relationship between in-hospital mortality, readmission into the intensive care nursing unit and/or operating theatre and nurse staffing levels. Journal of advanced nursing. 2012 May;68(5):1073-81.	ICU setting
	Hickey PA, Gauvreau K, Jenkins K, Fawcett J, Hayman L. Statewide and national impact of California's staffing law on pediatric cardiac surgery outcomes. JONA: The	ICU setting

	Journal of Nursing Administration. 2011 May 1;41(5):218-25.	
	Hickey P, Gauvreau K, Connor J, Sporing E, Jenkins K. The relationship of nurse staffing, skill mix, and Magnet® recognition to institutional volume and mortality for congenital heart surgery. JONA: The Journal of Nursing Administration. 2010 May 1;40(5):226-32.	ICU setting
	Dimick JB, Swoboda SM, Pronovost PJ, Lipsett PA. Effect of nurse-to-patient ratio in the intensive care unit on pulmonary complications and resource use after hepatectomy. American Journal of Critical Care. 2001 Nov 1;10(6):376.	ICU setting
	Kiekkas P, Sakellaropoulos GC, Brokalaki H, Manolis E, Samios A, Skartsani C, Baltopoulos GI. Association between nursing workload and mortality of intensive care unit patients. Journal of nursing scholarship. 2008 Dec;40(4):385-90.	ICU setting
	Olds DM, Aiken LH, Cimiotti JP, Lake ET. Association of nurse work environment and safety climate on patient mortality: A cross-sectional study. International journal of nursing studies. 2017 Sep 1;74:155-61.	wrong intervention (nurse work environment)
	Kendall-Gallagher D, Aiken LH, Sloane DM, Cimiotti JP. Nurse specialty certification, inpatient mortality, and failure to rescue. Journal of Nursing Scholarship. 2011 Jun;43(2):188-94.	wrong intervention (nurse education)
	Lane-Fall MB, Ramaswamy TS, Brown SE, He X, Gutsche JT, Fleisher LA, Neuman MD. Structural, nursing, and physician characteristics and 30-day mortality for patients undergoing cardiac	wrong intervention (nurse education)

	surgery in Pennsylvania. Critical care medicine. 2017 Sep;45(9):1472.	
	Diya L, Lesaffre E, Van den Heede K, Sermeus W, Vleugels A. Establishing the relationship between nurse staffing and hospital mortality using a clustered discrete-time logistic model. Statistics in medicine. 2010 Mar 30;29(7-8):778-85.	ICU setting
	Aiken LH, Clarke SP, Cheung RB, Sloane DM, Silber JH. Educational levels of hospital nurses and surgical patient mortality. Jama. 2003 Sep 24;290(12):1617-23.	wrong intervention (nurse education)
	Aiken LH, Clarke SP, Sloane DM, International Hospital Outcomes Research Consortium. Hospital staffing, organization, and quality of care: cross-national findings. International Journal for quality in Health care. 2002 Feb 1;14(1):5-14.	wrong outcome (nurse outcomes)
	Ozdemir BA, Sinha S, Karthikesalingam A, Poloniecki JD, Pearse RM, Grocott MP, Thompson MM, Holt PJ. Mortality of emergency general surgical patients and associations with hospital structures and processes. BJA: British Journal of Anaesthesia. 2016 Jan 1;116(1):54-62.	Emergency department
	Newhouse RP, Johantgen M, Pronovost PJ, Johnson E. Perioperative nurses and patient outcomes—mortality, complications, and length of stay. AORN journal. 2005 Mar 1;81(3):508-28.	Operating room/peri-operative setting
	Aiken LH, Sloane D, Griffiths P, Rafferty AM, Bruyneel L, McHugh M, Maier CB, Moreno-Casbas T, Ball JE, Ausserhofer D, Sermeus W. Nursing skill mix in European hospitals: cross-sectional study of the association with mortality, patient ratings, and quality of care.	wrong intervention (nursing skill mix)

	BMJ quality & safety. 2017 Jul 1;26(7):559-68.	
Wilson 2011		
	Archibald LK, Manning ML, Bell LM, Banerjee S, Jarvis WR. Patient density, nurse-to-patient ratio and nosocomial infection risk in a pediatric cardiac intensive care unit. The Pediatric infectious disease journal. 1997 Nov 1;16(11):1045-8.	ICU setting
	Cimiotti JP. Nurse staffing and healthcare-associated infections in the neonatal ICU. Columbia University; 2004	ICU setting
	Marcin JP, Rutan E, Rapetti PM, et al. Nurse staffing and unplanned extubation in the pediatric intensive care unit. Pediatr Crit Care Med. 2005;6:254-257.	ICU setting
Engineer 2015		
	Volpp KG, Rosen AK, Rosenbaum PR, Romano PS, Even-Shoshan O, Wang Y, Bellini L, Behringer T, Silber JH. Mortality among hospitalized Medicare beneficiaries in the first 2 years following ACGME resident duty hour reform. Jama. 2007 Sep 5;298(9):975-83.	wrong intervention
	Bradley EH, Herrin J, Curry L, Cherlin EJ, Wang Y, Webster TR, Drye EE, Normand SL, Krumholz HM. Variation in hospital mortality rates for patients with acute myocardial infarction. The American journal of cardiology. 2010 Oct 15;106(8):1108-12.	wrong intervention
	Weiner BJ, Alexander JA, Shortell SM, Baker LC, Becker M, Geppert JJ. Quality improvement implementation and hospital performance on quality indicators. Health services research. 2006 Apr;41(2):307-34.	wrong intervention

	Gaskin DJ, Spencer CS, Richard P, Anderson G, Powe NR, LaVeist TA. Do minority patients use lower quality hospitals?. INQUIRY: The Journal of Health Care Organization, Provision, and Financing. 2011 Aug;48(3):209-20.	wrong intervention
	Carretta HJ, Chukmaitov A, Tang A, Shin J. Examination of hospital characteristics and patient quality outcomes using four inpatient quality indicators and 30-day all-cause mortality. American Journal of Medical Quality. 2013 Jan;28(1):46-55.	wrong intervention
	Chukmaitov AS, Bazzoli GJ, Harless DW, Hurley RE, Devers KJ, Zhao M. Variations in inpatient mortality among hospitals in different system types, 1995 to 2000. Medical care. 2009 Apr 1:466-73.	wrong intervention
	Friedman B, Jiang HJ. Do Medicare Advantage enrollees tend to be admitted to hospitals with better or worse outcomes compared with fee-for-service enrollees?. International journal of health care finance and economics. 2010 Jun;10(2):171-85.	wrong intervention
	Romley JA, Jena AB, Goldman DP. Hospital spending and inpatient mortality: evidence from California: an observational study. Annals of internal medicine. 2011 Feb 1;154(3):160-7.	wrong intervention
	Ross JS, Normand SL, Wang Y, Nallamothu BK, Lichtman JH, Krumholz HM. Hospital remoteness and thirty-day mortality from three serious conditions. Health Affairs. 2008 Nov;27(6):1707-17.	wrong intervention
	Ross JS, Normand SL, Wang Y, Ko DT, Chen J, Drye EE, Keenan PS, Lichtman JH, Bueno H, Schreiner GC, Krumholz HM. Hospital volume and 30-day mortality for three common medical conditions. New England	wrong intervention

	Journal of Medicine. 2010 Mar 25;362(12):1110-8.	
	Sari N. Do competition and managed care improve quality?. Health economics. 2002 Oct;11(7):571-84.	wrong intervention
	Shahian DM, Nordberg P, Meyer GS, Blanchfield BB, Mort EA, Torchiana DF, Normand SL. Contemporary performance of US teaching and nonteaching hospitals. Academic Medicine. 2012 Jun 1;87(6):701-8.	wrong intervention
	Volpp KG, Rosen AK, Rosenbaum PR, Romano PS, Even-Shoshan O, Wang Y, Bellini L, Behringer T, Silber JH. Mortality among hospitalized Medicare beneficiaries in the first 2 years following ACGME resident duty hour reform. Jama. 2007 Sep 5;298(9):975-83.	wrong intervention
	Menachemi N, Chukmaitov A, Saunders C, Brooks RG. Hospital quality of care: does information technology matter? The relationship between information technology adoption and quality of care. Health care management review. 2008 Jan 1;33(1):51-9.	wrong intervention
Staplers et al		
	Kendall-Gallagher D, Blegen MA. Competence and certification of registered nurses and safety of patients in intensive care units. American Journal of Critical Care. 2009 Mar;18(2):106-13	ICU setting
	Hall LM, Doran D, Pink GH. Nurse staffing models, nursing hours, and patient safety outcomes. JONA: The Journal of Nursing Administration. 2004 Jan 1;34(1):41-5.	wrong intervention (skill mix)
	Bae SH, Mark B, Fried B. Impact of nursing unit turnover on patient outcomes in hospitals. Journal of Nursing Scholarship. 2010 Mar;42(1):40-9.	wrong intervention (turn over rates)

	Bae SH, Mark B, Fried B. Use of temporary nurses and nurse and patient safety outcomes in acute care hospital units. Health care management review. 2010 Oct 1;35(4):333-44.	wrong intervention (skill mix)
	Chang YK, Hughes LC, Mark B. Fitting in or standing out: Nursing workgroup diversity and unit-level outcomes. Nursing Research. 2006 Nov 1;55(6):373-80.	wrong intervention
	Krapohl G, Manojlovich M, Redman R, Zhang L. Nursing specialty certification and nursing-sensitive patient outcomes in the intensive care unit. American Journal of Critical Care. 2010 Nov;19(6):490-8.	wrong intervention
	Mallidou AA, Cummings GG, Estabrooks CA, Giovannetti PB. Nurse specialty subcultures and patient outcomes in acute care hospitals: A multiple-group structural equation modeling. International journal of nursing studies. 2011 Jan 1;48(1):81-93.	wrong intervention
	Manojlovich M, Antonakos CL, Ronis DL. Intensive care units, communication between nurses and physicians, and patients' outcomes. American Journal of Critical Care. 2009 Jan;18(1):21-30.	wrong intervention
	Manojlovich M, Sidani S, Covell CL, Antonakos CL. Nurse dose: linking staffing variables to adverse patient outcomes. Nursing research. 2011 Jul 1;60(4):214-20.	wrong intervention
	Purdy N, SPENCE LASCHINGER HK, Finegan J, Kerr M, Olivera F. Effects of work environments on nurse and patient outcomes. Journal of nursing management. 2010 Nov;18(8):901-13.	wrong intervention
	Stone PW, Mooney-Kane C, Larson EL, Horan T, Glance LG, Zwanziger J, Dick AW. Nurse working conditions and patient safety outcomes. Medical care. 2007 Jun 1:571-8.	ICU setting

	Wolf D, Lehman L, Quinlin R, Rosenzweig M, Friede S, Zullo T, Hoffman L. Can nurses impact patient outcomes using a patient-centered care model?. JONA: The Journal of Nursing Administration. 2008 Dec 1;38(12):532-40.	ICU setting
Lankshear et al		
	Aiken LH, Smith HL, Lake ET. Lower Medicare mortality among a set of hospitals known for good nursing care. Medical care. 1994 Aug 1:771-87.	wrong intervention (magnet versus non-magnet)
	Jarman B, Gault S, Alves B, Hider A, Dolan S, Cook A, Hurwitz B, Iezzoni LI. Explaining differences in English hospital death rates using routinely collected data. Bmj. 1999 Jun 5;318(7197):1515-20.	wrong intervention (Used other clinical staffing (doctors))
	Hall LM, Doran D, Pink GH. Nurse staffing models, nursing hours, and patient safety outcomes. JONA: The Journal of Nursing Administration. 2004 Jan 1;34(1):41-5.	skill mix
Mitchell et al		
	Andersen BM, Rasch M, Hochlin K, Tollefsen T, Sandvik L. Hospital-acquired infections before and after healthcare reorganization in a tertiary university hospital in Norway. Journal of public health. 2009 Mar 1;31(1):98-104.	wrong intervention (nursing workload)
	Geubbels EL, Wille JC, Nagelkerke NJ, Vandenbroucke-Grauls CM, Grobbee DE, de Boer AS. Hospital-related determinants for surgical-site infection following hip arthroplasty. Infection Control & Hospital Epidemiology. 2005 May;26(5):435-41.	Wrong intervention
	Roche M, Duffield C, Aisbett C, Diers D, Stasa H. Nursing work directions in Australia: Does evidence drive the policy?. Collegian. 2012 Dec 1;19(4):231-8.	wrong intervention (skill mix)

	Kelly D, Kutney-Lee A, Lake ET, Aiken LH. The critical care work environment and nurse-reported health care–associated infections. American Journal of Critical Care. 2013 Nov;22(6):482-8.	ICU setting
	Dimick JB, Swoboda SM, Pronovost PJ, Lipsett PA. Effect of nurse-to-patient ratio in the intensive care unit on pulmonary complications and resource use after hepatectomy. American Journal of Critical Care. 2001 Nov 1;10(6):376.	ICU setting
	Berney B, Needleman J. Impact of nursing overtime on nurse-sensitive patient outcomes in New York hospitals, 1995-2000. Policy, Politics, & Nursing Practice. 2006 May;7(2):87-100.	wrong intervention (nurses overtime)
	Alonso-Echanove J, Edwards JR, Richards MJ, Brennan P, Venezia RA, Keen J, Ashline V, Kirkland K, Chou E, Hupert M, Veeder AV. Effect of nurse staffing and antimicrobial-impregnated central venous catheters on the risk for bloodstream infections in intensive care units. Infection Control & Hospital Epidemiology. 2003 Dec;24(12):916-25.	ICU setting
	Cimiotti JP, Haas J, Saiman L, Larson EL. Impact of staffing on bloodstream infections in the neonatal intensive care unit. Archives of pediatrics & adolescent medicine. 2006 Aug 1;160(8):832-6.	ICU setting
	Pronovost PJ, Dang D, Dorman T, Lipsett PA, Garrett E, Jenckes M, Bass EB. Intensive care unit nurse staffing and the risk for complications after abdominal aortic surgery. Effective clinical practice: ECP. 2001 Sep 1;4(5):199-206.	ICU setting
	Robert J, Fridkin SK, Blumberg HM, Anderson B, White N, Ray SM, Chan J, Jarvis WR. The influence of the	ICU setting

	composition of the nursing staff on primary bloodstream infection rates in a surgical intensive care unit. Infection Control & Hospital Epidemiology. 2000 Jan;21(1):12-7.	
	UK Neonatal Staffing Study Group. Patient volume, staffing, and workload in relation to risk-adjusted outcomes in a random stratified sample of UK neonatal intensive care units: a prospective evaluation. The Lancet. 2002 Jan 12;359(9301):99-107.	ICU setting
	Hugonnet S, Uçkay I, Pittet D. Staffing level: a determinant of late-onset ventilator-associated pneumonia. Critical Care. 2007 Aug;11(4):1-7.	ICU setting
	Bae SH, Brewer CS, Kelly M, Spencer A. Use of temporary nursing staff and nosocomial infections in intensive care units. Journal of clinical nursing. 2015 Apr;24(7-8):980-90.	ICU setting
	Kendall-Gallagher D, Blegen MA. Competence and certification of registered nurses and safety of patients in intensive care units. American Journal of Critical Care. 2009 Mar;18(2):106-13.	ICU setting
	Stone PW, Mooney-Kane C, Larson EL, Horan T, Glance LG, Zwanziger J, Dick AW. Nurse working conditions and patient safety outcomes. Medical care. 2007 Jun 1:571-8.	ICU setting
	Schwab F, Meyer E, Geffers C, Gastmeier P. Understaffing, overcrowding, inappropriate nurse: ventilated patient ratio and nosocomial infections: which parameter is the best reflection of deficits?. Journal of Hospital Infection. 2012 Feb 1;80(2):133-9.	ICU setting
	Amaravadi RK, Dimick JB, Pronovost PJ, Lipsett PA. ICU nurse-to-patient ratio is associated with complications and resource use	ICU setting

	after esophagectomy. Intensive care medicine. 2000 Dec;26(12):1857-62.	
	Dancer SJ, Coyne M, Speekenbrink A, Samavedam S, Kennedy J, Wallace PG. MRSA acquisition in an intensive care unit. American journal of infection control. 2006 Feb 1;34(1):10-7.	ICU setting
	Grundmann H, Hori S, Winter B, Tami A, Austin DJ. Risk factors for the transmission of methicillin-resistant Staphylococcus aureus in an adult intensive care unit: fitting a model to the data. The Journal of infectious diseases. 2002 Feb 15;185(4):481-8.	ICU setting
	Kong F, Cook D, Paterson DL, Whitby M, Clements AC. Do staffing and workload levels influence the risk of new acquisitions of meticillin-resistant Staphylococcus aureus in a well-resourced intensive care unit?. Journal of Hospital Infection. 2012 Apr 1;80(4):331-9.	ICU setting
	Hugonnet S, Villaveces A, Pittet D. Nurse staffing level and nosocomial infections: empirical evaluation of the case-crossover and case-time-control designs. American journal of epidemiology. 2007 Jun 1;165(11):1321-7.	ICU setting
	Rogowski JA, Staiger D, Patrick T, Horbar J, Kenny M, Lake ET. Nurse staffing and NICU infection rates. JAMA pediatrics. 2013 May 1;167(5):444-50.	ICU setting
	Parikh A, Huang SA, Murthy P, Dombrovskiy V, Nolledo M, Lefton R, Scardella AT. Quality improvement and cost savings after implementation of the Leapfrog intensive care unit physician staffing standard at a community teaching hospital. Critical care medicine. 2012 Oct 1;40(10):2754-9.	ICU setting

	Hugonnet S, Chevrolet JC, Pittet D. The effect of workload on infection risk in critically ill patients. Critical care medicine. 2007 Jan 1;35(1):76-81.	ICU setting
	Maillet JM, Guérot E, Novara A, Le Guen J, Lahjibi-Paulet H, Kac G, Diehl JL, Fagon JY. Comparison of intensive-care-unit-acquired infections and their outcomes among patients over and under 80 years of age. Journal of Hospital Infection. 2014 Jul 1;87(3):152-8.	ICU setting
	Daud-Gallotti RM, Costa SF, Guimarães T, Padilha KG, Inoue EN, Vasconcelos TN, da Silva Cunha Rodrigues F, Barbosa EV, Figueiredo WB, Levin AS. Nursing workload as a risk factor for healthcare associated infections in ICU: a prospective study. PloS one. 2012 Dec 27;7(12):e52342.	ICU setting
	The U. Relationship between probable nosocomial bacteraemia and organisational and structural factors in UK neonatal intensive care units. Quality & safety in health care. 2005 Aug;14(4):264.	ICU setting
	Halwani M, Solaymani-Dodaran M, Grundmann H, Coupland C, Slack RJ. Cross-transmission of nosocomial pathogens in an adult intensive care unit: incidence and risk factors. Journal of Hospital Infection. 2006 May 1;63(1):39-46.	ICU setting
	Dorsey G, Borneo HT, Sun SJ, Wells J, Steele L, Howland K, Perdreau-Remington F, Bangsberg DR. A heterogeneous outbreak of Enterobacter cloacae and Serratia marcescens infections in a surgical intensive care unit. Infection Control & Hospital Epidemiology. 2000 Jul;21(7):465-9.	ICU setting
	Fraher MH, Collins CJ, Bourke J, Phelan D, Lynch M. Cost-effectiveness of employing a total	wrong intervention (Addition of a specialist nurse)

	parenteral nutrition surveillance nurse for the prevention of catheter-related bloodstream infections. Journal of Hospital Infection. 2009 Oct 1;73(2):129-34.	
	Barkell NP, Killinger KA, Schultz SD. The relationship between nurse staffing models and patient outcomes: a descriptive study. Outcomes Management. 2002 Jan 1;6(1):27-33.	wrong intervention (skill mix)
	Hall LM, Doran D, Pink GH. Nurse staffing models, nursing hours, and patient safety outcomes. JONA: The Journal of Nursing Administration. 2004 Jan 1;34(1):41-5.	wrong intervention (skill mix)
	Yang PH, Hung CH, Chen YM, Hu CY, Shieh SL. The impact of different nursing skill mix models on patient outcomes in a respiratory care center. Worldviews on Evidence-Based Nursing. 2012 Nov;9(4):227-33.	wrong intervention (skill mix)
	Virtanen M, Kurvinen T, Terho K, Oksanen T, Peltonen R, Vahtera J, Routamaa M, Elovainio M, Kivimäki M. Work hours, work stress, and collaboration among ward staff in relation to risk of hospital-associated infection among patients. Medical care. 2009 Mar 1:310-8.	wrong intervention (overtime)
	Dimick JB, Pronovost PJ, Heitmiller RF, Lipsett PA. Intensive care unit physician staffing is associated with decreased length of stay, hospital cost, and complications after esophageal resection. Critical care medicine. 2001 Apr 1;29(4):753-8.	wrong intervention (Physician staffing)

BMJ Publishing Group Limited (BMJ) disclaims all liability and responsibility arising from any reliance placed on this supplemental material which has been supplied by the author(s)
