# Supplementary material for: Identifying gaps in global evidence for nurse staffing and patient care outcomes research in low/middle-income countries: an umbrella review
Source: BMJ Open. 2022 Oct 12;12(10):e064050. doi: 10.1136/bmjopen-2022-064050 (PMC9562716; doi:10.1136/bmjopen-2022-064050)
Supplement: Supplementary data [file bmjopen-2022-064050supp004.pdf]

Online supplemental file 4: Overlap of primary papers across reviews

Study name	Thungjaroenkul P et al (2007)	Twigg et al (2020)	Assaye et al (2020)	Bourgon et al (2019)	Shin et al (2019)	Wilson et al (2011)	Kane et al (2007)	Griffiths et al (2019)	Lankshear et al (2005)	Engineer et al (2015)	Staplers et al (2015)	Mitchell et al (2018)	Lang et al (2004)	Hill (2017)	Frequency
Aiken LH, Cimiotti JP, Sloane DM, Smith HL, Flynn L, Neff DF. Effects of nurse staffing and nurse education on patient deaths in hospitals with different nurse work environments. The Journal of nursing administration. 2012	No	No	No	Yes	No	No	No	No	No	No	No	No	No	No	1

Oct;42(10 Suppl):S10.															
Aiken LH, Clarke SP, Cheung RB, Sloane DM, Silber JH. Educational levels of hospital nurses and surgical patient mortality. Jama. 2003 Sep 24;290(12):1617-23.	No	No	No	No	No	No	Yes	No	Yes	No	No	No	Yes	No	3
Aiken LH, Clarke SP, Sloane DM, Lake ET, Cheney T. Effects of hospital care environment on patient mortality and nurse outcomes. The Journal	No	No	No	Yes	No	No	No	No	No	No	No	No	No	No	1

of nursing administration. 2008 May;38(5):223.															
Aiken LH, Clarke SP, Sloane DM, Sochalski J, Silber JH. Hospital nurse staffing and patient mortality, nurse burnout, and job dissatisfaction. Jama. 2002 Oct 23;288(16):1987-93.	No	No	No	Yes	No	No	Yes	No	Yes	No	No	No	Yes	No	4
Aiken LH, Clarke SP, Sloane DM. Hospital restructuring: does it adversely affect care and outcomes?. JONA: The	No	No	No	No	No	No	No	No	No	No	No	No	Yes	No	1

Journal of Nursing Administration. 2000 Oct 1;30(10):457-65.															
Aiken LH, Sloane DM, Bruyneel L, Van den Heede K, Griffiths P, Busse R, Diomidous M, Kinnunen J, Kózka M, Lesaffre E, McHugh MD. Nurse staffing and education and hospital mortality in nine European countries: a retrospective observational study. The lancet. 2014 May 24;383(9931):1824-30.	No	No	No	Yes	No	No	No	No	No	No	No	No	No	No	1

Aiken LH, Sloane DM, Lake ET, Sochalski J, Weber AL. Organization and outcomes of inpatient AIDS care. Medical care. 1999 Aug 1:760-72.	No	No	No	No	No	No	Yes	No	No	No	No	No	No	No	1
Al-Kandari F, Thomas D. Factors contributing to nursing task incompletion as perceived by nurses working in Kuwait general hospitals. Journal of clinical nursing. 2009 Dec;18(24):3430-40.	No	No	No	No	No	No	No	Yes	No	No	No	No	No	No	1

American Nurses Association. Implementing nursing's report card: A study of RN staffing, length of stay, and patient outcomes. Amer Nurses Assn; 1997.	No	No	No	No	No	No	Yes	No	No	No	No	No	No	No	1
American Nurses Association. Nurse staffing and patient outcomes in the inpatient hospital setting. Amer Nurses Assn; 2000.	No	No	No	No	No	No	No	No	No	No	No	No	Yes	No	1
Arbesman MC, Wright C. Mechanical restraints,	No	No	No	No	No	No	No	No	No	No	No	No	Yes	No	1

rehabilitation therapies, and staffing adequacy as risk factors for falls in an elderly hospitalized population. Rehabilitation Nursing. 1999 May 6;24(3):122-8.															
Ausserhofer, D., Zander, B., Busse, R., Schubert, M., De Geest, S., & Rafferty, A. M., ... Consortium, R. C. (2014). Prevalence, patterns and predictors of nursing care left undone in European hospitals: Results from the multicountr	No	No	No	No	No	No	No	Yes	No	No	No	No	No	No	1

y cross-sectional RN4CAST study. BMJ Quality & Safety, 23(2), 126–135. https://doi.org/10.1136/bmjqs2013-002318														
Ball JE, Bruyneel L, Aiken LH, Sermeus W, Sloane DM, Rafferty AM, Lindqvist R, Tishelman C, Griffiths P, RN4Cast Consortium. Post-operative mortality, missed care and nurse staffing in nine countries: a cross-sectional study.	No	No	No	Yes	No	No	No	No	No	No	No	No	No	No	1

International journal of nursing studies. 2018 Feb 1;78:10-5.															
Ball, J. E., Griffiths, P., Rafferty, A. M., Lindqvist, R., Murrells, T., & Tishelman, C. (2016). A cross-sectional study of 'care left undone'on nursing shifts in hospitals. Journal of Advanced Nursing, 72, 2086–2097. https://doi.org/10.1111/jan.12976	No	No	No	No	No	No	No	Yes	No	No	No	No	No	No	1
Ball, J. E., Murrells, T., Rafferty, A. M., Morrow,	No	No	No	No	No	No	No	Yes	No	No	No	No	No	No	1

E., & Griffiths, P. (2014). 'Care left undone' during nursing shifts: Associations with workload and perceived quality of care. BMJ Quality & Safety, 23(2), 116–125. https://doi.org/10.1136/bmjqs-2012-001767															
Behner KG, Fogg LF, Fournier LC, Frankenbach JT, Robertson SB. Nursing resource management: analyzing the relationship	Yes	No	No	No	No	No	No	No	No	No	No	No	No	No	1

between costs and quality in staffing decisions. Health Care Management Review. 1990 Jan 1;15(4):63-71.														
Berney B, Needleman J. Impact of nursing overtime on nurse-sensitive patient outcomes in New York hospitals, 1995-2000. Policy, Politics, & Nursing Practice. 2006 May;7(2):87-100.	No	No	No	Yes	No	No	No	No	No	No	No	No	No	No	1
Blegen MA, Goode CJ, Reed L. Nurse	No	No	No	No	No	No	No	No	No	No	No	No	Yes	No	1

staffing and patient outcomes. Nursing research. 1998 Jan 1;47(1):43-50.															
Blegen MA, Goode CJ, Spetz J, Vaughn T, Park SH. Nurse staffing effects on patient outcomes: safety-net and non-safety-net hospitals. Medical care. 2011 Apr 1:406-14.	No	No	No	Yes	No	No	No	No	No	No	No	No	No	No	1
Blegen MA, Vaughn T. A multisite study of nurse staffing and patient occurrences.	No	No	No	No	No	No	No	No	Yes	No	No	No	Yes	No	2

Nursing Economics. 1998 Jul 1;16(4):196.															
Bond CA, Raehl CL, Franke T. Medication errors in United States hospitals. Pharmacotherapy: The Journal of Human Pharmacology and Drug Therapy. 2001 Sep;21(9):1023-36.	No	No	No	No	No	No	No	No	Yes	No	No	No	No	No	1
Bond CA, Raehl CL, Pitterle ME, Franke T. Health care professional staffing, hospital characteristics, and hospital mortality	No	No	No	No	No	No	No	No	Yes	No	No	No	Yes	No	2

rates. Pharmacotherapy: The Journal of Human Pharmacology and Drug Therapy. 1999 Feb;19(2):130-8.														
Breckenridge-Sproat S, Johantgen M, Patrician P. Influence of unit-level staffing on medication errors and falls in military hospitals. Western journal of nursing research. 2012 Jun;34(4):455-74.	No	No	No	No	No	No	No	No	No	No	Yes	No	No	No	1
Brooks Carthon JM, Kutney-Lee A, Jarrín O,	No	No	No	Yes	No	No	No	No	No	No	No	No	No	No	1

Sloane D, Aiken LH. Nurse staffing and postsurgical outcomes in black adults. Journal of the American Geriatrics Society. 2012 Jun;60(6):1078-84.														
Bruyneel, L., Li, B., Ausserhofer, D., Lesaffre, E., Dumitrescu, I., Smith, H. L., & Sermeus, W. (2015). Organization of hospital nursing, provision of nursing care and patient experiences with care in Europe.	No	No	No	No	No	No	No	Yes	No	No	No	No	No	No	1

Medical Care Research and Review, 72(6), 643–664. https://doi.org/10.1177/1077558715589188														
Burnes Bolton L, Aydin CE, Donaldson N, Storer Brown D, Sandhu M, Fridman M, Udin Aronow H. Mandated nurse staffing ratios in California: a comparison of staffing and nursing-sensitive outcomes pre-and postregulation. Policy, Politics, &	No	Yes	No	No	No	No	No	No	No	No	Yes	No	No	No	2

Nursing Practice. 2007 Nov;8(4):238-50.															
Carter JH, Mills AC, Homan SM, Blaesing SL, Heater BS, Stoll LD, Mornin C, Corrigan MK. Correlating the quality of care with nursing resources and patient parameters: a longitudinal study. NLN publications. 1987 Dec(20-2191):331-45.													Yes	No	1
Cheung RB. The relationship between nurse	No	No	No	No	No	No	Yes	No	No	No	No	No	No	No	1

staffing, nursing time, and adverse events in an acute care hospital. University of South Florida; 2002.															
Cho E, Lee NJ, Kim EY, Kim S, Lee K, Park KO, Sung YH. Nurse staffing level and overtime associated with patient safety, quality of care, and care left undone in hospitals: a cross-sectional study. Internationa l journal of nursing	No	No	No	No	No	N o	No	Yes	No	No	No	No	No	No	1

studies. 2016 Aug 1;60:263-71.														
Cho E, Sloane DM, Kim EY, Kim S, Choi M, Yoo IY, Lee HS, Aiken LH. Effects of nurse staffing, work environments, and education on patient mortality: an observational study. International journal of nursing studies. 2015 Feb 1;52(2):535-42.	No	No	No	Yes	No	No	No	No	No	No	No	No	No	No	1

Cho SH, Ketefian S, Barkauskas VH, Smith DG. The effects of nurse staffing on adverse events, morbidity, mortality, and medical costs. Nursing research. 2003 Mar 1;52(2):71-9.	Yes	No	No	No	No	No	Yes	No	Yes	No	No	No	Yes	No	4
Cho SH, Kim YS, Yeon KN, You SJ, Lee ID. Effects of increasing nurse staffing on missed nursing care. International nursing review. 2015	No	No	No	No	No	No	No	Yes	No	No	No	No	No	No	1

Jun;62(2):267-74.														
Christine K, Cheryl J, Chunliu Z, Peter JG, Jayasree B. Nurse staffing and postsurgical adverse events: an analysis of administrative data from a sample of US hospitals, 1990–1996. Health services research. 2002 Jun;37(3):611-29.	No	No	No	No	No	No	No	No	Yes	No	No	Yes	Yes	No	3
Cimiotti JP, Aiken LH, Sloane DM, Wu ES. Nurse staffing, burnout, and health	No	No	No	No	No	No	No	No	No	No	No	Yes	No	No	1

care–associated infection. American journal of infection control. 2012 Aug 1;40(6):486-90.															
Cook A, Gaynor M, Stephens Jr M, Taylor L. The effect of a hospital nurse staffing mandate on patient health outcomes: Evidence from California's minimum staffing regulation. Journal of Health Economics. 2012 Mar 1;31(2):340-8.	No	Yes	No	No	No	No	No	No	No	No	No	No	No	No	1

Dabney BW, Kalisch BJ. Nurse staffing levels and patient-reported missed nursing care. Journal of nursing care quality. 2015 Oct 1;30(4):306-12.	No	No	No	No	No	No	No	Yes	No	No	No	No	No	No	1
Dobal MT. The relationships among the context, structure, and performance of nursing units in hospitals (Doctoral dissertation, The University of Texas at Austin).	No	No	No	No	No	No	No	No	No	No	No	No	Yes	No	1

Donaldson N, Bolton LB, Aydin C, Brown D, Elashoff JD, Sandhu M. Impact of California's licensed nurse-patient ratios on unit-level nurse staffing and patient outcomes. Policy, Politics, & Nursing Practice. 2005 Aug;6(3):198-210.	No	Yes	No	No	No	No	No	No	No	No	Yes	No	No	No	2
Dunton N, Gajewski B, Taunton RL, Moore J. Nurse staffing and patient falls on acute care hospital units.	No	No	No	No	Yes	No	No	No	No	No	No	No	No	No	1

Nursing outlook. 2004 Feb 1;52(1):53-9.														
Elkassabany NM, Passarella M, Mehta S, Liu J, Neuman MD. Hospital characteristics, inpatient processes of care, and readmissions of older adults with hip fractures. Journal of the American Geriatrics Society. 2016 Aug;64(8):1656-61.	No	No	No	Yes	No	No	No	No	No	No	No	No	No	No	1
Elting LS, Pettaway C, Bekele BN, Grossman	No	No	No	No	No	No	Yes	No	No	No	No	No	No	No	1

HB, Cooksley C, Avritscher EB, Saldin K, Dinney CP. Correlation between annual volume of cystectomy, professional staffing, and outcomes: a statewide, population-based study. Cancer: Interdisciplinary International Journal of the American Cancer Society. 2005 Sep 1;104(5):975-84.														
Everhart, D. M., Schumacher, J. R., Duncan, R. P., Hall, A.	No	No	No	No	Yes	No	No	No	No	No	No	No	No	No	1

G., Neff, D. F., & Shorr, R. I. (2014). Determinants of hospital fall rate trajectory groups: A longitudinal assessment of nurse staffing and organiza-tional characteristics. Health Care Management Review, 39(4), 352–360														
Feleke SA, Mulatu MA, Yesmaw YS. Medication administration error: magnitude and associated factors among nurses in Ethiopia.	No	No	Yes	No	No	No	No	No	No	No	No	No	No	No	1

BMC nursing. 2015 Dec;14(1):1-8.															
Flood SD, Diers D. Nurse staffing, patient outcome and cost. Nursing Management. 1988 May 1;19(5):34-45.	No	No	No	No	No	No	No	No	No	No	No	No	Yes	No	1
Friese CR, Kalisch BJ, Lee KH. Patterns and correlates of missed nursing care in inpatient oncology units. Cancer nursing. 2013 Nov;36(6):E51.	No	No	No	No	No	No	No	Yes	No	No	No	No	No	No	1

Friese CR, Lake ET, Aiken LH, Silber JH, Sochalski J. Hospital nurse practice environments and outcomes for surgical oncology patients. Health services research. 2008 Aug;43(4):1145-63.	No	No	No	Yes	No	No	No	No	No	No	No	No	No	No	1
Frith KH, Anderson EF, Caspers B, Tseng F, Sanford K, Hoyt NG, Moore K. Effects of nurse staffing on hospital-acquired conditions and length	No	No	No	No	Yes	No	No	No	No	No	Yes	No	No	No	2

of stay in community hospitals. Quality Management in Healthcare. 2010 Apr 1;19(2):147-55.														
Ghaferi AA, Osborne NH, Birkmeyer JD, Dimick JB. Hospital characteristics associated with failure to rescue from complications after pancreatectomy. Journal of the American College of Surgeons. 2010 Sep 1;211(3):325-30.	No	No	No	Yes	No	No	No	No	No	No	No	No	No	No	1

Glance LG, Dick AW, Osler TM, Mukamel DB, Li Y, Stone PW. The association between nurse staffing and hospital outcomes in injured patients. BMC health services research. 2012 Dec;12(1):1-8	No	No	No	No	No	No	No	No	No	No	No	Yes	No	No	1
Goode CJ, Blegen MA, Park SH, Vaughn T, Spetz J. Comparison of patient outcomes in Magnet® and non-Magnet hospitals. JONA: The	No	No	No	No	No	No	No	No	No	No	Yes	No	No	No	1

Journal of Nursing Administration. 2011 Dec 1;41(12):517-23.															
Griffiths P, Dall'Ora C, Simon M, Ball J, Lindqvist R, Rafferty AM, Schoonhoven L, Tishelman C, Aiken LH. Nurses' shift length and overtime working in 12 European countries: the association with perceived quality of care and patient safety. Medical care. 2014	No	No	No	No	No	No	No	Yes	No	No	No	No	No	No	1

Nov;52(11): 975.														
Griffiths P, Jones S, Bottle A. Is "failure to rescue" derived from administrative data in England a nurse sensitive patient safety indicator for surgical care? Observational study. International Journal of Nursing Studies. 2013 Feb 1;50(2):292-300.	No	No	No	Yes	No	No	No	No	No	No	No	No	No	No	1

Gunningberg L, Donaldson N, Aydin C, Idvall E. Exploring variation in pressure ulcer prevalence in Sweden and the USA: benchmarking in action. Journal of evaluation in clinical practice. 2012 Aug;18(4):904-10.	No	No	No	No	No	No	No	No	No	No	Yes	No	No	No	1
Haley RW, Bregman DA. The role of understaffing and overcrowding in recurrent outbreaks of staphylococcal infection	No	No	No	No	No	No	No	No	No	No	No	No	Yes	No	1

in a neonatal special-care unit. Journal of Infectious Diseases. 1982 Jun 1;145(6):875-85.														
Halm M, Peterson M, Kandels M, Sabo J, Blalock M, Braden R, Gryczman A, Krisko-Hagel K, Larson D, Lemay D, Sisler B. Hospital nurse staffing and patient mortality, emotional exhaustion, and job dissatisfaction. Clinical Nurse Specialist. 2005 Sep	No	No	No	Yes	No	No	Yes	No	No	No	No	No	No	No	2

1;19(5):241-51.															
Halm M, Peterson M, Kandels M, Sabo J, Blalock M, Braden R, Gryczman A, Krisko-Hagel K, Larson D, Lemay D, Sisler B. Hospital nurse staffing and patient mortality, emotional exhaustion, and job dissatisfaction. Clinical Nurse Specialist. 2005 Sep 1;19(5):241-51.	No	No	No	No	No	No	No	No	No	No	No	No	No	Yes	1
Harless DW, Mark BA. Nurse staffing and	No	No	No	Yes	No	No	No	No	No	No	No	No	No	No	1

quality of care with direct measurement of inpatient staffing. Medical care. 2010 Jul 1:659-63.														
Hartz AJ, Krakauer H, Kuhn EM, Young M, Jacobsen SJ, Gay G, Muenz L, Katzoff M, Bailey RC, Rimm AA. Hospital characteristics and mortality rates. New England journal of medicine. 1989 Dec 21;321(25):1720-5.	No	No	No	No	No	No	No	No	No	No	No	No	Yes	No	1

Hickey, P. A., Gauvreau, K., Jenkins, K., Fawcett, J., & Hayman, L. (2011). Statewide and national impact of California's staffing law on pediatric cardiac surgery outcomes. JONA: the Journal of Nursing Administration, 41(5), 218–225.	No	Yes	No	No	No	No	No	No	No	No	No	No	No	No	1
Hinno S, Partanen P, Vehviläinen-Julkunen K. Nursing activities, nurse staffing and adverse patient outcomes as	No	No	No	No	Yes	No	No	No	No	No	No	No	No	No	1

perceived by hospital nurses. Journal of clinical nursing. 2012 Jun;21(11-12):1584-93.															
Hope J. Nosocomial Infections and Their Relationship to Nursing Workload in an Acute Care Hospital dissertation. Ottawa, Ontario, Canada: Queen's University. 2003.	No	No	No	No	No	N o	Yes	No	No	No	No	No	No	No	1
Houser EP. Nurse staffing levels and patient outcomes. The Johns Hopkins	No	No	No	No	No	N o	Yes	No	No	No	No	No	No	No	1

University; 2005.															
Jiang HJ, Stocks C, Wong CJ. Disparities between two common data sources on hospital nurse staffing. Journal of Nursing Scholarship. 2006 Jun;38(2):187-93.	No	No	No	No	No	No	No	No	No	No	Yes	No	No	No	1
Kalisch BJ, Doumit M, Lee KH, El Zein J. Missed nursing care, level of staffing, and job satisfaction: Lebanon versus the United	No	No	No	No	No	No	No	Yes	No	No	No	No	No	No	1

States. JONA: The Journal of Nursing Administration. 2013 May 1;43(5):274-9.														
Kalisch BJ, Tschannen D, Lee H, Friese CR. Hospital variation in missed nursing care. American Journal of Medical Quality. 2011 Jul;26(4):291-9.	No	No	No	No	No	No	No	Yes	No	No	No	No	No	No	1
Kalisch BJ, Tschannen D, Lee KH. Do staffing levels predict missed nursing care?.	No	No	No	No	No	No	No	Yes	No	No	No	No	No	No	1

International Journal for Quality in Health Care. 2011 Jun 1;23(3):302-8.														
Kim CG, Bae KS. Relationship between nurse staffing level and adult nursing-sensitive outcomes in tertiary hospitals of Korea: Retrospective observational study. International Journal of Nursing Studies. 2018 Apr 1;80:155-64.	No	No	No	No	Yes	No	No	No	No	No	No	No	No	No	1

Kim Y, Han K. Longitudinal associations of nursing staff turnover with patient outcomes in long-term care hospitals in Korea. Journal of Nursing Management. 2018 Jul;26(5):518-24.	No	No	No	No	Yes	No	No	No	No	No	No	No	No	No	1
Kouatly IA, Nassar N, Nizam M, Badr LK. Evidence on nurse staffing ratios and patient outcomes in a low-income country: Implications for future	No	No	Yes	No	Yes	No	No	No	No	No	No	No	No	No	2

research and practice. Worldviews on Evidence-Based Nursing. 2018 Oct;15(5):353-60.															
Kovner C, Gergen PJ. Nurse staffing levels and adverse events following surgery in US hospitals. Image: The Journal of Nursing Scholarship. 1998 Dec;30(4):315-21.	No	No	No	No	No	No	No	No	Yes	No	No	No	Yes	No	2
Kutney-Lee A, Aiken LH. Effect of nurse staffing and education	No	No	No	Yes	No	No	No	No	No	No	No	No	No	No	1

on the outcomes of surgical patients with comorbid serious mental illness. Psychiatric Services. 2008 Dec;59(12):1466-9.														
Li X, Bowman SM, Smith TC. Effects of registered nurse staffing level on hospital-acquired conditions in cardiac surgery patients: a propensity score matching analysis. Nursing outlook. 2016 Nov	No	No	No	Yes	No	No	No	No	No	No	No	No	No	No	1

1;64(6):533-41.														
Lichtig LK, Knauf RA, Milholland DK. Some impacts of nursing on acute care hospital outcomes. JONA: The Journal of Nursing Administration. 1999 Feb 1;29(2):25-33.	Yes	No	No	No	No	No	Yes	No	Yes	No	No	No	No	No	3
Magalhães AM, Dall'Agnol CM, Marck PB. Nursing workload and patient safety-a mixed method study with an	No	No	Yes	No	No	No	No	No	No	No	No	No	No	No	1

ecological restorative approach. Revista latino-americana de enfermagem. 2013;21:146-54.															
Manheim LM, Feinglass J, Shortell SM, Hughes EF. Regional variation in Medicare hospital mortality. Inquiry. 1992 Apr 1:55-66.	No	No	No	No	No	No	No	No	Yes	No	No	No	Yes	No	2
Manojlovich M, Sidani S, Covell CL, Antonakos CL. Nurse dose: linking staffing variables to adverse patient	No	No	No	No	No	No	No	No	No	No	No	Yes	No	No	1

outcomes. Nursing research. 2011 Jul 1;60(4):214-20.														
Mark BA, Harless DW, Berman WF. Nurse staffing and adverse events in hospitalized children. Policy, Politics, & Nursing Practice. 2007 May;8(2):83-92.	No	No	No	Yes	No	Yes	No	No	No	No	No	Yes	No	No	3
Mark BA, Harless DW, McCue M, Xu Y. A longitudinal examination of hospital registered nurse staffing and quality of care. Health	No	No	No	No	Yes	No	Yes	No	Yes	No	Yes	Yes	No	No	5

services research. 2004 Apr;39(2):279-300.															
Mark BA, Harless DW, McCue M. The impact of HMO penetration on the relationship between nurse staffing and quality. Health economics. 2005 Jul;14(7):737-53.	No	No	No	No	No	Yes	No	No	No	No	No	No	No	No	1
Mark BA, Harless DW, Spetz J, Reiter KL, Pink GH. California's minimum nurse staffing legislation: results from a natural	No	Yes	No	No	No	No	No	No	No	No	No	No	No	No	1

experiment. Health services research. 2013 Apr;48(2pt1):435-54.															
Mark BA, Harless DW. Nurse staffing and post-surgical complications using the present on admission indicator. Research in nursing & health. 2010 Feb;33(1):35-47.	No	No	No	No	Yes	No	No	No	No	No	No	Yes	No	No	2
McCloskey BA, Diers DK. Effects of New Zealand's health reengineering on nursing and patient outcomes. Medical care. 2005	No	No	No	Yes	No	No	No	No	No	No	Yes	No	No	No	2

Nov 1:1140-6.														
McHugh MD, Rochman MF, Sloane DM, Berg RA, Mancini ME, Nadkarni VM, Merchant RM, Aiken LH, American Heart Association's Get With The Guidelines-Resuscitation Investigators. Better nurse staffing and nurse work environments associated with increased	No	No	No	No	No	No	No	No	No	No	No	No	No	Yes	1

survival of in-hospital cardiac arrest patients. Medical care. 2016 Jan;54(1):74 .															
Morita K, Matsui H, Fushimi K, Yasunaga H. Association between nurse staffing and in-hospital bone fractures: a retrospective cohort study. Health services research. 2017 Jun;52(3):1005-23.	No	No	No	No	Yes	No	No	No	No	No	No	No	No	No	1
Needleman J, Buerhaus P, Mattke S, Stewart M, Zelevinsky K.	Yes	No	No	Yes	No	No	Yes	No	Yes	No	No	Yes	No	Yes	6

Nurse-staffing levels and the quality of care in hospitals. New England Journal of Medicine. 2002 May 30;346(22):1715-22.															
Needleman J, Buerhaus PI, Mattke S, Stewart M, Zelevinsky K. Measuring Hospital Quality: Can Medicare Data Substitute for All-Payer Data?. Health services research. 2003 Dec;38(6p1):1487-508.	No	No	No	No	No	No	No	No	No	No	No	Yes	No	No	1

Needleman J, Buerhaus PI, Mattke S, Stewart M, Zelevinsky K. Nurse staffing and patient outcomes in hospitals. Final report. Rockville, MD: Health Resources and Services Administration. 2001 Feb 28.	No	No	No	No	No	No	No	No	No	No	No	Yes	No	No	1
Neff DF, Cimiotti J, Sloane DM, Aiken LH. Utilization of non-US educated nurses in US hospitals: implications for hospital mortality. International Journal for Quality in Health Care.	No	No	No	Yes	No	No	No	No	No	No	No	No	No	No	1

2013 Sep 1;25(4):366-72.														
Orique SB, Patty CM, Woods E. Missed nursing care and unit-level nurse workload in the acute and post-acute settings. Journal of nursing care quality. 2016 Jan 1;31(1):84-9.	No	No	No	No	No	No	No	Yes	No	No	No	No	No	No	1
Palese A, Ambrosi E, Prosperi L, Guarnier A, Barelli P, Zambiasi P, Allegrini E, Bazoli L, Casson P, Marin M, Padovan M. Missed	No	No	No	No	No	No	No	Yes	No	No	No	No	No	No	1

nursing care and predicting factors in the Italian medical care setting. Internal and emergency medicine. 2015 Sep;10(6):693-702.														
Pappas S, Davidson N, Woodard J, Davis J, Welton JM. Risk-adjusted staffing to improve patient value. Nursing Economics. 2015 Mar 1;33(2):73.	No	No	No	No	No	No	No	No	No	No	No	Yes	No	No	1
Patrician PA, Loan L, McCarthy M, Fridman M, Donaldson	No	No	No	No	No	No	No	No	No	No	Yes	No	No	No	1

N, Bingham M, Brosch LR. The association of shift-level nurse staffing with adverse patient events. JONA: the Journal of Nursing Administration. 2011 Feb 1;41(2):64-70.														
Patrician PA, McCarthy MS, Swiger P, Raju D, Breckenridge-Sproat S, Su X, Randall KH, Loan LA. Association of temporal variations in staffing with hospital-acquired pressure injury in	No	No	No	No	Yes	No	No	No	No	No	No	No	No	No	1

military hospitals. Research in nursing & health. 2017 Apr;40(2):111-9.															
Person SD, Allison JJ, Kiefe CI, Weaver MT, Williams OD, Centor RM, Weissman NW. Nurse staffing and mortality for Medicare patients with acute myocardial infarction. Medical care. 2004 Jan 1:4-12.	No	No	No	No	No	No	Yes	No	Yes	No	No	No	No	No	2
Prot S, Fontan JE, Alberti C, Bourdon O, Farnoux C, Macher MA, Foureau A, Faye A,	No	No	No	No	No	Yes	No	No	No	No	No	No	No	No	1

Beaufils F, Gottot S, Brion F. Drug administration errors and their determinants in pediatric in-patients. International Journal for Quality in Health Care. 2005 Oct 1;17(5):381-9.														
Rafferty AM, Clarke SP, Coles J, Ball J, James P, McKee M, Aiken LH. Outcomes of variation in hospital nurse staffing in English hospitals: cross-sectional analysis of survey data	No	No	No	Yes	No	No	No	No	No	No	No	No	No	Yes	2

and discharge records. International journal of nursing studies. 2007 Feb 1;44(2):175-82.														
Razmus I. Factors associated with pediatric hospital-acquired pressure injuries. Journal of Wound, Ostomy and Continence Nursing. 2018 Mar 1;45(2):107-16.	No	No	No	No	Yes	No	No	No	No	No	No	No	No	No	1
Richet HM, Benbachir M, Brown DF, Giamarellou H, Gould I, Gubina M,	No	No	No	No	No	No	No	No	No	No	No	Yes	No	No	1

Heczko P, Kalenic S, Pana M, Pittet D, Redjeb SB. Are there regional variations in the diagnosis, surveillance, and control of methicillin-resistant Staphylococcus aureus?. Infection Control & Hospital Epidemiology. 2003 May;24(5):334-41.														
Robertson RH, Hassan M. Staffing intensity, skill mix and mortality outcomes: the case of chronic obstructive	No	No	No	No	No	No	Yes	No	No	No	No	No	No	No	1

lung disease. Health Services Management Research. 1999 Nov;12(4):258-68.														
Robertson RH, Hassan M. Staffing intensity, skill mix and mortality outcomes: the case of chronic obstructive lung disease. Health Services Management Research. 1999 Nov;12(4):258-68.	No	No	No	No	No	No	No	No	No	No	No	No	Yes	No	1
Sasichay-Akkadechanunt T, Scalzi CC, Jawad AF. The relationship	No	No	Yes	No	No	No	No	No	No	No	No	No	No	No	1

between nurse staffing and patient outcomes. JONA: The Journal of Nursing Administration. 2003 Sep 1;33(9):478-85.														
Schreuders LW, Bremner AP, Geelhoed E, Finn J. The relationship between nurse staffing and inpatient complications. Journal of advanced nursing. 2015 Apr;71(4):800-12.	No	No	No	Yes	No	No	No	No	No	No	No	No	No	No	1
Schubert M, Ausserhofer D, Desmedt M,	No	No	No	No	No	No	No	Yes	No	No	No	No	No	No	1

Schwendimann R, Lesaffre E, Li B, De Geest S. Levels and correlates of implicit rationing of nursing care in Swiss acute care hospitals—a cross sectional study. International journal of nursing studies. 2013 Feb 1;50(2):230-9.															
Schultz MA, van Servellen G, Chang BL, McNeese-Smith D, Waxenberg E. The relationship of hospital structural and financial	Yes	No	No	No	No	No	No	No	No	No	No	No	No	No	1

characteristics to mortality and length of stay in acute myocardial infarction patients. Outcomes Management for Nursing Practice. 1998 Jul 1;2(3):130-6.														
Seago JA, Williamson A, Atwood C. Longitudinal analyses of nurse staffing and patient outcomes: more about failure to rescue. JONA: The Journal of Nursing Administration. 2006 Jan	No	No	No	No	No	No	No	No	No	No	Yes	No	No	No	1

1;36(1):13-21.														
Shamian J, Hagen B, Hu TW, Fogarty TE. The relationship between length of stay and required nursing care hours. The Journal of nursing administration. 1994 Jul 1;24(7-8):52-8.	No	No	No	No	No	No	No	No	No	No	No	No	Yes	No	1
Shuldham C, Parkin C, Firouzi A, Roughton M, Lau-Walker M. The relationship between nurse staffing and patient outcomes: a	No	No	No	No	Yes	No	No	No	No	No	Yes	No	No	Yes	3

case study. International journal of nursing studies. 2009 Jul 1;46(7):986-92.														
Silber JH, Kennedy SK, Even-Shoshan O, Chen W, Koziol LF, Showan AM, Longnecker DE. Anesthesiologist direction and patient outcomes. The Journal of the American Society of Anesthesiologists. 2000 Jul 1;93(1):152-63.	No	No	No	No	No	No	Yes	No	No	No	No	No	No	No	1
Silber JH, Kennedy SK, Even-	No	No	No	No	No	No	No	No	Yes	No	No	No	No	No	1

Shoshan O, Chen W, Koziol LF, Showan AM, Longnecker DE. Anesthesiologist direction and patient outcomes. The Journal of the American Society of Anesthesiologists. 2000 Jul 1;93(1):152-63.															
Silber JH, Rosenbaum PR, Ross RN. Comparing the contributions of groups of predictors: which outcomes vary with hospital rather than	No	No	No	No	No	No	No	No	Yes	No	No	No	No	No	1

patient characteristics?. Journal of the American Statistical Association. 1995 Mar 1;90(429):7-18.															
Silber JH, Rosenbaum PR, Schwartz JS, Ross RN, Williams SV. Evaluation of the complication rate as a measure of quality of care in coronary artery bypass graft surgery. Jama. 1995 Jul 26;274(4):317-23.	No	No	No	No	No	No	No	No	No	No	No	No	Yes	No	1
Sochalski J, Konetzka RT, Zhu J, Volpp K. Will	No	No	No	Yes	No	No	No	No	No	Yes	No	No	No	No	2

mandated minimum nurse staffing ratios lead to better patient outcomes?. Medical care. 2008 Jun 1:606-13.															
Sovie M. Hospital Restructuring's Impact on Outcomes: Report to Participating Hospitals.	No	No	No	No	No	No	No	No	No	No	No	No	Yes	No	1
Sovie MD, Jawad AF. Hospital restructuring and its impact on outcomes: nursing staff regulations are premature. JONA: The Journal of	No	No	No	No	No	No	No	No	Yes	No	No	No	No	No	1

Nursing Administration. 2001 Dec 1;31(12):588-600.															
Spetz J, Harless DW, Herrera CN, Mark BA. Using minimum nurse staffing regulations to measure the relationship between nursing and hospital quality of care. Medical Care Research and Review. 2013 Aug;70(4):380-99.	No	Yes	No	No	No	No	No	No	No	No	No	No	No	No	1
Staggs VS, Knight JE, Dunton N. Understandi	No	No	No	No	Yes	No	No	No	No	No	No	No	No	No	1

ng unassisted falls: effects of nurse staffing level and nursing staff characteristics. Journal of Nursing Care Quality. 2012 Jul 1;27(3):194-9.														
Stegenga J, Bell E, Matlow A. The role of nurse understaffing in nosocomial viral gastrointestinal infections on a general pediatrics ward. Infection Control & Hospital Epidemiolog	No	No	No	No	No	Yes	No	No	No	No	No	Yes	No	No	2

y. 2002 Mar;23(3):133-6.														
Stratton KM. Pediatric nurse staffing and quality of care in the hospital setting. Journal of nursing care quality. 2008 Apr 1;23(2):105-14.	No	No	No	No	No	Yes	No	No	No	No	No	Yes	No	No	2
Sujijantararat R, Booth RZ, Davis LL. Nosocomial urinary tract infection: nursing-sensitive quality indicator in a Thai hospital. Journal of nursing care quality. 2005 Apr	No	No	No	No	No	No	No	No	No	No	No	Yes	No	No	1

1;20(2):134-9.															
Sujijantararat R. Nursing-sensitive quality indicators: Nosocomial urinary tract infection in a Thai hospital. The University of Alabama at Birmingham; 2001.	No	No	Yes	No	No	No	No	No	No	No	No	No	No	No	1
Taunton RL, Kleinbeck SV, Stafford R, Woods CQ, Bott MJ. Patient outcomes. Are they linked to registered nurse absenteeism, separation, or work load?. The	No	No	No	No	No	No	No	No	No	No	No	No	Yes	No	1

Journal of nursing administration. 1994 Apr 1;24(4 Suppl):48-55.														
Taylor JA, Dominici F, Agnew J, Gerwin D, Morlock L, Miller MR. Do nurse and patient injuries share common antecedents? An analysis of associations with safety climate and working conditions. BMJ quality & safety. 2012 Feb 1;21(2):101-11.	No	No	No	No	No	No	No	No	No	No	Yes	No	No	No	1

Titler MG, Shever LL, Kanak MF, Picone DM, Qin R. Factors associated with falls during hospitalization in an older adult population. Research and Theory for Nursing Practice. 2011 Jan 1;25(2):127-52.	No	No	No	No	No	No	No	No	No	No	Yes	No	No	No	1
Tourangeau AE, Giovannetti P, Tu JV, Wood M. Nursing-related determinants of 30-day mortality for hospitalized patients. Canadian Journal of	No	No	No	No	No	No	No	No	Yes	No	No	No	No	No	1

Nursing Research Archive. 2002.														
Trinkoff AM, Johantgen M, Storr CL, Gurses AP, Liang Y, Han K. Nurses' work schedule characteristics, nurse staffing, and patient mortality. Nursing research. 2011 Jan 1;60(1):1-8.	No	No	No	No	No	No	No	No	No	Yes	No	No	No	No	1
Twigg D, Duffield C, Bremner A, Rapley P, Finn J. The impact of the nursing hours per patient day (NHPPD) staffing method on patient	No	Yes	No	Yes	No	No	No	No	No	No	Yes	Yes	No	No	4

outcomes: a retrospective analysis of patient and staffing data. International journal of nursing studies. 2011 May 1;48(5):540-8.															
Unruh L. Licensed nurse staffing and adverse events in hospitals. Medical care. 2003 Jan 1:142-52.	No	No	No	No	Yes	No	No	No	Yes	No	No	Yes	Yes	No	4
Unruh LY, Zhang NJ. Nurse staffing and patient safety in hospitals: new variable and longitudinal	No	No	No	No	No	No	No	No	No	No	Yes	Yes	No	No	2

approaches. Nursing research. 2012 Jan 1;61(1):3-12.														
Van den Heede K, Lesaffre E, Diya L, Vleugels A, Clarke SP, Aiken LH, Sermeus W. The relationship between inpatient cardiac surgery mortality and nurse numbers and educational level: analysis of administrative data. International journal of nursing studies. 2009 Jun	No	No	No	Yes	No	No	No	No	No	No	No	No	No	No	1

1;46(6):796-803.															
Van den Heede K, Sermeus W, Diya L, Clarke SP, Lesaffre E, Vleugels A, Aiken LH. Nurse staffing and patient outcomes in Belgian acute hospitals: cross-sectional analysis of administrative data. International journal of nursing studies. 2009 Jul 1;46(7):928-39.	No	No	No	Yes	No	No	No	No	No	No	Yes	No	No	No	2
Van den Heede, K., Sermeus, W., Diya, L.,	No	No	No	No	Yes	No	No	No	No	No	No	No	No	No	1

Clarke, S. P., Lesaffre, E., Vleugels, A., & Aiken, L. H. (2009). Nurse staffing and patient out-comes in Belgian acute hospitals: Cross-sectional analysis of administrative data.														
Wan TT, Shukla RK. Contextual and organizational correlates of the quality of hospital nursing care. QRB-Quality Review Bulletin. 1987 Feb 1;13(2):61-4.	No	No	No	No	No	No	No	No	No	No	No	No	Yes	No	1

Weiss ME, Yakusheva O, Bobay KL. Quality and cost analysis of nurse staffing, discharge preparation, and postdischarge utilization. Health services research. 2011 Oct;46(5):1473-94.	No	No	No	No	No	No	No	Yes	No	No	No	No	No	No	1
Whitman GR, Kim Y, Davidson LJ, Wolf GA, Wang SL. The impact of staffing on patient outcomes across specialty units. JONA: the Journal of Nursing Administration. 2002	No	No	No	No	No	No	No	No	Yes	No	Yes	No	No	No	2

Dec 1;32(12):633-9.															
Wiltse Nicely KL, Sloane DM, Aiken LH. Lower mortality for abdominal aortic aneurysm repair in high-volume hospitals is contingent upon nurse staffing. Health services research. 2013 Jun;48(3):972-91.	No	No	No	Yes	No	No	No	No	No	No	No	No	No	No	1
Yang KP. Relationships between nurse staffing and patient outcomes. Journal of Nursing	No	No	No	No	No	No	No	No	No	No	No	Yes	No	No	1

Research. 2003 Sep 1;11(3):149-58.														
Yasunaga H, Hashimoto H, Horiguchi H, Miyata H, Matsuda S. Variation in cancer surgical outcomes associated with physician and nurse staffing: a retrospective observational study using the Japanese Diagnosis Procedure Combination Database. BMC health services research. 2012 Dec;12(1):1-7.	No	No	No	Yes	No	No	No	No	No	No	No	No	No	No	1

Yu D, Ma Y, Sun Q, Lu G, Xu P. A nursing care classification system for assessing workload and determining optimal nurse staffing in a teaching hospital in China: A pre-post intervention study. International journal of nursing practice. 2015 Aug;21(4):339-49.	No	No	Yes	No	No	No	No	No	No	No	No	No	No	No	1
Zhu, X.-W., You, L.-M., Zheng, J., Liu, K. E., Fang, J.-B., Hou, S.-X., … Zhang, L.-F. (2012).	No	No	No	No	Yes	No	No	No	No	No	No	No	No	No	1

Nurse staffing levels make a difference on patient outcomes: A multisite study in Chinese hospitals. Journal of Nursing Scholarship, 44(3), 266–273.															
