# Supplementary material for: Identifying gaps in global evidence for nurse staffing and patient care outcomes research in low/middle-income countries: an umbrella review
Source: BMJ Open. 2022 Oct 12;12(10):e064050. doi: 10.1136/bmjopen-2022-064050 (PMC9562716; doi:10.1136/bmjopen-2022-064050)
Supplement: Supplementary data [file bmjopen-2022-064050supp005.pdf]

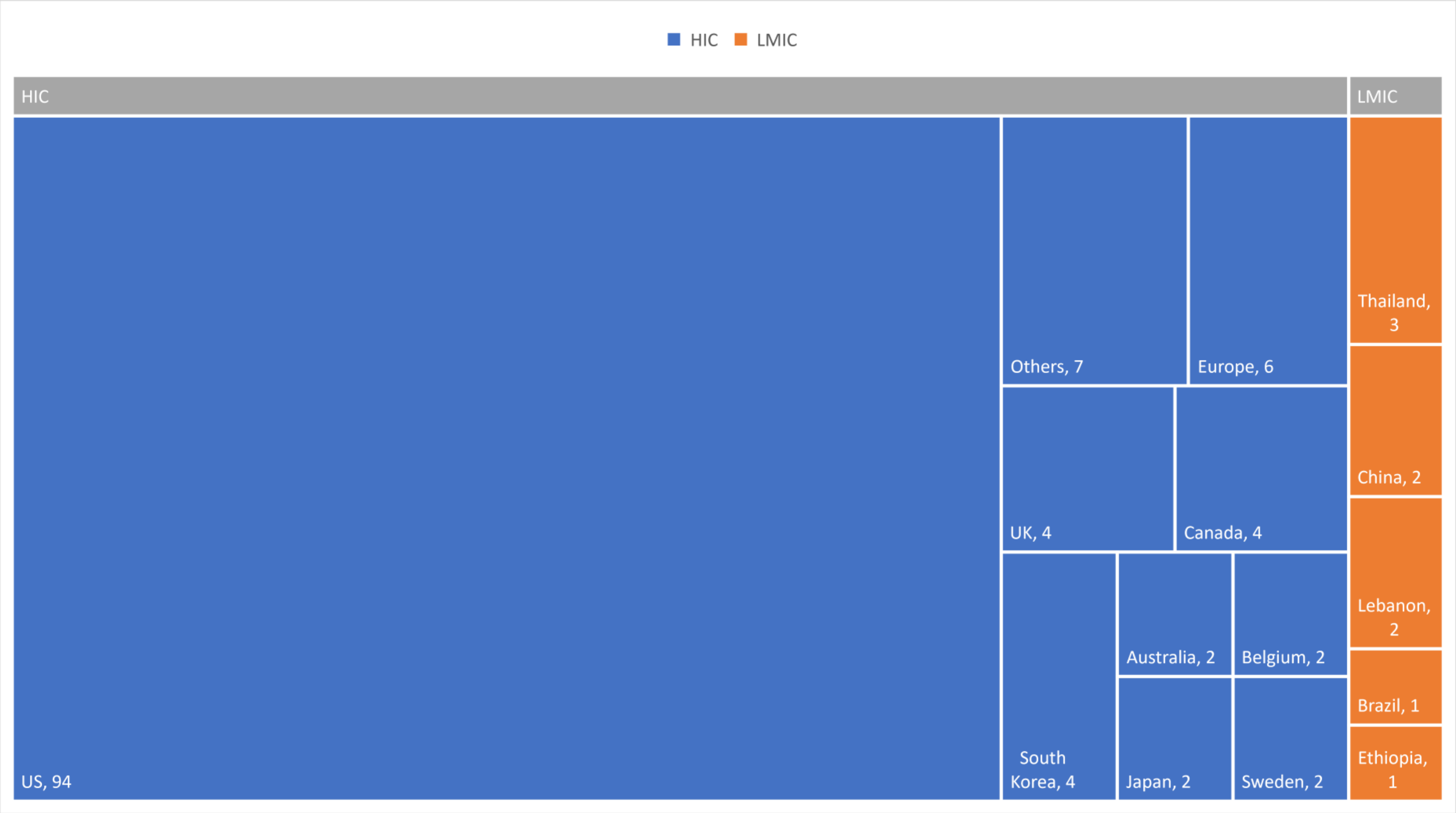

Online supplementary material 5 : Tree Map showing countries primary studies were conducted (n = 136).

Others group are HIC countries that had only 1 study – Kuwait, Taiwan, France, Finland + Netherlands (Dual country study), Multi-country, Italy, New Zealand.

*Studies conducted in Europe were conducted in multiple European countries. Two studies conducted in Lebanon and Sweden were also conducted in the US
