# Supplementary material for: Identifying gaps in global evidence for nurse staffing and patient care outcomes research in low/middle-income countries: an umbrella review
Source: BMJ Open. 2022 Oct 12;12(10):e064050. doi: 10.1136/bmjopen-2022-064050 (PMC9562716; doi:10.1136/bmjopen-2022-064050)
Supplement: Supplementary data [file bmjopen-2022-064050supp006.pdf]

Online supplemental material 6 – Summary of nursing metrics reported across papers

Nursing Metrics	Various definitions of individual metrics	Number of studies reporting a particular metric	Total number of studies reporting metrics
nursing hour per patient day	Total productive hours (care hours provided to patients) worked by registered nurses in a defined period/ Total number of patients admitted for care in the same defined period	33	45
	Total number of all nursing hours provided to patients in a defined period (includes any combination of registered nursing hours, licensed practice nursing hours and nurse assistant hours)/ Total number of patients admitted for care in the same defined period	11	
	Average number of nursing hours provided by registered nursing and licensed practice nursing in a defined period/average number of patients admitted for care in the same defined period	1	
Patient to nurse ratio per shift	Average reported number of patients by nurses on their last shift. Nurses' answers are averaged across a hospital to get a value.	32	32
Bed to nurse ratio/ Nurse per bed ratio	Total number of beds divided by the total number of full-time equivalent registered	2	10

	nurses (RNs) working in the general ward		
	Number of FTE nurses divided by the number of hospital beds	6	
	Number of inpatient nurses per 100 occupied beds	1	
	Infection control nurse to bed ratio	1	
Nurse to patient ratio/ patient to nurse ratio	The ratio of nurse productive hours worked to “patient census days/24	1	7
	The ratio of total nurse staff to the total number of patients in a defined period	2	
	The measure of the number of patients assigned to each nurse who has a direct patient care assignment	2	
	Undefined	2	
RN FTE per 1000 patient days	Number of Full-time registered nurses per 1000 patient-days	5	6
	The number of registered nurse full-time equivalents (FTEs) per 1000 in-patient days.	1	
RN per adjusted admission	Number of registered nurses/ adjusted admissions	1	2
	Number of full-time equivalent registered nurses per 100 adjusted admissions	1	
Others*		18	

*Others – Includes metrics that have frequencies of 1. This group includes - nursing hour per patient per shift, Inpatient clinical nursing worked hours per OCW), Mean difference between actual and expected nurse to patient ratio, RN hours per adjusted patient day, RN FTE, Total nursing hours, FTE RN to total hospital adjusted patient day, RN FTE per hospital, Daily average hours of care, All hours of direct care per patient day, RN per average daily census, The ratio of required to actual patient care hours, Required nursing personnel per shift, Number of licensed nurses, FTE RNs per adjusted average daily census, labour efficiency ratio, Daily average utilisation of RNs, Acuity-adjusted nursing hour per patient day
