# Supplementary material for: Identifying gaps in global evidence for nurse staffing and patient care outcomes research in low/middle-income countries: an umbrella review
Source: BMJ Open. 2022 Oct 12;12(10):e064050. doi: 10.1136/bmjopen-2022-064050 (PMC9562716; doi:10.1136/bmjopen-2022-064050)
Supplement: Author's manuscript [file bmjopen-2022-064050.draft_revisions.pdf]

BMJ Open is committed to open peer review. As part of this commitment we make the peer review history of every article we publish publicly available.

When an article is published we post the peer reviewers' comments and the authors' responses online. We also post the versions of the paper that were used during peer review. These are the versions that the peer review comments apply to.

The versions of the paper that follow are the versions that were submitted during the peer review process. They are not the versions of record or the final published versions. They should not be cited or distributed as the published version of this manuscript.

BMJ Open is an open access journal and the full, final, typeset and author-corrected version of record of the manuscript is available on our site with no access controls, subscription charges or pay-per-view fees (http://bmjopen.bmj.com).

If you have any questions on BMJ Open's open peer review process please email info.bmjopen@bmj.com

BMJ Open

IDENTIFYING GAPS IN GLOBAL EVIDENCE FOR NURSE STAFFING AND PATIENT CARE OUTCOMES RESEARCH IN LOW AND MIDDLE-INCOME COUNTRIES: AN UMBRELLA REVIEW

Journal:	*BMJ Open*
Manuscript ID	bmjopen-2022-064050
Article Type:	Original research
Date Submitted by the Author:	04-May-2022
Complete List of Authors:	Imam, Abdulazeez; KEMRI-Wellcome Trust Research Programme Nairobi, Health Services Unit; University of Oxford, Nuffield Department of Medicine<br>Obiesie, Sopuruchukwu; Independent researcher,<br>Aluvaala, Jalemba ; KEMRI-Wellcome Trust Research Programme Nairobi, Health Services Unit; University of Nairobi, Department of Paediatrics and Child Health<br>Maina, Jackson; KEMRI-Wellcome Trust Research Programme Nairobi, Health Services Unit<br>Gathara, David; KEMRI-Wellcome Trust Research Programme Nairobi, Wellcome Trust Research Program ; London School of Hygiene and Tropical Medicine Centre for Maternal Adolescent Reproductive and Child Health, MARCH Centre<br>English, Mike; KEMRI-Wellcome Trust Research Programme Nairobi, Health Services Unit; Nuffield Department of Clinical Medicine,
Keywords:	Health policy < HEALTH SERVICES ADMINISTRATION & MANAGEMENT, Health & safety < HEALTH SERVICES ADMINISTRATION & MANAGEMENT, Quality in health care < HEALTH SERVICES ADMINISTRATION & MANAGEMENT, PUBLIC HEALTH

SCHOLARONE™
Manuscripts

I, the Submitting Author has the right to grant and does grant on behalf of all authors of the Work (as defined in the below author licence), an exclusive licence and/or a non-exclusive licence for contributions from authors who are: i) UK Crown employees; ii) where BMJ has agreed a CC-BY licence shall apply, and/or iii) in accordance with the terms applicable for US Federal Government officers or employees acting as part of their official duties; on a worldwide, perpetual, irrevocable, royalty-free basis to BMJ Publishing Group Ltd ("BMJ") its licensees and where the relevant Journal is co-owned by BMJ to the co-owners of the Journal, to publish the Work in this journal and any other BMJ products and to exploit all rights, as set out in our licence.

The Submitting Author accepts and understands that any supply made under these terms is made by BMJ to the Submitting Author unless you are acting as an employee on behalf of your employer or a postgraduate student of an affiliated institution which is paying any applicable article publishing charge ("APC") for Open Access articles. Where the Submitting Author wishes to make the Work available on an Open Access basis (and intends to pay the relevant APC), the terms of reuse of such Open Access shall be governed by a Creative Commons licence – details of these licences and which Creative Commons licence will apply to this Work are set out in our licence referred to above.

Other than as permitted in any relevant BMJ Author's Self Archiving Policies, I confirm this Work has not been accepted for publication elsewhere, is not being considered for publication elsewhere and does not duplicate material already published. I confirm all authors consent to publication of this Work and authorise the granting of this licence.

For peer review only

IDENTIFYING GAPS IN GLOBAL EVIDENCE FOR NURSE STAFFING AND PATIENT CARE OUTCOMES RESEARCH IN LOW AND MIDDLE-INCOME COUNTRIES: AN UMBRELLA REVIEW

Abdulazeez Imam[1,2] Sopuruchukwu Obiesie[3] Jalemba Aluvaala[1,4] Michuki Maina[1] David Gathara[1,5] Mike English [1,2]

[1] KEMRI-Wellcome Trust Research Programme, Nairobi, Kenya

[2]Oxford Centre for Global Health Research, Nuffield Department of Medicine, University of Oxford, S Parks Rd, Oxford, OX1 3SY, UK

[3]Independent researcher, Delta State, Nigeria

[4]Department of Paediatrics, University of Nairobi, Nairobi, Kenya

[5]MARCH Centre, London School of Hygiene and Tropical Medicine, London, UK

Corresponding author:

Abdulazeez Imam

KEMRI-Wellcome Trust Research Programme, Nairobi, Kenya

Email: abdulimam2001@yahoo.com

Word Count - 3706

Keywords – Quality of care, Developing countries, Nursing staff, Patient safety, Health systems

For peer review only - http://bmjopen.bmj.com/site/about/guidelines.xhtml

Abstract

Objective To identify the global literature on nurse staffing and patient care outcomes from existing systematic reviews and map out the evidence gaps for Low-middle Income Countries (LMICs).

Design Umbrella review

Study selection - published systematic reviews that reported on the association between nurse staffing and patient care outcomes

Data sources - Cochrane Register of Systematic Reviews, the JBI Database of Systematic Reviews and Implementation Reports, Medline, Embase and Cumulative Index to Nursing and Allied Health Literature (CINAHL) from inception till July 2021.

Exposure Nurse staffing levels

Outcome Quality of patient care

Results From 843 papers, we identified 33 systematic reviews for full-text screening and included fourteen in our synthesis. There were overlaps in primary studies summarised across reviews, but overall, the reviews summarised 136 unique primary articles. Ten of 14 reviews had no LMIC publications and 9 (6.6%) of 136 unique primary articles were conducted in LMICs. Only 8 of 23 patient care outcomes were reported from LMICs. Less research was conducted in contexts with staffing levels that are typical of many LMIC contexts.

Conclusions Our umbrella review identified very limited data for nurse staffing and patient care outcomes in LMICs. More importantly, we identified data from HICs might not be good proxies for LMICs as staffing levels where this research was conducted had comparatively better staffing levels than the few LMIC studies. This highlights a critical need for the conduct of nurse staffing research in LMIC contexts.

PROSPERO registration number: CRD42021286908

For peer review only - http://bmjopen.bmj.com/site/about/guidelines.xhtml

KEY QUESTIONS

What is already known?

- Poor nurse staffing has a detrimental effect on the quality-of-care patients receive and most data on nurse staffing and patient care outcome has come from High-Income countries (HICs), where they have played a role in shaping nurse staffing policies.

What are the new findings?

- Most systematic reviews have no data from LMIC settings and the overall number of primary studies from LMICs is very limited. These LMIC studies have also investigated a narrower range of patient care outcomes compared to those from HICs. Overall, less research is conducted in areas with nurse staffing ratios that are similar to those of LMICs.
- There were 29 different nurse staffing metrics used across different studies which made effective pooling of data across studies difficult.

How this study might affect research, practice and/or policy?

- The available data on nurse staffing and patient care outcomes from HICs might not be reliable proxies for LMICs as the evidence comes from areas with better nurse staffing and there is a critical need to research nurse staffing and quality of patient care in LMICs to influence local staffing policies.
- There is a need to standardise data collection and reporting in this research area to enable comparative learning across multiple contexts.

For peer review only - http://bmjopen.bmj.com/site/about/guidelines.xhtml

BACKGROUND

Donabedian in his seminal work on quality of care describes a structure-process-outcome framework.(1) In his framework, structures present within health services influence the processes of care which in turn are likely to affect care outcomes.(1) A key element of health service structure is staffing and nurses who represent a large percentage of hospital staffing are thus likely to have significant effects on hospital patient outcomes.(2) Nurses play multiple, crucial roles planning, delivering and coordinating care and represent a key part of the hospitals' surveillance system in detecting adverse patient events.(3)

An adequate nursing workforce is central to the delivery of quality patient care. Research that comes from mainly High-Income Countries (HICs) demonstrates that poor ward nurse staffing is associated with negative patient care outcomes, for example, increased risk for patient mortality, prolonged hospital stay and an increased risk for hospital-acquired complications.(4-14). This has led to a shift towards better staff-to-patient ratios or minimum nurse staffing standards to guide ward nurse staffing levels in many HICs. In 1999, California state adopted a bill mandating the state Department of Health Services to enforce minimum staffing ratios across hospitals within the state and this has been the focus of multiple research studies investigating the effects on the quality of patient care.(15-18) Shortly after this, in 2002, the state of Western Australia used the Nursing Hour Per Patient Day method which classifies wards into seven categories based on patient care acuity and complexity to determine relative nurse staffing needs.(19, 20) In other countries such as the United Kingdom, the Royal College of Nursing also provides minimum staffing guidance for wards, for example, paediatric wards are required to maintain a minimum ratio of 1 registered nurse to 4 children with better ratios applied to high dependency and intensive care settings.(21)

For peer review only - http://bmjopen.bmj.com/site/about/guidelines.xhtml

The evolution and the evidence for nurse staffing in LMICs are less clear. In more resource challenge LMIC settings, nurse staffing ratios might be as low as 1 nurse caring for over 25 patients on a shift.(22, 23) For such settings, the World Health Organization has promoted the Workload Indicator Staffing Needs (WISN) planning tool which relies on data from health information systems.(24) The central focus of the WISN tool is the workload of the average health worker and the time to carry out their activities as defined by experts.(24) It aims to provide context-specific estimates of workforce requirements that are based on service types and complexity within local health facilities. However, the WISN approach often suggests workforce expansion that seems unachievable for countries due to the major increases in rates of production and financing required, highlighting the practical realities of human resource costs which can represent up to two-thirds of health service budgets.(25,26)

Over the last two decades, several systematic reviews have synthesised the evidence for nurse staffing and patient care outcomes.(27-31) It has not been formally investigated to what extent these reviews provide evidence for LMIC settings that have distinctively different practice environments and organizational contexts to HICs. Umbrella reviews (overviews of systematic reviews) can integrate research in a particular research area to provide broader knowledge on a specific topic.(32) Using this method, we appraised the existing evidence related to nurse staffing for LMICs put forward within reviews and compared this with the global literature to identify the evidence gaps for LMICs for nurse staffing and patient care outcome research. This also builds on a recent LMIC focused review in the subject area which demonstrated limited and poor quality evidence.(30) It is highly likely that the current extreme staffing ratios in LMICs contribute to poor patient outcomes and overall poor-quality care. To address this and conduct appropriate local research, however, we should first determine where gaps exist.

For peer review only - http://bmjopen.bmj.com/site/about/guidelines.xhtml

Aim and objectives

The primary objective of this umbrella review is to identify the evidence gaps for LMICs in the existing literature that investigates the association between hospital nurse staffing and patient care outcomes. We will address the following research questions:

a. Where are studies within published reviews conducted and what proportion of these are carried out in LMICs?
b. What patient care outcomes are reported across reviews and how do reported outcomes differ between HICs and LMIC studies?
c. What is the range of nurse staffing levels that have been researched across acute care settings and how do these differ between LMICs and HICs?

METHODS

Research design

We conducted an umbrella review using guidance from the Joanna Briggs Institute for the preparation and conduct of the review.(33) Our review protocol was registered with the International Prospective Register of Systematic Reviews (PROSPERO), Registration number - CRD42021286908 and has been published.(34)

Data sources and search strategy

To identify published systematic reviews that reported on the association between nurse staffing and patient care outcomes, we conducted a systematic search of 5 electronic databases from their inception

For peer review only - http://bmjopen.bmj.com/site/about/guidelines.xhtml

up until July 2021. These were the Cochrane Register of Systematic Reviews, the JBI Database of Systematic Reviews and Implementation Reports, Medline, Embase and Cumulative Index to Nursing and Allied Health Literature (CINAHL). Our search strategy for this is detailed in Supplemental file 1.

We included some of the following keywords and their synonyms: nurse, nursing, outcome, quality, missed nursing, mortality and identified some key MESH terms (Supplemental file 1). We combined these using Boolean operators "AND" and "OR" where appropriate. We also searched references of our included systematic reviews to identify other reviews.

Selection of systematic reviews

We included quantitative systematic reviews that: a.) summarised literature on nurse staffing and patient care outcomes in regular ward settings; b) were conducted in English language (due to limitations in translation among the research team).

Reviews were excluded if they: a) were conducted in a non-hospital setting; b) only summarised studies conducted in non-regular ward settings, for example, intensive care units; c) reported exclusively on non-patient care outcomes, such as nurse outcomes (for example, burnout and nurse satisfaction); d) reported on other nurse staffing characteristics, for example, skill mix; e) were qualitative and mixed-method reviews.

For reviews that reported mixed outcomes or care settings, for example, reviews summarising literature on both nurse and patient outcomes or those summarising research in both standard ward settings and intensive care units, we include these but only summarised and reported on specific data which focused on our inclusion criteria.

For peer review only - http://bmjopen.bmj.com/site/about/guidelines.xhtml

Screening

References were managed using the Zotero reference software.(35) We performed initial deduplication in Zotero and a 2nd round of deduplication in Microsoft excel. Following which we exported our final set of articles to Rayyan, a web-based application for screening,(36) where two reviewers, AI and SO independently screened the titles and abstracts of all identified systematic reviews for eligibility. They then read full texts of selected articles and agreed on a final set of papers.

Quality assessment

We evaluated each systematic review for risk of bias using the AMSTAR-2 criteria.(37) This tool comprises 16 appraisal questions rated 'Yes' when a criterion is fulfilled; 'No' when it is not fulfilled and 'Partial Yes' when it is somewhat fulfilled (This is based on explicitly stated criteria). The AMSTAR criteria cover important questions such as whether the PICO elements were clearly described by the review, whether the conduct of study selection, data abstraction and risk of bias were conducted by at least two reviewers and the use of a comprehensive search strategy.(37) The quality appraisal was performed independently by AI and SO and disagreements were managed through discussions.

Data extraction

Both reviewers (AI and SO) independently extracted data from the final set of systematic reviews using a pre-designed data abstraction tool. This data included the review publication year, objectives, reported patient care outcomes, number and origin of review studies that reported on nurse staffing and patient outcomes in regular ward care settings.

Summary data on the nurse staffing metrics were also retrieved from the individual paper summaries within systematic reviews. Where this information was unavailable as a summary within the reviews, we

For peer review only - http://bmjopen.bmj.com/site/about/guidelines.xhtml

abstracted them directly from the individually referenced articles. AI abstracted this data and SO cross-checked a random 40% of this.

Data synthesis

Our umbrella review findings are presented in narrative form using tables and figures. We tallied the countries of origin of the individually referenced papers within each review and classified these into LMIC and non-LMIC using the World Bank country and lending group classification system as of 23rd December 2021.(38) We also summarised and tallied all reported patient care outcomes from the included systematic reviews and made comparisons between made across LMICs and HICs.

We identified and collated summary statistics (range, mean or median) for nurse staffing metrics reported in individually referenced papers using tables and bar charts where these were reported. Because of the heterogeneity of retrieved metrics, a meta-analysis was not possible.

Patient and public involvement

There was no patient or public involvement in the design and conduct of this study.

Ethics statement

As we used secondary data sources, we did not require ethical approval for the conduct of this review.

For peer review only - http://bmjopen.bmj.com/site/about/guidelines.xhtml

RESULTS

Search results

Our initial search of databases identified 1365 articles. We identified and excluded 522 duplicate articles and screened the title and abstract of 843 remaining articles. Of the 33 systematic reviews that met our criteria for full text reviews, we included 14.(19, 27-30, 39- 48). The PRISMA flow chart in Figure 1 provides a summary of our screening process while Supplemental file 2 shows the list of excluded systematic reviews and the reasons for their exclusion.

Description of included reviews

We included 14 systematic reviews in our final analysis. The reviews covered periods from as early as 1980 up until 2020 (Table 1). The reviews were conducted on studies from predominantly medical, surgical or adult wards with only one review conducted on those from Paediatric ward settings (44). The reviews included between 5 and 54 primary studies. Of the individual studies within reviews, between 12.5% and 100.0% met our inclusion (Table 1). This was because some reviews in addition to summarising studies that investigated the effect of nurse staffing on patient care outcomes also included those that reported other outcomes, for example, nurse outcomes.(30) Other reviews also included studies conducted in non-regular ward settings such as intensive care unit settings,(41,43) or included a mixture of studies investigating nurse staffing and other interventions such as nurse skill mix.(28) In Supplemental file 3, for each review, we have provided a list of primary studies we did not report on and the reasons for this. There was some overlap in the primary articles summarised by the reviews, some papers were referenced in as many as 6 reviews (Supplemental file 4). In total 136 unique primary studies were identified across all systematic reviews (Supplemental file 4).

For peer review only - http://bmjopen.bmj.com/site/about/guidelines.xhtml

For peer review only

For peer review only - http://bmjopen.bmj.com/site/about/guidelines.xhtml

Table 1: An overview of the included systematic reviews showing the review objective and geographical locations where the reviews primary studies were conducted

First author (Year)	Period of review	Research setting	Number of primary studies included	Number of primary studies describing nurse staffing levels and patient outcomes in non-ICU settings[&]	Geographic locations of studies and frequency	Number of low-middle income countries included (%)	Number of high-income countries included (%)
Griffiths (2018)	2006 - 2016	Adult hospital in-patient wards	18	17	Kuwait - 1, Europe -3, UK-1, Sweden -1, US* - 7, Lebanon* -1, Switzerland-1,Italy- 1, South Korea- 2	1 (5.6)	17 (95.4)
Assaye (2020)	UR - 2019	Acute care hospital settings	27	6	Brazil – 1, Thailand – 2, China – 1, Ethiopia – 1, Lebanon - 1	6 (100.0)	0 (0.0)
Thungjaroenkul (2007)	1990 - 2006	ICUs, medical and surgical units	17	5	US - 5	0 (0.0)	5 (100.0)
Twigg (2020)	2000 - 2020	General medical, surgical, step-down units ,emergency departments, intensive care, and nursing homes	22	7	US – 6, Australia - 1	0 (0.0)	7 (100.0)
Shin (2019)	2000 - 2018	Medical and surgical units	19	17	US – 10, South Korea – 2, Finland* - 1, Netherland* - 1, Lebanon – 1, Belgium – 1, China -1, Japan - 1	2(11.1)	16 (88.9)

For peer review only - http://bmjopen.bmj.com/site/about/guidelines.xhtml

Lang (2004)	1980 - 2003	Acute care hospitals	43	24	US - 24	0 (0.0)	24 (100.0)
Lankashear (2005)	1990 - 2004	Acute care hospitals	22	19	US – 18, Canada -1	0 (0.0)	19 (100.0)
Kane (2007)	1990 - 2006	Acute care hospitals	28	17	US - 17	0 (0.0)	17 (100.0)
Bourgon (2019)	1996 - 2018	Surgical units	44	28	US – 17, Europe - 2, South Korea - 1, UK – 2, New Zealand – 1, Australia – 2, Belgium – 2, Japan - 1	0 (0.0)	28 (100.0)
Wilson (2011)	1993-2010	Paediatric wards	8	5	US – 2, France – 1, Canada -1, UK - 1	0 (0.0)	5 (100.0)
Engineer (2016)	2000-2012	Acute care hospitals	16	2	US - 2	0 (0.0)	2 (100.0)
Staplers (2015)	2004 - 2012	Acute care hospitals	29	16	US* - 12, Belgium* - 1, New Zealand – 1, Australia – 1, UK – 1, Sweden - 1	0 (0.0)	17 (100.0)
Mitchell (2018)	2000 - 2015	Hospital wards	54	19	US – 13, Thailand – 1, Australia -1, Taiwan – 1, Canada – 2, Multi-country - 1	1 (5.3)	18 (95.7)
Hill (2017)	1994 - 2017	Acute care hospitals	5	5	US – 3, UK - 2	0 (0.0)	5 (100.0)

*- one study conducted across both countries,

&- These are the number of studies that met the original review criteria .i.e., conducted in a regular ward setting on the role of nurse staffing and patient care outcomes. UR – Unreported

For peer review only - http://bmjopen.bmj.com/site/about/guidelines.xhtml

Quality assessment of included reviews

We used the AMSTAR-2 quality assessment tool to appraise individual systematic reviews.(37) As shown in Figure 2, Question 2 (Q2), 11 out of 14 of the reviews did not refer to a study protocol or provide explicit statements of their review methods being established before the review conduct. Most reviews (12 out of 14) did not provide a list of excluded studies with justifications on reasons for excluding these studies (Figure 2, Q7). Discussions on how the risk of bias assessments of individual studies might have impacted the review results were also limited (Figure 2, Q13)

Origin of primary studies and proportion of studies in systematic reviews conducted in LMIC settings.

Of the 136 unique studies, 94 (69.1%) were carried out in the USA. Only 5 LMIC countries, Thailand, Brazil, Lebanon, China, and Ethiopia accounted for 9 (6.6%) of 136 primary studies (Supplemental file 5). Studies from HICs came from more diverse locations. These studies were conducted in 16 other settings in addition to the USA (Supplemental file 5). Ten out of 14 of our included systematic reviews reported no LMIC publications on our subject of interest (nurse staffing and patient care outcomes) (Table 1). One review restricted to LMIC settings identified only 6 articles (Table 1).

Reported patient care outcomes and differences across research settings (LMICs and HICs)

In total, the included reviews reported 23 patient care outcomes. Each review reported between 1 and 15 outcomes and the most frequently reported (8 times each) were patient mortality, pressure ulcers and the incidence of hospital-acquired infections (Figure 3).

For peer review only

For peer review only - http://bmjopen.bmj.com/site/about/guidelines.xhtml

Only 8 of the 23 patient care outcomes were reported in an LMIC setting. These were missed nursing care, mortality, pressure ulcers, length of stay, treatment errors, hospital-acquired infections, falls and hospital-acquired injuries (Figure 3). Other outcomes such as postoperative complications, cardiac arrests, deep venous thrombosis, failure to rescue, unplanned extubations and incidence of restraint use were not reported in LMICs (Figure 3).

Measures of nurse staffing and reported staffing range across LMICs and HICs

There were 29 different reported nurse staffing metrics across the individual studies within our included systematic reviews. The most frequently reported metrics were the Nursing Hour Per Patient Day (NHPPD) and the patient to nurse ratio per shift which were reported by 48 and 34 primary studies respectively (Supplemental file 6). There was marked heterogeneity in how both metrics were reported by papers. Some reported absolute values, mean, median, ranges, percentiles, or categories. For those reporting either a range, mean or median, we have summarised their values in Figures 4 and 5.

Figure 4 depicts patients per nurse for 9 studies (8 HICs and 1 LMIC study). Studies from HICs reported best staffing ratios (smallest patient per nurse ratio) ranging between 4 patients per nurse and 7, (49-56) and this was in contrast to the sole LMIC study from Brazil which reported a best ratio of 9 patients per nurse.(57) Similarly, the worst rations documented in studies ranged between 9 and 18 patients per nurse within HICs in contrast to 27 patients per nurse in the LMIC study (Figure 4).(57)

All studies which reported the mean patient to nurse ratio per shift were in HICs and 16 of 18 reported mean values below 10 (Figure 5). (49, 50, 55, 56, 58-69) For studies reporting total mean Nursing Hour Per Patient Day (NHPPD), the 3 reported LMIC studies (from Thailand and Lebanon) had the lowest values,(70-72) and these were almost 2.5-fold below studies conducted in some HICs (Figure 5).(73-78)

For peer review only - http://bmjopen.bmj.com/site/about/guidelines.xhtml

DISCUSSION

This umbrella review of published systematic reviews on nurse staffing and patient care outcomes examined the evidence gaps for LMICs by appraising the global evidence. We found a dearth of evidence from LMICs with the link between nurse staffing and quality exploring a narrower range of patient care outcomes.

Of the 14 included systematic reviews in this study, 10 (71.4%) had no data from an LMIC setting. Across all 14 reviews only 9 of the 136 unique primary studies included were from LMICs and these data came from five countries (Thailand, Lebanon, China, Brazil and Ethiopia),(57, 70- 72, 79-83) with one from Africa (Ethiopia).(83) A recent LMIC-focused systematic review found only 6 nurse staffing and patient care outcome papers that were graded as providing low-quality evidence.(30) With 90% of the global deficit of nurses concentrated in LMIC settings,(2) research is needed in such settings to examine the impact these shortages have on the quality of hospital care. Ideally these studies would examine the impact of improving staffing numbers on the overall quality of patient care that is provided and patients' health outcomes. This data is critical to highlight the effects of the current staffing shortages and to guide policymakers and managers in developing appropriate staffing policies.

The data that currently exists illustrates the negative effects of low nurse staffing on quality of care but is almost exclusively from High-Income Countries (HIC).(52, 59, 60) Traditionally, these areas already have better staffing ratios than LMICs and yet relatively small absolute or relative improvements in staffing can demonstrate positive effects. We noted nurse staffing metrics reported in LMICs were more extreme in comparison with HICs. For example, patient to nurse ratio, a metric that measures the number of patients per nurse on a shift was reported to be as high as 27 patients in a Brazil study,(57) and this starkly contrasts with figures of between 4 and 9 patients per nurse from US studies.(49, 55) Also, nursing hour per patient

For peer review only - http://bmjopen.bmj.com/site/about/guidelines.xhtml

day (NHPPD) which measures the total number of nursing hours provided in a defined period adjusted for the number of admissions in the same period ranged between 4.25 and 5.33 in LMICs,(70-72) in contrast with some values from the US were 2.5 fold higher.(73) Because the nurse-to-patient ratios are comparatively poorer in LMICs, increasing nurse staffing might potentially lead to larger impacts on quality of patient care and overall patient outcomes. Local research in these contexts is however needed to confirm this.

Staffing levels and strength in the literature were measured using various proxy metrics. While NHPPD and Patient per nurse ratios were the most frequently employed staffing metrics across studies, we observed a wide heterogeneity in reported staffing metrics across studies. In total, there were 29 reported unique nurse staffing metrics across the literature and report of their summary statistics were inconsistent across studies. This limited our ability to collate findings across multiple studies to examine the range of nurse staffing where this research has been conducted. Previous reviews have also documented similar heterogeneity in nurse staffing research and literature.(28) Progress within this area of research would benefit from a move towards standardisation and reporting of core metrics across different studies and research settings to promote comparative learning across contexts.

As a proxy for the quality of patient care, a range of outcomes was reported across systematic reviews. Together all our included reviews reported 23 different outcomes with the most popular being patient mortality, pressure ulcers and the incidence of hospital-acquired infections. We however noted a narrower range of patient care outcomes were also reported in LMIC settings in comparison to HICs, only 8 outcomes were reported in studies from LMIC settings. The reported patient care outcomes in LMICs were missed nursing care, mortality, the incidence of pressure ulcers, patient length of stay, treatment errors, hospital-acquired infections, and falls. Other outcomes such as incidence of deep venous thrombosis or postoperative complications were not reported. One reason might be limited diagnostic capacity in LMICs; many patient complications may go unrecognised and so the burden of these is under-

For peer review only - http://bmjopen.bmj.com/site/about/guidelines.xhtml

recognised with no data collected. This finding probably also reflects limited health administrative and secondary data sources in LMICs which preclude the reporting of these data. Indeed, studies within these contexts have been based on reviewing paper-based case records which are usually unstructured and have not been curated for research purposes.(57, 83) There are now some examples of networks collecting secondary data in LMICs. For example, in Kenya since 2013, the Clinical Information Network collects secondary data using structured admission records from a network of 23 hospitals and this data has been used repeatedly in quality improvement research,(84) including tracking patient quality indicators such as the frequency at which patient vital signs were conducted.(85) Such databases might play a role in reducing the evidence gap and are likely to be crucial in guiding research to policy. Interestingly, one outcome which does not need advanced diagnostics or secondary data collection is patient satisfaction, but there was no report linking nurse staffing to patient satisfaction from an LMIC.

The majority of the reported outcomes within reviews (22 out of 23) were outcome-based measures of quality of care, except for missed nursing care, a care outcome that describes partially or completely omitted or delayed nursing care (86) which we identified as the sole process-based measure of the quality of care. Care processes occur more frequently than outcomes and as such are useful as studies employing them might be of more manageable size. They might also represent important measures for intervention research aimed at either demonstrating the influence of enhancements in nurse staffing or determining the likely influence of an intervention on the quality of patient care due to their likely proximal position in the causal pathway of quality of patient care.

Strengths and limitation

To the best of our knowledge, this is the first review to provide a comprehensive review of the evidence for LMICs in published systematic reviews on the association between nurse staffing and patient care

For peer review only - http://bmjopen.bmj.com/site/about/guidelines.xhtml

outcomes. We highlight critical gaps in these care settings that need to be the focus of future research in this area.

We recognise that the quality of the information provided by this umbrella review is highly dependent on the underlying quality of the systematic reviews. Some of our included systematic reviews scored low on our risk of bias scores, but because our focus was a broad description of the global evidence to identify gaps for LMICs we still include these reviews in our synthesis. Due to limitations in translation on the research team, we only considered systematic reviews published in English. We also did not consider qualitative reviews as our umbrella review focused on reviews that investigated an association between nurse staffing and patient care outcomes.

Conclusions

Our umbrella review demonstrates that the evidence examining links between nurse staffing and patient care outcomes in LMICs is very limited, both in terms of the number of studies and the outcomes that have been investigated. While there is research conducted in HICs, these might be poor proxies for LMIC settings as staffing ratios in these settings are quite different. Research in LMIC settings is clearly needed to examine the effect of nursing shortages and interventions addressing these on the overall quality of patient care and inform local staffing policy in these regions. To enhance learning across contexts there needs to be greater uniformity and standardization of metrics used to measure nurse staffing.

For peer review only - http://bmjopen.bmj.com/site/about/guidelines.xhtml

Figure 1 – PRISMA diagram for search

Figure 2 – Risk of Bias assessment using the AMSTAR-2 checklist. (Key: Red – No, Green – Yes, lighter green – Partially Yes, Yellow – Not applicable)

Figure 3: Stacked bar chart showing the range of patient care outcomes reported across reviews and the number of reviews which report this in a LMIC study (Blue- number of reviews reporting the outcome from a LMIC study, Orange – number of reviews reporting the outcome from only non-LMIC studies

Figure 4 –Bar chart showing the patient to nurse ratio per shift range described in individual studies that reported this metric and described a range (countries of origin and first author of the individual studies are presented, and the Y-axis represents the number of patients per nurse).

Figure 5 – Horizontal bar charts showing the mean total nursing hour per patient day (top) and the mean patient to nurse ratios (bottom) across primary studies reporting these metrics. (First author and countries in which individual studies were conducted are depicted on the Y-axis).

For peer review only

For peer review only - http://bmjopen.bmj.com/site/about/guidelines.xhtml

Authors contribution statement

AI conceptualised the idea for the manuscript with inputs from DG, MM, JA, and ME. AI and SO conducted the methods for the paper. AI drafted the manuscript with significant contribution from all authors and under the supervision of DG, MM, JA, and ME. All the authors reviewed all versions of the manuscript and agreed on a final version for submission.

Competing interests

All authors declare no competing interests

Funding

AI is supported for a PhD studentship at the University of Oxford by the National Institute for Health Research [NIHR130812]. ME and JA receive salary support from a Wellcome Trust Senior Research Fellowship (# 207522) awarded to ME. MM & DG receive salary support from the National Institute for Health Research (NIHR)(NIHR130812): Learning to Harness Innovation in Global Health for Quality Care (HIGH-Q) grant using UK aid from the UK government to support global health research. A Wellcome Trust core award to the KEMRI-Wellcome Trust Research Programme (#092654) enables this research.

The funders had no role in the study design, data collection and analysis, or preparation of the manuscript. the views expressed in this publication are those of the authors and not necessarily those of the Wellcome Trust, NIHR, or the UK government

For peer review only - http://bmjopen.bmj.com/site/about/guidelines.xhtml

Data availability statement

Our search strategy is available in the supplemental files and a list of both included and excluded studies have been included in our tables and supplemental files

Ethical approval

This review used secondary data from previously published systematic reviews and so ethical approval or patient consent was not required.

For peer review only

References

1. Donabedian A. Evaluating the quality of medical care. Milbank Mem Fund Q. 1966;44(3):166–206.

2. Organization WH. State of the world's nursing 2020: investing in education, jobs and leadership. 2020;

3. Clarke SP, Aiken LH. Failure to Rescue: Needless deaths are prime examples of the need for more nurses at the bedside. AJN Am J Nurs. 2003;103(1):42–7.

4. Aiken LH, Sloane D, Griffiths P, Rafferty AM, Bruyneel L, McHugh M, et al. Nursing skill mix in European hospitals: cross-sectional study of the association with mortality, patient ratings, and quality of care. BMJ Qual Saf. 2017;26(7):559–68.

5. Griffiths P, Maruotti A, Saucedo AR, Redfern OC, Ball JE, Briggs J, et al. Nurse staffing, nursing assistants and hospital mortality: retrospective longitudinal cohort study. BMJ Qual Saf. 2019;28(8):609–17.

6. Aiken LH, Clarke SP, Sloane DM, Sochalski J, Silber JH. Hospital nurse staffing and patient mortality, nurse burnout, and job dissatisfaction. Jama. 2002;288(16):1987–93.

7. Rafferty AM, Clarke SP, Coles J, Ball J, James P, McKee M, et al. Outcomes of variation in hospital nurse staffing in English hospitals: cross-sectional analysis of survey data and discharge records. Int J Nurs Stud. 2007;44(2):175–82.

8. Cho E, Sloane DM, Kim E-Y, Kim S, Choi M, Yoo IY, et al. Effects of nurse staffing, work environments, and education on patient mortality: an observational study. Int J Nurs Stud. 2015;52(2):535–42.

9. Tubbs-Cooley HL, Cimiotti JP, Silber JH, Sloane DM, Aiken LH. An observational study of nurse staffing ratios and hospital readmission among children admitted for common conditions. BMJ Qual Saf. 2013;22(9):735–42.

10. Griffiths P, Recio-Saucedo A, Dall'Ora C, Briggs J, Maruotti A, Meredith P, et al. The association between nurse staffing and omissions in nursing care: a systematic review. J Adv Nurs. 2018;74(7):1474–87.

11. Recio-Saucedo A, Dall'Ora C, Maruotti A, Ball J, Briggs J, Meredith P, et al. What impact does nursing care left undone have on patient outcomes? Review of the literature. J Clin Nurs. 2018;27(11–12):2248–59.

12. Driscoll A, Grant MJ, Carroll D, Dalton S, Deaton C, Jones I, et al. The effect of nurse-to-patient ratios on nurse-sensitive patient outcomes in acute specialist units: a systematic review and meta-analysis. Eur J Cardiovasc Nurs. 2018;17(1):6–22.

13. Shekelle PG. Nurse–patient ratios as a patient safety strategy: a systematic review. Ann Intern Med. 2013;158(5_Part_2):404–9.

For peer review only - http://bmjopen.bmj.com/site/about/guidelines.xhtml

14. Kane RL, Shamliyan TA, Mueller C, Duval S, Wilt TJ. The association of registered nurse staffing levels and patient outcomes: systematic review and meta-analysis. Med Care. 2007;1195–204.

15. Mark BA, Harless DW, Spetz J, Reiter KL, Pink GH. California's minimum nurse staffing legislation: results from a natural experiment. Health Serv Res. 2013;48(2pt1):435–54.

16. Cook A, Gaynor M, Stephens Jr M, Taylor L. The effect of a hospital nurse staffing mandate on patient health outcomes: Evidence from California's minimum staffing regulation. J Health Econ. 2012;31(2):340–8.

17. Burnes Bolton L, Aydin CE, Donaldson N, Storer Brown D, Sandhu M, Fridman M, et al. Mandated nurse staffing ratios in California: a comparison of staffing and nursing-sensitive outcomes pre-and postregulation. Policy Polit Nurs Pract. 2007;8(4):238–50.

18. Hickey PA, Gauvreau K, Jenkins K, Fawcett J, Hayman L. Statewide and national impact of California's staffing law on pediatric cardiac surgery outcomes. JONA J Nurs Adm. 2011;41(5):218–25.

19. Twigg DE, Whitehead L, Doleman G, El-Zaemey S. The impact of nurse staffing methodologies on nurse and patient outcomes: A systematic review. J Adv Nurs. 2021;(7609811, h3l).

20. Twigg D, Duffield C. A review of workload measures: a context for a new staffing methodology in Western Australia. Int J Nurs Stud. 2009;46(1):132–40.

21. NHS England » Safe, sustainable and productive staffing for neonatal care and children and young people's services [Internet]. [cited 2021 Jun 1]. Available from: https://www.england.nhs.uk/ourwork/safe-staffing/safe-sustainable-and-productive-staffing-for-neonatal-care-and-children-and-young-peoples-services/

22. Assaye AM, Wiechula R, Schultz TJ, Feo R. Nurse staffing models in medical-surgical units of acute care settings: A cross-sectional study. Int J Nurs Pract. 2020;26(1):e12812.

23. Gathara D, Serem G, Murphy GA, Obengo A, Tallam E, Jackson D, et al. Missed nursing care in newborn units: a cross-sectional direct observational study. BMJ Qual Saf. 2020;29(1):19–30.

24. WHO | Workload indicators of staffing need (WISN): selected country implementation experiences [Internet]. WHO. World Health Organization; [cited 2021 Jun 1]. Available from: http://www.who.int/hrh/resources/workload_indicators_health-obs15/en/

25. Gerdtz MF, Nelson S. 5-20: a model of minimum nurse-to-patient ratios in Victoria, Australia. J Nurs Manag Wiley-Blackwell. 2007 Jan;15(1):64–71.

26. WHO | The world health report 2000 - Health systems: improving performance [Internet]. WHO. World Health Organization; [cited 2021 May 31]. Available from: https://www.who.int/whr/2000/en/

27. Griffiths P, Recio-Saucedo A, Dall'Ora C, Briggs J, Maruotti A, Meredith P, et al. The association between nurse staffing and omissions in nursing care: A systematic review. J Adv Nurs John Wiley Sons Inc. 2018 Jul;74(7):1474–87.

For peer review only - http://bmjopen.bmj.com/site/about/guidelines.xhtml

28. Kane RL, Shamliyan TA, Mueller C, Duval S, Wilt TJ. The association of registered nurse staffing levels and patient outcomes: systematic review and meta-analysis. Med Care. 2007;1195–204.

29. Shamliyan TA, Duval S, Mueller C, Wilt TJ, Kane RL. The association of registered nurse staffing levels and patient outcomes: Systematic review and meta-analysis. Med Care. 2007;45(12):1195–204.

30. Assaye AM, Wiechula R, Schultz TJ, Feo R. Impact of nurse staffing on patient and nurse workforce outcomes in acute care settings in low- and middle-income countries: a systematic review. JBI Evid Synth. 2021;19(4):751–93.

31. Wilson S, Bremner A, Hauck Y. Association between nurse staffing and hospitalised children's health outcomes: A systematic review. JBI Libr Syst Rev. 2010;8(8 Suppl):1–15.

32. Aromataris E, Fernandez R, Godfrey CM, Holly C, Khalil H, Tungpunkom P. Summarizing systematic reviews: methodological development, conduct and reporting of an umbrella review approach. JBI Evid Implement. 2015;13(3):132–40.

33. Aromataris E, Fernandez R, Godfrey CM, Holly C, Khalil H, Tungpunkom P. Summarizing systematic reviews: methodological development, conduct and reporting of an umbrella review approach. JBI Evid Implement. 2015;13(3):132–40.

34. Imam A, Obiesie S, Aluvaala J, Maina M, Gathara D, English M. Nurse staffing and patient care outcomes: protocol for an umbrella review to identify evidence gaps for low and middle-income countries. Wellcome Open Res. 2021;6(363):363.

35. Zotero | Your personal research assistant [Internet]. [cited 2021 Oct 22]. Available from: https://www.zotero.org/

36. Ouzzani M, Hammady H, Fedorowicz Z, Elmagarmid A. Rayyan—a web and mobile app for systematic reviews. Syst Rev. 2016;5(1):1–10.

37. Shea BJ, Reeves BC, Wells G, Thuku M, Hamel C, Moran J, et al. AMSTAR 2: a critical appraisal tool for systematic reviews that include randomised or non-randomised studies of healthcare interventions, or both. bmj. 2017;358.

38. World Bank Country and Lending Groups – World Bank Data Help Desk [Internet]. [cited 2021 Jun 26]. Available from: https://datahelpdesk.worldbank.org/knowledgebase/articles/906519-world-bank-country-and-lending-groups

39. Stalpers D, de Brouwer BJM, Kaljouw MJ, Schuurmans MJ. Associations between characteristics of the nurse work environment and five nurse-sensitive patient outcomes in hospitals: a systematic review of literature. Int J Nurs Stud. 2015;52(4):817–35.

40. Hill B. Do nurse staffing levels affect patient mortality in acute secondary care?. Br J Nurs Mark Allen Publ. 2017;26(12):698–704.

For peer review only - http://bmjopen.bmj.com/site/about/guidelines.xhtml

For peer review only

41. Bourgon Labelle J, Audet L-A, Farand P, Rochefort CM. Are hospital nurse staffing practices associated with postoperative cardiac events and death? A systematic review. PloS One. 2019;14(10):e0223979.

42. Lang TA, Hodge M, Olson V, Romano PS, Kravitz RL. Nurse-patient ratios: a systematic review on the effects of nurse staffing on patient, nurse employee, and hospital outcomes. JONA J Nurs Adm. 2004 Aug 7;34(7/8):326–37.

43. Mitchell BG, Gardner A, Stone PW, Hall L, Pogorzelska-Maziarz M. Hospital Staffing and Health Care-Associated Infections: A Systematic Review of the Literature. Jt Comm J Qual Patient Saf. 2018;44(10):613–22.

44. Wilson S, Bremner A, Hauck Y, Finn J. The effect of nurse staffing on clinical outcomes of children in hospital: a systematic review. Int J Evid Based Healthc. 2011;9(2):97–121.

45. Thungjaroenkul P, Cummings GG, Embleton A. The impact of nurse staffing on hospital costs and patient length of stay: a systematic review. Nurs Econ. 2007;25(5):255–65.

46. Engineer LD, Winters BD, Weston CM, Zhang A, Sharma R, Bass E, et al. Hospital characteristics and the agency for healthcare research and quality inpatient quality indicators: A systematic review. J Healthc Qual. 2016;38(5):304–13.

47. Lankshear AJ, Sheldon TA, Maynard A. Nurse staffing and healthcare outcomes: a systematic review of the international research evidence. Adv Nurs Sci. 2005;28(2):163–74.

48. Shin S, Park J-H, Bae S-H. Nurse staffing and hospital-acquired conditions: A systematic review. J Clin Nurs. 2019;28(23–24):4264–75.

49. Kutney-Lee A, Aiken LH. Effect of nurse staffing and education on the outcomes of surgical patients with comorbid serious mental illness. Psychiatr Serv. 2008;59(12):1466–9.

50. Ausserhofer D, Zander B, Busse R, Schubert M, De Geest S, Rafferty AM, et al. Prevalence, patterns and predictors of nursing care left undone in European hospitals: results from the multicountry cross-sectional RN4CAST study. BMJ Qual Saf. 2014;23(2):126–35.

51. Bruyneel L, Li B, Ausserhofer D, Lesaffre E, Dumitrescu I, Smith HL, et al. Organization of hospital nursing, provision of nursing care, and patient experiences with care in Europe. Med Care Res Rev. 2015;72(6):643–64.

52. Rafferty AM, Clarke SP, Coles J, Ball J, James P, McKee M, et al. Outcomes of variation in hospital nurse staffing in English hospitals: cross-sectional analysis of survey data and discharge records. Int J Nurs Stud. 2007;44(2):175–82.

53. Palese A, Ambrosi E, Prosperi L, Guarnier A, Barelli P, Zambiasi P, et al. Missed nursing care and predicting factors in the Italian medical care setting. Intern Emerg Med. 2015;10(6):693–702.

54. Cho S-H, Kim Y-S, Yeon KN, You S-J, Lee ID. Effects of increasing nurse staffing on missed nursing care. Int Nurs Rev. 2015;62(2):267–74.

For peer review only

For peer review only - http://bmjopen.bmj.com/site/about/guidelines.xhtml

55. Friese CR, Kalisch BJ, Lee KH. Patterns and correlates of missed nursing care in inpatient oncology units. Cancer Nurs. 2013;36(6):E51.

56. Aiken LH, Sloane DM, Bruyneel L, Van den Heede K, Griffiths P, Busse R, et al. Nurse staffing and education and hospital mortality in nine European countries: a retrospective observational study. The lancet. 2014;383(9931):1824–30.

57. Magalhães AMM de, Dall'Agnol CM, Marck PB. Nursing workload and patient safety-a mixed method study with an ecological restorative approach. Rev Lat Am Enfermagem. 2013;21:146–54.

58. Hinno S, Partanen P, Vehviläinen-Julkunen K. Nursing activities, nurse staffing and adverse patient outcomes as perceived by hospital nurses. J Clin Nurs. 2012;21(11–12):1584–93.

59. Ball JE, Bruyneel L, Aiken LH, Sermeus W, Sloane DM, Rafferty AM, et al. Post-operative mortality, missed care and nurse staffing in nine countries: a cross-sectional study. Int J Nurs Stud. 2018;78:10–5.

60. Ball JE, Griffiths P, Rafferty AM, Lindqvist R, Murrells T, Tishelman C. A cross-sectional study of 'care left undone'on nursing shifts in hospitals. J Adv Nurs. 2016;72(9):2086–97.

61. Schubert M, Ausserhofer D, Desmedt M, Schwendimann R, Lesaffre E, Li B, et al. Levels and correlates of implicit rationing of nursing care in Swiss acute care hospitals—a cross sectional study. Int J Nurs Stud. 2013;50(2):230–9.

62. McHugh MD, Rochman MF, Sloane DM, Berg RA, Mancini ME, Nadkarni VM, et al. Better nurse staffing and nurse work environments associated with increased survival of in-hospital cardiac arrest patients. Med Care. 2016;54(1):74.

63. Cimiotti JP, Aiken LH, Sloane DM, Wu ES. Nurse staffing, burnout, and health care–associated infection. Am J Infect Control. 2012;40(6):486–90.

64. Aiken LH, Clarke SP, Cheung RB, Sloane DM, Silber JH. Educational levels of hospital nurses and surgical patient mortality. Jama. 2003;290(12):1617–23.

65. Brooks Carthon JM, Kutney-Lee A, Jarrín O, Sloane D, Aiken LH. Nurse staffing and postsurgical outcomes in black adults. J Am Geriatr Soc. 2012;60(6):1078–84.

66. Halm M, Peterson M, Kandels M, Sabo J, Blalock M, Braden R, et al. Hospital nurse staffing and patient mortality, emotional exhaustion, and job dissatisfaction. Clin Nurse Spec. 2005;19(5):241–51.

67. Al-Kandari F, Thomas D. Factors contributing to nursing task incompletion as perceived by nurses working in Kuwait general hospitals. J Clin Nurs. 2009;18(24):3430–40.

68. Cheung RB. The relationship between nurse staffing, nursing time, and adverse events in an acute care hospital. University of South Florida; 2002.

69. Prot S, Fontan JE, Alberti C, Bourdon O, Farnoux C, Macher MA, et al. Drug administration errors and their determinants in pediatric in-patients. Int J Qual Health Care. 2005;17(5):381–9.

For peer review only - http://bmjopen.bmj.com/site/about/guidelines.xhtml

For peer review only

70. Sujijantararat R. Nursing-sensitive quality indicators: Nosocomial urinary tract infection in a Thai hospital. The University of Alabama at Birmingham; 2001.

71. Sujijantararat R, Booth RZ, Davis LL. Nosocomial urinary tract infection: nursing-sensitive quality indicator in a Thai hospital. J Nurs Care Qual. 2005;20(2):134–9.

72. Kouatly IA, Nassar N, Nizam M, Badr LK. Evidence on Nurse Staffing Ratios and Patient Outcomes in a Low-Income Country: Implications for Future Research and Practice. Worldviews Evid Based Nurs. 2018;15(5):353–60.

73. Blegen MA, Goode CJ, Spetz J, Vaughn T, Park SH. Nurse staffing effects on patient outcomes: safety-net and non-safety-net hospitals. Med Care. 2011;406–14.

74. Blegen MA, Vaughn T. A multisite study of nurse staffing and patient occurrences. Nurs Econ. 1998;16(4):196.

75. Everhart D, Schumacher JR, Duncan RP, Hall AG, Neff DF, Shorr RI. Determinants of hospital fall rate trajectory groups: a longitudinal assessment of nurse staffing and organizational characteristics. Health Care Manage Rev. 2014;39(4):352.

76. Needleman J, Buerhaus P, Mattke S, Stewart M, Zelevinsky K. Nurse-staffing levels and the quality of care in hospitals. N Engl J Med. 2002;346(22):1715–22.

77. Trinkoff AM, Johantgen M, Storr CL, Gurses AP, Liang Y, Han K. Nurses' work schedule characteristics, nurse staffing, and patient mortality. Nurs Res. 2011;60(1):1–8.

78. Lichtig LK, Knauf RA, Milholland DK. Some impacts of nursing on acute care hospital outcomes. JONA J Nurs Adm. 1999;29(2):25–33.

79. Kalisch BJ, Doumit M, Lee KH, Zein JE. Missed nursing care, level of staffing, and job satisfaction: Lebanon versus the United States. J Nurs Adm. 2013;43(5):274–9.

80. Yu D, Ma Y, Sun Q, Lu G, Xu P. A nursing care classification system for assessing workload and determining optimal nurse staffing in a teaching hospital in C hina: A pre-post intervention study. Int J Nurs Pract. 2015;21(4):339–49.

81. Zhu X, You L, Zheng J, Liu K, Fang J, Hou S, et al. Nurse staffing levels make a difference on patient outcomes: a multisite study in Chinese hospitals. J Nurs Scholarsh. 2012;44(3):266–73.

82. Sasichay-Akkadechanunt T, Scalzi CC, Jawad AF. The relationship between nurse staffing and patient outcomes. JONA J Nurs Adm. 2003;33(9):478–85.

83. Feleke SA, Mulatu MA, Yesmaw YS. Medication administration error: magnitude and associated factors among nurses in Ethiopia. BMC Nurs. 2015;14(1):1–8.

84. English M, Irimu G, Akech S, Aluvaala J, Ogero M, Isaaka L, et al. Employing learning health system principles to advance research on severe neonatal and paediatric illness in Kenya. BMJ Glob Health. 2021;6(3):e005300.

For peer review only

For peer review only - http://bmjopen.bmj.com/site/about/guidelines.xhtml

85. Ogero M, Ayieko P, Boniface Makone TJ, Malla L, Oliwa J, Irimu G, et al. An observational study of monitoring of vital signs in children admitted to Kenyan hospitals: an insight into the quality of nursing care? J Glob Health. 2018;8(1).

86. Recio-Saucedo A, Dall'Ora C, Maruotti A, Ball J, Briggs J, Meredith P, et al. What impact does nursing care left undone have on patient outcomes? Review of the literature. J Clin Nurs. 2018;27(11–12):2248–59.

For peer review only

For peer review only - http://bmjopen.bmj.com/site/about/guidelines.xhtml

For peer review only

For peer review only - http://bmjopen.bmj.com/site/about/guidelines.xhtml

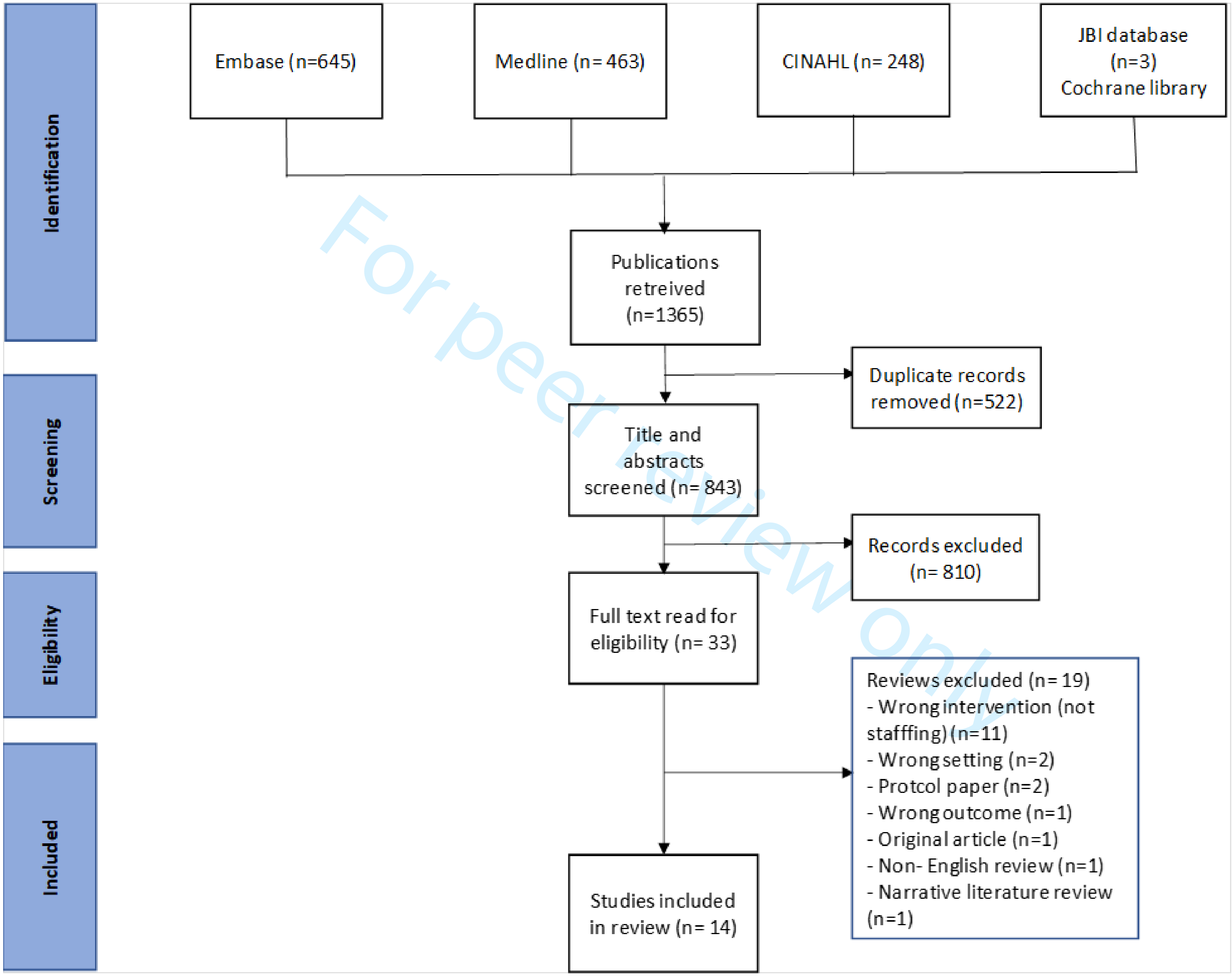

For peer review only

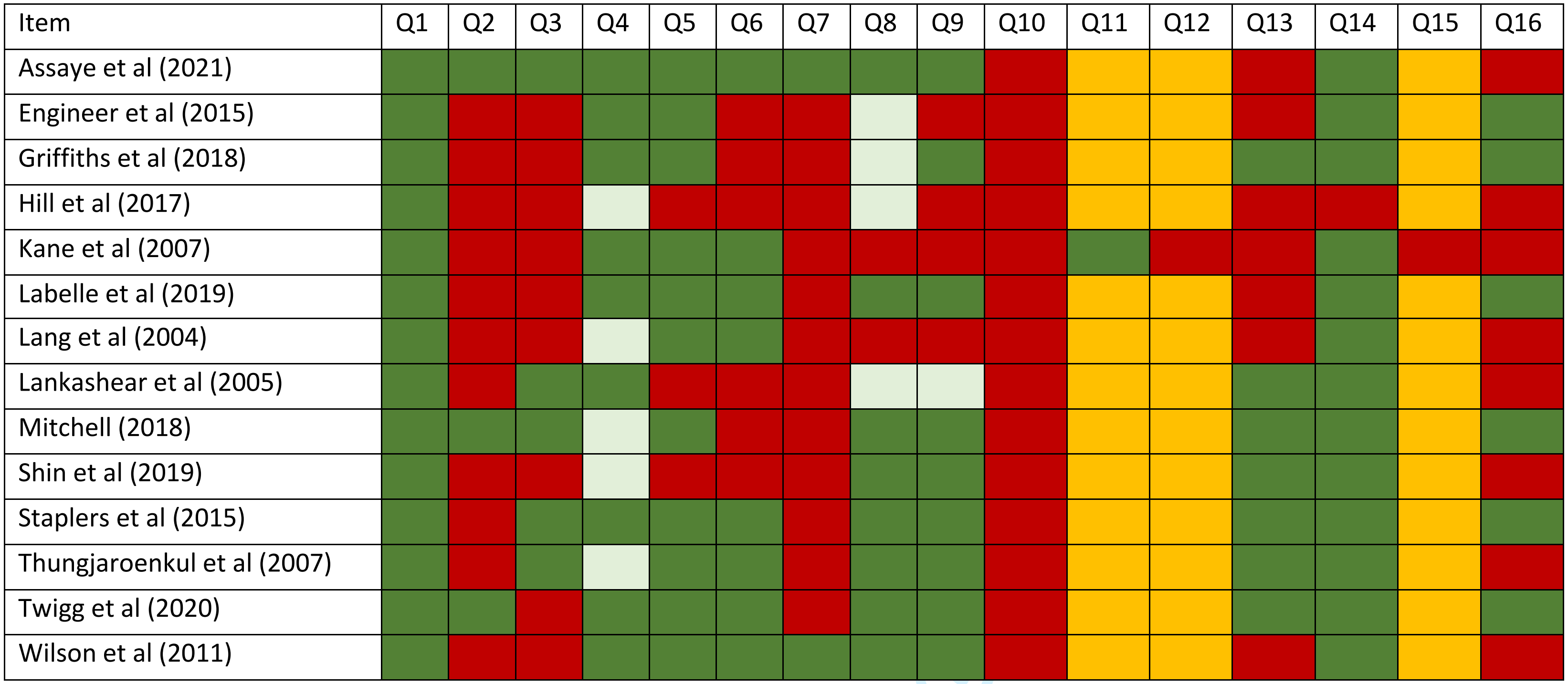

Figure 2 – Risk of Bias assessment using the AMSTAR-2 checklist. (Key: Red – No, Green – Yes, lighter green – Partially Yes, Yellow – Not applicable)

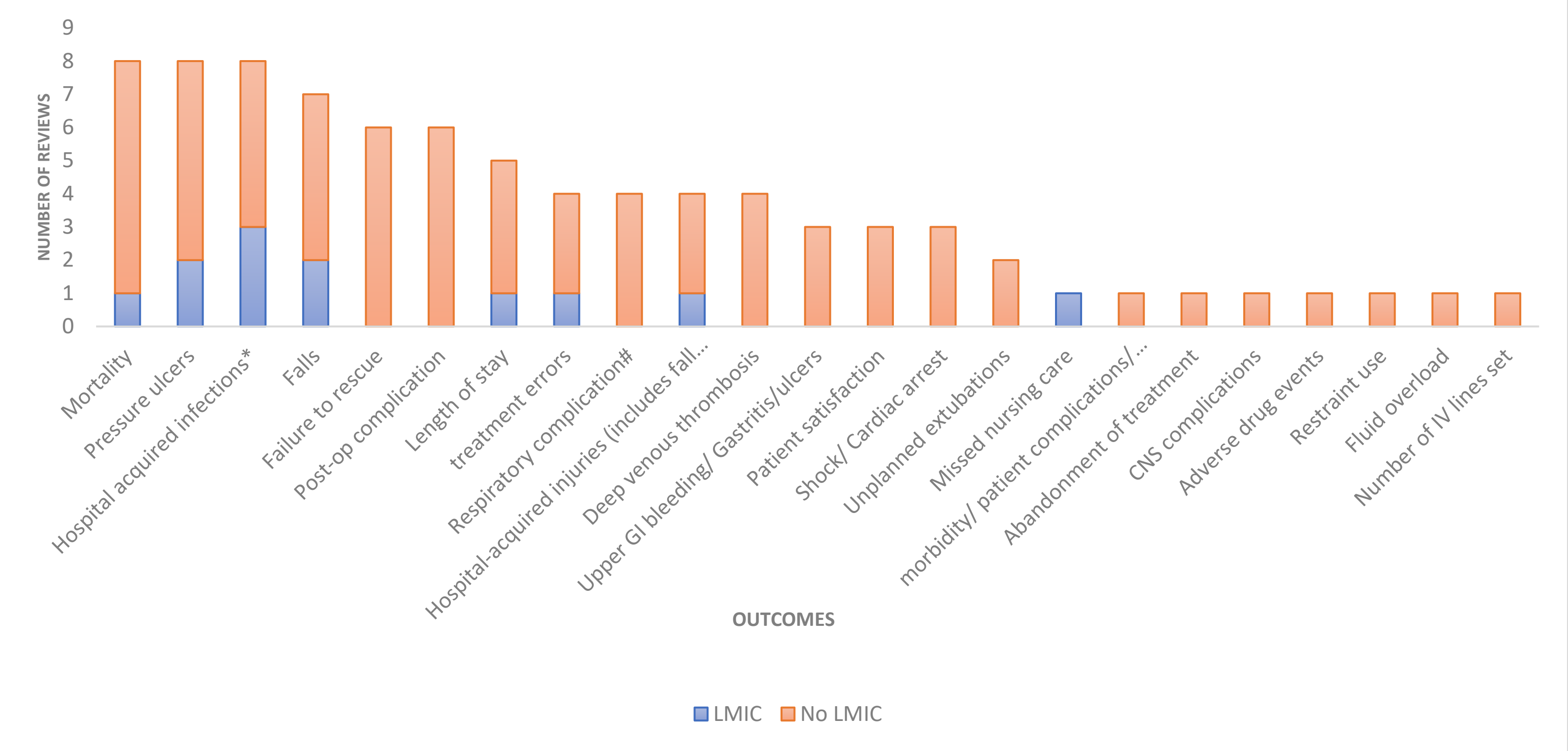

Figure 3: Stacked bar chart showing the range of patient care outcomes reported across reviews and the number of reviews which report this in a LMIC study (Blue- number of reviews reporting the outcome from a LMIC study, Orange – number of reviews reporting the outcome from only non-LMIC studies)

*- Hospital-acquired infections include pneumonia, UTI, central line-associated blood stream infections, nosocomial infections, wound infections

# - respiratory complications include pulmonary compromise/ respiratory failure/ pulmonary embolism)

The least reported patient care outcomes were missed nursing care, patient adverse events, abandonment of treatment, central nervous system complications, adverse drug events, restraint use, fluid overload and the number of intravenous cannulations which were all reported by 1 systematic review each.

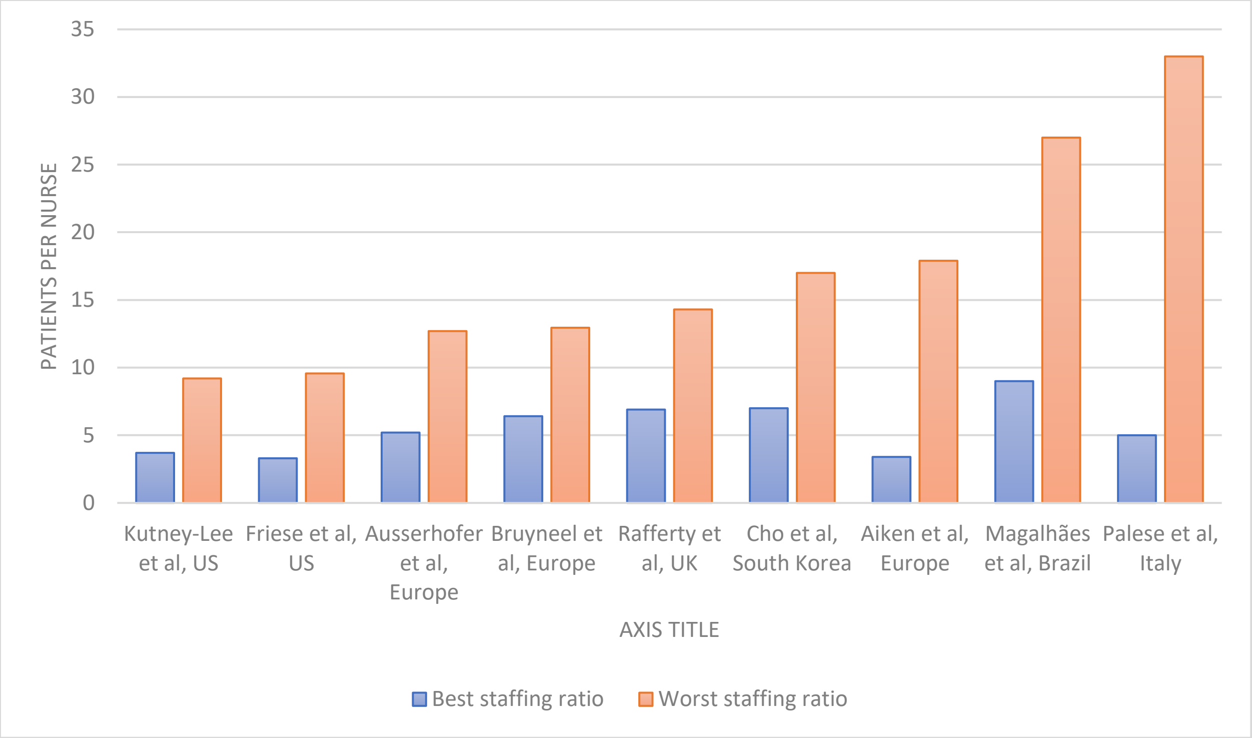

Figure 4 –Bar chart showing the patient to nurse ratio per shift range described in individual studies that reported this metric and described a range (countries of origin and first author of the individual studies are presented, and the Y-axis represents the number of patients per nurse).

The best staffing ratio in the study context is presented in blue while the worst is presented in orange, these best and worst ratios were used in study-specific exploratory of the association between staffing and nursing quality.

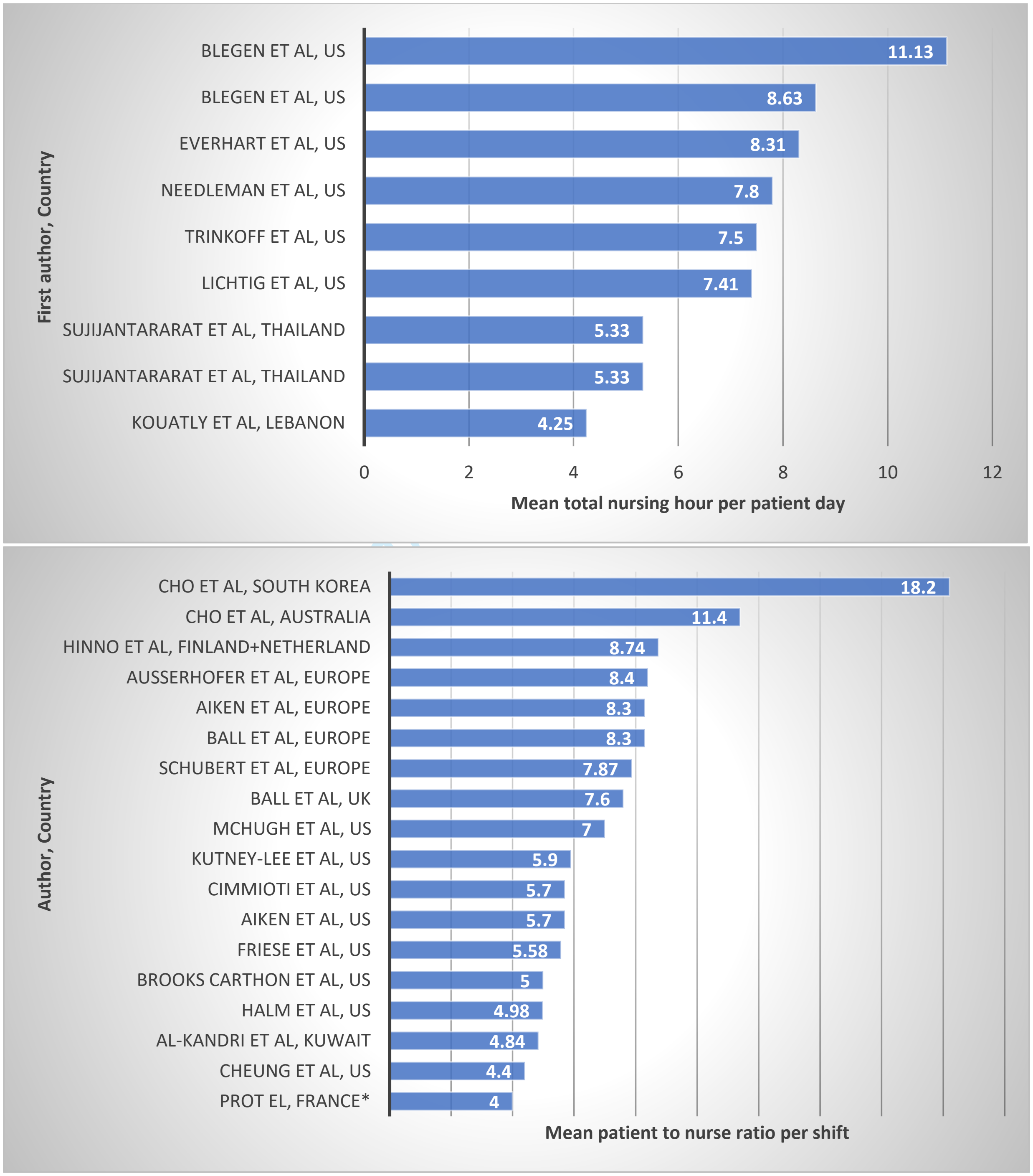

Figure 5 – Horizontal bar charts showing the mean total nursing hour per patient day (top) and the mean patient to nurse ratios (bottom) across primary studies reporting these metrics. (First author and countries in which individual studies were conducted are depicted on the Y-axis).

*- median value

Online supplemental file 1 : Medline and Embase search strategy

1.	Nursing Staff, Hospital/
2.	Nurse's Role/
3.	Specialties, Nursing/
4.	Nurse-Patient Relations/
5.	((nurse* or nursing) adj3 (staff* or ratio or ratios or workforce or workload or sensitive or mix or practitioner* or assistant* or practice* or performance)).ab,kw,ti.
6.	1 or 2 or 3 or 4 or 5
7.	"Personnel Staffing and Scheduling"/
8.	Health Care Rationing/
9.	"Quality of Health Care"/
10.	Quality Indicators, Health Care/
11.	Patient Safety/
12.	Outcome Assessment, Health Care/
13.	(outcome* or impact* or safety or quality or evidence or indicator* or advers* or mortality or death* or omission* or missed or missing or unmet or undone or rationed or rationing).ti.
14.	"missed nursing".ab,kw,ti.
15.	"missed care".ab,kw,ti.
16.	7 or 8 or 9 or 10 or 11 or 12 or 13 or 14 or 15
17.	(systematic and review).ti.
18.	("meta analysis" or metaanalysis).ti.
19.	"Systematic Review"/

20. Meta-Analysis/
21. 17 or 18 or 19 or 20
22. 6 and 16 and 21

Search strategy in CINAHL

S1	MH "NURSING STAFF, HOSPITAL"/ OR MH "NURSE'S ROLE"/ OR MH "SPECIALTIES, NURSING"/ OR MH "NURSE-PATIENT RELATIONS"/ OR (TI "nurse*" N3 "STAFF*") OR (AB "nurse*" N3 "STAFF*") OR (TI "nurse*" N3 "RATIO") OR (AB "nurse*" N3 "RATIO") OR (TI "nurse*" N3 "RATIOS") OR (AB "nurse*" N3 "RATIOS") OR (TI "nurse*" N3 "WORKFORCE") OR (AB "nurse*" N3 "WORKFORCE") OR (TI "nurse*" N3 "WORKLOAD") OR (AB "nurse*" N3 "WORKLOAD") OR (TI "nurse*" N3 "SENSITIVE") OR (AB "nurse*" N3 "SENSITIVE") OR (TI "nurse*" N3 "MIX") OR (AB "nurse*" N3 "MIX") OR (TI "nurse*" N3 "PRACTITIONER*") OR (AB "nurse*" N3 "PRACTITIONER*") OR (TI "nurse*" N3 "ASSISTANT*") OR (AB "nurse*" N3 "ASSISTANT*") OR (TI "nurse*" N3 "PRACTICE*") OR (AB "nurse*" N3 "PRACTICE*") OR (TI "nurse*" N3 "PERFORMANCE") OR (AB "nurse*" N3 "PERFORMANCE") OR (TI "nursing" N3 "STAFF*") OR (AB "nursing" N3 "STAFF*") OR (TI "nursing" N3 "RATIO") OR (AB "nursing" N3 "RATIO") OR (TI "nursing" N3 "RATIOS") OR (AB "nursing" N3 "RATIOS") OR (TI "nursing" N3 "WORKFORCE") OR (AB "nursing" N3 "WORKFORCE") OR (TI "nursing" N3 "WORKLOAD") OR (AB "nursing" N3 "WORKLOAD") OR (TI "nursing" N3 "SENSITIVE") OR (AB "nursing" N3 "SENSITIVE") OR (TI "nursing" N3 "MIX") OR (AB "nursing" N3 "MIX") OR (TI "nursing" N3 "PRACTITIONER*") OR (AB "nursing" N3 "PRACTITIONER*") OR (TI "nursing" N3 "ASSISTANT*") OR (AB "nursing" N3 "ASSISTANT*") OR (TI "nursing" N3 "PRACTICE*") OR (AB "nursing" N3 "PRACTICE*") OR (TI "nursing" N3 "PERFORMANCE") OR (AB "nursing" N3 "PERFORMANCE")
S2	MH "Personnel Staffing and Scheduling"/ OR MH "Health Care Rationing"/ OR MH "Quality of Health Care"/ OR MH "Quality Indicators, Health Care"/ OR MH "Patient Safety"/ OR MH "Outcome Assessment, Health Care"/ OR TI(outcome* OR impact* OR safety OR quality OR evidence OR indicator* OR advers* OR mortality OR death* OR omission* OR missed OR missing OR unmet OR undone OR rationed OR rationing) OR TI ("missed nursing") OR AB ("missed nursing") OR TI ("missed care") OR AB ("missed care")
S3	TI (systematic AND review) OR TI ("meta analysis" OR metaanalysis) OR MH "Systematic Review"/ OR MH "Meta-Analysis/")
S4	S1 AND S2 AND S3

For peer review only

Online supplemental file 2 – List of Excluded systematic reviews after full text screening

First Author	Year	Citation	Reason for exclusion	Explanation
Bae et al	2014	Bae SH, Fabry D. Assessing the relationships between nurse work hours/overtime and nurse and patient outcomes: systematic literature review. Nursing outlook. 2014 Mar 1;62(2):138-56.	Wrong intervention	Intervention was nurse working hours which is not a metric of nurse staffing level
Butler et al	2019	Butler M, Schultz TJ, Halligan P, Sheridan A, Kinsman L, Rotter T, Beaumier J, Kelly RG, Drennan J. Hospital nurse-staffing models and patient-and staff-related outcomes. Cochrane Database of Systematic Reviews. 2019(4).	Wrong intervention	Intervention was nurse staffing models not ratio change or change in other metrics of nurse staffing
Wilson et al	2010	Wilson S, Bremner A, Hauck Y. Association between nurse staffing and hospitalised children's health outcomes: A systematic review. JBI Evidence Synthesis. 2010 Jan 1;8(8):1-5.	Protocol paper	
Recio-Saucedo et al	2018	Recio-Saucedo A, Dall'Ora C, Maruotti A, Ball J, Briggs J, Meredith P, Redfern OC, Kovacs C, Prytherch D, Smith GB, Griffiths P. What impact does nursing care left undone have on patient outcomes? Review of the literature. Journal of clinical nursing. 2018 Jun;27(11-12):2248-59.	Wrong intervention	Impact of missed nursing care on patient care outcomes and not the impact of staffing
Shekelle et al	2013	Shekelle PG. Nurse–patient ratios as a patient safety strategy:	Literature review	Narrative literature review

For peer review only

		a systematic review. Annals of internal medicine. 2013 Mar 5;158(5_Part_2):404-9.		
Papastavrou et al	2014	Papastavrou E, Andreou P, Efstathiou G. Rationing of nursing care and nurse–patient outcomes: a systematic review of quantitative studies. The International journal of health planning and management. 2014 Jan;29(1):3-25.	Wrong intervention	Impact of missed nursing care on patient care outcomes and not the impact of nurse staffing
Driscoll et al	2018	Driscoll A, Grant MJ, Carroll D, Dalton S, Deaton C, Jones I, Lehwaldt D, McKee G, Munyombwe T, Astin F. The effect of nurse-to-patient ratios on nurse-sensitive patient outcomes in acute specialist units: a systematic review and meta-analysis. European Journal of Cardiovascular Nursing. 2018 Jan 1;17(1):6-22.	Wrong study setting (non-ward setting)	All papers from intensive care unit
Garcia et al	2010	García F, Sampietro-Colom L. Nursing staff and patient results: systematic review about the existing relationship. Revista de enfermeria (Barcelona, Spain). 2010 Jan 1;33(1):14-26.	Non-English paper	Spanish
Al-ghraiybah et al	2021	Al-ghraiybah T, Sim J, Lago L. The relationship between the nursing practice environment and five nursing-sensitive patient outcomes in acute care hospitals: A systematic review. Nursing Open. 2021 Mar 4.	Wrong intervention	Intervention is nurse practice environment and not nurse staffing

For peer review only

dit Dariel et al	2015	dit Dariel OP, Regnaux JP. Do Magnet®-accredited hospitals show improvements in nurse and patient outcomes compared to non-Magnet hospitals: a systematic review. JBI Evidence Synthesis. 2015 Jun 1;13(6):168-219.	Wrong intervention	Intervention is magnet accreditation and not nurse staffing
Kazanijan et al	2005	Kazanjian A, Green C, Wong J, Reid R. Effect of the hospital nursing environment on patient mortality: a systematic review. Journal of Health Services Research & Policy. 2005 Apr 1;10(2):111-7A.	Wrong intervention	Intervention is nurse practice environment and not nurse staffing
Johnston et al	2015	Johnston MJ, Arora S, King D, Bouras G, Almoudaris AM, Davis R, Darzi A. A systematic review to identify the factors that affect failure to rescue and escalation of care in surgery. Surgery. 2015 Apr 1;157(4):752-63.	Wrong intervention	Focused on factors that affect failure to rescue and not nurse staffing
Kushemererwa et al	2020	Kushemererwa D, Davis J, Moyo N, Gilbert S, Gray R. The Association between Nursing Skill Mix and Mortality for Adult Medical and Surgical Patients: Protocol for a Systematic Review. International Journal of Environmental Research and Public Health. 2020 Jan;17(22):8604.	Protocol paper	Protocol paper and not original article
Olley et al	2018	Olley R, Edwards I, Avery M, Cooper H. Systematic review of the evidence related to mandated nurse staffing ratios in	Wrong outcome	Outcome is on general evidence and not patient care outcomes

For peer review only

		acute hospitals. Australian Health Review. 2018 Apr 17;43(3):288-93.		
Copanitsanou et al	2017	Copanitsanou P, Fotos N, Brokalaki H. Effects of work environment on patient and nurse outcomes. British Journal of Nursing. 2017 Feb 9;26(3):172-6.	original paper	Original paper and not a systematic review
Myers et al	2018	Myers H, Pugh JD, Twigg DE. Identifying nurse-sensitive indicators for stand-alone high acuity areas: A systematic review. Collegian. 2018 Aug 1;25(4):447-56.	Wrong study setting (non-ward setting)	Intensive care unit
Bae et al	2021	Bae SH. Relationships between comprehensive characteristics of nurse work schedules and adverse patient outcomes: A systematic literature review. Journal of Clinical Nursing. 2021 Feb 22.	Wrong intervention	Intervention is nurses' working schedule and not nurse staffing
Butler et al	2011	Butler M, Collins R, Drennan J, Halligan P, O'Mathuna D, Schultz T, Sheridan A, Vilis E. Hospital nurse staffing models and patient and staff-related outcomes-an epic EPOC systematic review. JBI Evidence Implementation. 2011 Sep 1;9(3):326.	Wrong intervention	Intervention is nurse staffing models not a change in nurse staffing numbers
Bae et al	2011	BAE SH. Assessing the relationships between nurse working conditions and patient outcomes: systematic literature review. Journal of nursing management. 2011 Sep;19(6):700-13.	Wrong intervention	Intervention is nurse working conditions and not nurse staffing

For peer review only

For peer review only

Online supplemental file 3: Excluded primary studies and reasons for exclusion.

systematic review	Title	reason for exclusion
Griffith et al		
	Zander, B., Dobler, L., Bäumler, M. and Busse, R., 2014. Implicit rationing of care services in German acute care hospitals – Results of the international nursing study Rn4cast. Health Care, 76(11), pp.727-734.	German
Assaye et al		
	Soares M, Bozza FA, Angus DC, Japiassu AM, Viana WN, Costa R, et al. Organizational characteristics, outcomes, and resource use in 78 Brazilian intensive care units: the ORCHESTRA study. Intensive Care Med 2015;41(12):2149– 60.	ICU setting
	Silva MCMd, Sousa RMCd, Padilha KG. Factors associated with death and readmission into the Intensive Care Unit. Rev Lat Am Enfermagem 2011;19:911–9.	ICU setting
	Chittawatanarat K, Sataworn D, Thongchai C. Thai Society of Critical Care Medicine Study G. Effects of ICU characters, human resources and workload to outcome indicators in Thai ICUs: the results of ICU-RESOURCE I study. J Med Assoc Thai 2014;97(Suppl 1):S22–30.	ICU setting
	Cremasco MF, Wenzel F, Zanei SSV, Whitaker IY. Pressure ulcers in the intensive care unit: the relationship between nursing workload, illness severity and pressure ulcer risk. J Clin Nurs 2013;22(15/16):2183–91.	ICU setting
	Al Tehewy M, Fahim H, Gad NI, El Gafary M, Rahman SA. Medication administration errors in a university hospital. J Patient Saf 2016;12(1):34–9.	Wrong intervention (number and type of shift)

For peer review only

	Daud-Gallotti RM, Costa SF, Guimara˜es T, Padilha KG, Inoue EN, Vasconcelos TN, et al. Nursing workload as a risk factor for health care associated infections in ICU: a prospective study. PLoS One 2012;7(12):e52342.	ICU setting
	Celen MK, Tamam Y, Hosoglu S, Ayaz C, Geyik MF, Apak I. Multiresistant bacterial colonization due to increased nurse workload in a neurology intensive care unit. Neurosciences 2006;11(4):265–70.	ICU setting
	Aycan IO, Celen MK, Yilmaz A, Almaz MS, Dal T, Celik Y, et al. Bacterial colonization due to increased nurse workload in an intensive care unit. Rev Brasil Anestesiol 2015;65:180–5	ICU setting
	. Cremasco MF, Wenzel F, Zanei SSV, Whitaker IY. Pressure ulcers in the intensive care unit: the relationship between nursing workload, illness severity and pressure ulcer risk. J Clin Nurs 2013;22(15/16):2183–91	ICU setting
	Bjorklund de Lima L, Rejane Rabelo E. Nursing workload in the post-anesthesia care unit. Acta Paul Enferm 2013;26(2): 116–22	Wrong intervention (Nursing workload measure)
	Day SW. Evaluating the impact of the Guatemalan Nursing Program on staff, organizational, and clinical outcomes. University of Tennessee Health Science Center; 2010; p. 93	Wrong intervention (Addition of a nurse educator)
	Ilhan MN, Durukan E, Taner E, Maral I, Bumin MA. Burnout and its correlates among nursing staff: questionnaire survey. J Adv Nurs 2008;61(1):100–6	Nurse outcome (Burn out)
	Karakoc A, Yilmaz M, Alcalar N, Esen B, Kayabasi H, Sit D. Burnout syndrome among hemodialysis and peritoneal dialysis nurses. Iran J Kidney Dis 2016;10(6):395–404.	Nurse outcome (Burn out)
	Roomaney R, Steenkamp J, Kagee A. Predictors of burnout among HIV	Nurse outcome (Burn out)

For peer review only

	nurses in the Western Cape. Curationis 2017;40(1):1–9	
	Kunaviktikul W, Wichaikhum O, Nantsupawat A, Nantsupawat R, Chontawan R, Klunklin A et al. Nurses' extended work hours: patient, nurse and organizational outcomes. Int Nurs Rev 2015;62(3):386–93.	Wrong intervention (Nursing workload measure)
	De Paiva LC, Gomes Cana´rio AC, Corsino de Paiva China EL, Gonc¸alves AK. Burnout syndrome in health-care professionals in a university hospital. Clinics 2017;72(5):305–9.	Nurse outcome (Burn out)
	Lu MM, Ruan H, Xing WJ, Hu Y. Nurse burnout in China: a questionnaire survey on staffing, job satisfaction, and quality of care. J Nurs Manag 2015;23(4):4	Nurse outcome (Burn out)
	Mohammadpoorasl A, Maleki A, Sahebihagh MH. Prevalence of professional burnout and its related factors among nurses in Tabriz in 2010. IJNMR 2012;17(7):524–9	Nurse outcome (Burn out)
	Naz S, Hashmi AM, Asif A. Burnout and quality of life in nurses of a tertiary care hospital in Pakistan. J Pak Med Assoc 2016;66(5):532–6	Nurse outcome (Burn out)
	. Negi Y, Bagga R. Burnout among nursing professionals in tertiary care hospitals of Delhi. J Health Manag 2015;17(2):163–77	Nurse outcome (Burn out)
	Nantsupawat A, Srisuphan W, Kunaviktikul W, Wichaikhum, Aungsuroch Y, Aiken LH. Impact of nurse work environment and staffing on hospital nurse and quality of care in Thailand. J Nurs Scholarsh 2011;43(4):426–32	Nurse outcome (Burn out)
	Nantsupawat A, Nantsupawat R, Kulnaviktikul W, McHugh MD. Relationship between nurse staffing levels and nurse outcomes in community hospitals, Thailand. Nurs Health Sci 2015;17(1):112–8	Nurse outcome (Emotional exhaustion and Needle stick injuries)

For peer review only

Thungjaroenkul P, Cummings GG, Embleton A. The impact of nurse staffing on hospital costs and patient length of stay: a systematic review. Nursing Economics. 2007 Sep 1;25(5):255.		
	Amaravadi, R.K., Dimick, J.B., Pronovost, P.J., & Lipsett, P.A. (2000). ICU nurseto-patient ratio is associated with complications and resource use after esophagectomy. Intensive Care Medicine, 26, 1857-1862.	ICU setting
	Pronovost PJ, Jenckes MW, Dorman T, Garrett E, Breslow MJ, Rosenfeld BA, Lipsett PA, Bass E. Organizational characteristics of intensive care units related to outcomes of abdominal aortic surgery. Jama. 1999 Apr 14;281(14):1310-7.	ICU setting
	Lassnigg A, Hiesmayr M, Bauer P, Haisjackl M. Effect of centre-, patient-and procedure-related factors on intensive care resource utilisation after cardiac surgery. Intensive care medicine. 2002 Oct;28(10):1453-61.	ICU setting
	Dimick, Justin B., et al. "Effect of nurse-to-patient ratio in the intensive care unit on pulmonary complications and resource use after hepatectomy." American Journal of Critical Care 10.6 (2001): 376.	ICU setting

For peer review only

	Bloom JR, Alexander JA, Nuchols BA. Nurse staffing patterns and hospital efficiency in the United States. Social science & medicine. 1997 Jan 1;44(2):147-55.	Wrong outcome (cost of care)
	Lee TY, Yeh ML, Chen HH, Lien GH. The skill mix practice model for nursing: measuring outcome. Journal of advanced nursing. 2005 Aug;51(4):406-13.	Wrong outcome (cost of care)
	Hall LM, Doran D, Pink GH. Nurse staffing models, nursing hours, and patient safety outcomes. JONA: The Journal of Nursing Administration. 2004 Jan 1;34(1):41-5.	Wrong outcome (cost of care)
	Pratt RA, Burr G, Leelarthaepin B, Blizard P, Walsh S. The effects of All-RN and RN-EN staffing on the quality and cost of patient care. The Australian journal of advanced nursing: a quarterly publication of the Royal Australian Nursing Federation. 1993 Mar 1;10(3):27-39.	Wrong intervention (skill mix)
	Newhouse RP, Johantgen M, Pronovist PJ, Johnson E. Perioperative nurses and patient outcomes: mortality, complications, and length of stay. The Journal of nursing administration. 2010 Oct 1;40(10 Suppl):S54-67.	Wrong intervention (skill mix)
	Needleman J, Buerhaus PI, Stewart M, Zelevinsky K, Mattke S. Nurse staffing in hospitals: is there a business case for quality?. Health Affairs. 2006 Jan;25(1):204-11.	Wrong intervention (costs)
	Barkell NP, Killinger KA, Schultz SD. The relationship between nurse staffing models and patient outcomes: a descriptive study. Outcomes Management. 2002 Jan 1;6(1):27-33.	Wrong intervention (skill mix)
	McCue M, Mark BA, Harless DW. Nurse staffing, quality, and financial performance. Journal of health care finance. 2003 Jan 1;29(4):54-76.	Wrong outcome (cost of care)

For peer review only

Twigg DE, Whitehead L, Doleman G, El-Zaemey S. The impact of nurse staffing methodologies on nurse and patient outcomes: A systematic review. Journal of Advanced Nursing. 2021 Jun 3.		
	Bowblis, J. R., & Ghattas, A. (2017). The impact of minimum quality standard regulations on nursing home staffing, quality, and exit decisions. Review of Industrial Organization, 50(1), 43–68. https://doi. org/10.1007/s11151-016-9528-x	nursing home
	Chan, T. C., Killeen, J. P., Vilke, G. M., Marshall, J. B., & Castillo, E. M. (2010). Effect of mandated nurse–patient ratios on patient wait time and care time in the emergency department. Academic Emergency Medicine, 17(5), 545–552. https://doi. org/10.1111/j.1553-2712.2010.00727.x	Emergency department
	Chen, M. M., & Grabowski, D. C. (2015). Intended and unintended consequences of minimum staffing standards for nursing homes. Health Economics, 24(7), 822–839. https://doi.org/10.1002/hec.3063	nursing home
	Cox, K. S., Anderson, S. C., Teasley, S. L., Sexton, K. A., & Carroll, C. A. (2005). Nurses' work environment perceptions when employed in states with and without mandatory staffing ratios and/or mandatory staffing plans. Policy, Politics, &	Nurse outcomes

For peer review only

	Nursing Practice, 6(3), 191–197. https://doi.org/10.1177/1527154405279091	
	Hodgson, A., Morgan, D., & Peterson, R. (2016). Does better nurse staffing improve detection of depression and anxiety as secondary conditions in hospitalized patients with pneumonia? Nursing Economics, 34(3), 134	Mental health outcome
	Law, A. C., Stevens, J. P., Hohmann, S., & Walkey, A. J. (2018). Patient outcomes after the introduction of statewide intensive care unit nurse staffing regulations. Critical Care Medicine, 46(10), 1563.	ICU setting
	Leigh, J. P., Markis, C. A., Iosif, A.-M., & Romano, P. S. (2015). California's nurse-to-patient ratio law and occupational injury. International Archives of Occupational and Environmental Health, 88(4), 477–484.	Nurse outcome
	Park, J., & Stearns, S. C. (2009). Effects of state minimum staffing standards on nursing home staffing and quality of care. Health Services Research, 44(1), 56–78. https://doi.org/10.1111/j.1475-6773.2008.00906.x	nursing home
	Spetz J, Herrera C. Changes in nurse satisfaction in California, 2004 to 2008. Journal of Nursing Management. 2010 Jul;18(5):564-72.	nurse outcome
	Spetz, J. (2008). Nurse satisfaction and the implementation of minimum nurse staffing regulations. Policy, Politics, & Nursing Practice, 9(1), 15–21. https://doi.org/10.1177/1527154408316950	Nurse outcome
	Tellez, M. (2012). Work satisfaction among California registered nurses: A longitudinal comparative analysis. Nursing economic$, 30(2), 73–81	Nurse outcome

For peer review only - http://bmjopen.bmj.com/site/about/guidelines.xhtml

	Tellez, M., & Seago, J. A. (2013). California nurse staffing law and RN workforce changes. Nursing Economics, 31(1), 18	Nurse outcome
	Matsudaira, J. D. (2014). Government regulation and the quality of healthcare evidence from minimum staffing legislation for nursing homes. Journal of Human Resources, 49(1), 32–72. https://doi.org/10.1353/jhr.2014.0003	nursing home
	Weichenthal, L., & Hendey, G. W. (2011). The effect of mandatory nurse ratios on patient care in an emergency department. The Journal of Emergency Medicine, 40(1), 76–81. https://doi.org/10.1016/j.jemermed.2009.02.037	Emergency department
	Zhang, X., & Grabowski, D. C. (2004). Nursing home staffing and quality under the nursing home reform act. The Gerontologist, 44(1), 13–23. https://doi.org/10.1093/geront/44.1.13	nursing home
Shin et al 2019		
	UK Neonatal Staffing Study Group. Patient volume, staffing, and workload in relation to risk-adjusted outcomes in a random stratified sample of UK neonatal intensive care units: a prospective evaluation. The Lancet. 2002 Jan 12;359(9301):99-107.	ICU setting
	Brooks-Carthon JM, Kutney-Lee A, Sloane DM, Cimiotti JP, Aiken LH. Quality of care and patient satisfaction in hospitals with high concentrations of black patients. Journal of Nursing Scholarship. 2011 Sep;43(3):301-10.	wrong intervention (practice environment)
Lang 2004		
	Kustaborder MJ, Rigney M. Interventions for safety.	wrong intervention (nurse staffing mix)

For peer review only

	Bostrom J, Zimmerman J. Restructuring nursing for a competitive health care environment. Nursing economic$. 1993 Jan 1;11(1):35-41.	wrong intervention (nurse restructuring/ skill mix)
	Needleman J, Buerhaus P. Nurse staffing and patient safety: current knowledge and implications for action. International Journal for Quality in Health Care. 2003 Aug 1;15(4):275-7.	editorial
	Behner KG, Fogg LF, Fournier LC, Frankenbach JT, Robertson SB. Nursing resource management: analyzing the relationship between costs and quality in staffing decisions. Health Care Management Review. 1990 Jan 1;15(4):63-71.	wrong intervention (labour efficiency)
	Shukla RK. All-RN model of nursing care delivery: A cost-benefit evaluation. Inquiry. 1983 Jul 1:173-84.	wrong intervention
	Arndt M, Crane S. Influences on nursing care volume. Journal of the Society for Health Systems. 1998 Jan 1;5(4):38-49.	nurse outcome
	Hinshaw AS, Scofield R, Atwood JR. Staff, patient, and cost outcomes of all-registered nurse staffing. JONA: The Journal of Nursing Administration. 1981 Nov 1;11(11):30-6.	Wrong intervention (skill mix)
	Grillo-Peck AM, Risner PB. The effect of a partnership model on quality and length of stay. Nursing economic$. 1995 Nov 1;13(6):367-72.	wrong intervention (nurse partnership model)
	Ceria CD. Nursing absenteeism and its effects on the quality of patient care. The Journal of nursing administration. 1992 Dec;22(12):11-38.	wrong intervention (absenteeism)
	Bloom JR, Alexander JA, Nuchols BA. The effect of the social organization of work on the voluntary turnover rate of hospital nurses in the United	nurse outcome

For peer review only

	States. Soc Sci Med. 1992;34:1413-1424.	
	Lanza ML, Kayne HL, Gulliford D, et al. Staffing of inpatient psychiatric units and assault by patients. J Am Psychiatr Nurses Assoc. 1997;3:42-4	nurse outcome
	Shortell SM, Hughes EF. The effects of regulation, competition, and ownership on mortality rates among hospital inpatients. New England Journal of Medicine. 1988 Apr 28;318(17):1100-7.	wrong intervention
	Clarke SP, Sloane DM, Aiken LH. Effects of hospital staffing and organizational climate on needlestick injuries to nurses. American journal of public health. 2002 Jul;92(7):1115-9.	nurse outcome
	GLANDON GL, COLBERT KW, Thomasma M. Nursing delivery models and RN mix: cost implications. Nursing Management. 1989 May 1;20(5):30-3.	nurse outcome
	Halloran EJ. RN staffing: more care—less cost. Nursing Management. 1983 Sep 1;14(9):18-23.	nurse outcome
	Osinski EG, Powals JG. The cost of all RN staffed primary nursing. Supervisor nurse. 1980 Jan;11(1):16-21.	nurse outcome
	Bradbury RC, Golec JH, Steen PM. Relating hospital health outcomes and resource expenditures. Inquiry. 1994 Apr 1:56-65.	Wrong intervention (nurse skill mix)
	Krakauer H, Bailey RC, Skellan KJ, Stewart JD, Hartz AJ, Kuhn EM, Rimm AA. Evaluation of the HCFA model for the analysis of mortality following hospitalization. Health services research. 1992 Aug;27(3):317.	Wrong intervention (nurse skill mix)
	Kuhn EM, Hartz A, Gottlieb MS, Rimm AA. The relationship of hospital characteristics and the results of peer review in six large	nurse outcome

For peer review only

	states. Med Care. 1991;29:1028-1038.	
Kane 2007		
	Fridkin SK, Pear SM, Williamson TH, Galgiani JN, Jarvis WR. The role of understaffing in central venous catheter-associated bloodstream infection. Infection Control & Hospital Epidemiology. 1996 Mar;17(3):150-8.	ICU setting
	Amaravadi RK, Dimick JB, Pronovost PJ, et al. ICU nurse-to-patient ratio is associated with complications and resource use after esopha- gectomy. Intensive Care Med. 2000;26: 18	ICU setting
	Dang D, Johantgen ME, Pronovost PJ, et al. Postoperative complica-tions: does intensive care unit staff nursing make a difference? Heart Lung. 2002;3 1:219-	ICU setting
	Dimick JB, Swoboda SM, Pronovost PJ, et al. Effect of nurse-to-patient ratio in the intensive care unit on pulmonary complications and re-source use after hepatectomy. Am J Crit Care. 2001	ICU setting
	Pronovost PJ, Dang D, Dorman T, et al. Intensive care unit nurse staffing and the risk for complications after abdominal aortic surgery. EffClin Pract. 2001 ;4: 19	ICU setting
	Pronovost PJ, Jenckes MW, Dorman T, et al. Organizational charac-teristics of intensive care units related to outcomes of abdominal aortic surgery. JAMA. 1999;281:1310-1	ICU setting
	Shortell SM, Zimmerman JE, Rousseau DM, et al. The performance of intensive care units: does good management make a difference? Med Care. 1994;32:508-525.	ICU setting
	Cimiotti JP, Haas J, Saiman L, et al. Impact of staffing on bloodstream infections in the neonatal intensive	ICU setting

For peer review only

	care unit. Arch Pediatr Adolesc Med. 2006;160:832-836	
	Marcin JP, Rutan E, Rapetti PM, et al. Nurse staffing and unplanned extubation in the pediatric intensive care unit. Pediatr Crit Care Med. 2005;6:254-257.	ICU setting
	Cimiotti JP. Nurse Staffing and Healthcare-Associated Infections in the Neonatal ICU [dissertation]. Ann Arbor, MI: Columbia University; 2004: AAT 3128935.	ICU setting
	Robert J, Fridkin SK, Blumberg HM, Anderson B, White N, Ray SM, Chan J, Jarvis WR. The influence of the composition of the nursing staff on primary bloodstream infection rates in a surgical intensive care unit. Infection Control & Hospital Epidemiology. 2000 Jan;21(1):12-7.	ICU setting
Labelle 2019		
	Dang D, Johantgen ME, Pronovost PJ, Jenckes MW, Bass EB. Postoperative complications: does intensive care unit staff nursing make a difference?. Heart & Lung. 2002 May 1;31(3):219-28.	ICU setting
	Rao AD, Kumar A, McHugh M. Better nurse autonomy decreases the odds of 30-day mortality and failure to rescue. Journal of Nursing Scholarship. 2017 Jan;49(1):73-9.	wrong intervention (nurse work environment)
	Diya L, Van den Heede K, Sermeus W, Lesaffre E. The relationship between in-hospital mortality, readmission into the intensive care nursing unit and/or operating theatre and nurse staffing levels. Journal of advanced nursing. 2012 May;68(5):1073-81.	ICU setting
	Hickey PA, Gauvreau K, Jenkins K, Fawcett J, Hayman L. Statewide and national impact of California's staffing law on pediatric cardiac surgery outcomes. JONA: The	ICU setting

	Journal of Nursing Administration. 2011 May 1;41(5):218-25.	
	Hickey P, Gauvreau K, Connor J, Sporing E, Jenkins K. The relationship of nurse staffing, skill mix, and Magnet® recognition to institutional volume and mortality for congenital heart surgery. JONA: The Journal of Nursing Administration. 2010 May 1;40(5):226-32.	ICU setting
	Dimick JB, Swoboda SM, Pronovost PJ, Lipsett PA. Effect of nurse-to-patient ratio in the intensive care unit on pulmonary complications and resource use after hepatectomy. American Journal of Critical Care. 2001 Nov 1;10(6):376.	ICU setting
	Kiekkas P, Sakellaropoulos GC, Brokalaki H, Manolis E, Samios A, Skartsani C, Baltopoulos GI. Association between nursing workload and mortality of intensive care unit patients. Journal of nursing scholarship. 2008 Dec;40(4):385-90.	ICU setting
	Olds DM, Aiken LH, Cimiotti JP, Lake ET. Association of nurse work environment and safety climate on patient mortality: A cross-sectional study. International journal of nursing studies. 2017 Sep 1;74:155-61.	wrong intervention (nurse work environment)
	Kendall-Gallagher D, Aiken LH, Sloane DM, Cimiotti JP. Nurse specialty certification, inpatient mortality, and failure to rescue. Journal of Nursing Scholarship. 2011 Jun;43(2):188-94.	wrong intervention (nurse education)
	Lane-Fall MB, Ramaswamy TS, Brown SE, He X, Gutsche JT, Fleisher LA, Neuman MD. Structural, nursing, and physician characteristics and 30-day mortality for patients undergoing cardiac	wrong intervention (nurse education)

For peer review only

	surgery in Pennsylvania. Critical care medicine. 2017 Sep;45(9):1472.	
	Diya L, Lesaffre E, Van den Heede K, Sermeus W, Vleugels A. Establishing the relationship between nurse staffing and hospital mortality using a clustered discrete-time logistic model. Statistics in medicine. 2010 Mar 30;29(7-8):778-85.	ICU setting
	Aiken LH, Clarke SP, Cheung RB, Sloane DM, Silber JH. Educational levels of hospital nurses and surgical patient mortality. Jama. 2003 Sep 24;290(12):1617-23.	wrong intervention (nurse education)
	Aiken LH, Clarke SP, Sloane DM, International Hospital Outcomes Research Consortium. Hospital staffing, organization, and quality of care: cross-national findings. International Journal for quality in Health care. 2002 Feb 1;14(1):5-14.	wrong outcome (nurse outcomes)
	Ozdemir BA, Sinha S, Karthikesalingam A, Poloniecki JD, Pearse RM, Grocott MP, Thompson MM, Holt PJ. Mortality of emergency general surgical patients and associations with hospital structures and processes. BJA: British Journal of Anaesthesia. 2016 Jan 1;116(1):54-62.	Emergency department
	Newhouse RP, Johantgen M, Pronovost PJ, Johnson E. Perioperative nurses and patient outcomes—mortality, complications, and length of stay. AORN journal. 2005 Mar 1;81(3):508-28.	Operating room/peri-operative setting
	Aiken LH, Sloane D, Griffiths P, Rafferty AM, Bruyneel L, McHugh M, Maier CB, Moreno-Casbas T, Ball JE, Ausserhofer D, Sermeus W. Nursing skill mix in European hospitals: cross-sectional study of the association with mortality, patient ratings, and quality of care.	wrong intervention (nursing skill mix)

For peer review only

	BMJ quality & safety. 2017 Jul 1;26(7):559-68.	
Wilson 2011		
	Archibald LK, Manning ML, Bell LM, Banerjee S, Jarvis WR. Patient density, nurse-to-patient ratio and nosocomial infection risk in a pediatric cardiac intensive care unit. The Pediatric infectious disease journal. 1997 Nov 1;16(11):1045-8.	ICU setting
	Cimiotti JP. Nurse staffing and healthcare-associated infections in the neonatal ICU. Columbia University; 2004	ICU setting
	Marcin JP, Rutan E, Rapetti PM, et al. Nurse staffing and unplanned extubation in the pediatric intensive care unit. Pediatr Crit Care Med. 2005;6:254-257.	ICU setting
Engineer 2015		
	Volpp KG, Rosen AK, Rosenbaum PR, Romano PS, Even-Shoshan O, Wang Y, Bellini L, Behringer T, Silber JH. Mortality among hospitalized Medicare beneficiaries in the first 2 years following ACGME resident duty hour reform. Jama. 2007 Sep 5;298(9):975-83.	wrong intervention
	Bradley EH, Herrin J, Curry L, Cherlin EJ, Wang Y, Webster TR, Drye EE, Normand SL, Krumholz HM. Variation in hospital mortality rates for patients with acute myocardial infarction. The American journal of cardiology. 2010 Oct 15;106(8):1108-12.	wrong intervention
	Weiner BJ, Alexander JA, Shortell SM, Baker LC, Becker M, Geppert JJ. Quality improvement implementation and hospital performance on quality indicators. Health services research. 2006 Apr;41(2):307-34.	wrong intervention

For peer review only

	Gaskin DJ, Spencer CS, Richard P, Anderson G, Powe NR, LaVeist TA. Do minority patients use lower quality hospitals?. INQUIRY: The Journal of Health Care Organization, Provision, and Financing. 2011 Aug;48(3):209-20.	wrong intervention
	Carretta HJ, Chukmaitov A, Tang A, Shin J. Examination of hospital characteristics and patient quality outcomes using four inpatient quality indicators and 30-day all-cause mortality. American Journal of Medical Quality. 2013 Jan;28(1):46-55.	wrong intervention
	Chukmaitov AS, Bazzoli GJ, Harless DW, Hurley RE, Devers KJ, Zhao M. Variations in inpatient mortality among hospitals in different system types, 1995 to 2000. Medical care. 2009 Apr 1:466-73.	wrong intervention
	Friedman B, Jiang HJ. Do Medicare Advantage enrollees tend to be admitted to hospitals with better or worse outcomes compared with fee-for-service enrollees?. International journal of health care finance and economics. 2010 Jun;10(2):171-85.	wrong intervention
	Romley JA, Jena AB, Goldman DP. Hospital spending and inpatient mortality: evidence from California: an observational study. Annals of internal medicine. 2011 Feb 1;154(3):160-7.	wrong intervention
	Ross JS, Normand SL, Wang Y, Nallamothu BK, Lichtman JH, Krumholz HM. Hospital remoteness and thirty-day mortality from three serious conditions. Health Affairs. 2008 Nov;27(6):1707-17.	wrong intervention
	Ross JS, Normand SL, Wang Y, Ko DT, Chen J, Drye EE, Keenan PS, Lichtman JH, Bueno H, Schreiner GC, Krumholz HM. Hospital volume and 30-day mortality for three common medical conditions. New England	wrong intervention

For peer review only

	Journal of Medicine. 2010 Mar 25;362(12):1110-8.	
	Sari N. Do competition and managed care improve quality?. Health economics. 2002 Oct;11(7):571-84.	wrong intervention
	Shahian DM, Nordberg P, Meyer GS, Blanchfield BB, Mort EA, Torchiana DF, Normand SL. Contemporary performance of US teaching and nonteaching hospitals. Academic Medicine. 2012 Jun 1;87(6):701-8.	wrong intervention
	Volpp KG, Rosen AK, Rosenbaum PR, Romano PS, Even-Shoshan O, Wang Y, Bellini L, Behringer T, Silber JH. Mortality among hospitalized Medicare beneficiaries in the first 2 years following ACGME resident duty hour reform. Jama. 2007 Sep 5;298(9):975-83.	wrong intervention
	Menachemi N, Chukmaitov A, Saunders C, Brooks RG. Hospital quality of care: does information technology matter? The relationship between information technology adoption and quality of care. Health care management review. 2008 Jan 1;33(1):51-9.	wrong intervention
Staplers et al		
	Kendall-Gallagher D, Blegen MA. Competence and certification of registered nurses and safety of patients in intensive care units. American Journal of Critical Care. 2009 Mar;18(2):106-13	ICU setting
	Hall LM, Doran D, Pink GH. Nurse staffing models, nursing hours, and patient safety outcomes. JONA: The Journal of Nursing Administration. 2004 Jan 1;34(1):41-5.	wrong intervention (skill mix)
	Bae SH, Mark B, Fried B. Impact of nursing unit turnover on patient outcomes in hospitals. Journal of Nursing Scholarship. 2010 Mar;42(1):40-9.	wrong intervention (turn over rates)

For peer review only

	Bae SH, Mark B, Fried B. Use of temporary nurses and nurse and patient safety outcomes in acute care hospital units. Health care management review. 2010 Oct 1;35(4):333-44.	wrong intervention (skill mix)
	Chang YK, Hughes LC, Mark B. Fitting in or standing out: Nursing workgroup diversity and unit-level outcomes. Nursing Research. 2006 Nov 1;55(6):373-80.	wrong intervention
	Krapohl G, Manojlovich M, Redman R, Zhang L. Nursing specialty certification and nursing-sensitive patient outcomes in the intensive care unit. American Journal of Critical Care. 2010 Nov;19(6):490-8.	wrong intervention
	Mallidou AA, Cummings GG, Estabrooks CA, Giovannetti PB. Nurse specialty subcultures and patient outcomes in acute care hospitals: A multiple-group structural equation modeling. International journal of nursing studies. 2011 Jan 1;48(1):81-93.	wrong intervention
	Manojlovich M, Antonakos CL, Ronis DL. Intensive care units, communication between nurses and physicians, and patients' outcomes. American Journal of Critical Care. 2009 Jan;18(1):21-30.	wrong intervention
	Manojlovich M, Sidani S, Covell CL, Antonakos CL. Nurse dose: linking staffing variables to adverse patient outcomes. Nursing research. 2011 Jul 1;60(4):214-20.	wrong intervention
	Purdy N, SPENCE LASCHINGER HK, Finegan J, Kerr M, Olivera F. Effects of work environments on nurse and patient outcomes. Journal of nursing management. 2010 Nov;18(8):901-13.	wrong intervention
	Stone PW, Mooney-Kane C, Larson EL, Horan T, Glance LG, Zwanziger J, Dick AW. Nurse working conditions and patient safety outcomes. Medical care. 2007 Jun 1:571-8.	ICU setting

For peer review only

	Wolf D, Lehman L, Quinlin R, Rosenzweig M, Friede S, Zullo T, Hoffman L. Can nurses impact patient outcomes using a patient-centered care model?. JONA: The Journal of Nursing Administration. 2008 Dec 1;38(12):532-40.	ICU setting
Lankashear et al		
	Aiken LH, Smith HL, Lake ET. Lower Medicare mortality among a set of hospitals known for good nursing care. Medical care. 1994 Aug 1:771-87.	wrong intervention (magnet versus non-magnet)
	Jarman B, Gault S, Alves B, Hider A, Dolan S, Cook A, Hurwitz B, Iezzoni LI. Explaining differences in English hospital death rates using routinely collected data. Bmj. 1999 Jun 5;318(7197):1515-20.	wrong intervention (Used other clinical staffing (doctors))
	Hall LM, Doran D, Pink GH. Nurse staffing models, nursing hours, and patient safety outcomes. JONA: The Journal of Nursing Administration. 2004 Jan 1;34(1):41-5.	skill mix
Mitchell et al		
	Andersen BM, Rasch M, Hochlin K, Tollefsen T, Sandvik L. Hospital-acquired infections before and after healthcare reorganization in a tertiary university hospital in Norway. Journal of public health. 2009 Mar 1;31(1):98-104.	wrong intervention (nursing workload)
	Geubbels EL, Wille JC, Nagelkerke NJ, Vandenbroucke-Grauls CM, Grobbee DE, de Boer AS. Hospital-related determinants for surgical-site infection following hip arthroplasty. Infection Control & Hospital Epidemiology. 2005 May;26(5):435-41.	Wrong intervention
	Roche M, Duffield C, Aisbett C, Diers D, Stasa H. Nursing work directions in Australia: Does evidence drive the policy?. Collegian. 2012 Dec 1;19(4):231-8.	wrong intervention (skill mix)

For peer review only

	Kelly D, Kutney-Lee A, Lake ET, Aiken LH. The critical care work environment and nurse-reported health care–associated infections. American Journal of Critical Care. 2013 Nov;22(6):482-8.	ICU setting
	Dimick JB, Swoboda SM, Pronovost PJ, Lipsett PA. Effect of nurse-to-patient ratio in the intensive care unit on pulmonary complications and resource use after hepatectomy. American Journal of Critical Care. 2001 Nov 1;10(6):376.	ICU setting
	Berney B, Needleman J. Impact of nursing overtime on nurse-sensitive patient outcomes in New York hospitals, 1995-2000. Policy, Politics, & Nursing Practice. 2006 May;7(2):87-100.	wrong intervention (nurses overtime)
	Alonso-Echanove J, Edwards JR, Richards MJ, Brennan P, Venezia RA, Keen J, Ashline V, Kirkland K, Chou E, Hupert M, Veeder AV. Effect of nurse staffing and antimicrobial-impregnated central venous catheters on the risk for bloodstream infections in intensive care units. Infection Control & Hospital Epidemiology. 2003 Dec;24(12):916-25.	ICU setting
	Cimiotti JP, Haas J, Saiman L, Larson EL. Impact of staffing on bloodstream infections in the neonatal intensive care unit. Archives of pediatrics & adolescent medicine. 2006 Aug 1;160(8):832-6.	ICU setting
	Pronovost PJ, Dang D, Dorman T, Lipsett PA, Garrett E, Jenckes M, Bass EB. Intensive care unit nurse staffing and the risk for complications after abdominal aortic surgery. Effective clinical practice: ECP. 2001 Sep 1;4(5):199-206.	ICU setting
	Robert J, Fridkin SK, Blumberg HM, Anderson B, White N, Ray SM, Chan J, Jarvis WR. The influence of the	ICU setting

	composition of the nursing staff on primary bloodstream infection rates in a surgical intensive care unit. Infection Control & Hospital Epidemiology. 2000 Jan;21(1):12-7.	
	UK Neonatal Staffing Study Group. Patient volume, staffing, and workload in relation to risk-adjusted outcomes in a random stratified sample of UK neonatal intensive care units: a prospective evaluation. The Lancet. 2002 Jan 12;359(9301):99-107.	ICU setting
	Hugonnet S, Uçkay I, Pittet D. Staffing level: a determinant of late-onset ventilator-associated pneumonia. Critical Care. 2007 Aug;11(4):1-7.	ICU setting
	Bae SH, Brewer CS, Kelly M, Spencer A. Use of temporary nursing staff and nosocomial infections in intensive care units. Journal of clinical nursing. 2015 Apr;24(7-8):980-90.	ICU setting
	Kendall-Gallagher D, Blegen MA. Competence and certification of registered nurses and safety of patients in intensive care units. American Journal of Critical Care. 2009 Mar;18(2):106-13.	ICU setting
	Stone PW, Mooney-Kane C, Larson EL, Horan T, Glance LG, Zwanziger J, Dick AW. Nurse working conditions and patient safety outcomes. Medical care. 2007 Jun 1:571-8.	ICU setting
	Schwab F, Meyer E, Geffers C, Gastmeier P. Understaffing, overcrowding, inappropriate nurse: ventilated patient ratio and nosocomial infections: which parameter is the best reflection of deficits?. Journal of Hospital Infection. 2012 Feb 1;80(2):133-9.	ICU setting
	Amaravadi RK, Dimick JB, Pronovost PJ, Lipsett PA. ICU nurse-to-patient ratio is associated with complications and resource use	ICU setting

For peer review only

	after esophagectomy. Intensive care medicine. 2000 Dec;26(12):1857-62.	
	Dancer SJ, Coyne M, Speekenbrink A, Samavedam S, Kennedy J, Wallace PG. MRSA acquisition in an intensive care unit. American journal of infection control. 2006 Feb 1;34(1):10-7.	ICU setting
	Grundmann H, Hori S, Winter B, Tami A, Austin DJ. Risk factors for the transmission of methicillin-resistant Staphylococcus aureus in an adult intensive care unit: fitting a model to the data. The Journal of infectious diseases. 2002 Feb 15;185(4):481-8.	ICU setting
	Kong F, Cook D, Paterson DL, Whitby M, Clements AC. Do staffing and workload levels influence the risk of new acquisitions of meticillin-resistant Staphylococcus aureus in a well-resourced intensive care unit?. Journal of Hospital Infection. 2012 Apr 1;80(4):331-9.	ICU setting
	Hugonnet S, Villaveces A, Pittet D. Nurse staffing level and nosocomial infections: empirical evaluation of the case-crossover and case-time-control designs. American journal of epidemiology. 2007 Jun 1;165(11):1321-7.	ICU setting
	Rogowski JA, Staiger D, Patrick T, Horbar J, Kenny M, Lake ET. Nurse staffing and NICU infection rates. JAMA pediatrics. 2013 May 1;167(5):444-50.	ICU setting
	Parikh A, Huang SA, Murthy P, Dombrovskiy V, Nolledo M, Lefton R, Scardella AT. Quality improvement and cost savings after implementation of the Leapfrog intensive care unit physician staffing standard at a community teaching hospital. Critical care medicine. 2012 Oct 1;40(10):2754-9.	ICU setting

For peer review only

	Hugonnet S, Chevrolet JC, Pittet D. The effect of workload on infection risk in critically ill patients. Critical care medicine. 2007 Jan 1;35(1):76-81.	ICU setting
	Maillet JM, Guérot E, Novara A, Le Guen J, Lahjibi-Paulet H, Kac G, Diehl JL, Fagon JY. Comparison of intensive-care-unit-acquired infections and their outcomes among patients over and under 80 years of age. Journal of Hospital Infection. 2014 Jul 1;87(3):152-8.	ICU setting
	Daud-Gallotti RM, Costa SF, Guimarães T, Padilha KG, Inoue EN, Vasconcelos TN, da Silva Cunha Rodrigues F, Barbosa EV, Figueiredo WB, Levin AS. Nursing workload as a risk factor for healthcare associated infections in ICU: a prospective study. PloS one. 2012 Dec 27;7(12):e52342.	ICU setting
	The U. Relationship between probable nosocomial bacteraemia and organisational and structural factors in UK neonatal intensive care units. Quality & safety in health care. 2005 Aug;14(4):264.	ICU setting
	Halwani M, Solaymani-Dodaran M, Grundmann H, Coupland C, Slack RJ. Cross-transmission of nosocomial pathogens in an adult intensive care unit: incidence and risk factors. Journal of Hospital Infection. 2006 May 1;63(1):39-46.	ICU setting
	Dorsey G, Borneo HT, Sun SJ, Wells J, Steele L, Howland K, Perdreau-Remington F, Bangsberg DR. A heterogeneous outbreak of Enterobacter cloacae and Serratia marcescens infections in a surgical intensive care unit. Infection Control & Hospital Epidemiology. 2000 Jul;21(7):465-9.	ICU setting
	Fraher MH, Collins CJ, Bourke J, Phelan D, Lynch M. Cost-effectiveness of employing a total	wrong intervention (Addition of a specialist nurse)

For peer review only

	parenteral nutrition surveillance nurse for the prevention of catheter-related bloodstream infections. Journal of Hospital Infection. 2009 Oct 1;73(2):129-34.	
	Barkell NP, Killinger KA, Schultz SD. The relationship between nurse staffing models and patient outcomes: a descriptive study. Outcomes Management. 2002 Jan 1;6(1):27-33.	wrong intervention (skill mix)
	Hall LM, Doran D, Pink GH. Nurse staffing models, nursing hours, and patient safety outcomes. JONA: The Journal of Nursing Administration. 2004 Jan 1;34(1):41-5.	wrong intervention (skill mix)
	Yang PH, Hung CH, Chen YM, Hu CY, Shieh SL. The impact of different nursing skill mix models on patient outcomes in a respiratory care center. Worldviews on Evidence-Based Nursing. 2012 Nov;9(4):227-33.	wrong intervention (skill mix)
	Virtanen M, Kurvinen T, Terho K, Oksanen T, Peltonen R, Vahtera J, Routamaa M, Elovainio M, Kivimäki M. Work hours, work stress, and collaboration among ward staff in relation to risk of hospital-associated infection among patients. Medical care. 2009 Mar 1:310-8.	wrong intervention (overtime)
	Dimick JB, Pronovost PJ, Heitmiller RF, Lipsett PA. Intensive care unit physician staffing is associated with decreased length of stay, hospital cost, and complications after esophageal resection. Critical care medicine. 2001 Apr 1;29(4):753-8.	wrong intervention (Physician staffing)

For peer review only

For peer review only

Online supplemental file 4: Overlap of primary papers across reviews

Study name	Thungjaroenkul P et al (2007)	Twigg et al (2020)	Assaye et al (2020)	Bourgon et al (2019)	Shin et al (2019)	Wilson et al (2011)	Kane et al (2007)	Griffiths et al (2019)	Lankashear et al (2005)	Engineer et al (2015)	Staplers et al (2015)	Mitchell et al (2018)	Lang et al (2004)	Hill (2017)	Frequency
Aiken LH, Cimiotti JP, Sloane DM, Smith HL, Flynn L, Neff DF. Effects of nurse staffing and nurse education on patient deaths in hospitals with different nurse work environments. The Journal of nursing administration. 2012	No	No	No	Yes	No	No	No	No	No	No	No	No	No	No	1

For peer review only

Oct;42(10 Suppl):S10.															
Aiken LH, Clarke SP, Cheung RB, Sloane DM, Silber JH. Educational levels of hospital nurses and surgical patient mortality. Jama. 2003 Sep 24;290(12): 1617-23.	No	No	No	No	No	No	Yes	No	Yes	No	No	No	Yes	No	3
Aiken LH, Clarke SP, Sloane DM, Lake ET, Cheney T. Effects of hospital care environment on patient mortality and nurse outcomes.	No	No	No	Yes	No	No	No	No	No	No	No	No	No	No	1

For peer review only

The Journal of nursing administration. 2008 May;38(5):223.															
Aiken LH, Clarke SP, Sloane DM, Sochalski J, Silber JH. Hospital nurse staffing and patient mortality, nurse burnout, and job dissatisfaction. Jama. 2002 Oct 23;288(16):1987-93.	No	No	No	Yes	No	No	Yes	No	Yes	No	No	No	Yes	No	4
Aiken LH, Clarke SP, Sloane DM. Hospital restructuring: does it adversely affect care and outcomes?.	No	No	No	No	No	No	No	No	No	No	No	No	Yes	No	1

For peer review only

For peer review only - http://bmjopen.bmj.com/site/about/guidelines.xhtml

JONA: The Journal of Nursing Administration. 2000 Oct 1;30(10):457-65.															
Aiken LH, Sloane DM, Bruyneel L, Van den Heede K, Griffiths P, Busse R, Diomidous M, Kinnunen J, Kózka M, Lesaffre E, McHugh MD. Nurse staffing and education and hospital mortality in nine European countries: a retrospective observational study. The lancet. 2014	No	No	No	Yes	No	No	No	No	No	No	No	No	No	No	1

May 24;383(9931):1824-30.															
Aiken LH, Sloane DM, Lake ET, Sochalski J, Weber AL. Organization and outcomes of inpatient AIDS care. Medical care. 1999 Aug 1:760-72.	No	No	No	No	No	No	Yes	No	No	No	No	No	No	No	1
Al-Kandari F, Thomas D. Factors contributing to nursing task incompletion as perceived by nurses working in Kuwait general hospitals.	No	No	No	No	No	No	No	Yes	No	No	No	No	No	No	1

For peer review only

Journal of clinical nursing. 2009 Dec;18(24): 3430-40.															
American Nurses Association. Implementing nursing's report card: A study of RN staffing, length of stay, and patient outcomes. Amer Nurses Assn; 1997.	No	No	No	No	No	N o	Yes	No	No	No	No	No	No	No	1
American Nurses Association. Nurse staffing and patient outcomes in the inpatient hospital setting. Amer	No	No	No	No	No	N o	No	No	No	No	No	No	Yes	No	1

For peer review only

Nurses Assn; 2000.														
Arbesman MC, Wright C. Mechanical restraints, rehabilitation therapies, and staffing adequacy as risk factors for falls in an elderly hospitalized population. Rehabilitation Nursing. 1999 May 6;24(3):122-8.	No	No	No	No	No	No	No	No	No	No	No	No	Yes	No	1
Ausserhofer, D., Zander, B., Busse, R., Schubert, M., De Geest, S., & Rafferty, A. M., ... Consortium, R. C. (2014). Prevalence,	No	No	No	No	No	No	No	Yes	No	No	No	No	No	No	1

For peer review only

patterns and predictors of nursing care left undone in European hospitals: Results from the multicountry cross-sectional RN4CAST study. BMJ Quality & Safety, 23(2), 126–135. https://doi.org/10.1136/bmjqs2013-002318															
Ball JE, Bruyneel L, Aiken LH, Sermeus W, Sloane DM, Rafferty AM, Lindqvist R, Tishelman C, Griffiths P, RN4Cast Consortium.	No	No	No	Yes	No	No	No	No	No	No	No	No	No	No	1

For peer review only

Post-operative mortality, missed care and nurse staffing in nine countries: a cross-sectional study. International journal of nursing studies. 2018 Feb 1;78:10-5.															
Ball, J. E., Griffiths, P., Rafferty, A. M., Lindqvist, R., Murrells, T., & Tishelman, C. (2016). A cross-sectional study of 'care left undone'on nursing shifts in hospitals.	No	No	No	No	No	No	No	Yes	No	No	No	No	No	No	1

For peer review only

Journal of Advanced Nursing, 72, 2086–2097. https://doi.org/10.1111/jan.12976														
Ball, J. E., Murrells, T., Rafferty, A. M., Morrow, E., & Griffiths, P. (2014). 'Care left undone' during nursing shifts: Associations with workload and perceived quality of care. BMJ Quality & Safety, 23(2), 116–125. https://doi.org/10.1136/bmjqs-	No	No	No	No	No	No	No	Yes	No	No	No	No	No	No	1

For peer review only

2012-001767														
Behner KG, Fogg LF, Fournier LC, Frankenbach JT, Robertson SB. Nursing resource management: analyzing the relationship between costs and quality in staffing decisions. Health Care Management Review. 1990 Jan 1;15(4):63-71.	Yes	No	No	No	No	No	No	No	No	No	No	No	No	No	1
Berney B, Needleman J. Impact of nursing overtime on nurse-sensitive	No	No	No	Yes	No	No	No	No	No	No	No	No	No	No	1

patient outcomes in New York hospitals, 1995-2000. Policy, Politics, & Nursing Practice. 2006 May;7(2):87 -100.															
Blegen MA, Goode CJ, Reed L. Nurse staffing and patient outcomes. Nursing research. 1998 Jan 1;47(1):43-50.	No	No	No	No	No	N o	No	No	No	No	No	No	Yes	No	1
Blegen MA, Goode CJ, Spetz J, Vaughn T, Park SH. Nurse staffing effects on patient outcomes:	No	No	No	Yes	No	N o	No	No	No	No	No	No	No	No	1

For peer review only

safety-net and non-safety-net hospitals. Medical care. 2011 Apr 1:406-14.															
Blegen MA, Vaughn T. A multisite study of nurse staffing and patient occurrences. Nursing Economics. 1998 Jul 1;16(4):196.	No	No	No	No	No	N o	No	No	Yes	No	No	No	Yes	No	2
Bond CA, Raehl CL, Franke T. Medication errors in United States hospitals. Pharmacotherapy: The Journal of Human Pharmacology and Drug	No	No	No	No	No	N o	No	No	Yes	No	No	No	No	No	1

For peer review only

Therapy. 2001 Sep;21(9):1023-36.															
Bond CA, Raehl CL, Pitterle ME, Franke T. Health care professional staffing, hospital characteristics, and hospital mortality rates. Pharmacotherapy: The Journal of Human Pharmacology and Drug Therapy. 1999 Feb;19(2):130-8.	No	No	No	No	No	N o	No	No	Yes	No	No	No	Yes	No	2
Breckenridge-Sproat S, Johantgen M, Patrician P. Influence of unit-level staffing on	No	No	No	No	No	N o	No	No	No	No	Yes	No	No	No	1

For peer review only

medication errors and falls in military hospitals. Western journal of nursing research. 2012 Jun;34(4):455-74.														
Brooks Carthon JM, Kutney-Lee A, Jarrín O, Sloane D, Aiken LH. Nurse staffing and postsurgical outcomes in black adults. Journal of the American Geriatrics Society. 2012 Jun;60(6):1078-84.	No	No	No	Yes	No	No	No	No	No	No	No	No	No	No	1

For peer review only

Bruyneel, L., Li, B., Ausserhofer, D., Lesaffre, E., Dumitrescu, I., Smith, H. L., & Sermeus, W. (2015). Organization of hospital nursing, provision of nursing care and patient experiences with care in Europe. Medical Care Research and Review, 72(6), 643–664. https://doi.org/10.1177/1077558715589188	No	No	No	No	No	No	No	Yes	No	No	No	No	No	No	1

For peer review only

For peer review only - http://bmjopen.bmj.com/site/about/guidelines.xhtml

Burnes Bolton L, Aydin CE, Donaldson N, Storer Brown D, Sandhu M, Fridman M, Udin Aronow H. Mandated nurse staffing ratios in California: a comparison of staffing and nursing-sensitive outcomes pre-and postregulation. Policy, Politics, & Nursing Practice. 2007 Nov;8(4):238-50.	No	Yes	No	No	No	No	No	No	No	No	Yes	No	No	No	2
Carter JH, Mills AC, Homan SM, Blaesing SL, Heater BS,													Yes	No	1

For peer review only

Stoll LD, Mornin C, Corrigan MK. Correlating the quality of care with nursing resources and patient parameters: a longitudinal study. NLN publications . 1987 Dec(20-2191):331-45.														
Cheung RB. The relationship between nurse staffing, nursing time, and adverse events in an acute care hospital. University of South	No	No	No	No	No	N o	Yes	No	No	No	No	No	No	No	1

For peer review only

Florida; 2002.															
Cho E, Lee NJ, Kim EY, Kim S, Lee K, Park KO, Sung YH. Nurse staffing level and overtime associated with patient safety, quality of care, and care left undone in hospitals: a cross-sectional study. International journal of nursing studies. 2016 Aug 1;60:263-71.	No	No	No	No	No	N o	No	Yes	No	No	No	No	No	No	1

For peer review only

Cho E, Sloane DM, Kim EY, Kim S, Choi M, Yoo IY, Lee HS, Aiken LH. Effects of nurse staffing, work environments, and education on patient mortality: an observational study. International journal of nursing studies. 2015 Feb 1;52(2):535-42.	No	No	No	Yes	No	No	No	No	No	No	No	No	No	No	1
Cho SH, Ketefian S, Barkauskas VH, Smith DG. The effects of nurse staffing on adverse	Yes	No	No	No	No	No	Yes	No	Yes	No	No	No	Yes	No	4

For peer review only

events, morbidity, mortality, and medical costs. Nursing research. 2003 Mar 1;52(2):71-9.															
Cho SH, Kim YS, Yeon KN, You SJ, Lee ID. Effects of increasing nurse staffing on missed nursing care. International nursing review. 2015 Jun;62(2):267-74.	No	No	No	No	No	No	No	Yes	No	No	No	No	No	No	1
Christine K, Cheryl J, Chunliu Z, Peter JG, Jayasree B. Nurse staffing and postsurgical	No	No	No	No	No	No	No	No	Yes	No	No	Yes	Yes	No	3

For peer review only

adverse events: an analysis of administrative data from a sample of US hospitals, 1990–1996. Health services research. 2002 Jun;37(3):611-29.															
Cimiotti JP, Aiken LH, Sloane DM, Wu ES. Nurse staffing, burnout, and health care–associated infection. American journal of infection control. 2012 Aug 1;40(6):486-90.	No	No	No	No	No	No	No	No	No	No	No	Yes	No	No	1

For peer review only

Cook A, Gaynor M, Stephens Jr M, Taylor L. The effect of a hospital nurse staffing mandate on patient health outcomes: Evidence from California's minimum staffing regulation. Journal of Health Economics. 2012 Mar 1;31(2):340-8.	No	Yes	No	No	No	No	No	No	No	No	No	No	No	No	1
Dabney BW, Kalisch BJ. Nurse staffing levels and patient-reported missed nursing care. Journal	No	No	No	No	No	No	No	Yes	No	No	No	No	No	No	1

For peer review only

of nursing care quality. 2015 Oct 1;30(4):306-12.															
Dobal MT. The relationships among the context, structure, and performance of nursing units in hospitals (Doctoral dissertation, The University of Texas at Austin).	No	No	No	No	No	N o	No	No	No	No	No	No	Yes	No	1
Donaldson N, Bolton LB, Aydin C, Brown D, Elashoff JD, Sandhu M. Impact of California's licensed nurse-patient	No	Yes	No	No	No	N o	No	No	No	No	Yes	No	No	No	2

For peer review only

ratios on unit-level nurse staffing and patient outcomes. Policy, Politics, & Nursing Practice. 2005 Aug;6(3):198-210.															
Dunton N, Gajewski B, Taunton RL, Moore J. Nurse staffing and patient falls on acute care hospital units. Nursing outlook. 2004 Feb 1;52(1):53-9.	No	No	No	No	Yes	No	No	No	No	No	No	No	No	No	1
Elkassabany NM, Passarella M, Mehta S, Liu J,	No	No	No	Yes	No	No	No	No	No	No	No	No	No	No	1

For peer review only

Neuman MD. Hospital characteristics, inpatient processes of care, and readmissions of older adults with hip fractures. Journal of the American Geriatrics Society. 2016 Aug;64(8):1656-61.															
Elting LS, Pettaway C, Bekele BN, Grossman HB, Cooksley C, Avritscher EB, Saldin K, Dinney CP. Correlation between annual volume of cystectomy,	No	No	No	No	No	No	Yes	No	No	No	No	No	No	No	1

For peer review only

professional staffing, and outcomes: a statewide, population-based study. Cancer: Interdisciplinary International Journal of the American Cancer Society. 2005 Sep 1;104(5):975-84.															
Everhart, D. M., Schumacher, J. R., Duncan, R. P., Hall, A. G., Neff, D. F., & Shorr, R. I. (2014). Determinants of hospital fall rate trajectory groups: A longitudinal assessment	No	No	No	No	Yes	N o	No	No	No	No	No	No	No	No	1

For peer review only

of nurse staffing and organiza-tional characteristics. Health Care Management Review, 39(4), 352–360														
Feleke SA, Mulatu MA, Yesmaw YS. Medication administration error: magnitude and associated factors among nurses in Ethiopia. BMC nursing. 2015 Dec;14(1):1-8.	No	No	Yes	No	No	No	No	No	No	No	No	No	No	No	1
Flood SD, Diers D. Nurse staffing, patient	No	No	No	No	No	No	No	No	No	No	No	No	Yes	No	1

For peer review only

outcome and cost. Nursing Management. 1988 May 1;19(5):34-45.															
Friese CR, Kalisch BJ, Lee KH. Patterns and correlates of missed nursing care in inpatient oncology units. Cancer nursing. 2013 Nov;36(6):E51.	No	No	No	No	No	No	No	Yes	No	No	No	No	No	No	1
Friese CR, Lake ET, Aiken LH, Silber JH, Sochalski J. Hospital nurse practice environments and outcomes for surgical	No	No	No	Yes	No	No	No	No	No	No	No	No	No	No	1

For peer review only

oncology patients. Health services research. 2008 Aug;43(4):1 145-63.															
Frith KH, Anderson EF, Caspers B, Tseng F, Sanford K, Hoyt NG, Moore K. Effects of nurse staffing on hospital-acquired conditions and length of stay in community hospitals. Quality Management in Healthcare. 2010 Apr 1;19(2):147-55.	No	No	No	No	Yes	No	No	No	No	No	Yes	No	No	No	2

For peer review only

Ghaferi AA, Osborne NH, Birkmeyer JD, Dimick JB. Hospital characteristics associated with failure to rescue from complications after pancreatectomy. Journal of the American College of Surgeons. 2010 Sep 1;211(3):325-30.	No	No	No	Yes	No	No	No	No	No	No	No	No	No	No	1
Glance LG, Dick AW, Osler TM, Mukamel DB, Li Y, Stone PW. The association between nurse	No	No	No	No	No	No	No	No	No	No	No	Yes	No	No	1

For peer review only

staffing and hospital outcomes in injured patients. BMC health services research. 2012 Dec;12(1):1-8														
Goode CJ, Blegen MA, Park SH, Vaughn T, Spetz J. Comparison of patient outcomes in Magnet® and non-Magnet hospitals. JONA: The Journal of Nursing Administration. 2011 Dec 1;41(12):517-23.	No	No	No	No	No	No	No	No	No	No	Yes	No	No	No	1

Griffiths P, Dall'Ora C, Simon M, Ball J, Lindqvist R, Rafferty AM, Schoonhoven L, Tishelman C, Aiken LH. Nurses' shift length and overtime working in 12 European countries: the association with perceived quality of care and patient safety. Medical care. 2014 Nov;52(11): 975.	No	No	No	No	No	No	No	Yes	No	No	No	No	No	No	1
Griffiths P, Jones S, Bottle A. Is "failure to rescue" derived	No	No	No	Yes	No	No	No	No	No	No	No	No	No	No	1

For peer review only

from administrative data in England a nurse sensitive patient safety indicator for surgical care? Observational study. International Journal of Nursing Studies. 2013 Feb 1;50(2):292-300.															
Gunningberg L, Donaldson N, Aydin C, Idvall E. Exploring variation in pressure ulcer prevalence in Sweden and the USA: benchmarki	No	No	No	No	No	No	No	No	No	No	Yes	No	No	No	1

For peer review only

ng in action. Journal of evaluation in clinical practice. 2012 Aug;18(4):904-10.															
Haley RW, Bregman DA. The role of understaffing and overcrowding in recurrent outbreaks of staphylococcal infection in a neonatal special-care unit. Journal of Infectious Diseases. 1982 Jun 1;145(6):875-85.	No	No	No	No	No	No	No	No	No	No	No	No	Yes	No	1
Halm M, Peterson M, Kandels M, Sabo J, Blalock M,	No	No	No	Yes	No	No	Yes	No	No	No	No	No	No	No	2

For peer review only

Braden R, Gryczman A, Krisko-Hagel K, Larson D, Lemay D, Sisler B. Hospital nurse staffing and patient mortality, emotional exhaustion, and job dissatisfacti on. Clinical Nurse Specialist. 2005 Sep 1;19(5):241-51.															
Halm M, Peterson M, Kandels M, Sabo J, Blalock M, Braden R, Gryczman A, Krisko-Hagel K, Larson D, Lemay D, Sisler B. Hospital nurse	No	No	No	No	No	N o	No	No	No	No	No	No	No	Yes	1

For peer review only

staffing and patient mortality, emotional exhaustion, and job dissatisfaction. Clinical Nurse Specialist. 2005 Sep 1;19(5):241-51.															
Harless DW, Mark BA. Nurse staffing and quality of care with direct measurement of inpatient staffing. Medical care. 2010 Jul 1:659-63.	No	No	No	Yes	No	No	No	No	No	No	No	No	No	No	1
Hartz AJ, Krakauer H, Kuhn EM, Young M, Jacobsen SJ, Gay G, Muenz L,	No	No	No	No	No	No	No	No	No	No	No	No	Yes	No	1

For peer review only

Katzoff M, Bailey RC, Rimm AA. Hospital characteristics and mortality rates. New England journal of medicine. 1989 Dec 21;321(25): 1720-5.															
Hickey, P. A., Gauvreau, K., Jenkins, K., Fawcett, J., & Hayman, L. (2011). Statewide and national impact of California's staffing law on pediatric cardiac surgery outcomes. JONA: the Journal of Nursing	No	Yes	No	No	No	No	No	No	No	No	No	No	No	No	1

Administration, 41(5), 218–225.															
Hinno S, Partanen P, Vehviläinen-Julkunen K. Nursing activities, nurse staffing and adverse patient outcomes as perceived by hospital nurses. Journal of clinical nursing. 2012 Jun;21(11-12):1584-93.	No	No	No	No	Yes	N o	No	No	No	No	No	No	No	No	1
Hope J. Nosocomial Infections and Their Relationship to Nursing Workload in an Acute Care	No	No	No	No	No	N o	Yes	No	No	No	No	No	No	No	1

For peer review only

Hospital dissertation. Ottawa, Ontario, Canada: Queen's University. 2003.															
Houser EP. Nurse staffing levels and patient outcomes. The Johns Hopkins University; 2005.	No	No	No	No	No	N o	Yes	No	No	No	No	No	No	No	1
Jiang HJ, Stocks C, Wong CJ. Disparities between two common data sources on hospital nurse staffing. Journal of Nursing Scholarship. 2006	No	No	No	No	No	N o	No	No	No	No	Yes	No	No	No	1

For peer review only

Jun;38(2):187-93.															
Kalisch BJ, Doumit M, Lee KH, El Zein J. Missed nursing care, level of staffing, and job satisfaction: Lebanon versus the United States. JONA: The Journal of Nursing Administration. 2013 May 1;43(5):274-9.	No	No	No	No	No	No	No	Yes	No	No	No	No	No	No	1
Kalisch BJ, Tschannen D, Lee H, Friese CR. Hospital variation in missed nursing	No	No	No	No	No	No	No	Yes	No	No	No	No	No	No	1

For peer review only

care. American Journal of Medical Quality. 2011 Jul;26(4):291-9.															
Kalisch BJ, Tschannen D, Lee KH. Do staffing levels predict missed nursing care?. International Journal for Quality in Health Care. 2011 Jun 1;23(3):302-8.	No	No	No	No	No	No	No	Yes	No	No	No	No	No	No	1
Kim CG, Bae KS. Relationship between nurse staffing level and adult nursing-sensitive outcomes in	No	No	No	No	Yes	No	No	No	No	No	No	No	No	No	1

For peer review only

tertiary hospitals of Korea: Retrospective observational study. International Journal of Nursing Studies. 2018 Apr 1;80:155-64.														
Kim Y, Han K. Longitudinal associations of nursing staff turnover with patient outcomes in long-term care hospitals in Korea. Journal of Nursing Management. 2018 Jul;26(5):518-24.	No	No	No	No	Yes	No	No	No	No	No	No	No	No	No	1

Kouatly IA, Nassar N, Nizam M, Badr LK. Evidence on nurse staffing ratios and patient outcomes in a low-income country: Implications for future research and practice. Worldviews on Evidence-Based Nursing. 2018 Oct;15(5):353-60.	No	No	Yes	No	Yes	No	No	No	No	No	No	No	No	No	2
Kovner C, Gergen PJ. Nurse staffing levels and adverse events following	No	No	No	No	No	No	No	No	Yes	No	No	No	Yes	No	2

For peer review only

surgery in US hospitals. Image: The Journal of Nursing Scholarship. 1998 Dec;30(4):315-21.														
Kutney-Lee A, Aiken LH. Effect of nurse staffing and education on the outcomes of surgical patients with comorbid serious mental illness. Psychiatric Services. 2008 Dec;59(12):1466-9.	No	No	No	Yes	No	No	No	No	No	No	No	No	No	No	1

For peer review only

Li X, Bowman SM, Smith TC. Effects of registered nurse staffing level on hospital-acquired conditions in cardiac surgery patients: a propensity score matching analysis. Nursing outlook. 2016 Nov 1;64(6):533-41.	No	No	No	Yes	No	No	No	No	No	No	No	No	No	No	1
Lichtig LK, Knauf RA, Milholland DK. Some impacts of nursing on acute care hospital outcomes. JONA: The Journal of	Yes	No	No	No	No	No	Yes	No	Yes	No	No	No	No	No	3

For peer review only

Nursing Administration. 1999 Feb 1;29(2):25-33.															
Magalhães AM, Dall'Agnol CM, Marck PB. Nursing workload and patient safety-a mixed method study with an ecological restorative approach. Revista latino-americana de enfermagem. 2013;21:146-54.	No	No	Yes	No	No	No	No	No	No	No	No	No	No	No	1
Manheim LM, Feinglass J, Shortell SM, Hughes EF.	No	No	No	No	No	No	No	No	Yes	No	No	No	Yes	No	2

For peer review only

Regional variation in Medicare hospital mortality. Inquiry. 1992 Apr 1:55-66.															
Manojlovich M, Sidani S, Covell CL, Antonakos CL. Nurse dose: linking staffing variables to adverse patient outcomes. Nursing research. 2011 Jul 1;60(4):214-20.	No	No	No	No	No	N o	No	No	No	No	No	Yes	No	No	1
Mark BA, Harless DW, Berman WF. Nurse staffing and adverse events in hospitalized children. Policy,	No	No	No	Yes	No	Y es	No	No	No	No	No	Yes	No	No	3

For peer review only

Politics, & Nursing Practice. 2007 May;8(2):83-92.														
Mark BA, Harless DW, McCue M, Xu Y. A longitudinal examination of hospital registered nurse staffing and quality of care. Health services research. 2004 Apr;39(2):279-300.	No	No	No	No	Yes	No	Yes	No	Yes	No	Yes	Yes	No	No	5
Mark BA, Harless DW, McCue M. The impact of HMO penetration on the relationship between nurse staffing and	No	No	No	No	No	Yes	No	No	No	No	No	No	No	No	1

For peer review only

quality. Health economics. 2005 Jul;14(7):737-53.															
Mark BA, Harless DW, Spetz J, Reiter KL, Pink GH. California's minimum nurse staffing legislation: results from a natural experiment. Health services research. 2013 Apr;48(2pt1):435-54.	No	Yes	No	No	No	No	No	No	No	No	No	No	No	No	1
Mark BA, Harless DW. Nurse staffing and post-surgical complications using the present on admission	No	No	No	No	Yes	No	No	No	No	No	No	Yes	No	No	2

For peer review only

indicator. Research in nursing & health. 2010 Feb;33(1):35-47.															
McCloskey BA, Diers DK. Effects of New Zealand's health reengineering on nursing and patient outcomes. Medical care. 2005 Nov 1:1140-6.	No	No	No	Yes	No	No	No	No	No	No	Yes	No	No	No	2
McHugh MD, Rochman MF, Sloane DM, Berg RA, Mancini ME, Nadkarni VM, Merchant RM, Aiken LH, American	No	No	No	No	No	No	No	No	No	No	No	No	No	Yes	1

For peer review only

Heart Association's Get With The Guidelines-Resuscitation Investigators. Better nurse staffing and nurse work environments associated with increased survival of in-hospital cardiac arrest patients. Medical care. 2016 Jan;54(1):74.															
Morita K, Matsui H, Fushimi K, Yasunaga H. Association between nurse staffing and	No	No	No	No	Yes	No	No	No	No	No	No	No	No	No	1

For peer review only

in-hospital bone fractures: a retrospective cohort study. Health services research. 2017 Jun;52(3):1005-23.															
Needleman J, Buerhaus P, Mattke S, Stewart M, Zelevinsky K. Nurse-staffing levels and the quality of care in hospitals. New England Journal of Medicine. 2002 May 30;346(22):1715-22.	Yes	No	No	Yes	No	No	Yes	No	Yes	No	No	Yes	No	Yes	6
Needleman J, Buerhaus PI, Mattke S, Stewart M,	No	No	No	No	No	No	No	No	No	No	No	Yes	No	No	1

For peer review only

Zelevinsky K. Measuring Hospital Quality: Can Medicare Data Substitute for All-Payer Data?. Health services research. 2003 Dec;38(6p1):1487-508.															
Needleman J, Buerhaus PI, Mattke S, Stewart M, Zelevinsky K. Nurse staffing and patient outcomes in hospitals. Final report. Rockville, MD: Health Resources and Services Administration. 2001 Feb 28.	No	No	No	No	No	No	No	No	No	No	No	Yes	No	No	1

For peer review only

Neff DF, Cimiotti J, Sloane DM, Aiken LH. Utilization of non-US educated nurses in US hospitals: implications for hospital mortality. International Journal for Quality in Health Care. 2013 Sep 1;25(4):366-72.	No	No	No	Yes	No	No	No	No	No	No	No	No	No	No	1
Orique SB, Patty CM, Woods E. Missed nursing care and unit-level nurse workload in the acute and post-acute settings. Journal of nursing care quality.	No	No	No	No	No	No	No	Yes	No	No	No	No	No	No	1

For peer review only

2016 Jan 1;31(1):84-9.															
Palese A, Ambrosi E, Prosperi L, Guarnier A, Barelli P, Zambiasi P, Allegrini E, Bazoli L, Casson P, Marin M, Padovan M. Missed nursing care and predicting factors in the Italian medical care setting. Internal and emergency medicine. 2015 Sep;10(6):693-702.	No	No	No	No	No	No	No	Yes	No	No	No	No	No	No	1

For peer review only

Pappas S, Davidson N, Woodard J, Davis J, Welton JM. Risk-adjusted staffing to improve patient value. Nursing Economics. 2015 Mar 1;33(2):73.	No	No	No	No	No	No	No	No	No	No	No	Yes	No	No	1
Patrician PA, Loan L, McCarthy M, Fridman M, Donaldson N, Bingham M, Brosch LR. The association of shift-level nurse staffing with adverse patient events. JONA: the Journal of Nursing	No	No	No	No	No	No	No	No	No	No	Yes	No	No	No	1

For peer review only

Administration. 2011 Feb 1;41(2):64-70.															
Patrician PA, McCarthy MS, Swiger P, Raju D, Breckenridge-Sproat S, Su X, Randall KH, Loan LA. Association of temporal variations in staffing with hospital-acquired pressure injury in military hospitals. Research in nursing & health. 2017 Apr;40(2):111-9.	No	No	No	No	Yes	No	No	No	No	No	No	No	No	No	1
Person SD, Allison JJ, Kiefe CI, Weaver MT, Williams	No	No	No	No	No	No	Yes	No	Yes	No	No	No	No	No	2

For peer review only

OD, Centor RM, Weissman NW. Nurse staffing and mortality for Medicare patients with acute myocardial infarction. Medical care. 2004 Jan 1:4-12.															
Prot S, Fontan JE, Alberti C, Bourdon O, Farnoux C, Macher MA, Foureau A, Faye A, Beaufils F, Gottot S, Brion F. Drug administration errors and their determinants in pediatric in-patients. International Journal for	No	No	No	No	No	Yes	No	No	No	No	No	No	No	No	1

For peer review only

Quality in Health Care. 2005 Oct 1;17(5):381-9.														
Rafferty AM, Clarke SP, Coles J, Ball J, James P, McKee M, Aiken LH. Outcomes of variation in hospital nurse staffing in English hospitals: cross-sectional analysis of survey data and discharge records. International journal of nursing studies. 2007 Feb 1;44(2):175-82.	No	No	No	Yes	No	No	No	No	No	No	No	No	No	Yes	2

For peer review only

Razmus I. Factors associated with pediatric hospital-acquired pressure injuries. Journal of Wound, Ostomy and Continence Nursing. 2018 Mar 1;45(2):107-16.	No	No	No	No	Yes	No	No	No	No	No	No	No	No	No	1
Richet HM, Benbachir M, Brown DF, Giamarellou H, Gould I, Gubina M, Heczko P, Kalenic S, Pana M, Pittet D, Redjeb SB. Are there regional variations in the diagnosis,	No	No	No	No	No	No	No	No	No	No	No	Yes	No	No	1

For peer review only

surveillance, and control of methicillin-resistant Staphylococcus aureus?. Infection Control & Hospital Epidemiology. 2003 May;24(5):334-41.															
Robertson RH, Hassan M. Staffing intensity, skill mix and mortality outcomes: the case of chronic obstructive lung disease. Health Services Management Research. 1999 Nov;12(4):258-68.	No	No	No	No	No	No	Yes	No	No	No	No	No	No	No	1

For peer review only

Robertson RH, Hassan M. Staffing intensity, skill mix and mortality outcomes: the case of chronic obstructive lung disease. Health Services Management Research. 1999 Nov;12(4):258-68.	No	No	No	No	No	No	No	No	No	No	No	No	Yes	No	1
Sasichay-Akkadechanunt T, Scalzi CC, Jawad AF. The relationship between nurse staffing and patient outcomes. JONA: The Journal of Nursing Administrati	No	No	Yes	No	No	No	No	No	No	No	No	No	No	No	1

For peer review only

on. 2003 Sep 1;33(9):478-85.															
Schreuders LW, Bremner AP, Geelhoed E, Finn J. The relationship between nurse staffing and inpatient complications. Journal of advanced nursing. 2015 Apr;71(4):800-12.	No	No	No	Yes	No	No	No	No	No	No	No	No	No	No	1
Schubert M, Ausserhofer D, Desmedt M, Schwendimann R, Lesaffre E, Li B, De Geest S. Levels and correlates of implicit rationing of	No	No	No	No	No	No	No	Yes	No	No	No	No	No	No	1

For peer review only

nursing care in Swiss acute care hospitals—a cross sectional study. International journal of nursing studies. 2013 Feb 1;50(2):230-9.															
Schultz MA, van Servellen G, Chang BL, McNeese-Smith D, Waxenberg E. The relationship of hospital structural and financial characteristics to mortality and length of stay in acute myocardial infarction	Yes	No	No	No	No	No	No	No	No	No	No	No	No	No	1

patients. Outcomes Management for Nursing Practice. 1998 Jul 1;2(3):130-6.															
Seago JA, Williamson A, Atwood C. Longitudinal analyses of nurse staffing and patient outcomes: more about failure to rescue. JONA: The Journal of Nursing Administration. 2006 Jan 1;36(1):13-21.	No	No	No	No	No	No	No	No	No	No	Yes	No	No	No	1
Shamian J, Hagen B, Hu TW, Fogarty TE. The relationship between	No	No	No	No	No	No	No	No	No	No	No	No	Yes	No	1

For peer review only

length of stay and required nursing care hours. The Journal of nursing administration. 1994 Jul 1;24(7-8):52-8.															
Shuldham C, Parkin C, Firouzi A, Roughton M, Lau-Walker M. The relationship between nurse staffing and patient outcomes: a case study. International journal of nursing studies. 2009 Jul 1;46(7):986-92.	No	No	No	No	Yes	No	No	No	No	No	Yes	No	No	Yes	3

For peer review only

Silber JH, Kennedy SK, Even-Shoshan O, Chen W, Koziol LF, Showan AM, Longnecker DE. Anesthesiologist direction and patient outcomes. The Journal of the American Society of Anesthesiologists. 2000 Jul 1;93(1):152-63.	No	No	No	No	No	No	Yes	No	No	No	No	No	No	No	1
Silber JH, Kennedy SK, Even-Shoshan O, Chen W, Koziol LF, Showan AM, Longnecker DE. Anesthesiologist	No	No	No	No	No	No	No	No	Yes	No	No	No	No	No	1

For peer review only

direction and patient outcomes. The Journal of the American Society of Anesthesiologists. 2000 Jul 1;93(1):152-63.															
Silber JH, Rosenbaum PR, Ross RN. Comparing the contributions of groups of predictors: which outcomes vary with hospital rather than patient characteristics?. Journal of the American Statistical Association. 1995 Mar	No	No	No	No	No	No	No	No	Yes	No	No	No	No	No	1

For peer review only

1;90(429):7-18.															
Silber JH, Rosenbaum PR, Schwartz JS, Ross RN, Williams SV. Evaluation of the complication rate as a measure of quality of care in coronary artery bypass graft surgery. Jama. 1995 Jul 26;274(4):317-23.	No	No	No	No	No	No	No	No	No	No	No	No	Yes	No	1
Sochalski J, Konetzka RT, Zhu J, Volpp K. Will mandated minimum nurse staffing	No	No	No	Yes	No	No	No	No	No	Yes	No	No	No	No	2

For peer review only

ratios lead to better patient outcomes?. Medical care. 2008 Jun 1:606-13.															
Sovie M. Hospital Restructuring's Impact on Outcomes: Report to Participating Hospitals.	No	No	No	No	No	No	No	No	No	No	No	No	Yes	No	1
Sovie MD, Jawad AF. Hospital restructuring and its impact on outcomes: nursing staff regulations are premature. JONA: The Journal of Nursing Administration. 2001 Dec	No	No	No	No	No	No	No	No	Yes	No	No	No	No	No	1

For peer review only

1;31(12):588-600.															
Spetz J, Harless DW, Herrera CN, Mark BA. Using minimum nurse staffing regulations to measure the relationship between nursing and hospital quality of care. Medical Care Research and Review. 2013 Aug;70(4):380-99.	No	Yes	No	No	No	No	No	No	No	No	No	No	No	No	1
Staggs VS, Knight JE, Dunton N. Understanding unassisted	No	No	No	No	Yes	No	No	No	No	No	No	No	No	No	1

For peer review only

falls: effects of nurse staffing level and nursing staff characteristics. Journal of Nursing Care Quality. 2012 Jul 1;27(3):194-9.														
Stegenga J, Bell E, Matlow A. The role of nurse understaffing in nosocomial viral gastrointestinal infections on a general pediatrics ward. Infection Control & Hospital Epidemiology. 2002	No	No	No	No	No	Yes	No	No	No	No	No	Yes	No	No	2

For peer review only

Mar;23(3):1 33-6.															
Stratton KM. Pediatric nurse staffing and quality of care in the hospital setting. Journal of nursing care quality. 2008 Apr 1;23(2):105-14.	No	No	No	No	No	Y es	No	No	No	No	No	Yes	No	No	2
Sujijantarara t R, Booth RZ, Davis LL. Nosocomial urinary tract infection: nursing-sensitive quality indicator in a Thai hospital. Journal of nursing care quality.	No	No	No	No	No	N o	No	No	No	No	No	Yes	No	No	1

For peer review only

2005 Apr 1;20(2):134-9.															
Sujijantararat R. Nursing-sensitive quality indicators: Nosocomial urinary tract infection in a Thai hospital. The University of Alabama at Birmingham; 2001.	No	No	Yes	No	No	No	No	No	No	No	No	No	No	No	1
Taunton RL, Kleinbeck SV, Stafford R, Woods CQ, Bott MJ. Patient outcomes. Are they linked to registered nurse absenteeism, separation, or work load?. The	No	No	No	No	No	No	No	No	No	No	No	No	Yes	No	1

For peer review only

Journal of nursing administration. 1994 Apr 1;24(4 Suppl):48-55.														
Taylor JA, Dominici F, Agnew J, Gerwin D, Morlock L, Miller MR. Do nurse and patient injuries share common antecedents? An analysis of associations with safety climate and working conditions. BMJ quality & safety. 2012 Feb 1;21(2):101-11.	No	No	No	No	No	No	No	No	No	No	Yes	No	No	No	1

For peer review only

Titler MG, Shever LL, Kanak MF, Picone DM, Qin R. Factors associated with falls during hospitalization in an older adult population. Research and Theory for Nursing Practice. 2011 Jan 1;25(2):127-52.	No	No	No	No	No	No	No	No	No	No	Yes	No	No	No	1
Tourangeau AE, Giovannetti P, Tu JV, Wood M. Nursing-related determinants of 30-day mortality for hospitalized patients. Canadian Journal of	No	No	No	No	No	No	No	No	Yes	No	No	No	No	No	1

Nursing Research Archive. 2002.															
Trinkoff AM, Johantgen M, Storr CL, Gurses AP, Liang Y, Han K. Nurses' work schedule characteristics, nurse staffing, and patient mortality. Nursing research. 2011 Jan 1;60(1):1-8.	No	No	No	No	No	N o	No	No	No	Yes	No	No	No	No	1
Twigg D, Duffield C, Bremner A, Rapley P, Finn J. The impact of the nursing hours per patient day (NHPPD) staffing method on patient	No	Yes	No	Yes	No	N o	No	No	No	No	Yes	Yes	No	No	4

For peer review only

outcomes: a retrospective analysis of patient and staffing data. International journal of nursing studies. 2011 May 1;48(5):540-8.															
Unruh L. Licensed nurse staffing and adverse events in hospitals. Medical care. 2003 Jan 1:142-52.	No	No	No	No	Yes	No	No	No	Yes	No	No	Yes	Yes	No	4
Unruh LY, Zhang NJ. Nurse staffing and patient safety in hospitals: new variable and longitudinal	No	No	No	No	No	No	No	No	No	No	Yes	Yes	No	No	2

For peer review only

approaches. Nursing research. 2012 Jan 1;61(1):3-12.														
Van den Heede K, Lesaffre E, Diya L, Vleugels A, Clarke SP, Aiken LH, Sermeus W. The relationship between inpatient cardiac surgery mortality and nurse numbers and educational level: analysis of administrative data. International journal of nursing studies. 2009 Jun	No	No	No	Yes	No	No	No	No	No	No	No	No	No	No	1

For peer review only

1;46(6):796-803.															
Van den Heede K, Sermeus W, Diya L, Clarke SP, Lesaffre E, Vleugels A, Aiken LH. Nurse staffing and patient outcomes in Belgian acute hospitals: cross-sectional analysis of administrati ve data. Internationa l journal of nursing studies. 2009 Jul 1;46(7):928-39.	No	No	No	Yes	No	N o	No	No	No	No	Yes	No	No	No	2
Van den Heede, K., Sermeus, W., Diya, L.,	No	No	No	No	Yes	N o	No	No	No	No	No	No	No	No	1

For peer review only

Clarke, S. P., Lesaffre, E., Vleugels, A., & Aiken, L. H. (2009). Nurse staffing and patient out-comes in Belgian acute hospitals: Cross-sectional analysis of administrati ve data.															
Wan TT, Shukla RK. Contextual and organization al correlates of the quality of hospital nursing care. QRB-Quality Review Bulletin. 1987 Feb 1;13(2):61-4.	No	No	No	No	No	N o	No	No	No	No	No	No	Yes	No	1

For peer review only

Weiss ME, Yakusheva O, Bobay KL. Quality and cost analysis of nurse staffing, discharge preparation, and postdischarge utilization. Health services research. 2011 Oct;46(5):1473-94.	No	No	No	No	No	No	No	Yes	No	No	No	No	No	No	1
Whitman GR, Kim Y, Davidson LJ, Wolf GA, Wang SL. The impact of staffing on patient outcomes across specialty units. JONA: the Journal of Nursing Administration. 2002	No	No	No	No	No	No	No	No	Yes	No	Yes	No	No	No	2

For peer review only

Dec 1;32(12):633-9.															
Wiltse Nicely KL, Sloane DM, Aiken LH. Lower mortality for abdominal aortic aneurysm repair in high-volume hospitals is contingent upon nurse staffing. Health services research. 2013 Jun;48(3):972-91.	No	No	No	Yes	No	No	No	No	No	No	No	No	No	No	1
Yang KP. Relationships between nurse staffing and patient outcomes. Journal of Nursing	No	No	No	No	No	No	No	No	No	No	No	Yes	No	No	1

For peer review only

Research. 2003 Sep 1;11(3):149-58.															
Yasunaga H, Hashimoto H, Horiguchi H, Miyata H, Matsuda S. Variation in cancer surgical outcomes associated with physician and nurse staffing: a retrospective observational study using the Japanese Diagnosis Procedure Combination Database. BMC health services research. 2012 Dec;12(1):1-7.	No	No	No	Yes	No	No	No	No	No	No	No	No	No	No	1

For peer review only

Yu D, Ma Y, Sun Q, Lu G, Xu P. A nursing care classification system for assessing workload and determining optimal nurse staffing in a teaching hospital in China: A pre-post intervention study. International journal of nursing practice. 2015 Aug;21(4):339-49.	No	No	Yes	No	No	No	No	No	No	No	No	No	No	No	1
Zhu, X.-W., You, L.-M., Zheng, J., Liu, K. E., Fang, J.-B., Hou, S.-X., … Zhang, L.-F. (2012).	No	No	No	No	Yes	No	No	No	No	No	No	No	No	No	1

For peer review only

Nurse staffing levels make a difference on patient outcomes: A multisite study in Chinese hospitals. Journal of Nursing Scholarship, 44(3), 266–273.															

For peer review only

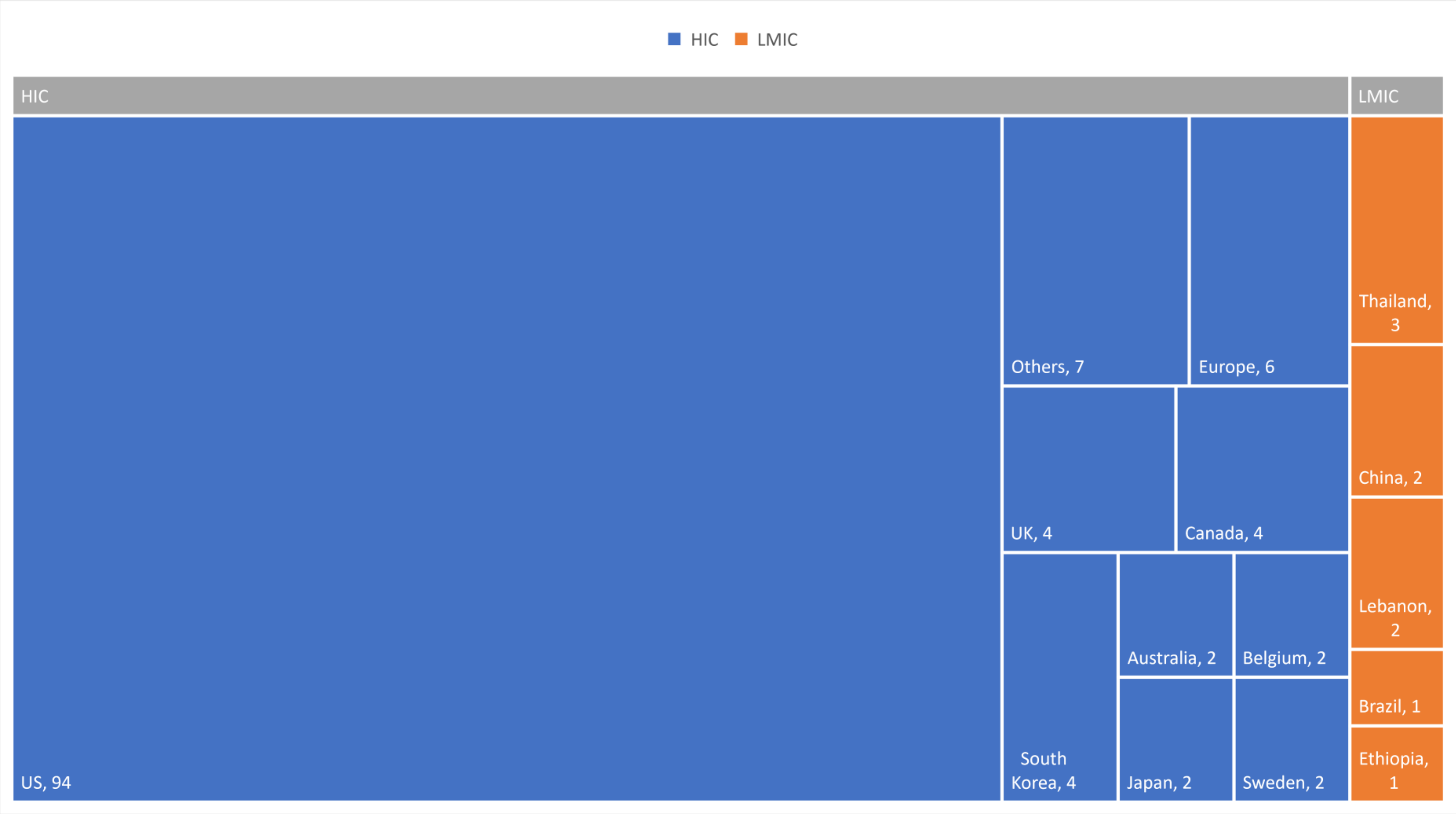

Online supplementary material 5 : Tree Map showing countries primary studies were conducted (n = 136).

Others group are HIC countries that had only 1 study – Kuwait, Taiwan, France, Finland + Netherlands (Dual country study), Multi-country, Italy, New Zealand.

*Studies conducted in Europe were conducted in multiple European countries. Two studies conducted in Lebanon and Sweden were also conducted in the US

For peer review only

Online supplemental material 6 – Summary of nursing metrics reported across papers

Nursing Metrics	Various definitions of individual metrics	Number of studies reporting a particular metric	Total number of studies reporting metrics
nursing hour per patient day	Total productive hours (care hours provided to patients) worked by registered nurses in a defined period/ Total number of patients admitted for care in the same defined period	33	45
	Total number of all nursing hours provided to patients in a defined period (includes any combination of registered nursing hours, licensed practice nursing hours and nurse assistant hours)/ Total number of patients admitted for care in the same defined period	11	
	Average number of nursing hours provided by registered nursing and licensed practice nursing in a defined period/average number of patients admitted for care in the same defined period	1	
Patient to nurse ratio per shift	Average reported number of patients by nurses on their last shift. Nurses' answers are averaged across a hospital to get a value.	32	32
Bed to nurse ratio/ Nurse per bed ratio	Total number of beds divided by the total number of full-time equivalent registered	2	10

For peer review only

	nurses (RNs) working in the general ward		
	Number of FTE nurses divided by the number of hospital beds	6	
	Number of inpatient nurses per 100 occupied beds	1	
	Infection control nurse to bed ratio	1	
Nurse to patient ratio/ patient to nurse ratio	The ratio of nurse productive hours worked to "patient census days/24	1	7
	The ratio of total nurse staff to the total number of patients in a defined period	2	
	The measure of the number of patients assigned to each nurse who has a direct patient care assignment	2	
	Undefined	2	
RN FTE per 1000 patient days	Number of Full-time registered nurses per 1000 patient-days	5	6
	The number of registered nurse full-time equivalents (FTEs) per 1000 in-patient days.	1	
RN per adjusted admission	Number of registered nurses/ adjusted admissions	1	2
	Number of full-time equivalent registered nurses per 100 adjusted admissions	1	
Others*		18	

*Others – Includes metrics that have frequencies of 1. This group includes - nursing hour per patient per shift, Inpatient clinical nursing worked hours per OCW), Mean difference between actual and expected nurse to patient ratio, RN hours per adjusted patient day, RN FTE, Total nursing hours, FTE RN to total hospital adjusted patient day, RN FTE per hospital, Daily average hours of care, All hours of direct care per patient day, RN per average daily census, The ratio of required to actual patient care hours, Required nursing personnel per shift, Number of licensed nurses, FTE RNs per adjusted average daily census, labour efficiency ratio, Daily average utilisation of RNs, Acuity-adjusted nursing hour per patient day

For peer review only

For peer review only

PRISMA 2020 Checklist

Section and Topic	Item #	Checklist item	Location where item is reported
**TITLE**			
Title	1	Identify the report as a systematic review.	Title page
**ABSTRACT**			
Abstract	2	See the PRISMA 2020 for Abstracts checklist.	Abstract (pg 2)
**INTRODUCTION**			
Rationale	3	Describe the rationale for the review in the context of existing knowledge.	Background (pg 5)
Objectives	4	Provide an explicit statement of the objective(s) or question(s) the review addresses.	Aims and Objectives (pg 6)
**METHODS**			
Eligibility criteria	5	Specify the inclusion and exclusion criteria for the review and how studies were grouped for the syntheses.	Selection of systematic reviews (pg 7)
Information sources	6	Specify all databases, registers, websites, organisations, reference lists and other sources searched or consulted to identify studies. Specify the date when each source was last searched or consulted.	Data sources and search strategy (pg 6 & 7)
Search strategy	7	Present the full search strategies for all databases, registers and websites, including any filters and limits used.	Data sources and search strategy (pg 6 & 7)
Selection process	8	Specify the methods used to decide whether a study met the inclusion criteria of the review, including how many reviewers screened each record and each report retrieved, whether they worked independently, and if applicable, details of automation tools used in the process.	Selection of systematic reviews (pg 7)
Data collection process	9	Specify the methods used to collect data from reports, including how many reviewers collected data from each report, whether they worked independently, any processes for obtaining or confirming data from study investigators, and if applicable, details of automation tools used in the process.	Data extraction (pg 8)
Data items	10a	List and define all outcomes for which data were sought. Specify whether all results that were compatible with each outcome domain in each study were sought (e.g. for all measures, time points, analyses), and if not, the methods used to decide which results to collect.	Selection of systematic reviews (pg 7)
	10b	List and define all other variables for which data were sought (e.g. participant and intervention characteristics, funding sources). Describe any assumptions made about any missing or unclear information.	Selection of systematic reviews (pg 7)
Study risk of bias assessment	11	Specify the methods used to assess risk of bias in the included studies, including details of the tool(s) used, how many reviewers assessed each study and whether they worked independently, and if applicable, details of automation tools used in the process.	Quality assessment (pg 8)
Effect measures	12	Specify for each outcome the effect measure(s) (e.g. risk ratio, mean difference) used in the synthesis or presentation of results.	Selection of systematic reviews, Data

For peer review only

PRISMA 2020 Checklist

Section and Topic	Item #	Checklist item	Location where item is reported
			synthesis (pg 7 and pg 9)
Synthesis methods	13a	Describe the processes used to decide which studies were eligible for each synthesis (e.g. tabulating the study intervention characteristics and comparing against the planned groups for each synthesis (item #5)).	Data synthesis (pg 9)
	13b	Describe any methods required to prepare the data for presentation or synthesis, such as handling of missing summary statistics, or data conversions.	Data synthesis (pg 9)
	13c	Describe any methods used to tabulate or visually display results of individual studies and syntheses.	Data synthesis (pg 9)
	13d	Describe any methods used to synthesize results and provide a rationale for the choice(s). If meta-analysis was performed, describe the model(s), method(s) to identify the presence and extent of statistical heterogeneity, and software package(s) used.	Data synthesis (pg 9)
	13e	Describe any methods used to explore possible causes of heterogeneity among study results (e.g. subgroup analysis, meta-regression).	Not applicable
	13f	Describe any sensitivity analyses conducted to assess robustness of the synthesized results.	Not applicable
Reporting bias assessment	14	Describe any methods used to assess risk of bias due to missing results in a synthesis (arising from reporting biases).	Quality assessment (pg 8)
Certainty assessment	15	Describe any methods used to assess certainty (or confidence) in the body of evidence for an outcome.	Not applicable
**RESULTS**			
Study selection	16a	Describe the results of the search and selection process, from the number of records identified in the search to the number of studies included in the review, ideally using a flow diagram.	Search results (figure 1)
	16b	Cite studies that might appear to meet the inclusion criteria, but which were excluded, and explain why they were excluded.	Supplementary file 2
Study characteristics	17	Cite each included study and present its characteristics.	Table 1 (pg 11 & 12)
Risk of bias in studies	18	Present assessments of risk of bias for each included study.	Figure 2
Results of individual studies	19	For all outcomes, present, for each study: (a) summary statistics for each group (where appropriate) and (b) an effect estimate and its precision (e.g. confidence/credible interval), ideally using structured tables or plots.	Not applicable
Results of syntheses	20a	For each synthesis, briefly summarise the characteristics and risk of bias among contributing studies.	Quality assessment of included studies (pg 13), Figure 2
	20b	Present results of all statistical syntheses conducted. If meta-analysis was done, present for each the summary estimate and its precision (e.g. confidence/credible interval) and measures of statistical heterogeneity. If comparing groups, describe the direction of the effect.	Figure 3, Figure 4, Supplementary file 5, 6 and 7

For peer review only

PRISMA 2020 Checklist

Section and Topic	Item #	Checklist item	Location where item is reported
	20c	Present results of all investigations of possible causes of heterogeneity among study results.	Not applicable
	20d	Present results of all sensitivity analyses conducted to assess the robustness of the synthesized results.	Not applicable
Reporting biases	21	Present assessments of risk of bias due to missing results (arising from reporting biases) for each synthesis assessed.	Not applicable
Certainty of evidence	22	Present assessments of certainty (or confidence) in the body of evidence for each outcome assessed.	Not applicable
**DISCUSSION**			
Discussion	23a	Provide a general interpretation of the results in the context of other evidence.	Discussion (pg 15 – 17)
	23b	Discuss any limitations of the evidence included in the review.	Strength and limitations (pg 17 & 18)
	23c	Discuss any limitations of the review processes used.	Strength and limitations (pg 17 & 18)
	23d	Discuss implications of the results for practice, policy, and future research.	Discussion (pg 15 – 17)
**OTHER INFORMATION**			
Registration and protocol	24a	Provide registration information for the review, including register name and registration number, or state that the review was not registered.	Abstract (pg 2), Research design (pg 6)
	24b	Indicate where the review protocol can be accessed, or state that a protocol was not prepared.	Research design (pg 6)
	24c	Describe and explain any amendments to information provided at registration or in the protocol.	Research design (pg 6)
Support	25	Describe sources of financial or non-financial support for the review, and the role of the funders or sponsors in the review.	Funding (pg 20)
Competing interests	26	Declare any competing interests of review authors.	Competing interests (pg 20)
Availability of data, code and other materials	27	Report which of the following are publicly available and where they can be found: template data collection forms; data extracted from included studies; data used for all analyses; analytic code; any other materials used in the review.	Data availability statement (pg 20)

From: Page MJ, McKenzie JE, Bossuyt PM, Boutron I, Hoffmann TC, Mulrow CD, et al. The PRISMA 2020 statement: an updated guideline for reporting systematic reviews. BMJ 2021;372:n71. doi: 10.1136/bmj.n71

For more information, visit: http://www.prisma-statement.org/

For peer review only

BMJ Open

IDENTIFYING GAPS IN GLOBAL EVIDENCE FOR NURSE STAFFING AND PATIENT CARE OUTCOMES RESEARCH IN LOW AND MIDDLE-INCOME COUNTRIES: AN UMBRELLA REVIEW

Journal:	*BMJ Open*
Manuscript ID	bmjopen-2022-064050.R1
Article Type:	Original research
Date Submitted by the Author:	24-Sep-2022
Complete List of Authors:	Imam, Abdulazeez; KEMRI-Wellcome Trust Research Programme Nairobi, Health Services Unit; University of Oxford, Nuffield Department of Medicine<br>Obiesie, Sopuruchukwu; Independent researcher,<br>Aluvaala, Jalemba ; KEMRI-Wellcome Trust Research Programme Nairobi, Health Services Unit; University of Nairobi, Department of Paediatrics and Child Health<br>Maina, Jackson; KEMRI-Wellcome Trust Research Programme Nairobi, Health Services Unit<br>Gathara, David; KEMRI-Wellcome Trust Research Programme Nairobi, Wellcome Trust Research Program ; London School of Hygiene and Tropical Medicine Centre for Maternal Adolescent Reproductive and Child Health, MARCH Centre<br>English, Mike; KEMRI-Wellcome Trust Research Programme Nairobi, Health Services Unit; Nuffield Department of Clinical Medicine,
**Primary Subject Heading**:	Global health
Secondary Subject Heading:	Nursing
Keywords:	Health policy < HEALTH SERVICES ADMINISTRATION & MANAGEMENT, Health & safety < HEALTH SERVICES ADMINISTRATION & MANAGEMENT, Quality in health care < HEALTH SERVICES ADMINISTRATION & MANAGEMENT, PUBLIC HEALTH

SCHOLARONE™
Manuscripts

I, the Submitting Author has the right to grant and does grant on behalf of all authors of the Work (as defined in the below author licence), an exclusive licence and/or a non-exclusive licence for contributions from authors who are: i) UK Crown employees; ii) where BMJ has agreed a CC-BY licence shall apply, and/or iii) in accordance with the terms applicable for US Federal Government officers or employees acting as part of their official duties; on a worldwide, perpetual, irrevocable, royalty-free basis to BMJ Publishing Group Ltd ("BMJ") its licensees and where the relevant Journal is co-owned by BMJ to the co-owners of the Journal, to publish the Work in this journal and any other BMJ products and to exploit all rights, as set out in our licence.

The Submitting Author accepts and understands that any supply made under these terms is made by BMJ to the Submitting Author unless you are acting as an employee on behalf of your employer or a postgraduate student of an affiliated institution which is paying any applicable article publishing charge ("APC") for Open Access articles. Where the Submitting Author wishes to make the Work available on an Open Access basis (and intends to pay the relevant APC), the terms of reuse of such Open Access shall be governed by a Creative Commons licence – details of these licences and which Creative Commons licence will apply to this Work are set out in our licence referred to above.

Other than as permitted in any relevant BMJ Author's Self Archiving Policies, I confirm this Work has not been accepted for publication elsewhere, is not being considered for publication elsewhere and does not duplicate material already published. I confirm all authors consent to publication of this Work and authorise the granting of this licence.

For peer review only

IDENTIFYING GAPS IN GLOBAL EVIDENCE FOR NURSE STAFFING AND PATIENT CARE OUTCOMES RESEARCH IN LOW AND MIDDLE-INCOME COUNTRIES: AN UMBRELLA REVIEW

Abdulazeez Imam[1,2] Sopuruchukwu Obiesie[3] Jalemba Aluvaala[1,4] Michuki Maina[1] David Gathara[1,5] Mike English [1,2]

[1] KEMRI-Wellcome Trust Research Programme, Nairobi, Kenya

[2]Oxford Centre for Global Health Research, Nuffield Department of Medicine, University of Oxford, S Parks Rd, Oxford, OX1 3SY, UK

[3]Independent researcher, Delta State, Nigeria

[4]Department of Paediatrics, University of Nairobi, Nairobi, Kenya

[5]MARCH Centre, London School of Hygiene and Tropical Medicine, London, UK

Corresponding author:

Abdulazeez Imam

KEMRI-Wellcome Trust Research Programme, Nairobi, Kenya

Email: abdulimam2001@yahoo.com

Word Count - 3706

Keywords – Quality of care, Developing countries, Nursing staff, Patient safety, Health systems

For peer review only - http://bmjopen.bmj.com/site/about/guidelines.xhtml

Abstract

Objective To identify nurse staffing and patient care outcome literature in published systematic reviews and map out the evidence gaps for Low-middle Income Countries (LMICs).

Methods

We included quantitative systematic reviews on nurse staffing levels and patient care outcomes in regular ward settings published in English. We excluded qualitative reviews or reviews on nursing skill mix. We searched the Cochrane Register of Systematic Reviews, the JBI Database of Systematic Reviews and Implementation Reports, Medline, Embase and Cumulative Index to Nursing and Allied Health Literature (CINAHL) from inception till July 2021. We used the AMSTAR-2 criteria for risk of bias assessment and conducted a narrative synthesis.

Results From 843 papers, we included fourteen in our final synthesis. There were overlaps in primary studies summarised across reviews, but overall, the reviews summarised 136 unique primary articles. Only four out of 14 reviews had data on a LMIC publications and only 9 (6.6%) of 136 unique primary articles were conducted in LMICs. Only 8 of 23 patient care outcomes were reported from LMICs. Less research was conducted in contexts with staffing levels that are typical of many LMIC contexts.

Discussion Our umbrella review identified very limited data for nurse staffing and patient care outcomes in LMICs. We also identified data from HICs might not be good proxies for LMICs as staffing levels where this research was conducted had comparatively better staffing levels than the few LMIC studies. This highlights a critical need for the conduct of nurse staffing research in LMIC contexts.

Limitations We included data on systematic reviews that scored low on our risk of bias assessment because we sort to provide a broad description of the research area. We only considered systematic reviews published in English and did not include any qualitative reviews in our synthesis.

For peer review only - http://bmjopen.bmj.com/site/about/guidelines.xhtml

PROSPERO registration number: CRD42021286908.

Strengths and limitations

- This umbrella review comprehensively searched across 5 electronic databases for systematic reviews on the association between nurse staffing and patient care outcomes
- We conducted our umbrella review using guidance from the Joanna Briggs Institute, which is the standard for conducting umbrella reviews.
- We had a broad focus to describe the global evidence and identify gaps for LMICs and so we included reviews that scored low on our risk of bias scores.
- We did not include systematic reviews published in English or qualitative reviews

For peer review only - http://bmjopen.bmj.com/site/about/guidelines.xhtml

BACKGROUND

Donabedian in his seminal work on quality of care describes a structure-process-outcome framework.(1) In his framework, structures present within health services influence the processes of care which in turn are likely to affect care outcomes.(1) A key element of health service structure is staffing and nurses who represent a large percentage of hospital staffing are thus likely to have significant effects on hospital patient outcomes.(2) Nurses play multiple, crucial roles planning, delivering and coordinating care and represent a key part of the hospitals' surveillance system in detecting adverse patient events.(3)

An adequate nursing workforce is central to the delivery of quality patient care. Research that comes from mainly High-Income Countries (HICs) demonstrates that poorer ward nurse staffing is associated with negative patient care outcomes, for example, increased risk for patient mortality, prolonged hospital stay and an increased risk for hospital-acquired complications.(4 - 14)In these studies, staffing levels have been measured using metrics which measure the number of nurses or nursing care hours delivered by them relative to patient numbers or proxy measures for patient numbers, for example hospital beds. (4 - 14) This has led to a shift towards improved staff-to-patient ratios or minimum nurse staffing standards to guide ward nurse staffing levels in many HICs. In 1999, California state adopted a bill mandating the state Department of Health Services to enforce minimum staffing ratios across hospitals within the state and this has been the focus of multiple research studies investigating the effects on the quality of patient care. (15 - 18) Shortly after this, in 2002, the state of Western Australia used the Nursing Hour Per Patient Day method which classifies wards into seven categories based on patient care acuity and complexity to determine relative nurse staffing needs. (19, 20) In other countries such as the United Kingdom, the Royal College of Nursing also provides minimum staffing guidance for wards, for example, paediatric wards are

For peer review only - http://bmjopen.bmj.com/site/about/guidelines.xhtml

required to maintain a minimum ratio of 1 registered nurse to 4 children with better ratios applied to high dependency and intensive care settings. (21)

The evolution and the evidence for nurse staffing in LMICs are less clear. In more resource challenged LMIC settings, nurse staffing ratios might be as low as 1 nurse caring for over 25 patients on a shift.(22, 23) For such settings, the World Health Organization has promoted the Workload Indicator Staffing Needs (WISN) planning tool which relies on data from health information systems. (24) The central focus of the WISN tool is the workload of the average health worker and the time to carry out their activities as defined by experts. (24) It aims to provide context-specific estimates of workforce requirements that are based on service types and complexity within local health facilities. However, the WISN approach often suggests workforce expansion that seems unachievable for countries due to the major increases in rates of production and financing required, highlighting the practical realities of human resource costs which can represent up to two-thirds of health service budgets. (25, 26) It is highly likely that the current extreme staffing ratios in LMICs contribute to poor patient outcomes and overall poor-quality care and research to understand this is crucial.

A recent systematic review on the impact of nurse staffing on patient and nurse workforce outcomes in LMICs demonstrated limited and poor-quality evidence on the role of nurse staffing and patient care outcome in LMICs. (27) A crucial next step to guide the conduct of research in these settings is to examine the existing evidence gaps for research in LMICs. One way to do this, would be to compare the evidence in these settings to HICs which have comparatively greater volume of research. An immediate challenge to this is the increasing and expanding volume of literature in this area of research which has likely resulted in more focused systematic reviews over the last decade. (10, 27, 28) More recent reviews have either focused on specific patient populations or regions of the world,(27) or specific patient care outcomes, (10) (28) contrasting earlier reviews which were more broad-based. (14, 29)

For peer review only - http://bmjopen.bmj.com/site/about/guidelines.xhtml

Umbrella reviews synthesise the information from systematic reviews and can serve as a more efficient synthesis method for areas where large volumes of research have been conducted, integrating research to provide broader knowledge on specific topics. (30) Using this method, we appraised the existing global evidence related to nurse staffing and patient care outcome research in published systematic reviews and compared the evidence available for LMICs to HICs , so we could identify the existing evidence gaps for research in LMICs.

Aim and objectives

The primary objective of this umbrella review is to identify the evidence gaps for LMICs in the existing literature that investigates the association between hospital nurse staffing and patient care outcomes. We will address the following research questions:

a. Where are studies within published reviews conducted and what proportion of these are carried out in LMICs?
b. What patient care outcomes are reported across reviews and how do reported outcomes differ between HICs and LMIC studies?
c. What is the range of nurse staffing levels that have been researched across acute care settings and how do these differ between LMICs and HICs?

For peer review only

For peer review only - http://bmjopen.bmj.com/site/about/guidelines.xhtml

METHODS

Research design

We conducted an umbrella review using guidance from the Joanna Briggs Institute for the preparation and conduct of the review. (30) Our review protocol was registered with the International Prospective Register of Systematic Reviews (PROSPERO), Registration number - CRD42021286908 and has been published. (31)

Data sources and search strategy

To identify published systematic reviews that reported on the association between nurse staffing and patient care outcomes, we conducted a systematic search of 5 electronic databases from their inception up until July 2021. These were the Cochrane Register of Systematic Reviews, the JBI Database of Systematic Reviews and Implementation Reports, Medline, Embase and Cumulative Index to Nursing and Allied Health Literature (CINAHL). Our search strategy for this is detailed in Supplemental file 1 and has previously been published.(31)

We included some of the following keywords and their synonyms: nurse, nursing, outcome, quality, missed nursing, mortality and identified some key MESH terms (Supplemental file 1). We combined these using Boolean operators “AND” and “OR” where appropriate. We also searched the reference list of our included systematic reviews to identify other reviews.

For peer review only - http://bmjopen.bmj.com/site/about/guidelines.xhtml

144 ## Selection of systematic reviews

145 We included quantitative systematic reviews that: a.) summarised literature on nurse staffing levels and
146 patient care outcomes in regular ward settings; b) were conducted in English language (due to limitations
147 in translation among the research team).

148 Reviews were excluded if they: a) were conducted in a non-hospital setting; b) only summarised studies
149 conducted in non-regular ward settings, for example, intensive care units; c) reported exclusively on non-
150 patient care outcomes, such as nurse outcomes (for example, burnout and nurse satisfaction); d) reported
151 on other nurse staffing characteristics other than staffing levels, for example, skill mix (a measure of nurse
152 organisation); e) were qualitative and mixed-method reviews.

153 ### Population

154 The participants in the included systematic reviews were patient admitted to regular hospital ward settings.
155 For reviews reporting on patients in both standard ward settings and intensive care units, we include these
156 but only summarised and reported on specific data which focused on our inclusion criteria.

157 ### Exposure

158 Our exposure for this review is the level of nurse staffing and thus reflects nursing metrics that focus on
159 nursing numbers or nursing time available to patients, for example, nurse-to-patient ratios or nursing
160 hours per patient days and not on how the nursing workforce is organised.(31)

161 ### Outcome

162 Our outcome of interest was patient care outcomes, for example, mortality and health care associated
163 infection. For reviews that reported mixed outcomes or care settings, for example, reviews summarising
164 literature on both nurse and patient outcomes.

For peer review only - http://bmjopen.bmj.com/site/about/guidelines.xhtml

Screening

References were managed using the Zotero reference software. (32) We performed initial deduplication in Zotero and a 2nd round of deduplication in Microsoft excel. Following which we exported our final set of articles to Rayyan, a web-based application for screening, (33) where two reviewers, AI and SO independently screened the titles and abstracts of all identified systematic reviews for eligibility. They then read full texts of selected articles and agreed on a final set of papers.

Quality assessment

We evaluated each systematic review for risk of bias using the AMSTAR-2 criteria.(34) This tool comprises 16 appraisal questions rated 'Yes' when a criterion is fulfilled; 'No' when it is not fulfilled and 'Partial Yes' when it is somewhat fulfilled (This is based on explicitly stated criteria). The AMSTAR criteria cover important questions such as whether the PICO elements were clearly described by the review, whether the conduct of study selection, data abstraction and risk of bias were conducted by at least two reviewers and the use of a comprehensive search strategy.(34) The quality appraisal was performed independently by AI and SO and disagreements were managed through discussions.

Data extraction

Both reviewers (AI and SO) independently extracted data from the final set of systematic reviews using a pre-designed data abstraction tool. This data included the review publication year, objectives, reported patient care outcomes, number and origin of review studies that reported on nurse staffing and patient outcomes in regular ward care settings.

Summary data on the nurse staffing metrics were also retrieved from the individual paper summaries within systematic reviews. Where this information was unavailable as a summary within the reviews, we

For peer review only

For peer review only - http://bmjopen.bmj.com/site/about/guidelines.xhtml

abstracted them directly from the individually referenced articles. AI abstracted this data and SO cross-checked a random 40% of this.

Data synthesis

Our umbrella review findings are presented in narrative form using tables and figures. We tallied the countries of origin of the individually referenced papers within each review and classified these into LMIC and non-LMIC using the World Bank country and lending group classification system as of 23rd December 2021.(35) We also summarised and tallied all reported patient care outcomes from the included systematic reviews and made comparisons between made across LMICs and HICs.

We identified and collated summary statistics (range, mean or median) for nurse staffing metrics reported in individually referenced papers using tables and bar charts where these were reported. Because of the heterogeneity of retrieved metrics, a meta-analysis was not possible.

Patient and public involvement

There was no patient or public involvement in the design and conduct of this study.

Ethics statement

As we used secondary data sources, we did not require ethical approval for the conduct of this review.

For peer review only - http://bmjopen.bmj.com/site/about/guidelines.xhtml

RESULTS

Search results

Our initial search of databases identified 1365 articles. We identified and excluded 522 duplicate articles and screened the title and abstract of 843 remaining articles. Of the 33 systematic reviews that met our criteria for full text reviews, we included 14. (10, 14, 19 ,27 - 29, 36 - 43) The PRISMA flow chart in Figure 1 provides a summary of our screening process while Supplemental file 2 shows the list of excluded systematic reviews and the reasons for their exclusion.

Description of included reviews

We included 14 systematic reviews in our final analysis. The reviews covered periods from as early as 1980 up until 2020 (Table 1). The reviews were conducted on studies from predominantly medical, surgical or adult wards with only one review conducted on those from Paediatric ward settings (39). The reviews included between 5 and 54 primary studies. Of the individual studies within reviews, between 12.5% and 100.0% met our inclusion (Table 1). This was because some reviews in addition to summarising studies that investigated the effect of nurse staffing on patient care outcomes also included those that reported other outcomes, for example, nurse outcomes. (27) Other reviews also included studies conducted in non-regular ward settings such as intensive care unit settings, (28, 38) or included a mixture of studies investigating nurse staffing and other interventions such as nurse skill mix. (14) In Supplemental file 3, for each review, we have provided a list of primary studies we did not report on and the reasons for this. There was some overlap in the primary articles summarised by the reviews, some papers were referenced in as many as 6 reviews (Supplemental file 4). In total 136 unique primary studies were identified across all systematic reviews (Supplemental file 4).

For peer review only - http://bmjopen.bmj.com/site/about/guidelines.xhtml

For peer review only

For peer review only - http://bmjopen.bmj.com/site/about/guidelines.xhtml

Table 1: An overview of the included systematic reviews showing the review objective and geographical locations where the reviews primary studies were conducted

First author (Year)	Period of review	Research setting	Number of primary studies included	Number of primary studies describing nurse staffing levels and patient outcomes in non-ICU settings[&]	Geographic locations of studies and frequency	Number of low-middle income countries included (%)	Number of high-income countries included (%)
Griffiths (2018)	2006 - 2016	Adult hospital in-patient wards	18	17	Kuwait - 1, Europe -3, UK-1, Sweden -1, US* - 7, Lebanon* -1, Switzerland-1,Italy- 1, South Korea- 2	1 (5.6)	17 (95.4)
Assaye (2020)	UR - 2019	Acute care hospital settings	27	6	Brazil – 1, Thailand – 2, China – 1, Ethiopia – 1, Lebanon - 1	6 (100.0)	0 (0.0)
Thungjaroenkul (2007)	1990 - 2006	ICUs, medical and surgical units	17	5	US - 5	0 (0.0)	5 (100.0)
Twigg (2020)[$]	2000 - 2020	General medical, surgical, step-down units ,emergency departments, intensive care, and nursing homes	22	7	US – 6, Australia - 1	0 (0.0)	7 (100.0)
Shin (2019)	2000 - 2018	Medical and surgical units	19	17	US – 10, South Korea – 2, Finland* - 1, Netherland* - 1, Lebanon – 1, Belgium – 1, China -1, Japan - 1	2(11.1)	16 (88.9)

For peer review only - http://bmjopen.bmj.com/site/about/guidelines.xhtml

Lang (2004)$	1980 - 2003	Acute care hospitals	43	24	US - 24	0 (0.0)	24 (100.0)
Lankshear (2005)	1990 - 2004	Acute care hospitals	22	19	US – 18, Canada -1	0 (0.0)	19 (100.0)
Kane (2007)	1990 - 2006	Acute care hospitals	28	17	US - 17	0 (0.0)	17 (100.0)
Bourgon (2019)	1996 - 2018	Surgical units	44	28	US – 17, Europe - 2, South Korea - 1, UK – 2, New Zealand – 1, Australia – 2, Belgium – 2, Japan - 1	0 (0.0)	28 (100.0)
Wilson (2011)	1993-2010	Paediatric wards	8	5	US – 2, France – 1, Canada -1, UK - 1	0 (0.0)	5 (100.0)
Engineer (2016)$	2000-2012	Acute care hospitals	16	2	US - 2	0 (0.0)	2 (100.0)
Staplers (2015)	2004 - 2012	Acute care hospitals	29	16	US* - 12, Belgium* - 1, New Zealand – 1, Australia – 1, UK – 1, Sweden - 1	0 (0.0)	17 (100.0)
Mitchell (2018)	2000 - 2015	Hospital wards	54	19	US – 13, Thailand – 1, Australia -1, Taiwan – 1, Canada – 2, Multi-country - 1	1 (4.3)	18 (95.7)
Hill (2017)	1994 - 2017	Acute care hospitals	5	5	US – 3, UK - 2	0 (0.0)	5 (100.0)

*- one study conducted across both countries, $- restricted search criteria to High income countries

&- These are the number of studies that met the original review criteria .i.e., conducted in a regular ward setting on the role of nurse staffing and patient care outcomes. UR – Unreported

For peer review only - http://bmjopen.bmj.com/site/about/guidelines.xhtml

Quality assessment of included reviews

We used the AMSTAR-2 quality assessment tool to appraise individual systematic reviews. (34) As shown in Figure 2, Question 2 (Q2), 11 out of 14 of the reviews did not refer to a study protocol or provide explicit statements of their review methods being established before the review conduct. Most reviews (12 out of 14) did not provide a list of excluded studies with justifications on reasons for excluding these studies (Figure 2, Q7). Discussions on how the risk of bias assessments of individual studies might have impacted the review results were also limited (Figure 2, Q13)

Origin of primary studies and proportion of studies in systematic reviews conducted in LMIC settings.

Of the 136 unique studies, 94 (69.1%) were carried out in the USA. Only 5 LMIC countries, Thailand, Brazil, Lebanon, China, and Ethiopia accounted for 9 (6.6%) of 136 primary studies (Supplemental file 5). Studies from HICs came from more diverse locations. These studies were conducted in 16 other settings in addition to the USA (Supplemental file 5). Four out of 14 of our included systematic reviews reported data on our subject of interest (nurse staffing and patient care outcomes) from a LMIC (Table 1). One review restricted to LMIC settings identified only 6 articles, while 3 reviews were primarily restricted to HICs (Table 1).

For peer review only

Reported patient care outcomes and differences across research settings (LMICs and HICs)

In total, the included reviews reported 23 patient care outcomes. Each review reported between 1 and 15 outcomes and the most frequently reported (8 times each) were patient mortality, pressure ulcers and the incidence of hospital-acquired infections (Figure 3).

Only 8 of the 23 patient care outcomes were reported in an LMIC setting. These were missed nursing care, mortality, pressure ulcers, length of stay, treatment errors, hospital-acquired infections, falls and hospital-acquired injuries (Figure 3). Other outcomes such as postoperative complications, cardiac arrests, deep venous thrombosis, failure to rescue, unplanned extubations and incidence of restraint use were not reported in LMICs (Figure 3).

Measures of nurse staffing and reported staffing range across LMICs and HICs

There were 29 different reported nurse staffing metrics across the individual studies within our included systematic reviews. The most frequently reported metrics were the Nursing Hour Per Patient Day (NHPPD) and the patient to nurse ratio per shift which were reported by 48 and 34 primary studies respectively (Supplemental file 6). There was marked heterogeneity in how both metrics were reported by papers. Some reported absolute values, mean, median, ranges, percentiles, or categories. For those reporting either a range, mean or median, we have summarised their values in Figures 4 and 5.

Figure 4 depicts patients per nurse for 9 studies (8 HICs and 1 LMIC study). Studies from HICs reported best staffing ratios (smallest patient per nurse ratio) ranging between 4 patients per nurse and 7, (7, 44 - 50) and this was in contrast to the sole LMIC study from Brazil which reported a best ratio of 9 patients

For peer review only - http://bmjopen.bmj.com/site/about/guidelines.xhtml

per nurse.(51) Similarly, the worst rations documented in studies ranged between 9 and 18 patients per nurse within HICs in contrast to 27 patients per nurse in the LMIC study (Figure 4).(51)

All studies which reported the mean patient to nurse ratio per shift were in HICs and 16 of 18 reported mean values below 10 (Figure 5). (44, 45, 49, 50, 52, 53 - 62) For studies reporting total mean Nursing Hour Per Patient Day (NHPPD), the 3 reported LMIC studies (from Thailand and Lebanon) had the lowest values,(63, 64) and these were almost 2.5-fold below studies conducted in some HICs (Figure 5). (65 - 70)

DISCUSSION

This umbrella review of published systematic reviews on nurse staffing and patient care outcomes examined the evidence gaps for LMICs by appraising the global evidence. We found a dearth of evidence from LMICs with the link between nurse staffing and quality exploring a narrower range of patient care outcomes.

Of the 14 included systematic reviews in this study, 10 (71.4%) had no data from an LMIC setting. Across all 14 reviews only 9 of the 136 unique primary studies included were from LMICs and these data came from five countries (Thailand, Lebanon, China, Brazil and Ethiopia), (51, 63, 64, 71-76) with one from Africa (Ethiopia).(76) A recent LMIC-focused systematic review found only 6 nurse staffing and patient care outcome papers that were graded as providing low-quality evidence.(27) With 90% of the global deficit of nurses concentrated in LMIC settings,(2) research is needed in such settings to examine the impact these shortages have on the quality of hospital care. Ideally these studies would examine the impact of improving staffing numbers on the overall quality of patient care that is provided and patients' health outcomes. This data is critical to highlight the effects of the current staffing shortages and to guide policymakers and managers in developing appropriate staffing policies.

For peer review only - http://bmjopen.bmj.com/site/about/guidelines.xhtml

The data that currently exists illustrates the negative effects of low nurse staffing on quality of care but is almost exclusively from High-Income Countries (HIC). (7, 53, 77) Traditionally, these areas already have better staffing ratios than LMICs and yet relatively small absolute or relative improvements in staffing can demonstrate positive effects. We noted nurse staffing metrics reported in LMICs were more extreme in comparison with HICs. For example, patient to nurse ratio, a metric that measures the number of patients per nurse on a shift was reported to be as high as 27 patients in a Brazil study,(51) and this starkly contrasts with figures of between 4 and 9 patients per nurse from US studies.(44, 49) Also, nursing hour per patient day (NHPPD) which measures the total number of nursing hours provided in a defined period adjusted for the number of admissions in the same period ranged between 4.25 and 5.33 in LMICs,(63, 64, 71) in contrast with some values from the US were 2.5 fold higher.(65) Because the nurse-to-patient ratios are comparatively poorer in LMICs, increasing nurse staffing might potentially lead to larger impacts on quality of patient care and overall patient outcomes. Local research in these contexts is however needed to confirm this.

Staffing levels in the literature were measured using various proxy metrics. While NHPPD and Patient per nurse ratios were the most frequently employed staffing metrics across studies, we observed a wide heterogeneity in reported staffing metrics across studies. In total, there were 29 reported unique nurse staffing metrics across the literature and report of their summary statistics were inconsistent across studies. This limited our ability to collate findings across multiple studies to examine the range of nurse staffing where this research has been conducted. Previous reviews have also documented similar heterogeneity in nurse staffing research and literature.(14) Progress within this area of research would benefit from a move towards standardisation and reporting of core metrics across different studies and research settings to promote comparative learning across contexts.

As a proxy for the quality of patient care, a range of outcomes was reported across systematic reviews. Together all our included reviews reported 23 different outcomes with the most popular being patient

For peer review only - http://bmjopen.bmj.com/site/about/guidelines.xhtml

mortality, pressure ulcers and the incidence of hospital-acquired infections. Only 8 of these outcomes were reported in studies from LMIC settings. The reported patient care outcomes in LMICs were missed nursing care, mortality, the incidence of pressure ulcers, patient length of stay, treatment errors, hospital-acquired infections, and falls. Other outcomes such as incidence of deep venous thrombosis or postoperative complications were not reported. One reason might be limited diagnostic capacity in LMICs; many patient complications may go unrecognised and so the burden of these is under-recognised with no data collected. This finding probably also reflects limited health administrative and secondary data sources in LMICs which preclude the reporting of these data. Indeed, studies within these contexts have been based on reviewing paper-based case records which are usually unstructured and have not been curated for research purposes. (51, 76) There are now some examples of networks collecting secondary data in LMICs. For example, in Kenya since 2013, the Clinical Information Network collects secondary data using structured admission records from a network of 23 hospitals and this data has been used repeatedly in quality improvement research,(78) including tracking patient quality indicators such as the frequency at which patient vital signs were conducted.(79) Such databases might play a role in reducing the evidence gap and are likely to be crucial in guiding research to policy. Interestingly, one outcome which does not need advanced diagnostics or secondary data collection is patient satisfaction, but there was no report linking nurse staffing to patient satisfaction from an LMIC.

The majority of the reported outcomes within reviews (22 out of 23) were outcome-based measures of quality of care, except for missed nursing care, a care outcome that describes partially or completely omitted or delayed nursing care (11) which we identified as the sole process-based measure of the quality of care. Care processes occur more frequently than outcomes and as such are useful as studies employing them might be of more manageable size. They might also represent important measures for intervention research aimed at either demonstrating the influence of enhancements in nurse staffing or determining

For peer review only - http://bmjopen.bmj.com/site/about/guidelines.xhtml

the likely influence of an intervention on the quality of patient care due to their likely proximal position in the causal pathway of quality of patient care.

Strengths and limitation

To the best of our knowledge, this is the first review to provide a comprehensive review of the evidence for LMICs in published systematic reviews on the association between nurse staffing and patient care outcomes. We highlight critical gaps in these care settings that need to be the focus of future research in this area.

We recognise that the quality of the information provided by this umbrella review is highly dependent on the underlying quality of the systematic reviews. Some of our included systematic reviews scored low on our risk of bias scores, but because our focus was a broad description of the global evidence to identify gaps for LMICs we still include these reviews in our synthesis. Due to limitations in translation on the research team, we only considered systematic reviews published in English. We also did not consider qualitative reviews as our umbrella review focused on reviews that investigated an association between nurse staffing and patient care outcomes.

Conclusions

Our umbrella review demonstrates that the evidence examining links between nurse staffing and patient care outcomes in LMICs is very limited, both in terms of the number of studies and the outcomes that have been investigated. While there is research conducted in HICs, these might be poor proxies for LMIC settings as staffing ratios in these settings are quite different. Research in LMIC settings is clearly needed to examine the effect of nursing shortages and interventions addressing these on the overall quality of

For peer review only - http://bmjopen.bmj.com/site/about/guidelines.xhtml

patient care and inform local staffing policy in these regions. To enhance learning across contexts there needs to be greater uniformity and standardization of metrics used to measure nurse staffing.

Figure 1 – PRISMA diagram for search

Figure 2 – Risk of Bias assessment using the AMSTAR-2 checklist. (Key: Red – No, Green – Yes, lighter green – Partially Yes, Yellow – Not applicable)

Figure 3: Stacked bar chart showing the range of patient care outcomes reported across reviews and the number of reviews which report this in a LMIC study (Blue- number of reviews reporting the outcome from a LMIC study, Orange – number of reviews reporting the outcome from only non-LMIC studies

Figure 4 –Bar chart showing the patient to nurse ratio per shift range described in individual studies that reported this metric and described a range (countries of origin and first author of the individual studies are presented, and the Y-axis represents the number of patients per nurse).

Figure 5 – Horizontal bar charts showing the mean total nursing hour per patient day (top) and the mean patient to nurse ratios (bottom) across primary studies reporting these metrics. (First author and countries in which individual studies were conducted are depicted on the Y-axis).

For peer review only - http://bmjopen.bmj.com/site/about/guidelines.xhtml

Authors contribution statement

AI conceptualised the idea for the manuscript with inputs from DG, MM, JA, and ME. AI and SO conducted the methods for the paper. AI drafted the manuscript with significant contribution from all authors and under the supervision of DG, MM, JA, and ME. All the authors reviewed all versions of the manuscript and agreed on a final version for submission.

Competing interests

All authors declare no competing interests

Funding

AI is supported for a PhD studentship at the University of Oxford by the National Institute for Health Research [NIHR130812]. ME and JA receive salary support from a Wellcome Trust Senior Research Fellowship (# 207522) awarded to ME. MM & DG receive salary support from the National Institute for Health Research (NIHR)(NIHR130812): Learning to Harness Innovation in Global Health for Quality Care (HIGH-Q) grant using UK aid from the UK government to support global health research. A Wellcome Trust core award to the KEMRI-Wellcome Trust Research Programme (#092654) enables this research.

The funders had no role in the study design, data collection and analysis, or preparation of the manuscript. the views expressed in this publication are those of the authors and not necessarily those of the Wellcome Trust, NIHR, or the UK government

For peer review only - http://bmjopen.bmj.com/site/about/guidelines.xhtml

Data availability statement

Our search strategy is available in the supplemental files and a list of both included and excluded studies have been included in our tables and supplemental files

Ethical approval

This review used secondary data from previously published systematic reviews and so ethical approval or patient consent was not required.

For peer review only

For peer review only - http://bmjopen.bmj.com/site/about/guidelines.xhtml

References

1. Donabedian A. Evaluating the quality of medical care. Milbank Mem Fund Q. 1966;44(3):166–206.

2. Organization WH. State of the world's nursing 2020: investing in education, jobs and leadership. 2020;

3. Clarke SP, Aiken LH. Failure to Rescue: Needless deaths are prime examples of the need for more nurses at the bedside. AJN Am J Nurs. 2003;103(1):42–7.

4. Aiken LH, Sloane D, Griffiths P, Rafferty AM, Bruyneel L, McHugh M, et al. Nursing skill mix in European hospitals: cross-sectional study of the association with mortality, patient ratings, and quality of care. BMJ Qual Saf. 2017;26(7):559–68.

5. Griffiths P, Maruotti A, Saucedo AR, Redfern OC, Ball JE, Briggs J, et al. Nurse staffing, nursing assistants and hospital mortality: retrospective longitudinal cohort study. BMJ Qual Saf. 2019;28(8):609–17.

6. Aiken LH, Clarke SP, Sloane DM, Sochalski J, Silber JH. Hospital nurse staffing and patient mortality, nurse burnout, and job dissatisfaction. Jama. 2002;288(16):1987–93.

7. Rafferty AM, Clarke SP, Coles J, Ball J, James P, McKee M, et al. Outcomes of variation in hospital nurse staffing in English hospitals: cross-sectional analysis of survey data and discharge records. Int J Nurs Stud. 2007;44(2):175–82.

8. Cho E, Sloane DM, Kim EY, Kim S, Choi M, Yoo IY, et al. Effects of nurse staffing, work environments, and education on patient mortality: an observational study. Int J Nurs Stud. 2015;52(2):535–42.

9. Tubbs-Cooley HL, Cimiotti JP, Silber JH, Sloane DM, Aiken LH. An observational study of nurse staffing ratios and hospital readmission among children admitted for common conditions. BMJ Qual Saf. 2013;22(9):735–42.

10. Griffiths P, Recio-Saucedo A, Dall'Ora C, Briggs J, Maruotti A, Meredith P, et al. The association between nurse staffing and omissions in nursing care: A systematic review. J Adv Nurs John Wiley Sons Inc. 2018 Jul;74(7):1474–87.

11. Recio-Saucedo A, Dall'Ora C, Maruotti A, Ball J, Briggs J, Meredith P, et al. What impact does nursing care left undone have on patient outcomes? Review of the literature. J Clin Nurs. 2018;27(11–12):2248–59.

12. Driscoll A, Grant MJ, Carroll D, Dalton S, Deaton C, Jones I, et al. The effect of nurse-to-patient ratios on nurse-sensitive patient outcomes in acute specialist units: a systematic review and meta-analysis. Eur J Cardiovasc Nurs. 2018;17(1):6–22.

13. Shekelle PG. Nurse-patient ratios as a patient safety strategy: A systematic review. Ann Intern Med. 2013;158(5 PART 2):404–9.

For peer review only - http://bmjopen.bmj.com/site/about/guidelines.xhtml

14. Kane RL, Shamliyan TA, Mueller C, Duval S, Wilt TJ. The association of registered nurse staffing levels and patient outcomes: systematic review and meta-analysis. Med Care. 2007;1195–204.

15. Mark BA, Harless DW, Spetz J, Reiter KL, Pink GH. California's minimum nurse staffing legislation: results from a natural experiment. Health Serv Res. 2013;48(2pt1):435–54.

16. Cook A, Gaynor M, Stephens Jr M, Taylor L. The effect of a hospital nurse staffing mandate on patient health outcomes: Evidence from California's minimum staffing regulation. J Health Econ. 2012;31(2):340–8.

17. Burnes Bolton L, Aydin CE, Donaldson N, Storer Brown D, Sandhu M, Fridman M, et al. Mandated nurse staffing ratios in California: a comparison of staffing and nursing-sensitive outcomes pre-and postregulation. Policy Polit Nurs Pract. 2007;8(4):238–50.

18. Hickey PA, Gauvreau K, Jenkins K, Fawcett J, Hayman L. Statewide and national impact of California's staffing law on pediatric cardiac surgery outcomes. JONA J Nurs Adm. 2011;41(5):218–25.

19. Twigg DE, Whitehead L, Doleman G, El-Zaemey S. The impact of nurse staffing methodologies on nurse and patient outcomes: A systematic review. J Adv Nurs. 2021;(7609811, h3l).

20. Twigg D, Duffield C. A review of workload measures: a context for a new staffing methodology in Western Australia. Int J Nurs Stud. 2009;46(1):132–40.

21. Safe, sustainable and productive staffing for neonatal care and children and young people's services. - Search [Internet]. [cited 2022 Apr 7]. Available from: https://www.bing.com/search?q=Safe%2C+sustainable+and+productive+staffing+for+neonatal+care+and+children+and+young+people%E2%80%99s+services.&form=ANNTH1&refig=be16fb2e144349afa9dd5c46a1b5d2ba

22. Assaye AM, Wiechula R, Schultz TJ, Feo R. Nurse staffing models in medical-surgical units of acute care settings: A cross-sectional study. Int J Nurs Pract. 2020;26(1):e12812.

23. Gathara D, Serem G, Murphy GA, Obengo A, Tallam E, Jackson D, et al. Missed nursing care in newborn units: a cross-sectional direct observational study. BMJ Qual Saf. 2020;29(1):19–30.

24. WHO | Workload indicators of staffing need (WISN): selected country implementation experiences [Internet]. WHO. World Health Organization; [cited 2021 Jun 1]. Available from: http://www.who.int/hrh/resources/workload_indicators_health-obs15/en/

25. Gerdtz MF, Nelson S. 5-20: a model of minimum nurse-to-patient ratios in Victoria, Australia. J Nurs Manag Wiley-Blackwell. 2007 Jan;15(1):64–71.

26. WHO | The world health report 2000 - Health systems: improving performance [Internet]. WHO. World Health Organization; [cited 2021 May 31]. Available from: https://www.who.int/whr/2000/en/

For peer review only - http://bmjopen.bmj.com/site/about/guidelines.xhtml

27. Assaye AM, Wiechula R, Schultz TJ, Feo R. Impact of nurse staffing on patient and nurse workforce outcomes in acute care settings in low- and middle-income countries: a systematic review. JBI Evid Synth. 2021;19(4):751–93.

28. Mitchell BG, Gardner A, Stone PW, Hall L, Pogorzelska-Maziarz M. Hospital Staffing and Health Care-Associated Infections: A Systematic Review of the Literature. Jt Comm J Qual Patient Saf. 2018;44(10):613–22.

29. Lang TA, Hodge M, Olson V, Romano PS, Kravitz RL. Nurse-patient ratios: a systematic review on the effects of nurse staffing on patient, nurse employee, and hospital outcomes. JONA J Nurs Adm. 2004 Aug 7;34(7/8):326–37.

30. Aromataris E, Fernandez R, Godfrey CM, Holly C, Khalil H, Tungpunkom P. Summarizing systematic reviews: methodological development, conduct and reporting of an umbrella review approach. JBI Evid Implement. 2015;13(3):132–40.

31. Imam A, Obiesie S, Aluvaala J, Maina M, Gathara D, English M. Nurse staffing and patient care outcomes: protocol for an umbrella review to identify evidence gaps for low and middle-income countries. Wellcome Open Res. 2021;6(363):363.

32. Zotero | Your personal research assistant [Internet]. [cited 2021 Oct 22]. Available from: https://www.zotero.org/

33. Ouzzani M, Hammady H, Fedorowicz Z, Elmagarmid A. Rayyan—a web and mobile app for systematic reviews. Syst Rev. 2016;5(1):1–10.

34. Shea BJ, Reeves BC, Wells G, Thuku M, Hamel C, Moran J, et al. AMSTAR 2: a critical appraisal tool for systematic reviews that include randomised or non-randomised studies of healthcare interventions, or both. bmj. 2017;358.

35. World Bank Country and Lending Groups – World Bank Data Help Desk [Internet]. [cited 2021 Jun 26]. Available from: https://datahelpdesk.worldbank.org/knowledgebase/articles/906519-world-bank-country-and-lending-groups

36. Stalpers D, de Brouwer BJM, Kaljouw MJ, Schuurmans MJ. Associations between characteristics of the nurse work environment and five nurse-sensitive patient outcomes in hospitals: a systematic review of literature. Int J Nurs Stud. 2015;52(4):817–35.

37. Hill B. Do nurse staffing levels affect patient mortality in acute secondary care?. Br J Nurs Mark Allen Publ. 2017;26(12):698–704.

38. Bourgon Labelle J, Audet LA, Farand P, Rochefort CM. Are hospital nurse staffing practices associated with postoperative cardiac events and death? A systematic review. PloS One. 2019;14(10):e0223979.

39. Wilson S, Bremner A, Hauck Y, Finn J. The effect of nurse staffing on clinical outcomes of children in hospital: a systematic review. Int J Evid Based Healthc. 2011;9(2):97–121.

For peer review only - http://bmjopen.bmj.com/site/about/guidelines.xhtml

40. Thungjaroenkul P, Cummings GG, Embleton A. The impact of nurse staffing on hospital costs and patient length of stay: a systematic review. Nurs Econ. 2007;25(5):255–65.

41. Engineer LD, Winters BD, Weston CM, Zhang A, Sharma R, Bass E, et al. Hospital characteristics and the agency for healthcare research and quality inpatient quality indicators: A systematic review. J Healthc Qual. 2016;38(5):304–13.

42. Lankshear AJ, Sheldon TA, Maynard A. Nurse staffing and healthcare outcomes: a systematic review of the international research evidence. Adv Nurs Sci. 2005;28(2):163–74.

43. Shin S, Park JH, Bae SH. Nurse staffing and hospital-acquired conditions: A systematic review. J Clin Nurs. 2019;28(23–24):4264–75.

44. Kutney-Lee A, Aiken LH. Effect of nurse staffing and education on the outcomes of surgical patients with comorbid serious mental illness. Psychiatr Serv. 2008;59(12):1466–9.

45. Ausserhofer D, Zander B, Busse R, Schubert M, De Geest S, Rafferty AM, et al. Prevalence, patterns and predictors of nursing care left undone in European hospitals: results from the multicountry cross-sectional RN4CAST study. BMJ Qual Saf. 2014;23(2):126–35.

46. Bruyneel L, Li B, Ausserhofer D, Lesaffre E, Dumitrescu I, Smith HL, et al. Organization of hospital nursing, provision of nursing care, and patient experiences with care in Europe. Med Care Res Rev. 2015;72(6):643–64.

47. Palese A, Ambrosi E, Prosperi L, Guarnier A, Barelli P, Zambiasi P, et al. Missed nursing care and predicting factors in the Italian medical care setting. Intern Emerg Med. 2015;10(6):693–702.

48. Cho SH, Kim YS, Yeon KN, You SJ, Lee ID. Effects of increasing nurse staffing on missed nursing care. Int Nurs Rev. 2015;62(2):267–74.

49. Friese CR, Kalisch BJ, Lee KH. Patterns and correlates of missed nursing care in inpatient oncology units. Cancer Nurs. 2013;36(6):E51.

50. Aiken LH, Sloane DM, Bruyneel L, Van den Heede K, Griffiths P, Busse R, et al. Nurse staffing and education and hospital mortality in nine European countries: a retrospective observational study. The lancet. 2014;383(9931):1824–30.

51. Magalhães AMM de, Dall'Agnol CM, Marck PB. Nursing workload and patient safety-a mixed method study with an ecological restorative approach. Rev Lat Am Enfermagem. 2013;21:146–54.

52. Hinno S, Partanen P, Vehviläinen-Julkunen K. Nursing activities, nurse staffing and adverse patient outcomes as perceived by hospital nurses. J Clin Nurs. 2012;21(11–12):1584–93.

53. Ball JE, Griffiths P, Rafferty AM, Lindqvist R, Murrells T, Tishelman C. A cross-sectional study of 'care left undone'on nursing shifts in hospitals. J Adv Nurs. 2016;72(9):2086–97.

54. Schubert M, Ausserhofer D, Desmedt M, Schwendimann R, Lesaffre E, Li B, et al. Levels and correlates of implicit rationing of nursing care in Swiss acute care hospitals—a cross sectional study. Int J Nurs Stud. 2013;50(2):230–9.

For peer review only

For peer review only - http://bmjopen.bmj.com/site/about/guidelines.xhtml

55. McHugh MD, Rochman MF, Sloane DM, Berg RA, Mancini ME, Nadkarni VM, et al. Better nurse staffing and nurse work environments associated with increased survival of in-hospital cardiac arrest patients. Med Care. 2016;54(1):74.

56. Cimiotti JP, Aiken LH, Sloane DM, Wu ES. Nurse staffing, burnout, and health care–associated infection. Am J Infect Control. 2012;40(6):486–90.

57. Aiken LH, Clarke SP, Cheung RB, Sloane DM, Silber JH. Educational levels of hospital nurses and surgical patient mortality. Jama. 2003;290(12):1617–23.

58. Brooks Carthon JM, Kutney-Lee A, Jarrín O, Sloane D, Aiken LH. Nurse staffing and postsurgical outcomes in black adults. J Am Geriatr Soc. 2012;60(6):1078–84.

59. Halm M, Peterson M, Kandels M, Sabo J, Blalock M, Braden R, et al. Hospital nurse staffing and patient mortality, emotional exhaustion, and job dissatisfaction. Clin Nurse Spec. 2005;19(5):241–51.

60. Al-Kandari F, Thomas D. Factors contributing to nursing task incompletion as perceived by nurses working in Kuwait general hospitals. J Clin Nurs. 2009;18(24):3430–40.

61. Cheung RB. The relationship between nurse staffing, nursing time, and adverse events in an acute care hospital. University of South Florida; 2002.

62. Prot S, Fontan JE, Alberti C, Bourdon O, Farnoux C, Macher MA, et al. Drug administration errors and their determinants in pediatric in-patients. Int J Qual Health Care. 2005;17(5):381–9.

63. Sujijantararat R. Nursing-sensitive quality indicators: Nosocomial urinary tract infection in a Thai hospital. The University of Alabama at Birmingham; 2001.

64. Sujijantararat R, Booth RZ, Davis LL. Nosocomial urinary tract infection: nursing-sensitive quality indicator in a Thai hospital. J Nurs Care Qual. 2005;20(2):134–9.

65. Blegen MA, Goode CJ, Spetz J, Vaughn T, Park SH. Nurse staffing effects on patient outcomes: safety-net and non-safety-net hospitals. Med Care. 2011;406–14.

66. Blegen MA, Vaughn T. A multisite study of nurse staffing and patient occurrences. Nurs Econ. 1998;16(4):196.

67. Everhart D, Schumacher JR, Duncan RP, Hall AG, Neff DF, Shorr RI. Determinants of hospital fall rate trajectory groups: a longitudinal assessment of nurse staffing and organizational characteristics. Health Care Manage Rev. 2014;39(4):352.

68. Needleman J, Buerhaus P, Mattke S, Stewart M, Zelevinsky K. Nurse-staffing levels and the quality of care in hospitals. N Engl J Med. 2002;346(22):1715–22.

69. Trinkoff AM, Johantgen M, Storr CL, Gurses AP, Liang Y, Han K. Nurses' work schedule characteristics, nurse staffing, and patient mortality. Nurs Res. 2011;60(1):1–8.

For peer review only - http://bmjopen.bmj.com/site/about/guidelines.xhtml

70. Lichtig LK, Knauf RA, Milholland DK. Some impacts of nursing on acute care hospital outcomes. JONA J Nurs Adm. 1999;29(2):25–33.

71. Kouatly IA, Nassar N, Nizam M, Badr LK. Evidence on Nurse Staffing Ratios and Patient Outcomes in a Low-Income Country: Implications for Future Research and Practice. Worldviews Evid Based Nurs. 2018;15(5):353–60.

72. Kalisch BJ, Doumit M, Lee KH, Zein JE. Missed nursing care, level of staffing, and job satisfaction: Lebanon versus the United States. J Nurs Adm. 2013;43(5):274–9.

73. Yu D, Ma Y, Sun Q, Lu G, Xu P. A nursing care classification system for assessing workload and determining optimal nurse staffing in a teaching hospital in China: A pre-post intervention study. Int J Nurs Pract. 2015;21(4):339–49.

74. Zhu X wen, You L ming, Zheng J, Liu K, Fang J bo, Hou S xiao, et al. Nurse staffing levels make a difference on patient outcomes: a multisite study in Chinese hospitals. J Nurs Scholarsh. 2012;44(3):266–73.

75. Sasichay-Akkadechanunt T, Scalzi CC, Jawad AF. The relationship between nurse staffing and patient outcomes. JONA J Nurs Adm. 2003;33(9):478–85.

76. Feleke SA, Mulatu MA, Yesmaw YS. Medication administration error: magnitude and associated factors among nurses in Ethiopia. BMC Nurs. 2015;14(1):1–8.

77. Ball JE, Bruyneel L, Aiken LH, Sermeus W, Sloane DM, Rafferty AM, et al. Post-operative mortality, missed care and nurse staffing in nine countries: a cross-sectional study. Int J Nurs Stud. 2018;78:10–5.

78. English M, Irimu G, Akech S, Aluvaala J, Ogero M, Isaaka L, et al. Employing learning health system principles to advance research on severe neonatal and paediatric illness in Kenya. BMJ Glob Health. 2021;6(3):e005300.

79. Ogero M, Ayieko P, Boniface Makone TJ, Malla L, Oliwa J, Irimu G, et al. An observational study of monitoring of vital signs in children admitted to Kenyan hospitals: an insight into the quality of nursing care? J Glob Health. 2018;8(1).

For peer review only

For peer review only - http://bmjopen.bmj.com/site/about/guidelines.xhtml

For peer review only

For peer review only - http://bmjopen.bmj.com/site/about/guidelines.xhtml

For peer review only

For peer review only - http://bmjopen.bmj.com/site/about/guidelines.xhtml

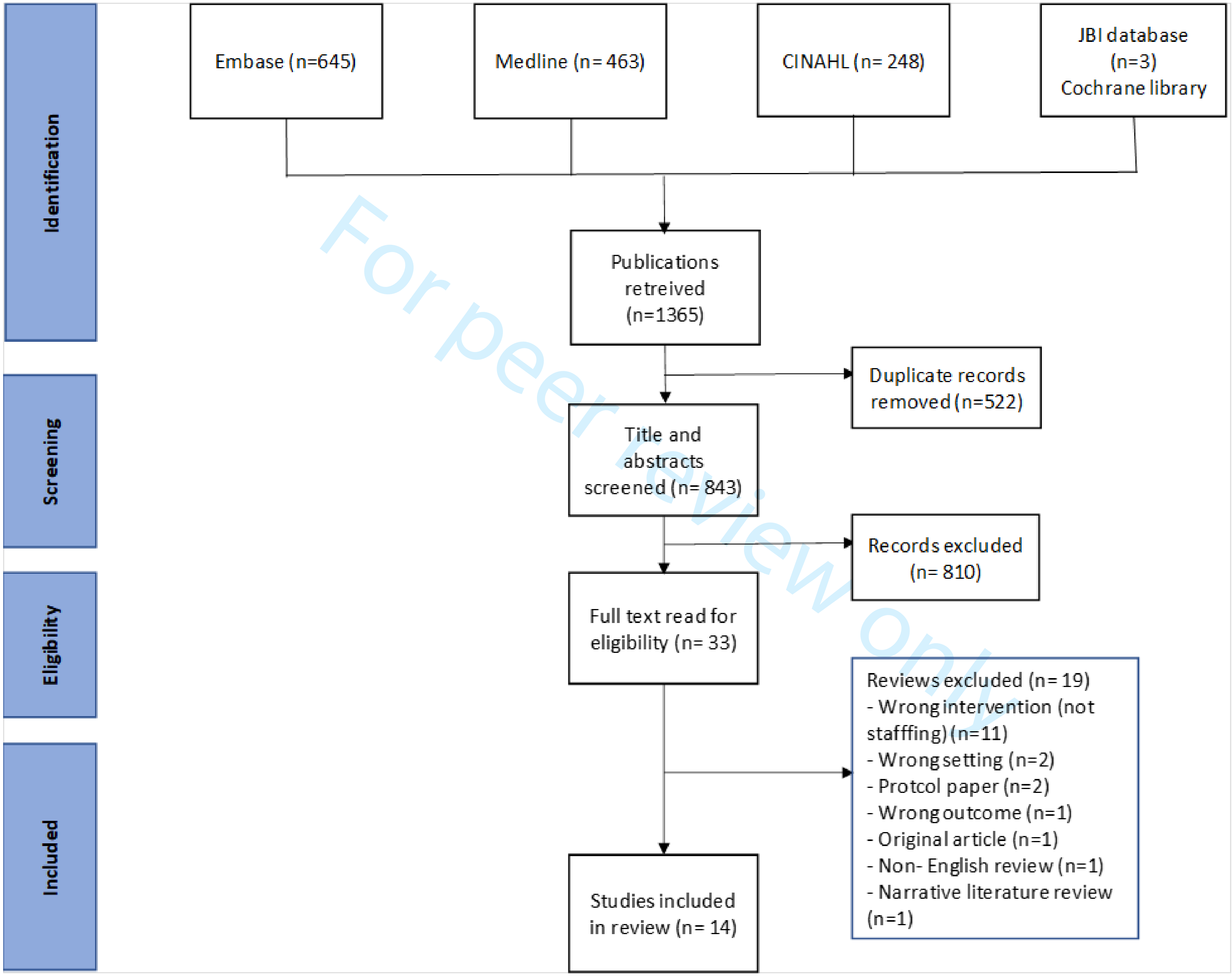

For peer review only

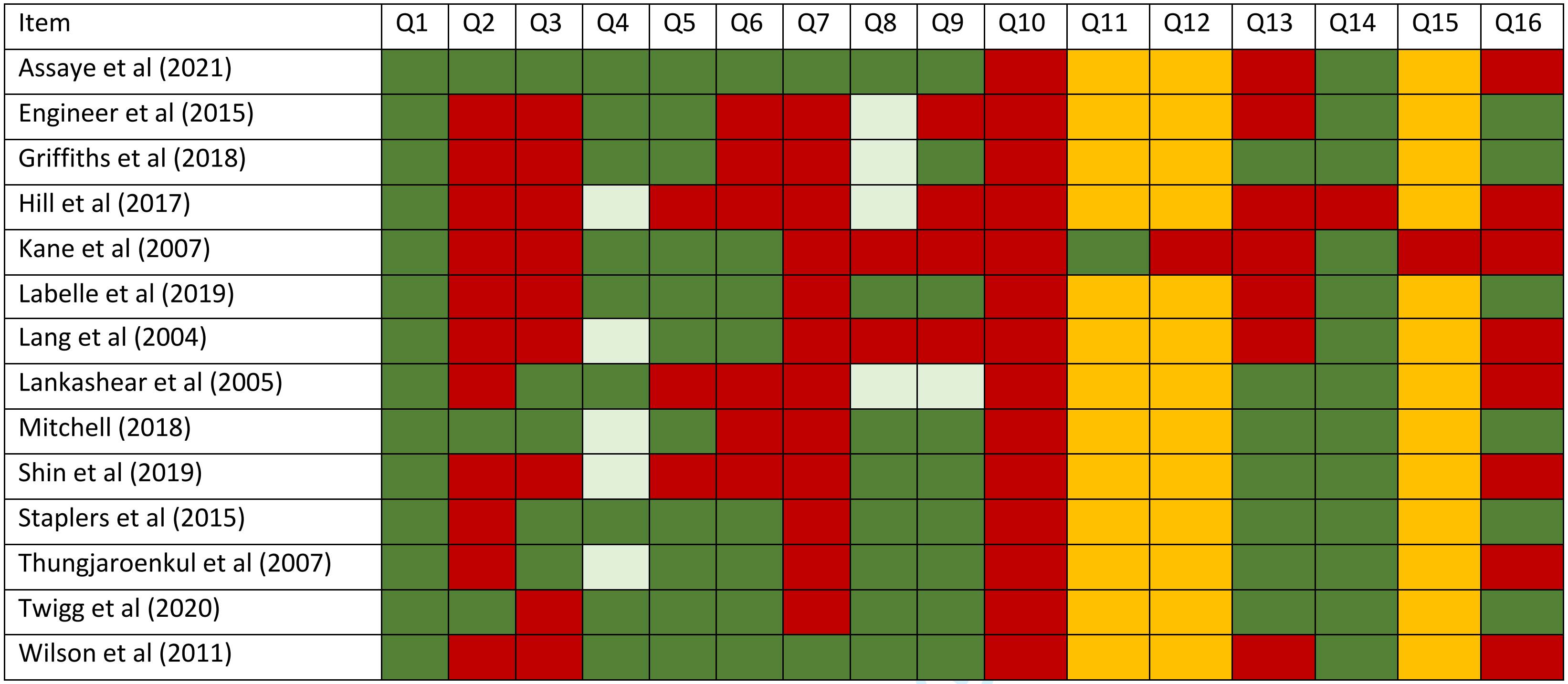

Figure 2 – Risk of Bias assessment using the AMSTAR-2 checklist. (Key: Red – No, Green – Yes, lighter green – Partially Yes, Yellow – Not applicable)

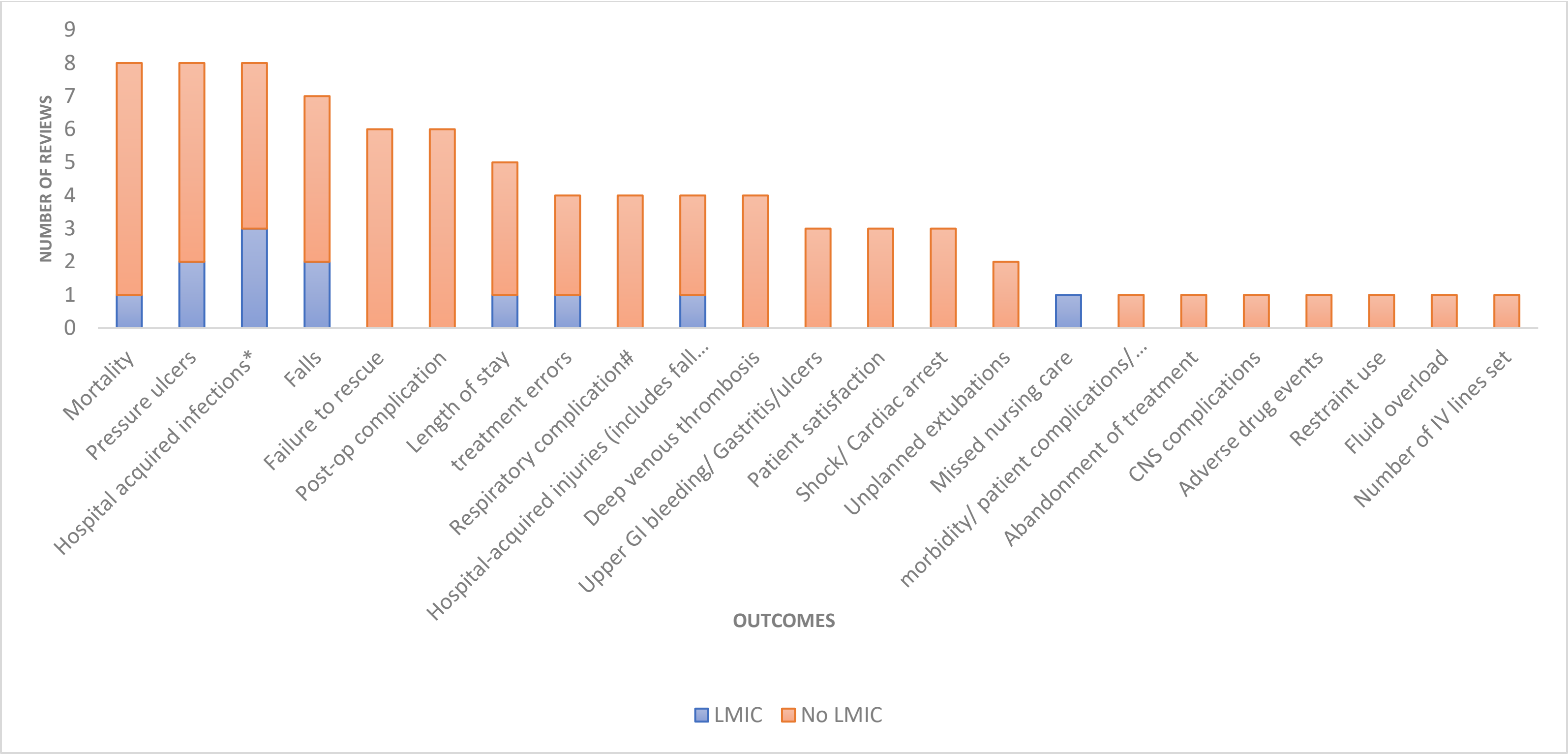

Figure 3: Stacked bar chart showing the range of patient care outcomes reported across reviews and the number of reviews which report this in a LMIC study (Blue- number of reviews reporting the outcome from a LMIC study, Orange – number of reviews reporting the outcome from only non-LMIC studies)

*- Hospital-acquired infections include pneumonia, UTI, central line-associated blood stream infections, nosocomial infections, wound infections

# - respiratory complications include pulmonary compromise/ respiratory failure/ pulmonary embolism)

The least reported patient care outcomes were missed nursing care, patient adverse events, abandonment of treatment, central nervous system complications, adverse drug events, restraint use, fluid overload and the number of intravenous cannulations which were all reported by 1 systematic review each.

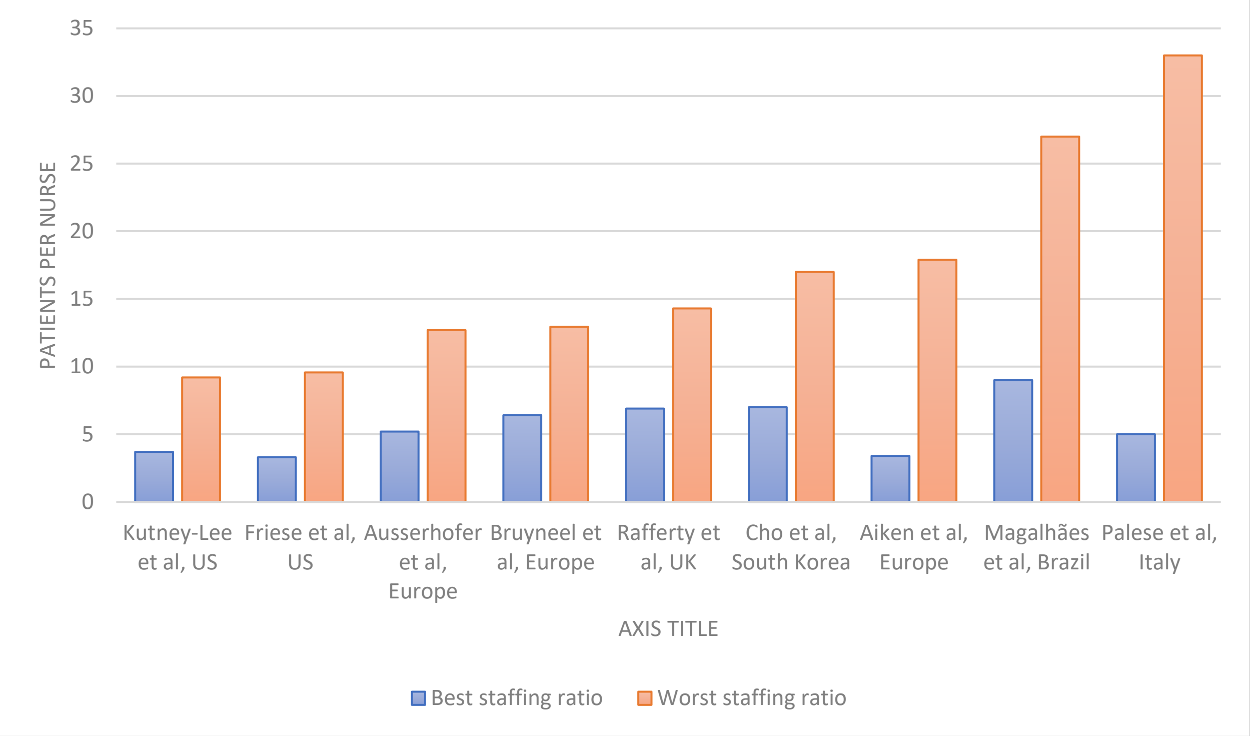

Figure 4 –Bar chart showing the patient to nurse ratio per shift range described in individual studies that reported this metric and described a range (countries of origin and first author of the individual studies are presented, and the Y-axis represents the number of patients per nurse).

The best staffing ratio in the study context is presented in blue while the worst is presented in orange, these best and worst ratios were used in study-specific exploratory of the association between staffing and nursing quality.

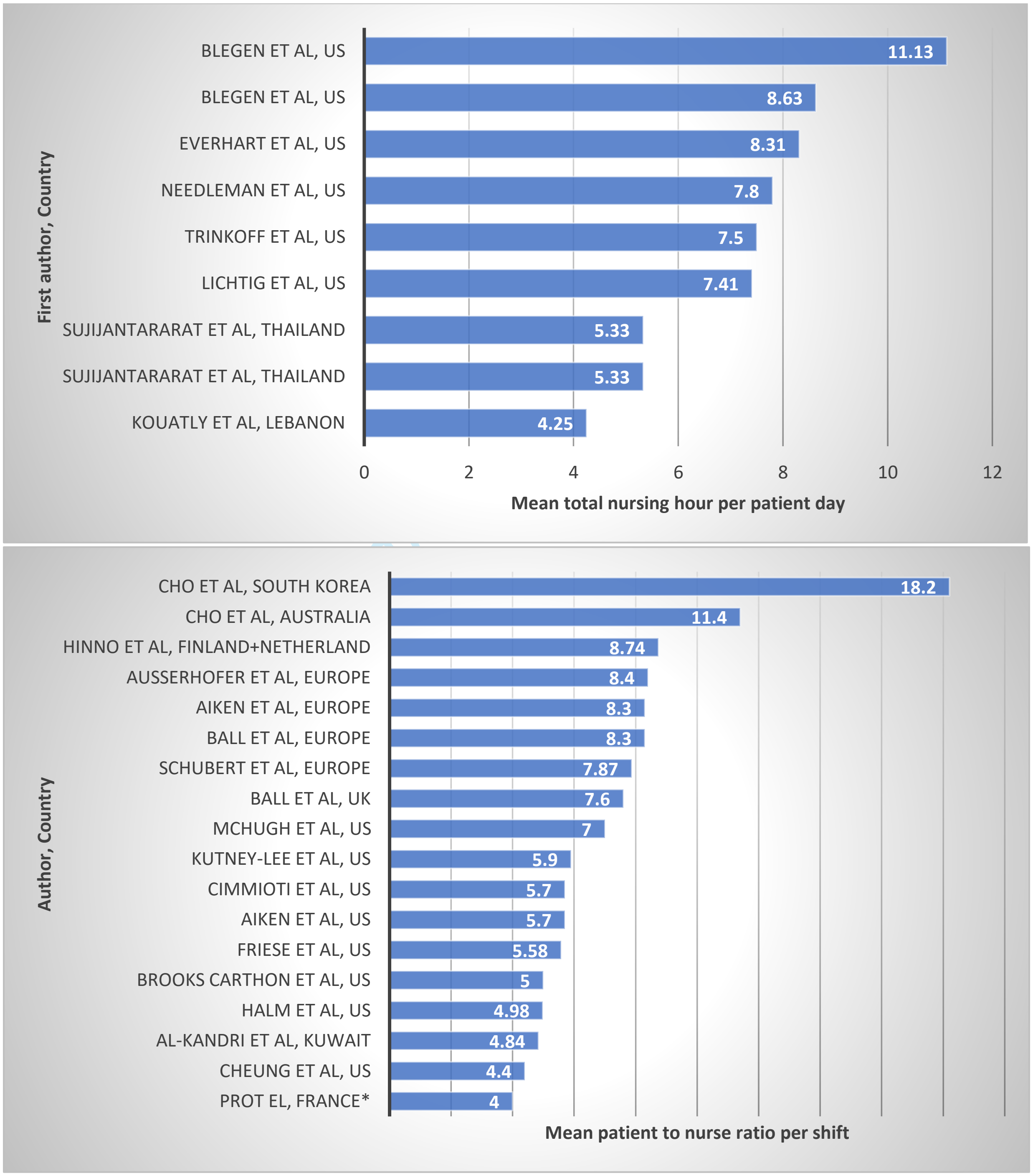

Figure 5 – Horizontal bar charts showing the mean total nursing hour per patient day (top) and the mean patient to nurse ratios (bottom) across primary studies reporting these metrics. (First author and countries in which individual studies were conducted are depicted on the Y-axis).

*- median value

Online supplemental file 1 : Medline and Embase search strategy

1.	Nursing Staff, Hospital/
2.	Nurse's Role/
3.	Specialties, Nursing/
4.	Nurse-Patient Relations/
5.	((nurse* or nursing) adj3 (staff* or ratio or ratios or workforce or workload or sensitive or mix or practitioner* or assistant* or practice* or performance)).ab,kw,ti.
6.	1 or 2 or 3 or 4 or 5
7.	"Personnel Staffing and Scheduling"/
8.	Health Care Rationing/
9.	"Quality of Health Care"/
10.	Quality Indicators, Health Care/
11.	Patient Safety/
12.	Outcome Assessment, Health Care/
13.	(outcome* or impact* or safety or quality or evidence or indicator* or advers* or mortality or death* or omission* or missed or missing or unmet or undone or rationed or rationing).ti.
14.	"missed nursing".ab,kw,ti.
15.	"missed care".ab,kw,ti.
16.	7 or 8 or 9 or 10 or 11 or 12 or 13 or 14 or 15
17.	(systematic and review).ti.
18.	("meta analysis" or metaanalysis).ti.
19.	"Systematic Review"/

20.	Meta-Analysis/
21.	17 or 18 or 19 or 20
22.	6 and 16 and 21

Search strategy in CINAHL

S1	MH "NURSING STAFF, HOSPITAL"/ OR MH "NURSE'S ROLE"/ OR MH "SPECIALTIES, NURSING"/ OR MH "NURSE-PATIENT RELATIONS"/ OR (TI "nurse*" N3 "STAFF*") OR (AB "nurse*" N3 "STAFF*") OR (TI "nurse*" N3 "RATIO") OR (AB "nurse*" N3 "RATIO") OR (TI "nurse*" N3 "RATIOS") OR (AB "nurse*" N3 "RATIOS") OR (TI "nurse*" N3 "WORKFORCE") OR (AB "nurse*" N3 "WORKFORCE") OR (TI "nurse*" N3 "WORKLOAD") OR (AB "nurse*" N3 "WORKLOAD") OR (TI "nurse*" N3 "SENSITIVE") OR (AB "nurse*" N3 "SENSITIVE") OR (TI "nurse*" N3 "MIX") OR (AB "nurse*" N3 "MIX") OR (TI "nurse*" N3 "PRACTITIONER*") OR (AB "nurse*" N3 "PRACTITIONER*") OR (TI "nurse*" N3 "ASSISTANT*") OR (AB "nurse*" N3 "ASSISTANT*") OR (TI "nurse*" N3 "PRACTICE*") OR (AB "nurse*" N3 "PRACTICE*") OR (TI "nurse*" N3 "PERFORMANCE") OR (AB "nurse*" N3 "PERFORMANCE") OR (TI "nursing" N3 "STAFF*") OR (AB "nursing" N3 "STAFF*") OR (TI "nursing" N3 "RATIO") OR (AB "nursing" N3 "RATIO") OR (TI "nursing" N3 "RATIOS") OR (AB "nursing" N3 "RATIOS") OR (TI "nursing" N3 "WORKFORCE") OR (AB "nursing" N3 "WORKFORCE") OR (TI "nursing" N3 "WORKLOAD") OR (AB "nursing" N3 "WORKLOAD") OR (TI "nursing" N3 "SENSITIVE") OR (AB "nursing" N3 "SENSITIVE") OR (TI "nursing" N3 "MIX") OR (AB "nursing" N3 "MIX") OR (TI "nursing" N3 "PRACTITIONER*") OR (AB "nursing" N3 "PRACTITIONER*") OR (TI "nursing" N3 "ASSISTANT*") OR (AB "nursing" N3 "ASSISTANT*") OR (TI "nursing" N3 "PRACTICE*") OR (AB "nursing" N3 "PRACTICE*") OR (TI "nursing" N3 "PERFORMANCE") OR (AB "nursing" N3 "PERFORMANCE")
S2	MH "Personnel Staffing and Scheduling"/ OR MH "Health Care Rationing"/ OR MH "Quality of Health Care"/ OR MH "Quality Indicators, Health Care"/ OR MH "Patient Safety"/ OR MH "Outcome Assessment, Health Care"/ OR TI(outcome* OR impact* OR safety OR quality OR evidence OR indicator* OR advers* OR mortality OR death* OR omission* OR missed OR missing OR unmet OR undone OR rationed OR rationing) OR TI ("missed nursing") OR AB ("missed nursing") OR TI ("missed care") OR AB ("missed care")
S3	TI (systematic AND review) OR TI ("meta analysis" OR metaanalysis) OR MH "Systematic Review"/ OR MH "Meta-Analysis/")
S4	S1 AND S2 AND S3

For peer review only

Online supplemental file 2 – List of Excluded systematic reviews after full text screening

First Author	Year	Citation	Reason for exclusion	Explanation
Bae et al	2014	Bae SH, Fabry D. Assessing the relationships between nurse work hours/overtime and nurse and patient outcomes: systematic literature review. Nursing outlook. 2014 Mar 1;62(2):138-56.	Wrong intervention	Intervention was nurse working hours which is not a metric of nurse staffing level
Butler et al	2019	Butler M, Schultz TJ, Halligan P, Sheridan A, Kinsman L, Rotter T, Beaumier J, Kelly RG, Drennan J. Hospital nurse-staffing models and patient-and staff-related outcomes. Cochrane Database of Systematic Reviews. 2019(4).	Wrong intervention	Intervention was nurse staffing models not ratio change or change in other metrics of nurse staffing
Wilson et al	2010	Wilson S, Bremner A, Hauck Y. Association between nurse staffing and hospitalised children's health outcomes: A systematic review. JBI Evidence Synthesis. 2010 Jan 1;8(8):1-5.	Protocol paper	
Recio-Saucedo et al	2018	Recio-Saucedo A, Dall'Ora C, Maruotti A, Ball J, Briggs J, Meredith P, Redfern OC, Kovacs C, Prytherch D, Smith GB, Griffiths P. What impact does nursing care left undone have on patient outcomes? Review of the literature. Journal of clinical nursing. 2018 Jun;27(11-12):2248-59.	Wrong intervention	Impact of missed nursing care on patient care outcomes and not the impact of staffing
Shekelle et al	2013	Shekelle PG. Nurse–patient ratios as a patient safety strategy:	Literature review	Narrative literature review

For peer review only

		a systematic review. Annals of internal medicine. 2013 Mar 5;158(5_Part_2):404-9.		
Papastavrou et al	2014	Papastavrou E, Andreou P, Efstathiou G. Rationing of nursing care and nurse–patient outcomes: a systematic review of quantitative studies. The International journal of health planning and management. 2014 Jan;29(1):3-25.	Wrong intervention	Impact of missed nursing care on patient care outcomes and not the impact of nurse staffing
Driscoll et al	2018	Driscoll A, Grant MJ, Carroll D, Dalton S, Deaton C, Jones I, Lehwaldt D, McKee G, Munyombwe T, Astin F. The effect of nurse-to-patient ratios on nurse-sensitive patient outcomes in acute specialist units: a systematic review and meta-analysis. European Journal of Cardiovascular Nursing. 2018 Jan 1;17(1):6-22.	Wrong study setting (non-ward setting)	All papers from intensive care unit
Garcia et al	2010	García F, Sampietro-Colom L. Nursing staff and patient results: systematic review about the existing relationship. Revista de enfermeria (Barcelona, Spain). 2010 Jan 1;33(1):14-26.	Non-English paper	Spanish
Al-ghraiybah et al	2021	Al-ghraiybah T, Sim J, Lago L. The relationship between the nursing practice environment and five nursing-sensitive patient outcomes in acute care hospitals: A systematic review. Nursing Open. 2021 Mar 4.	Wrong intervention	Intervention is nurse practice environment and not nurse staffing

For peer review only

dit Dariel et al	2015	dit Dariel OP, Regnaux JP. Do Magnet®-accredited hospitals show improvements in nurse and patient outcomes compared to non-Magnet hospitals: a systematic review. JBI Evidence Synthesis. 2015 Jun 1;13(6):168-219.	Wrong intervention	Intervention is magnet accreditation and not nurse staffing
Kazanijan et al	2005	Kazanjian A, Green C, Wong J, Reid R. Effect of the hospital nursing environment on patient mortality: a systematic review. Journal of Health Services Research & Policy. 2005 Apr 1;10(2):111-7A.	Wrong intervention	Intervention is nurse practice environment and not nurse staffing
Johnston et al	2015	Johnston MJ, Arora S, King D, Bouras G, Almoudaris AM, Davis R, Darzi A. A systematic review to identify the factors that affect failure to rescue and escalation of care in surgery. Surgery. 2015 Apr 1;157(4):752-63.	Wrong intervention	Focused on factors that affect failure to rescue and not nurse staffing
Kushemererwa et al	2020	Kushemererwa D, Davis J, Moyo N, Gilbert S, Gray R. The Association between Nursing Skill Mix and Mortality for Adult Medical and Surgical Patients: Protocol for a Systematic Review. International Journal of Environmental Research and Public Health. 2020 Jan;17(22):8604.	Protocol paper	Protocol paper and not original article
Olley et al	2018	Olley R, Edwards I, Avery M, Cooper H. Systematic review of the evidence related to mandated nurse staffing ratios in	Wrong outcome	Outcome is on general evidence and not patient care outcomes

For peer review only

		acute hospitals. Australian Health Review. 2018 Apr 17;43(3):288-93.		
Copanitsanou et al	2017	Copanitsanou P, Fotos N, Brokalaki H. Effects of work environment on patient and nurse outcomes. British Journal of Nursing. 2017 Feb 9;26(3):172-6.	original paper	Original paper and not a systematic review
Myers et al	2018	Myers H, Pugh JD, Twigg DE. Identifying nurse-sensitive indicators for stand-alone high acuity areas: A systematic review. Collegian. 2018 Aug 1;25(4):447-56.	Wrong study setting (non-ward setting)	Intensive care unit
Bae et al	2021	Bae SH. Relationships between comprehensive characteristics of nurse work schedules and adverse patient outcomes: A systematic literature review. Journal of Clinical Nursing. 2021 Feb 22.	Wrong intervention	Intervention is nurses' working schedule and not nurse staffing
Butler et al	2011	Butler M, Collins R, Drennan J, Halligan P, O'Mathuna D, Schultz T, Sheridan A, Vilis E. Hospital nurse staffing models and patient and staff-related outcomes-an epic EPOC systematic review. JBI Evidence Implementation. 2011 Sep 1;9(3):326.	Wrong intervention	Intervention is nurse staffing models not a change in nurse staffing numbers
Bae et al	2011	BAE SH. Assessing the relationships between nurse working conditions and patient outcomes: systematic literature review. Journal of nursing management. 2011 Sep;19(6):700-13.	Wrong intervention	Intervention is nurse working conditions and not nurse staffing

For peer review only

For peer review only

Online supplemental file 3: Excluded primary studies and reasons for exclusion.

systematic review	Title	reason for exclusion
Griffith et al		
	Zander, B., Dobler, L., Bäumler, M. and Busse, R., 2014. Implicit rationing of care services in German acute care hospitals – Results of the international nursing study Rn4cast. Health Care, 76(11), pp.727-734.	German
Assaye et al		
	Soares M, Bozza FA, Angus DC, Japiassu AM, Viana WN, Costa R, et al. Organizational characteristics, outcomes, and resource use in 78 Brazilian intensive care units: the ORCHESTRA study. Intensive Care Med 2015;41(12):2149– 60.	ICU setting
	Silva MCMd, Sousa RMCd, Padilha KG. Factors associated with death and readmission into the Intensive Care Unit. Rev Lat Am Enfermagem 2011;19:911–9.	ICU setting
	Chittawatanarat K, Sataworn D, Thongchai C. Thai Society of Critical Care Medicine Study G. Effects of ICU characters, human resources and workload to outcome indicators in Thai ICUs: the results of ICU-RESOURCE I study. J Med Assoc Thai 2014;97(Suppl 1):S22–30.	ICU setting
	Cremasco MF, Wenzel F, Zanei SSV, Whitaker IY. Pressure ulcers in the intensive care unit: the relationship between nursing workload, illness severity and pressure ulcer risk. J Clin Nurs 2013;22(15/16):2183–91.	ICU setting
	Al Tehewy M, Fahim H, Gad NI, El Gafary M, Rahman SA. Medication administration errors in a university hospital. J Patient Saf 2016;12(1):34–9.	Wrong intervention (number and type of shift)

For peer review only

	Daud-Gallotti RM, Costa SF, Guimara˜es T, Padilha KG, Inoue EN, Vasconcelos TN, et al. Nursing workload as a risk factor for health care associated infections in ICU: a prospective study. PLoS One 2012;7(12):e52342.	ICU setting
	Celen MK, Tamam Y, Hosoglu S, Ayaz C, Geyik MF, Apak I. Multiresistant bacterial colonization due to increased nurse workload in a neurology intensive care unit. Neurosciences 2006;11(4):265–70.	ICU setting
	Aycan IO, Celen MK, Yilmaz A, Almaz MS, Dal T, Celik Y, et al. Bacterial colonization due to increased nurse workload in an intensive care unit. Rev Brasil Anestesiol 2015;65:180–5	ICU setting
	. Cremasco MF, Wenzel F, Zanei SSV, Whitaker IY. Pressure ulcers in the intensive care unit: the relationship between nursing workload, illness severity and pressure ulcer risk. J Clin Nurs 2013;22(15/16):2183–91	ICU setting
	Bjorklund de Lima L, Rejane Rabelo E. Nursing workload in the post-anesthesia care unit. Acta Paul Enferm 2013;26(2): 116–22	Wrong intervention (Nursing workload measure)
	Day SW. Evaluating the impact of the Guatemalan Nursing Program on staff, organizational, and clinical outcomes. University of Tennessee Health Science Center; 2010; p. 93	Wrong intervention (Addition of a nurse educator)
	Ilhan MN, Durukan E, Taner E, Maral I, Bumin MA. Burnout and its correlates among nursing staff: questionnaire survey. J Adv Nurs 2008;61(1):100–6	Nurse outcome (Burn out)
	Karakoc A, Yilmaz M, Alcalar N, Esen B, Kayabasi H, Sit D. Burnout syndrome among hemodialysis and peritoneal dialysis nurses. Iran J Kidney Dis 2016;10(6):395–404.	Nurse outcome (Burn out)
	Roomaney R, Steenkamp J, Kagee A. Predictors of burnout among HIV	Nurse outcome (Burn out)

For peer review only

	nurses in the Western Cape. Curationis 2017;40(1):1–9	
	Kunaviktikul W, Wichaikhum O, Nantsupawat A, Nantsupawat R, Chontawan R, Klunklin A et al. Nurses' extended work hours: patient, nurse and organizational outcomes. Int Nurs Rev 2015;62(3):386–93.	Wrong intervention (Nursing workload measure)
	De Paiva LC, Gomes Cana´rio AC, Corsino de Paiva China EL, Gonc¸alves AK. Burnout syndrome in health-care professionals in a university hospital. Clinics 2017;72(5):305–9.	Nurse outcome (Burn out)
	Lu MM, Ruan H, Xing WJ, Hu Y. Nurse burnout in China: a questionnaire survey on staffing, job satisfaction, and quality of care. J Nurs Manag 2015;23(4):4	Nurse outcome (Burn out)
	Mohammadpoorasl A, Maleki A, Sahebihagh MH. Prevalence of professional burnout and its related factors among nurses in Tabriz in 2010. IJNMR 2012;17(7):524–9	Nurse outcome (Burn out)
	Naz S, Hashmi AM, Asif A. Burnout and quality of life in nurses of a tertiary care hospital in Pakistan. J Pak Med Assoc 2016;66(5):532–6	Nurse outcome (Burn out)
	. Negi Y, Bagga R. Burnout among nursing professionals in tertiary care hospitals of Delhi. J Health Manag 2015;17(2):163–77	Nurse outcome (Burn out)
	Nantsupawat A, Srisuphan W, Kunaviktikul W, Wichaikhum, Aungsuroch Y, Aiken LH. Impact of nurse work environment and staffing on hospital nurse and quality of care in Thailand. J Nurs Scholarsh 2011;43(4):426–32	Nurse outcome (Burn out)
	Nantsupawat A, Nantsupawat R, Kulnaviktikul W, McHugh MD. Relationship between nurse staffing levels and nurse outcomes in community hospitals, Thailand. Nurs Health Sci 2015;17(1):112–8	Nurse outcome (Emotional exhaustion and Needle stick injuries)

For peer review only

Thungjaroenkul P, Cummings GG, Embleton A. The impact of nurse staffing on hospital costs and patient length of stay: a systematic review. Nursing Economics. 2007 Sep 1;25(5):255.		
	Amaravadi, R.K., Dimick, J.B., Pronovost, P.J., & Lipsett, P.A. (2000). ICU nurseto-patient ratio is associated with complications and resource use after esophagectomy. Intensive Care Medicine, 26, 1857-1862.	ICU setting
	Pronovost PJ, Jenckes MW, Dorman T, Garrett E, Breslow MJ, Rosenfeld BA, Lipsett PA, Bass E. Organizational characteristics of intensive care units related to outcomes of abdominal aortic surgery. Jama. 1999 Apr 14;281(14):1310-7.	ICU setting
	Lassnigg A, Hiesmayr M, Bauer P, Haisjackl M. Effect of centre-, patient-and procedure-related factors on intensive care resource utilisation after cardiac surgery. Intensive care medicine. 2002 Oct;28(10):1453-61.	ICU setting
	Dimick, Justin B., et al. "Effect of nurse-to-patient ratio in the intensive care unit on pulmonary complications and resource use after hepatectomy." American Journal of Critical Care 10.6 (2001): 376.	ICU setting

For peer review only

	Bloom JR, Alexander JA, Nuchols BA. Nurse staffing patterns and hospital efficiency in the United States. Social science & medicine. 1997 Jan 1;44(2):147-55.	Wrong outcome (cost of care)
	Lee TY, Yeh ML, Chen HH, Lien GH. The skill mix practice model for nursing: measuring outcome. Journal of advanced nursing. 2005 Aug;51(4):406-13.	Wrong outcome (cost of care)
	Hall LM, Doran D, Pink GH. Nurse staffing models, nursing hours, and patient safety outcomes. JONA: The Journal of Nursing Administration. 2004 Jan 1;34(1):41-5.	Wrong outcome (cost of care)
	Pratt RA, Burr G, Leelarthaepin B, Blizard P, Walsh S. The effects of All-RN and RN-EN staffing on the quality and cost of patient care. The Australian journal of advanced nursing: a quarterly publication of the Royal Australian Nursing Federation. 1993 Mar 1;10(3):27-39.	Wrong intervention (skill mix)
	Newhouse RP, Johantgen M, Pronovist PJ, Johnson E. Perioperative nurses and patient outcomes: mortality, complications, and length of stay. The Journal of nursing administration. 2010 Oct 1;40(10 Suppl):S54-67.	Wrong intervention (skill mix)
	Needleman J, Buerhaus PI, Stewart M, Zelevinsky K, Mattke S. Nurse staffing in hospitals: is there a business case for quality?. Health Affairs. 2006 Jan;25(1):204-11.	Wrong intervention (costs)
	Barkell NP, Killinger KA, Schultz SD. The relationship between nurse staffing models and patient outcomes: a descriptive study. Outcomes Management. 2002 Jan 1;6(1):27-33.	Wrong intervention (skill mix)
	McCue M, Mark BA, Harless DW. Nurse staffing, quality, and financial performance. Journal of health care finance. 2003 Jan 1;29(4):54-76.	Wrong outcome (cost of care)

For peer review only

Twigg DE, Whitehead L, Doleman G, El-Zaemey S. The impact of nurse staffing methodologies on nurse and patient outcomes: A systematic review. Journal of Advanced Nursing. 2021 Jun 3.		
	Bowblis, J. R., & Ghattas, A. (2017). The impact of minimum quality standard regulations on nursing home staffing, quality, and exit decisions. Review of Industrial Organization, 50(1), 43–68. https://doi. org/10.1007/s11151-016-9528-x	nursing home
	Chan, T. C., Killeen, J. P., Vilke, G. M., Marshall, J. B., & Castillo, E. M. (2010). Effect of mandated nurse–patient ratios on patient wait time and care time in the emergency department. Academic Emergency Medicine, 17(5), 545–552. https://doi. org/10.1111/j.1553-2712.2010.00727.x	Emergency department
	Chen, M. M., & Grabowski, D. C. (2015). Intended and unintended consequences of minimum staffing standards for nursing homes. Health Economics, 24(7), 822–839. https://doi.org/10.1002/hec.3063	nursing home
	Cox, K. S., Anderson, S. C., Teasley, S. L., Sexton, K. A., & Carroll, C. A. (2005). Nurses' work environment perceptions when employed in states with and without mandatory staffing ratios and/or mandatory staffing plans. Policy, Politics, &	Nurse outcomes

For peer review only

	Nursing Practice, 6(3), 191–197. https://doi.org/10.1177/1527154405279091	
	Hodgson, A., Morgan, D., & Peterson, R. (2016). Does better nurse staffing improve detection of depression and anxiety as secondary conditions in hospitalized patients with pneumonia? Nursing Economics, 34(3), 134	Mental health outcome
	Law, A. C., Stevens, J. P., Hohmann, S., & Walkey, A. J. (2018). Patient outcomes after the introduction of statewide intensive care unit nurse staffing regulations. Critical Care Medicine, 46(10), 1563.	ICU setting
	Leigh, J. P., Markis, C. A., Iosif, A.-M., & Romano, P. S. (2015). California's nurse-to-patient ratio law and occupational injury. International Archives of Occupational and Environmental Health, 88(4), 477–484.	Nurse outcome
	Park, J., & Stearns, S. C. (2009). Effects of state minimum staffing standards on nursing home staffing and quality of care. Health Services Research, 44(1), 56–78. https://doi.org/10.1111/j.1475-6773.2008.00906.x	nursing home
	Spetz J, Herrera C. Changes in nurse satisfaction in California, 2004 to 2008. Journal of Nursing Management. 2010 Jul;18(5):564-72.	nurse outcome
	Spetz, J. (2008). Nurse satisfaction and the implementation of minimum nurse staffing regulations. Policy, Politics, & Nursing Practice, 9(1), 15–21. https://doi.org/10.1177/1527154408316950	Nurse outcome
	Tellez, M. (2012). Work satisfaction among California registered nurses: A longitudinal comparative analysis. Nursing economic$, 30(2), 73–81	Nurse outcome

For peer review only

	Tellez, M., & Seago, J. A. (2013). California nurse staffing law and RN workforce changes. Nursing Economics, 31(1), 18	Nurse outcome
	Matsudaira, J. D. (2014). Government regulation and the quality of healthcare evidence from minimum staffing legislation for nursing homes. Journal of Human Resources, 49(1), 32–72. https://doi.org/10.1353/jhr.2014.0003	nursing home
	Weichenthal, L., & Hendey, G. W. (2011). The effect of mandatory nurse ratios on patient care in an emergency department. The Journal of Emergency Medicine, 40(1), 76–81. https://doi.org/10.1016/j.jemermed.2009.02.037	Emergency department
	Zhang, X., & Grabowski, D. C. (2004). Nursing home staffing and quality under the nursing home reform act. The Gerontologist, 44(1), 13–23. https://doi.org/10.1093/geront/44.1.13	nursing home
Shin et al 2019		
	UK Neonatal Staffing Study Group. Patient volume, staffing, and workload in relation to risk-adjusted outcomes in a random stratified sample of UK neonatal intensive care units: a prospective evaluation. The Lancet. 2002 Jan 12;359(9301):99-107.	ICU setting
	Brooks-Carthon JM, Kutney-Lee A, Sloane DM, Cimiotti JP, Aiken LH. Quality of care and patient satisfaction in hospitals with high concentrations of black patients. Journal of Nursing Scholarship. 2011 Sep;43(3):301-10.	wrong intervention (practice environment)
Lang 2004		
	Kustaborder MJ, Rigney M. Interventions for safety.	wrong intervention (nurse staffing mix)

For peer review only

	Bostrom J, Zimmerman J. Restructuring nursing for a competitive health care environment. Nursing economic$. 1993 Jan 1;11(1):35-41.	wrong intervention (nurse restructuring/ skill mix)
	Needleman J, Buerhaus P. Nurse staffing and patient safety: current knowledge and implications for action. International Journal for Quality in Health Care. 2003 Aug 1;15(4):275-7.	editorial
	Behner KG, Fogg LF, Fournier LC, Frankenbach JT, Robertson SB. Nursing resource management: analyzing the relationship between costs and quality in staffing decisions. Health Care Management Review. 1990 Jan 1;15(4):63-71.	wrong intervention (labour efficiency)
	Shukla RK. All-RN model of nursing care delivery: A cost-benefit evaluation. Inquiry. 1983 Jul 1:173-84.	wrong intervention
	Arndt M, Crane S. Influences on nursing care volume. Journal of the Society for Health Systems. 1998 Jan 1;5(4):38-49.	nurse outcome
	Hinshaw AS, Scofield R, Atwood JR. Staff, patient, and cost outcomes of all-registered nurse staffing. JONA: The Journal of Nursing Administration. 1981 Nov 1;11(11):30-6.	Wrong intervention (skill mix)
	Grillo-Peck AM, Risner PB. The effect of a partnership model on quality and length of stay. Nursing economic$. 1995 Nov 1;13(6):367-72.	wrong intervention (nurse partnership model)
	Ceria CD. Nursing absenteeism and its effects on the quality of patient care. The Journal of nursing administration. 1992 Dec;22(12):11-38.	wrong intervention (absenteeism)
	Bloom JR, Alexander JA, Nuchols BA. The effect of the social organization of work on the voluntary turnover rate of hospital nurses in the United	nurse outcome

For peer review only

	States. Soc Sci Med. 1992;34:1413-1424.	
	Lanza ML, Kayne HL, Gulliford D, et al. Staffing of inpatient psychiatric units and assault by patients. J Am Psychiatr Nurses Assoc. 1997;3:42-4	nurse outcome
	Shortell SM, Hughes EF. The effects of regulation, competition, and ownership on mortality rates among hospital inpatients. New England Journal of Medicine. 1988 Apr 28;318(17):1100-7.	wrong intervention
	Clarke SP, Sloane DM, Aiken LH. Effects of hospital staffing and organizational climate on needlestick injuries to nurses. American journal of public health. 2002 Jul;92(7):1115-9.	nurse outcome
	GLANDON GL, COLBERT KW, Thomasma M. Nursing delivery models and RN mix: cost implications. Nursing Management. 1989 May 1;20(5):30-3.	nurse outcome
	Halloran EJ. RN staffing: more care—less cost. Nursing Management. 1983 Sep 1;14(9):18-23.	nurse outcome
	Osinski EG, Powals JG. The cost of all RN staffed primary nursing. Supervisor nurse. 1980 Jan;11(1):16-21.	nurse outcome
	Bradbury RC, Golec JH, Steen PM. Relating hospital health outcomes and resource expenditures. Inquiry. 1994 Apr 1:56-65.	Wrong intervention (nurse skill mix)
	Krakauer H, Bailey RC, Skellan KJ, Stewart JD, Hartz AJ, Kuhn EM, Rimm AA. Evaluation of the HCFA model for the analysis of mortality following hospitalization. Health services research. 1992 Aug;27(3):317.	Wrong intervention (nurse skill mix)
	Kuhn EM, Hartz A, Gottlieb MS, Rimm AA. The relationship of hospital characteristics and the results of peer review in six large	nurse outcome

For peer review only

	states. Med Care. 1991;29:1028-1038.	
Kane 2007		
	Fridkin SK, Pear SM, Williamson TH, Galgiani JN, Jarvis WR. The role of understaffing in central venous catheter-associated bloodstream infection. Infection Control & Hospital Epidemiology. 1996 Mar;17(3):150-8.	ICU setting
	Amaravadi RK, Dimick JB, Pronovost PJ, et al. ICU nurse-to-patient ratio is associated with complications and resource use after esopha- gectomy. Intensive Care Med. 2000;26: 18	ICU setting
	Dang D, Johantgen ME, Pronovost PJ, et al. Postoperative complica-tions: does intensive care unit staff nursing make a difference? Heart Lung. 2002;3 1:219-	ICU setting
	Dimick JB, Swoboda SM, Pronovost PJ, et al. Effect of nurse-to-patient ratio in the intensive care unit on pulmonary complications and re-source use after hepatectomy. Am J Crit Care. 2001	ICU setting
	Pronovost PJ, Dang D, Dorman T, et al. Intensive care unit nurse staffing and the risk for complications after abdominal aortic surgery. EffClin Pract. 2001 ;4: 19	ICU setting
	Pronovost PJ, Jenckes MW, Dorman T, et al. Organizational charac-teristics of intensive care units related to outcomes of abdominal aortic surgery. JAMA. 1999;281:1310-1	ICU setting
	Shortell SM, Zimmerman JE, Rousseau DM, et al. The performance of intensive care units: does good management make a difference? Med Care. 1994;32:508-525.	ICU setting
	Cimiotti JP, Haas J, Saiman L, et al. Impact of staffing on bloodstream infections in the neonatal intensive	ICU setting

For peer review only

	care unit. Arch Pediatr Adolesc Med. 2006;160:832-836	
	Marcin JP, Rutan E, Rapetti PM, et al. Nurse staffing and unplanned extubation in the pediatric intensive care unit. Pediatr Crit Care Med. 2005;6:254-257.	ICU setting
	Cimiotti JP. Nurse Staffing and Healthcare-Associated Infections in the Neonatal ICU [dissertation]. Ann Arbor, MI: Columbia University; 2004: AAT 3128935.	ICU setting
	Robert J, Fridkin SK, Blumberg HM, Anderson B, White N, Ray SM, Chan J, Jarvis WR. The influence of the composition of the nursing staff on primary bloodstream infection rates in a surgical intensive care unit. Infection Control & Hospital Epidemiology. 2000 Jan;21(1):12-7.	ICU setting
Labelle 2019		
	Dang D, Johantgen ME, Pronovost PJ, Jenckes MW, Bass EB. Postoperative complications: does intensive care unit staff nursing make a difference?. Heart & Lung. 2002 May 1;31(3):219-28.	ICU setting
	Rao AD, Kumar A, McHugh M. Better nurse autonomy decreases the odds of 30-day mortality and failure to rescue. Journal of Nursing Scholarship. 2017 Jan;49(1):73-9.	wrong intervention (nurse work environment)
	Diya L, Van den Heede K, Sermeus W, Lesaffre E. The relationship between in-hospital mortality, readmission into the intensive care nursing unit and/or operating theatre and nurse staffing levels. Journal of advanced nursing. 2012 May;68(5):1073-81.	ICU setting
	Hickey PA, Gauvreau K, Jenkins K, Fawcett J, Hayman L. Statewide and national impact of California's staffing law on pediatric cardiac surgery outcomes. JONA: The	ICU setting

For peer review only

	Journal of Nursing Administration. 2011 May 1;41(5):218-25.	
	Hickey P, Gauvreau K, Connor J, Sporing E, Jenkins K. The relationship of nurse staffing, skill mix, and Magnet® recognition to institutional volume and mortality for congenital heart surgery. JONA: The Journal of Nursing Administration. 2010 May 1;40(5):226-32.	ICU setting
	Dimick JB, Swoboda SM, Pronovost PJ, Lipsett PA. Effect of nurse-to-patient ratio in the intensive care unit on pulmonary complications and resource use after hepatectomy. American Journal of Critical Care. 2001 Nov 1;10(6):376.	ICU setting
	Kiekkas P, Sakellaropoulos GC, Brokalaki H, Manolis E, Samios A, Skartsani C, Baltopoulos GI. Association between nursing workload and mortality of intensive care unit patients. Journal of nursing scholarship. 2008 Dec;40(4):385-90.	ICU setting
	Olds DM, Aiken LH, Cimiotti JP, Lake ET. Association of nurse work environment and safety climate on patient mortality: A cross-sectional study. International journal of nursing studies. 2017 Sep 1;74:155-61.	wrong intervention (nurse work environment)
	Kendall-Gallagher D, Aiken LH, Sloane DM, Cimiotti JP. Nurse specialty certification, inpatient mortality, and failure to rescue. Journal of Nursing Scholarship. 2011 Jun;43(2):188-94.	wrong intervention (nurse education)
	Lane-Fall MB, Ramaswamy TS, Brown SE, He X, Gutsche JT, Fleisher LA, Neuman MD. Structural, nursing, and physician characteristics and 30-day mortality for patients undergoing cardiac	wrong intervention (nurse education)

For peer review only

	surgery in Pennsylvania. Critical care medicine. 2017 Sep;45(9):1472.	
	Diya L, Lesaffre E, Van den Heede K, Sermeus W, Vleugels A. Establishing the relationship between nurse staffing and hospital mortality using a clustered discrete-time logistic model. Statistics in medicine. 2010 Mar 30;29(7-8):778-85.	ICU setting
	Aiken LH, Clarke SP, Cheung RB, Sloane DM, Silber JH. Educational levels of hospital nurses and surgical patient mortality. Jama. 2003 Sep 24;290(12):1617-23.	wrong intervention (nurse education)
	Aiken LH, Clarke SP, Sloane DM, International Hospital Outcomes Research Consortium. Hospital staffing, organization, and quality of care: cross-national findings. International Journal for quality in Health care. 2002 Feb 1;14(1):5-14.	wrong outcome (nurse outcomes)
	Ozdemir BA, Sinha S, Karthikesalingam A, Poloniecki JD, Pearse RM, Grocott MP, Thompson MM, Holt PJ. Mortality of emergency general surgical patients and associations with hospital structures and processes. BJA: British Journal of Anaesthesia. 2016 Jan 1;116(1):54-62.	Emergency department
	Newhouse RP, Johantgen M, Pronovost PJ, Johnson E. Perioperative nurses and patient outcomes—mortality, complications, and length of stay. AORN journal. 2005 Mar 1;81(3):508-28.	Operating room/peri-operative setting
	Aiken LH, Sloane D, Griffiths P, Rafferty AM, Bruyneel L, McHugh M, Maier CB, Moreno-Casbas T, Ball JE, Ausserhofer D, Sermeus W. Nursing skill mix in European hospitals: cross-sectional study of the association with mortality, patient ratings, and quality of care.	wrong intervention (nursing skill mix)

For peer review only

	BMJ quality & safety. 2017 Jul 1;26(7):559-68.	
Wilson 2011		
	Archibald LK, Manning ML, Bell LM, Banerjee S, Jarvis WR. Patient density, nurse-to-patient ratio and nosocomial infection risk in a pediatric cardiac intensive care unit. The Pediatric infectious disease journal. 1997 Nov 1;16(11):1045-8.	ICU setting
	Cimiotti JP. Nurse staffing and healthcare-associated infections in the neonatal ICU. Columbia University; 2004	ICU setting
	Marcin JP, Rutan E, Rapetti PM, et al. Nurse staffing and unplanned extubation in the pediatric intensive care unit. Pediatr Crit Care Med. 2005;6:254-257.	ICU setting
Engineer 2015		
	Volpp KG, Rosen AK, Rosenbaum PR, Romano PS, Even-Shoshan O, Wang Y, Bellini L, Behringer T, Silber JH. Mortality among hospitalized Medicare beneficiaries in the first 2 years following ACGME resident duty hour reform. Jama. 2007 Sep 5;298(9):975-83.	wrong intervention
	Bradley EH, Herrin J, Curry L, Cherlin EJ, Wang Y, Webster TR, Drye EE, Normand SL, Krumholz HM. Variation in hospital mortality rates for patients with acute myocardial infarction. The American journal of cardiology. 2010 Oct 15;106(8):1108-12.	wrong intervention
	Weiner BJ, Alexander JA, Shortell SM, Baker LC, Becker M, Geppert JJ. Quality improvement implementation and hospital performance on quality indicators. Health services research. 2006 Apr;41(2):307-34.	wrong intervention

For peer review only

	Gaskin DJ, Spencer CS, Richard P, Anderson G, Powe NR, LaVeist TA. Do minority patients use lower quality hospitals?. INQUIRY: The Journal of Health Care Organization, Provision, and Financing. 2011 Aug;48(3):209-20.	wrong intervention
	Carretta HJ, Chukmaitov A, Tang A, Shin J. Examination of hospital characteristics and patient quality outcomes using four inpatient quality indicators and 30-day all-cause mortality. American Journal of Medical Quality. 2013 Jan;28(1):46-55.	wrong intervention
	Chukmaitov AS, Bazzoli GJ, Harless DW, Hurley RE, Devers KJ, Zhao M. Variations in inpatient mortality among hospitals in different system types, 1995 to 2000. Medical care. 2009 Apr 1:466-73.	wrong intervention
	Friedman B, Jiang HJ. Do Medicare Advantage enrollees tend to be admitted to hospitals with better or worse outcomes compared with fee-for-service enrollees?. International journal of health care finance and economics. 2010 Jun;10(2):171-85.	wrong intervention
	Romley JA, Jena AB, Goldman DP. Hospital spending and inpatient mortality: evidence from California: an observational study. Annals of internal medicine. 2011 Feb 1;154(3):160-7.	wrong intervention
	Ross JS, Normand SL, Wang Y, Nallamothu BK, Lichtman JH, Krumholz HM. Hospital remoteness and thirty-day mortality from three serious conditions. Health Affairs. 2008 Nov;27(6):1707-17.	wrong intervention
	Ross JS, Normand SL, Wang Y, Ko DT, Chen J, Drye EE, Keenan PS, Lichtman JH, Bueno H, Schreiner GC, Krumholz HM. Hospital volume and 30-day mortality for three common medical conditions. New England	wrong intervention

For peer review only

	Journal of Medicine. 2010 Mar 25;362(12):1110-8.	
	Sari N. Do competition and managed care improve quality?. Health economics. 2002 Oct;11(7):571-84.	wrong intervention
	Shahian DM, Nordberg P, Meyer GS, Blanchfield BB, Mort EA, Torchiana DF, Normand SL. Contemporary performance of US teaching and nonteaching hospitals. Academic Medicine. 2012 Jun 1;87(6):701-8.	wrong intervention
	Volpp KG, Rosen AK, Rosenbaum PR, Romano PS, Even-Shoshan O, Wang Y, Bellini L, Behringer T, Silber JH. Mortality among hospitalized Medicare beneficiaries in the first 2 years following ACGME resident duty hour reform. Jama. 2007 Sep 5;298(9):975-83.	wrong intervention
	Menachemi N, Chukmaitov A, Saunders C, Brooks RG. Hospital quality of care: does information technology matter? The relationship between information technology adoption and quality of care. Health care management review. 2008 Jan 1;33(1):51-9.	wrong intervention
Staplers et al		
	Kendall-Gallagher D, Blegen MA. Competence and certification of registered nurses and safety of patients in intensive care units. American Journal of Critical Care. 2009 Mar;18(2):106-13	ICU setting
	Hall LM, Doran D, Pink GH. Nurse staffing models, nursing hours, and patient safety outcomes. JONA: The Journal of Nursing Administration. 2004 Jan 1;34(1):41-5.	wrong intervention (skill mix)
	Bae SH, Mark B, Fried B. Impact of nursing unit turnover on patient outcomes in hospitals. Journal of Nursing Scholarship. 2010 Mar;42(1):40-9.	wrong intervention (turn over rates)

For peer review only

	Bae SH, Mark B, Fried B. Use of temporary nurses and nurse and patient safety outcomes in acute care hospital units. Health care management review. 2010 Oct 1;35(4):333-44.	wrong intervention (skill mix)
	Chang YK, Hughes LC, Mark B. Fitting in or standing out: Nursing workgroup diversity and unit-level outcomes. Nursing Research. 2006 Nov 1;55(6):373-80.	wrong intervention
	Krapohl G, Manojlovich M, Redman R, Zhang L. Nursing specialty certification and nursing-sensitive patient outcomes in the intensive care unit. American Journal of Critical Care. 2010 Nov;19(6):490-8.	wrong intervention
	Mallidou AA, Cummings GG, Estabrooks CA, Giovannetti PB. Nurse specialty subcultures and patient outcomes in acute care hospitals: A multiple-group structural equation modeling. International journal of nursing studies. 2011 Jan 1;48(1):81-93.	wrong intervention
	Manojlovich M, Antonakos CL, Ronis DL. Intensive care units, communication between nurses and physicians, and patients' outcomes. American Journal of Critical Care. 2009 Jan;18(1):21-30.	wrong intervention
	Manojlovich M, Sidani S, Covell CL, Antonakos CL. Nurse dose: linking staffing variables to adverse patient outcomes. Nursing research. 2011 Jul 1;60(4):214-20.	wrong intervention
	Purdy N, SPENCE LASCHINGER HK, Finegan J, Kerr M, Olivera F. Effects of work environments on nurse and patient outcomes. Journal of nursing management. 2010 Nov;18(8):901-13.	wrong intervention
	Stone PW, Mooney-Kane C, Larson EL, Horan T, Glance LG, Zwanziger J, Dick AW. Nurse working conditions and patient safety outcomes. Medical care. 2007 Jun 1:571-8.	ICU setting

For peer review only

	Wolf D, Lehman L, Quinlin R, Rosenzweig M, Friede S, Zullo T, Hoffman L. Can nurses impact patient outcomes using a patient-centered care model?. JONA: The Journal of Nursing Administration. 2008 Dec 1;38(12):532-40.	ICU setting
Lankshear et al		
	Aiken LH, Smith HL, Lake ET. Lower Medicare mortality among a set of hospitals known for good nursing care. Medical care. 1994 Aug 1:771-87.	wrong intervention (magnet versus non-magnet)
	Jarman B, Gault S, Alves B, Hider A, Dolan S, Cook A, Hurwitz B, Iezzoni LI. Explaining differences in English hospital death rates using routinely collected data. Bmj. 1999 Jun 5;318(7197):1515-20.	wrong intervention (Used other clinical staffing (doctors))
	Hall LM, Doran D, Pink GH. Nurse staffing models, nursing hours, and patient safety outcomes. JONA: The Journal of Nursing Administration. 2004 Jan 1;34(1):41-5.	skill mix
Mitchell et al		
	Andersen BM, Rasch M, Hochlin K, Tollefsen T, Sandvik L. Hospital-acquired infections before and after healthcare reorganization in a tertiary university hospital in Norway. Journal of public health. 2009 Mar 1;31(1):98-104.	wrong intervention (nursing workload)
	Geubbels EL, Wille JC, Nagelkerke NJ, Vandenbroucke-Grauls CM, Grobbee DE, de Boer AS. Hospital-related determinants for surgical-site infection following hip arthroplasty. Infection Control & Hospital Epidemiology. 2005 May;26(5):435-41.	Wrong intervention
	Roche M, Duffield C, Aisbett C, Diers D, Stasa H. Nursing work directions in Australia: Does evidence drive the policy?. Collegian. 2012 Dec 1;19(4):231-8.	wrong intervention (skill mix)

For peer review only

	Kelly D, Kutney-Lee A, Lake ET, Aiken LH. The critical care work environment and nurse-reported health care–associated infections. American Journal of Critical Care. 2013 Nov;22(6):482-8.	ICU setting
	Dimick JB, Swoboda SM, Pronovost PJ, Lipsett PA. Effect of nurse-to-patient ratio in the intensive care unit on pulmonary complications and resource use after hepatectomy. American Journal of Critical Care. 2001 Nov 1;10(6):376.	ICU setting
	Berney B, Needleman J. Impact of nursing overtime on nurse-sensitive patient outcomes in New York hospitals, 1995-2000. Policy, Politics, & Nursing Practice. 2006 May;7(2):87-100.	wrong intervention (nurses overtime)
	Alonso-Echanove J, Edwards JR, Richards MJ, Brennan P, Venezia RA, Keen J, Ashline V, Kirkland K, Chou E, Hupert M, Veeder AV. Effect of nurse staffing and antimicrobial-impregnated central venous catheters on the risk for bloodstream infections in intensive care units. Infection Control & Hospital Epidemiology. 2003 Dec;24(12):916-25.	ICU setting
	Cimiotti JP, Haas J, Saiman L, Larson EL. Impact of staffing on bloodstream infections in the neonatal intensive care unit. Archives of pediatrics & adolescent medicine. 2006 Aug 1;160(8):832-6.	ICU setting
	Pronovost PJ, Dang D, Dorman T, Lipsett PA, Garrett E, Jenckes M, Bass EB. Intensive care unit nurse staffing and the risk for complications after abdominal aortic surgery. Effective clinical practice: ECP. 2001 Sep 1;4(5):199-206.	ICU setting
	Robert J, Fridkin SK, Blumberg HM, Anderson B, White N, Ray SM, Chan J, Jarvis WR. The influence of the	ICU setting

For peer review only

	composition of the nursing staff on primary bloodstream infection rates in a surgical intensive care unit. Infection Control & Hospital Epidemiology. 2000 Jan;21(1):12-7.	
	UK Neonatal Staffing Study Group. Patient volume, staffing, and workload in relation to risk-adjusted outcomes in a random stratified sample of UK neonatal intensive care units: a prospective evaluation. The Lancet. 2002 Jan 12;359(9301):99-107.	ICU setting
	Hugonnet S, Uçkay I, Pittet D. Staffing level: a determinant of late-onset ventilator-associated pneumonia. Critical Care. 2007 Aug;11(4):1-7.	ICU setting
	Bae SH, Brewer CS, Kelly M, Spencer A. Use of temporary nursing staff and nosocomial infections in intensive care units. Journal of clinical nursing. 2015 Apr;24(7-8):980-90.	ICU setting
	Kendall-Gallagher D, Blegen MA. Competence and certification of registered nurses and safety of patients in intensive care units. American Journal of Critical Care. 2009 Mar;18(2):106-13.	ICU setting
	Stone PW, Mooney-Kane C, Larson EL, Horan T, Glance LG, Zwanziger J, Dick AW. Nurse working conditions and patient safety outcomes. Medical care. 2007 Jun 1:571-8.	ICU setting
	Schwab F, Meyer E, Geffers C, Gastmeier P. Understaffing, overcrowding, inappropriate nurse: ventilated patient ratio and nosocomial infections: which parameter is the best reflection of deficits?. Journal of Hospital Infection. 2012 Feb 1;80(2):133-9.	ICU setting
	Amaravadi RK, Dimick JB, Pronovost PJ, Lipsett PA. ICU nurse-to-patient ratio is associated with complications and resource use	ICU setting

For peer review only

	after esophagectomy. Intensive care medicine. 2000 Dec;26(12):1857-62.	
	Dancer SJ, Coyne M, Speekenbrink A, Samavedam S, Kennedy J, Wallace PG. MRSA acquisition in an intensive care unit. American journal of infection control. 2006 Feb 1;34(1):10-7.	ICU setting
	Grundmann H, Hori S, Winter B, Tami A, Austin DJ. Risk factors for the transmission of methicillin-resistant Staphylococcus aureus in an adult intensive care unit: fitting a model to the data. The Journal of infectious diseases. 2002 Feb 15;185(4):481-8.	ICU setting
	Kong F, Cook D, Paterson DL, Whitby M, Clements AC. Do staffing and workload levels influence the risk of new acquisitions of meticillin-resistant Staphylococcus aureus in a well-resourced intensive care unit?. Journal of Hospital Infection. 2012 Apr 1;80(4):331-9.	ICU setting
	Hugonnet S, Villaveces A, Pittet D. Nurse staffing level and nosocomial infections: empirical evaluation of the case-crossover and case-time-control designs. American journal of epidemiology. 2007 Jun 1;165(11):1321-7.	ICU setting
	Rogowski JA, Staiger D, Patrick T, Horbar J, Kenny M, Lake ET. Nurse staffing and NICU infection rates. JAMA pediatrics. 2013 May 1;167(5):444-50.	ICU setting
	Parikh A, Huang SA, Murthy P, Dombrovskiy V, Nolledo M, Lefton R, Scardella AT. Quality improvement and cost savings after implementation of the Leapfrog intensive care unit physician staffing standard at a community teaching hospital. Critical care medicine. 2012 Oct 1;40(10):2754-9.	ICU setting

For peer review only

	Hugonnet S, Chevrolet JC, Pittet D. The effect of workload on infection risk in critically ill patients. Critical care medicine. 2007 Jan 1;35(1):76-81.	ICU setting
	Maillet JM, Guérot E, Novara A, Le Guen J, Lahjibi-Paulet H, Kac G, Diehl JL, Fagon JY. Comparison of intensive-care-unit-acquired infections and their outcomes among patients over and under 80 years of age. Journal of Hospital Infection. 2014 Jul 1;87(3):152-8.	ICU setting
	Daud-Gallotti RM, Costa SF, Guimarães T, Padilha KG, Inoue EN, Vasconcelos TN, da Silva Cunha Rodrigues F, Barbosa EV, Figueiredo WB, Levin AS. Nursing workload as a risk factor for healthcare associated infections in ICU: a prospective study. PloS one. 2012 Dec 27;7(12):e52342.	ICU setting
	The U. Relationship between probable nosocomial bacteraemia and organisational and structural factors in UK neonatal intensive care units. Quality & safety in health care. 2005 Aug;14(4):264.	ICU setting
	Halwani M, Solaymani-Dodaran M, Grundmann H, Coupland C, Slack RJ. Cross-transmission of nosocomial pathogens in an adult intensive care unit: incidence and risk factors. Journal of Hospital Infection. 2006 May 1;63(1):39-46.	ICU setting
	Dorsey G, Borneo HT, Sun SJ, Wells J, Steele L, Howland K, Perdreau-Remington F, Bangsberg DR. A heterogeneous outbreak of Enterobacter cloacae and Serratia marcescens infections in a surgical intensive care unit. Infection Control & Hospital Epidemiology. 2000 Jul;21(7):465-9.	ICU setting
	Fraher MH, Collins CJ, Bourke J, Phelan D, Lynch M. Cost-effectiveness of employing a total	wrong intervention (Addition of a specialist nurse)

For peer review only

	parenteral nutrition surveillance nurse for the prevention of catheter-related bloodstream infections. Journal of Hospital Infection. 2009 Oct 1;73(2):129-34.	
	Barkell NP, Killinger KA, Schultz SD. The relationship between nurse staffing models and patient outcomes: a descriptive study. Outcomes Management. 2002 Jan 1;6(1):27-33.	wrong intervention (skill mix)
	Hall LM, Doran D, Pink GH. Nurse staffing models, nursing hours, and patient safety outcomes. JONA: The Journal of Nursing Administration. 2004 Jan 1;34(1):41-5.	wrong intervention (skill mix)
	Yang PH, Hung CH, Chen YM, Hu CY, Shieh SL. The impact of different nursing skill mix models on patient outcomes in a respiratory care center. Worldviews on Evidence-Based Nursing. 2012 Nov;9(4):227-33.	wrong intervention (skill mix)
	Virtanen M, Kurvinen T, Terho K, Oksanen T, Peltonen R, Vahtera J, Routamaa M, Elovainio M, Kivimäki M. Work hours, work stress, and collaboration among ward staff in relation to risk of hospital-associated infection among patients. Medical care. 2009 Mar 1:310-8.	wrong intervention (overtime)
	Dimick JB, Pronovost PJ, Heitmiller RF, Lipsett PA. Intensive care unit physician staffing is associated with decreased length of stay, hospital cost, and complications after esophageal resection. Critical care medicine. 2001 Apr 1;29(4):753-8.	wrong intervention (Physician staffing)

For peer review only

For peer review only

Online supplemental file 4: Overlap of primary papers across reviews

Study name	Thungjaroenkul P et al (2007)	Twigg et al (2020)	Assaye et al (2020)	Bourgon et al (2019)	Shin et al (2019)	Wilson et al (2011)	Kane et al (2007)	Griffiths et al (2019)	Lankshear et al (2005)	Engineer et al (2015)	Staplers et al (2015)	Mitchell et al (2018)	Lang et al (2004)	Hill (2017)	Frequency
Aiken LH, Cimiotti JP, Sloane DM, Smith HL, Flynn L, Neff DF. Effects of nurse staffing and nurse education on patient deaths in hospitals with different nurse work environments. The Journal of nursing administration. 2012	No	No	No	Yes	No	No	No	No	No	No	No	No	No	No	1

For peer review only

Oct;42(10 Suppl):S10.															
Aiken LH, Clarke SP, Cheung RB, Sloane DM, Silber JH. Educational levels of hospital nurses and surgical patient mortality. Jama. 2003 Sep 24;290(12):1617-23.	No	No	No	No	No	No	Yes	No	Yes	No	No	No	Yes	No	3
Aiken LH, Clarke SP, Sloane DM, Lake ET, Cheney T. Effects of hospital care environment on patient mortality and nurse outcomes. The Journal	No	No	No	Yes	No	No	No	No	No	No	No	No	No	No	1

For peer review only

of nursing administration. 2008 May;38(5):223.															
Aiken LH, Clarke SP, Sloane DM, Sochalski J, Silber JH. Hospital nurse staffing and patient mortality, nurse burnout, and job dissatisfaction. Jama. 2002 Oct 23;288(16):1987-93.	No	No	No	Yes	No	No	Yes	No	Yes	No	No	No	Yes	No	4
Aiken LH, Clarke SP, Sloane DM. Hospital restructuring: does it adversely affect care and outcomes?. JONA: The	No	No	No	No	No	No	No	No	No	No	No	No	Yes	No	1

For peer review only

Journal of Nursing Administration. 2000 Oct 1;30(10):457-65.														
Aiken LH, Sloane DM, Bruyneel L, Van den Heede K, Griffiths P, Busse R, Diomidous M, Kinnunen J, Kózka M, Lesaffre E, McHugh MD. Nurse staffing and education and hospital mortality in nine European countries: a retrospective observational study. The lancet. 2014 May 24;383(9931):1824-30.	No	No	No	Yes	No	No	No	No	No	No	No	No	No	No	1

For peer review only

Aiken LH, Sloane DM, Lake ET, Sochalski J, Weber AL. Organization and outcomes of inpatient AIDS care. Medical care. 1999 Aug 1:760-72.	No	No	No	No	No	No	Yes	No	No	No	No	No	No	No	1
Al-Kandari F, Thomas D. Factors contributing to nursing task incompletion as perceived by nurses working in Kuwait general hospitals. Journal of clinical nursing. 2009 Dec;18(24):3430-40.	No	No	No	No	No	No	No	Yes	No	No	No	No	No	No	1

For peer review only

American Nurses Association. Implementing nursing's report card: A study of RN staffing, length of stay, and patient outcomes. Amer Nurses Assn; 1997.	No	No	No	No	No	No	Yes	No	No	No	No	No	No	No	1
American Nurses Association. Nurse staffing and patient outcomes in the inpatient hospital setting. Amer Nurses Assn; 2000.	No	No	No	No	No	No	No	No	No	No	No	No	Yes	No	1
Arbesman MC, Wright C. Mechanical restraints,	No	No	No	No	No	No	No	No	No	No	No	No	Yes	No	1

For peer review only

rehabilitation therapies, and staffing adequacy as risk factors for falls in an elderly hospitalized population. Rehabilitation Nursing. 1999 May 6;24(3):122-8.															
Ausserhofer, D., Zander, B., Busse, R., Schubert, M., De Geest, S., & Rafferty, A. M., ... Consortium, R. C. (2014). Prevalence, patterns and predictors of nursing care left undone in European hospitals: Results from the multicountr	No	No	No	No	No	No	No	Yes	No	No	No	No	No	No	1

For peer review only

y cross-sectional RN4CAST study. BMJ Quality & Safety, 23(2), 126–135. https://doi.org/10.1136/bmjqs2013-002318														
Ball JE, Bruyneel L, Aiken LH, Sermeus W, Sloane DM, Rafferty AM, Lindqvist R, Tishelman C, Griffiths P, RN4Cast Consortium. Post-operative mortality, missed care and nurse staffing in nine countries: a cross-sectional study.	No	No	No	Yes	No	No	No	No	No	No	No	No	No	No	1

For peer review only

International journal of nursing studies. 2018 Feb 1;78:10-5.															
Ball, J. E., Griffiths, P., Rafferty, A. M., Lindqvist, R., Murrells, T., & Tishelman, C. (2016). A cross-sectional study of 'care left undone'on nursing shifts in hospitals. Journal of Advanced Nursing, 72, 2086–2097. https://doi.org/10.1111/jan.12976	No	No	No	No	No	No	No	Yes	No	No	No	No	No	No	1
Ball, J. E., Murrells, T., Rafferty, A. M., Morrow,	No	No	No	No	No	No	No	Yes	No	No	No	No	No	No	1

For peer review only

E., & Griffiths, P. (2014). 'Care left undone' during nursing shifts: Associations with workload and perceived quality of care. BMJ Quality & Safety, 23(2), 116–125. https://doi.org/10.1136/bmjqs-2012-001767														
Behner KG, Fogg LF, Fournier LC, Frankenbach JT, Robertson SB. Nursing resource management: analyzing the relationship	Yes	No	No	No	No	No	No	No	No	No	No	No	No	No	1

For peer review only

between costs and quality in staffing decisions. Health Care Management Review. 1990 Jan 1;15(4):63-71.															
Berney B, Needleman J. Impact of nursing overtime on nurse-sensitive patient outcomes in New York hospitals, 1995-2000. Policy, Politics, & Nursing Practice. 2006 May;7(2):87-100.	No	No	No	Yes	No	No	No	No	No	No	No	No	No	No	1
Blegen MA, Goode CJ, Reed L. Nurse	No	No	No	No	No	No	No	No	No	No	No	No	Yes	No	1

For peer review only

staffing and patient outcomes. Nursing research. 1998 Jan 1;47(1):43-50.															
Blegen MA, Goode CJ, Spetz J, Vaughn T, Park SH. Nurse staffing effects on patient outcomes: safety-net and non-safety-net hospitals. Medical care. 2011 Apr 1:406-14.	No	No	No	Yes	No	No	No	No	No	No	No	No	No	No	1
Blegen MA, Vaughn T. A multisite study of nurse staffing and patient occurrences.	No	No	No	No	No	No	No	No	Yes	No	No	No	Yes	No	2

For peer review only

Nursing Economics. 1998 Jul 1;16(4):196.															
Bond CA, Raehl CL, Franke T. Medication errors in United States hospitals. Pharmacotherapy: The Journal of Human Pharmacology and Drug Therapy. 2001 Sep;21(9):1023-36.	No	No	No	No	No	No	No	No	Yes	No	No	No	No	No	1
Bond CA, Raehl CL, Pitterle ME, Franke T. Health care professional staffing, hospital characteristics, and hospital mortality	No	No	No	No	No	No	No	No	Yes	No	No	No	Yes	No	2

For peer review only

rates. Pharmacotherapy: The Journal of Human Pharmacology and Drug Therapy. 1999 Feb;19(2):130-8.															
Breckenridge-Sproat S, Johantgen M, Patrician P. Influence of unit-level staffing on medication errors and falls in military hospitals. Western journal of nursing research. 2012 Jun;34(4):455-74.	No	No	No	No	No	No	No	No	No	No	Yes	No	No	No	1
Brooks Carthon JM, Kutney-Lee A, Jarrín O,	No	No	No	Yes	No	No	No	No	No	No	No	No	No	No	1

For peer review only

Sloane D, Aiken LH. Nurse staffing and postsurgical outcomes in black adults. Journal of the American Geriatrics Society. 2012 Jun;60(6):1078-84.														
Bruyneel, L., Li, B., Ausserhofer, D., Lesaffre, E., Dumitrescu, I., Smith, H. L., & Sermeus, W. (2015). Organization of hospital nursing, provision of nursing care and patient experiences with care in Europe.	No	No	No	No	No	No	No	Yes	No	No	No	No	No	No	1

Medical Care Research and Review, 72(6), 643–664. https://doi.org/10.1177/1077558715589188															
Burnes Bolton L, Aydin CE, Donaldson N, Storer Brown D, Sandhu M, Fridman M, Udin Aronow H. Mandated nurse staffing ratios in California: a comparison of staffing and nursing-sensitive outcomes pre-and postregulation. Policy, Politics, &	No	Yes	No	No	No	No	No	No	No	No	Yes	No	No	No	2

For peer review only

Nursing Practice. 2007 Nov;8(4):238-50.															
Carter JH, Mills AC, Homan SM, Blaesing SL, Heater BS, Stoll LD, Mornin C, Corrigan MK. Correlating the quality of care with nursing resources and patient parameters: a longitudinal study. NLN publications. 1987 Dec(20-2191):331-45.													Yes	No	1
Cheung RB. The relationship between nurse	No	No	No	No	No	No	Yes	No	No	No	No	No	No	No	1

For peer review only

staffing, nursing time, and adverse events in an acute care hospital. University of South Florida; 2002.															
Cho E, Lee NJ, Kim EY, Kim S, Lee K, Park KO, Sung YH. Nurse staffing level and overtime associated with patient safety, quality of care, and care left undone in hospitals: a cross-sectional study. International journal of nursing	No	No	No	No	No	N o	No	Yes	No	No	No	No	No	No	1

For peer review only

studies. 2016 Aug 1;60:263-71.															
Cho E, Sloane DM, Kim EY, Kim S, Choi M, Yoo IY, Lee HS, Aiken LH. Effects of nurse staffing, work environments, and education on patient mortality: an observational study. International journal of nursing studies. 2015 Feb 1;52(2):535-42.	No	No	No	Yes	No	No	No	No	No	No	No	No	No	No	1

For peer review only

Cho SH, Ketefian S, Barkauskas VH, Smith DG. The effects of nurse staffing on adverse events, morbidity, mortality, and medical costs. Nursing research. 2003 Mar 1;52(2):71-9.	Yes	No	No	No	No	No	Yes	No	Yes	No	No	No	Yes	No	4
Cho SH, Kim YS, Yeon KN, You SJ, Lee ID. Effects of increasing nurse staffing on missed nursing care. International nursing review. 2015	No	No	No	No	No	No	No	Yes	No	No	No	No	No	No	1

For peer review only

Jun;62(2):267-74.														
Christine K, Cheryl J, Chunliu Z, Peter JG, Jayasree B. Nurse staffing and postsurgical adverse events: an analysis of administrative data from a sample of US hospitals, 1990–1996. Health services research. 2002 Jun;37(3):611-29.	No	No	No	No	No	No	No	No	Yes	No	No	Yes	Yes	No	3
Cimiotti JP, Aiken LH, Sloane DM, Wu ES. Nurse staffing, burnout, and health	No	No	No	No	No	No	No	No	No	No	No	Yes	No	No	1

For peer review only

care–associated infection. American journal of infection control. 2012 Aug 1;40(6):486-90.															
Cook A, Gaynor M, Stephens Jr M, Taylor L. The effect of a hospital nurse staffing mandate on patient health outcomes: Evidence from California's minimum staffing regulation. Journal of Health Economics. 2012 Mar 1;31(2):340-8.	No	Yes	No	No	No	No	No	No	No	No	No	No	No	No	1

For peer review only

Dabney BW, Kalisch BJ. Nurse staffing levels and patient-reported missed nursing care. Journal of nursing care quality. 2015 Oct 1;30(4):306-12.	No	No	No	No	No	No	No	Yes	No	No	No	No	No	No	1
Dobal MT. The relationships among the context, structure, and performance of nursing units in hospitals (Doctoral dissertation, The University of Texas at Austin).	No	No	No	No	No	No	No	No	No	No	No	No	Yes	No	1

For peer review only

Donaldson N, Bolton LB, Aydin C, Brown D, Elashoff JD, Sandhu M. Impact of California's licensed nurse-patient ratios on unit-level nurse staffing and patient outcomes. Policy, Politics, & Nursing Practice. 2005 Aug;6(3):198-210.	No	Yes	No	No	No	No	No	No	No	No	Yes	No	No	No	2
Dunton N, Gajewski B, Taunton RL, Moore J. Nurse staffing and patient falls on acute care hospital units.	No	No	No	No	Yes	No	No	No	No	No	No	No	No	No	1

For peer review only

Nursing outlook. 2004 Feb 1;52(1):53-9.															
Elkassabany NM, Passarella M, Mehta S, Liu J, Neuman MD. Hospital characteristics, inpatient processes of care, and readmissions of older adults with hip fractures. Journal of the American Geriatrics Society. 2016 Aug;64(8):1656-61.	No	No	No	Yes	No	No	No	No	No	No	No	No	No	No	1
Elting LS, Pettaway C, Bekele BN, Grossman	No	No	No	No	No	No	Yes	No	No	No	No	No	No	No	1

For peer review only

HB, Cooksley C, Avritscher EB, Saldin K, Dinney CP. Correlation between annual volume of cystectomy, professional staffing, and outcomes: a statewide, population-based study. Cancer: Interdisciplinary International Journal of the American Cancer Society. 2005 Sep 1;104(5):975-84.															
Everhart, D. M., Schumacher, J. R., Duncan, R. P., Hall, A.	No	No	No	No	Yes	No	No	No	No	No	No	No	No	No	1

G., Neff, D. F., & Shorr, R. I. (2014). Determinants of hospital fall rate trajectory groups: A longitudinal assessment of nurse staffing and organiza-tional characteristics. Health Care Management Review, 39(4), 352–360														
Feleke SA, Mulatu MA, Yesmaw YS. Medication administration error: magnitude and associated factors among nurses in Ethiopia.	No	No	Yes	No	No	No	No	No	No	No	No	No	No	No	1

For peer review only

BMC nursing. 2015 Dec;14(1):1-8.														
Flood SD, Diers D. Nurse staffing, patient outcome and cost. Nursing Management. 1988 May 1;19(5):34-45.	No	No	No	No	No	No	No	No	No	No	No	No	Yes	No	1
Friese CR, Kalisch BJ, Lee KH. Patterns and correlates of missed nursing care in inpatient oncology units. Cancer nursing. 2013 Nov;36(6):E51.	No	No	No	No	No	No	No	Yes	No	No	No	No	No	No	1

For peer review only

Friese CR, Lake ET, Aiken LH, Silber JH, Sochalski J. Hospital nurse practice environments and outcomes for surgical oncology patients. Health services research. 2008 Aug;43(4):1145-63.	No	No	No	Yes	No	No	No	No	No	No	No	No	No	No	1
Frith KH, Anderson EF, Caspers B, Tseng F, Sanford K, Hoyt NG, Moore K. Effects of nurse staffing on hospital-acquired conditions and length	No	No	No	No	Yes	No	No	No	No	No	Yes	No	No	No	2

For peer review only

of stay in community hospitals. Quality Management in Healthcare. 2010 Apr 1;19(2):147-55.															
Ghaferi AA, Osborne NH, Birkmeyer JD, Dimick JB. Hospital characteristics associated with failure to rescue from complications after pancreatectomy. Journal of the American College of Surgeons. 2010 Sep 1;211(3):325-30.	No	No	No	Yes	No	No	No	No	No	No	No	No	No	No	1

For peer review only

Glance LG, Dick AW, Osler TM, Mukamel DB, Li Y, Stone PW. The association between nurse staffing and hospital outcomes in injured patients. BMC health services research. 2012 Dec;12(1):1-8	No	No	No	No	No	N o	No	No	No	No	No	Yes	No	No	1
Goode CJ, Blegen MA, Park SH, Vaughn T, Spetz J. Comparison of patient outcomes in Magnet® and non-Magnet hospitals. JONA: The	No	No	No	No	No	N o	No	No	No	No	Yes	No	No	No	1

For peer review only

Journal of Nursing Administration. 2011 Dec 1;41(12):517-23.															
Griffiths P, Dall'Ora C, Simon M, Ball J, Lindqvist R, Rafferty AM, Schoonhoven L, Tishelman C, Aiken LH. Nurses' shift length and overtime working in 12 European countries: the association with perceived quality of care and patient safety. Medical care. 2014	No	No	No	No	No	No	No	Yes	No	No	No	No	No	No	1

Nov;52(11): 975.														
Griffiths P, Jones S, Bottle A. Is "failure to rescue" derived from administrative data in England a nurse sensitive patient safety indicator for surgical care? Observational study. International Journal of Nursing Studies. 2013 Feb 1;50(2):292-300.	No	No	No	Yes	No	No	No	No	No	No	No	No	No	No	1

For peer review only

Gunningberg L, Donaldson N, Aydin C, Idvall E. Exploring variation in pressure ulcer prevalence in Sweden and the USA: benchmarking in action. Journal of evaluation in clinical practice. 2012 Aug;18(4):904-10.	No	No	No	No	No	No	No	No	No	No	Yes	No	No	No	1
Haley RW, Bregman DA. The role of understaffing and overcrowding in recurrent outbreaks of staphylococcal infection	No	No	No	No	No	No	No	No	No	No	No	No	Yes	No	1

For peer review only

in a neonatal special-care unit. Journal of Infectious Diseases. 1982 Jun 1;145(6):875-85.															
Halm M, Peterson M, Kandels M, Sabo J, Blalock M, Braden R, Gryczman A, Krisko-Hagel K, Larson D, Lemay D, Sisler B. Hospital nurse staffing and patient mortality, emotional exhaustion, and job dissatisfaction. Clinical Nurse Specialist. 2005 Sep	No	No	No	Yes	No	No	Yes	No	No	No	No	No	No	No	2

For peer review only

1;19(5):241-51.														
Halm M, Peterson M, Kandels M, Sabo J, Blalock M, Braden R, Gryczman A, Krisko-Hagel K, Larson D, Lemay D, Sisler B. Hospital nurse staffing and patient mortality, emotional exhaustion, and job dissatisfaction. Clinical Nurse Specialist. 2005 Sep 1;19(5):241-51.	No	No	No	No	No	No	No	No	No	No	No	No	No	Yes	1
Harless DW, Mark BA. Nurse staffing and	No	No	No	Yes	No	No	No	No	No	No	No	No	No	No	1

For peer review only

quality of care with direct measurement of inpatient staffing. Medical care. 2010 Jul 1:659-63.															
Hartz AJ, Krakauer H, Kuhn EM, Young M, Jacobsen SJ, Gay G, Muenz L, Katzoff M, Bailey RC, Rimm AA. Hospital characteristics and mortality rates. New England journal of medicine. 1989 Dec 21;321(25):1720-5.	No	No	No	No	No	No	No	No	No	No	No	No	Yes	No	1

For peer review only

Hickey, P. A., Gauvreau, K., Jenkins, K., Fawcett, J., & Hayman, L. (2011). Statewide and national impact of California's staffing law on pediatric cardiac surgery outcomes. JONA: the Journal of Nursing Administration, 41(5), 218–225.	No	Yes	No	No	No	No	No	No	No	No	No	No	No	No	1
Hinno S, Partanen P, Vehviläinen-Julkunen K. Nursing activities, nurse staffing and adverse patient outcomes as	No	No	No	No	Yes	No	No	No	No	No	No	No	No	No	1

For peer review only

perceived by hospital nurses. Journal of clinical nursing. 2012 Jun;21(11-12):1584-93.															
Hope J. Nosocomial Infections and Their Relationship to Nursing Workload in an Acute Care Hospital dissertation. Ottawa, Ontario, Canada: Queen's University. 2003.	No	No	No	No	No	No	Yes	No	No	No	No	No	No	No	1
Houser EP. Nurse staffing levels and patient outcomes. The Johns Hopkins	No	No	No	No	No	No	Yes	No	No	No	No	No	No	No	1

For peer review only

University; 2005.														
Jiang HJ, Stocks C, Wong CJ. Disparities between two common data sources on hospital nurse staffing. Journal of Nursing Scholarship. 2006 Jun;38(2):187-93.	No	No	No	No	No	No	No	No	No	No	Yes	No	No	No	1
Kalisch BJ, Doumit M, Lee KH, El Zein J. Missed nursing care, level of staffing, and job satisfaction: Lebanon versus the United	No	No	No	No	No	No	No	Yes	No	No	No	No	No	No	1

For peer review only

States. JONA: The Journal of Nursing Administration. 2013 May 1;43(5):274-9.														
Kalisch BJ, Tschannen D, Lee H, Friese CR. Hospital variation in missed nursing care. American Journal of Medical Quality. 2011 Jul;26(4):291-9.	No	No	No	No	No	No	No	Yes	No	No	No	No	No	No	1
Kalisch BJ, Tschannen D, Lee KH. Do staffing levels predict missed nursing care?.	No	No	No	No	No	No	No	Yes	No	No	No	No	No	No	1

For peer review only

International Journal for Quality in Health Care. 2011 Jun 1;23(3):302-8.															
Kim CG, Bae KS. Relationship between nurse staffing level and adult nursing-sensitive outcomes in tertiary hospitals of Korea: Retrospective observational study. International Journal of Nursing Studies. 2018 Apr 1;80:155-64.	No	No	No	No	Yes	No	No	No	No	No	No	No	No	No	1

For peer review only

Kim Y, Han K. Longitudinal associations of nursing staff turnover with patient outcomes in long-term care hospitals in Korea. Journal of Nursing Management. 2018 Jul;26(5):518-24.	No	No	No	No	Yes	No	No	No	No	No	No	No	No	No	1
Kouatly IA, Nassar N, Nizam M, Badr LK. Evidence on nurse staffing ratios and patient outcomes in a low-income country: Implications for future	No	No	Yes	No	Yes	No	No	No	No	No	No	No	No	No	2

For peer review only

research and practice. Worldviews on Evidence-Based Nursing. 2018 Oct;15(5):353-60.															
Kovner C, Gergen PJ. Nurse staffing levels and adverse events following surgery in US hospitals. Image: The Journal of Nursing Scholarship. 1998 Dec;30(4):315-21.	No	No	No	No	No	No	No	No	Yes	No	No	No	Yes	No	2
Kutney-Lee A, Aiken LH. Effect of nurse staffing and education	No	No	No	Yes	No	No	No	No	No	No	No	No	No	No	1

For peer review only

on the outcomes of surgical patients with comorbid serious mental illness. Psychiatric Services. 2008 Dec;59(12):1466-9.															
Li X, Bowman SM, Smith TC. Effects of registered nurse staffing level on hospital-acquired conditions in cardiac surgery patients: a propensity score matching analysis. Nursing outlook. 2016 Nov	No	No	No	Yes	No	No	No	No	No	No	No	No	No	No	1

For peer review only

1;64(6):533-41.															
Lichtig LK, Knauf RA, Milholland DK. Some impacts of nursing on acute care hospital outcomes. JONA: The Journal of Nursing Administration. 1999 Feb 1;29(2):25-33.	Yes	No	No	No	No	No	Yes	No	Yes	No	No	No	No	No	3
Magalhães AM, Dall'Agnol CM, Marck PB. Nursing workload and patient safety-a mixed method study with an	No	No	Yes	No	No	No	No	No	No	No	No	No	No	No	1

For peer review only

ecological restorative approach. Revista latino-americana de enfermagem. 2013;21:146-54.															
Manheim LM, Feinglass J, Shortell SM, Hughes EF. Regional variation in Medicare hospital mortality. Inquiry. 1992 Apr 1:55-66.	No	No	No	No	No	No	No	No	Yes	No	No	No	Yes	No	2
Manojlovich M, Sidani S, Covell CL, Antonakos CL. Nurse dose: linking staffing variables to adverse patient	No	No	No	No	No	No	No	No	No	No	No	Yes	No	No	1

For peer review only

outcomes. Nursing research. 2011 Jul 1;60(4):214-20.															
Mark BA, Harless DW, Berman WF. Nurse staffing and adverse events in hospitalized children. Policy, Politics, & Nursing Practice. 2007 May;8(2):83-92.	No	No	No	Yes	No	Yes	No	No	No	No	No	Yes	No	No	3
Mark BA, Harless DW, McCue M, Xu Y. A longitudinal examination of hospital registered nurse staffing and quality of care. Health	No	No	No	No	Yes	No	Yes	No	Yes	No	Yes	Yes	No	No	5

For peer review only

services research. 2004 Apr;39(2):279-300.															
Mark BA, Harless DW, McCue M. The impact of HMO penetration on the relationship between nurse staffing and quality. Health economics. 2005 Jul;14(7):737-53.	No	No	No	No	No	Yes	No	No	No	No	No	No	No	No	1
Mark BA, Harless DW, Spetz J, Reiter KL, Pink GH. California's minimum nurse staffing legislation: results from a natural	No	Yes	No	No	No	No	No	No	No	No	No	No	No	No	1

For peer review only

experiment. Health services research. 2013 Apr;48(2pt1):435-54.															
Mark BA, Harless DW. Nurse staffing and post-surgical complications using the present on admission indicator. Research in nursing & health. 2010 Feb;33(1):35-47.	No	No	No	No	Yes	N o	No	No	No	No	No	Yes	No	No	2
McCloskey BA, Diers DK. Effects of New Zealand's health reengineering on nursing and patient outcomes. Medical care. 2005	No	No	No	Yes	No	N o	No	No	No	No	Yes	No	No	No	2

For peer review only

Nov 1:1140-6.														
McHugh MD, Rochman MF, Sloane DM, Berg RA, Mancini ME, Nadkarni VM, Merchant RM, Aiken LH, American Heart Association's Get With The Guidelines-Resuscitation Investigators. Better nurse staffing and nurse work environments associated with increased	No	No	No	No	No	No	No	No	No	No	No	No	No	Yes	1

For peer review only

survival of in-hospital cardiac arrest patients. Medical care. 2016 Jan;54(1):74 .															
Morita K, Matsui H, Fushimi K, Yasunaga H. Association between nurse staffing and in-hospital bone fractures: a retrospective cohort study. Health services research. 2017 Jun;52(3):1005-23.	No	No	No	No	Yes	No	No	No	No	No	No	No	No	No	1
Needleman J, Buerhaus P, Mattke S, Stewart M, Zelevinsky K.	Yes	No	No	Yes	No	No	Yes	No	Yes	No	No	Yes	No	Yes	6

For peer review only

Nurse-staffing levels and the quality of care in hospitals. New England Journal of Medicine. 2002 May 30;346(22):1715-22.															
Needleman J, Buerhaus PI, Mattke S, Stewart M, Zelevinsky K. Measuring Hospital Quality: Can Medicare Data Substitute for All-Payer Data?. Health services research. 2003 Dec;38(6p1):1487-508.	No	No	No	No	No	No	No	No	No	No	No	Yes	No	No	1

For peer review only

For peer review only - http://bmjopen.bmj.com/site/about/guidelines.xhtml

Needleman J, Buerhaus PI, Mattke S, Stewart M, Zelevinsky K. Nurse staffing and patient outcomes in hospitals. Final report. Rockville, MD: Health Resources and Services Administration. 2001 Feb 28.	No	No	No	No	No	No	No	No	No	No	No	Yes	No	No	1
Neff DF, Cimiotti J, Sloane DM, Aiken LH. Utilization of non-US educated nurses in US hospitals: implications for hospital mortality. International Journal for Quality in Health Care.	No	No	No	Yes	No	No	No	No	No	No	No	No	No	No	1

For peer review only

2013 Sep 1;25(4):366-72.															
Orique SB, Patty CM, Woods E. Missed nursing care and unit-level nurse workload in the acute and post-acute settings. Journal of nursing care quality. 2016 Jan 1;31(1):84-9.	No	No	No	No	No	No	No	Yes	No	No	No	No	No	No	1
Palese A, Ambrosi E, Prosperi L, Guarnier A, Barelli P, Zambiasi P, Allegrini E, Bazoli L, Casson P, Marin M, Padovan M. Missed	No	No	No	No	No	No	No	Yes	No	No	No	No	No	No	1

For peer review only

nursing care and predicting factors in the Italian medical care setting. Internal and emergency medicine. 2015 Sep;10(6):693-702.															
Pappas S, Davidson N, Woodard J, Davis J, Welton JM. Risk-adjusted staffing to improve patient value. Nursing Economics. 2015 Mar 1;33(2):73.	No	No	No	No	No	No	No	No	No	No	No	Yes	No	No	1
Patrician PA, Loan L, McCarthy M, Fridman M, Donaldson	No	No	No	No	No	No	No	No	No	No	Yes	No	No	No	1

For peer review only

N, Bingham M, Brosch LR. The association of shift-level nurse staffing with adverse patient events. JONA: the Journal of Nursing Administration. 2011 Feb 1;41(2):64-70.															
Patrician PA, McCarthy MS, Swiger P, Raju D, Breckenridge-Sproat S, Su X, Randall KH, Loan LA. Association of temporal variations in staffing with hospital-acquired pressure injury in	No	No	No	No	Yes	N o	No	No	No	No	No	No	No	No	1

For peer review only

military hospitals. Research in nursing & health. 2017 Apr;40(2):111-9.															
Person SD, Allison JJ, Kiefe CI, Weaver MT, Williams OD, Centor RM, Weissman NW. Nurse staffing and mortality for Medicare patients with acute myocardial infarction. Medical care. 2004 Jan 1:4-12.	No	No	No	No	No	No	Yes	No	Yes	No	No	No	No	No	2
Prot S, Fontan JE, Alberti C, Bourdon O, Farnoux C, Macher MA, Foureau A, Faye A,	No	No	No	No	No	Yes	No	No	No	No	No	No	No	No	1

For peer review only

Beaufils F, Gottot S, Brion F. Drug administration errors and their determinants in pediatric in-patients. International Journal for Quality in Health Care. 2005 Oct 1;17(5):381-9.															
Rafferty AM, Clarke SP, Coles J, Ball J, James P, McKee M, Aiken LH. Outcomes of variation in hospital nurse staffing in English hospitals: cross-sectional analysis of survey data	No	No	No	Yes	No	No	No	No	No	No	No	No	No	Yes	2

For peer review only

and discharge records. International journal of nursing studies. 2007 Feb 1;44(2):175-82.														
Razmus I. Factors associated with pediatric hospital-acquired pressure injuries. Journal of Wound, Ostomy and Continence Nursing. 2018 Mar 1;45(2):107-16.	No	No	No	No	Yes	N o	No	No	No	No	No	No	No	No	1
Richet HM, Benbachir M, Brown DF, Giamarellou H, Gould I, Gubina M,	No	No	No	No	No	N o	No	No	No	No	No	Yes	No	No	1

For peer review only

Heczko P, Kalenic S, Pana M, Pittet D, Redjeb SB. Are there regional variations in the diagnosis, surveillance, and control of methicillin-resistant Staphylococcus aureus?. Infection Control & Hospital Epidemiology. 2003 May;24(5):334-41.															
Robertson RH, Hassan M. Staffing intensity, skill mix and mortality outcomes: the case of chronic obstructive	No	No	No	No	No	No	Yes	No	No	No	No	No	No	No	1

For peer review only

lung disease. Health Services Management Research. 1999 Nov;12(4):258-68.															
Robertson RH, Hassan M. Staffing intensity, skill mix and mortality outcomes: the case of chronic obstructive lung disease. Health Services Management Research. 1999 Nov;12(4):258-68.	No	No	No	No	No	No	No	No	No	No	No	No	Yes	No	1
Sasichay-Akkadechanunt T, Scalzi CC, Jawad AF. The relationship	No	No	Yes	No	No	No	No	No	No	No	No	No	No	No	1

For peer review only

between nurse staffing and patient outcomes. JONA: The Journal of Nursing Administration. 2003 Sep 1;33(9):478-85.															
Schreuders LW, Bremner AP, Geelhoed E, Finn J. The relationship between nurse staffing and inpatient complications. Journal of advanced nursing. 2015 Apr;71(4):800-12.	No	No	No	Yes	No	No	No	No	No	No	No	No	No	No	1
Schubert M, Ausserhofer D, Desmedt M,	No	No	No	No	No	No	No	Yes	No	No	No	No	No	No	1

For peer review only

Schwendimann R, Lesaffre E, Li B, De Geest S. Levels and correlates of implicit rationing of nursing care in Swiss acute care hospitals—a cross sectional study. International journal of nursing studies. 2013 Feb 1;50(2):230-9.															
Schultz MA, van Servellen G, Chang BL, McNeese-Smith D, Waxenberg E. The relationship of hospital structural and financial	Yes	No	No	No	No	No	No	No	No	No	No	No	No	No	1

For peer review only

characteristics to mortality and length of stay in acute myocardial infarction patients. Outcomes Management for Nursing Practice. 1998 Jul 1;2(3):130-6.															
Seago JA, Williamson A, Atwood C. Longitudinal analyses of nurse staffing and patient outcomes: more about failure to rescue. JONA: The Journal of Nursing Administration. 2006 Jan	No	No	No	No	No	No	No	No	No	No	Yes	No	No	No	1

For peer review only

1;36(1):13-21.															
Shamian J, Hagen B, Hu TW, Fogarty TE. The relationship between length of stay and required nursing care hours. The Journal of nursing administration. 1994 Jul 1;24(7-8):52-8.	No	No	No	No	No	No	No	No	No	No	No	No	Yes	No	1
Shuldham C, Parkin C, Firouzi A, Roughton M, Lau-Walker M. The relationship between nurse staffing and patient outcomes: a	No	No	No	No	Yes	No	No	No	No	No	Yes	No	No	Yes	3

For peer review only

case study. International journal of nursing studies. 2009 Jul 1;46(7):986-92.														
Silber JH, Kennedy SK, Even-Shoshan O, Chen W, Koziol LF, Showan AM, Longnecker DE. Anesthesiologist direction and patient outcomes. The Journal of the American Society of Anesthesiologists. 2000 Jul 1;93(1):152-63.	No	No	No	No	No	No	Yes	No	No	No	No	No	No	No	1
Silber JH, Kennedy SK, Even-	No	No	No	No	No	No	No	No	Yes	No	No	No	No	No	1

For peer review only

Shoshan O, Chen W, Koziol LF, Showan AM, Longnecker DE. Anesthesiologist direction and patient outcomes. The Journal of the American Society of Anesthesiologists. 2000 Jul 1;93(1):152-63.															
Silber JH, Rosenbaum PR, Ross RN. Comparing the contributions of groups of predictors: which outcomes vary with hospital rather than	No	No	No	No	No	No	No	No	Yes	No	No	No	No	No	1

For peer review only

patient characteristics?. Journal of the American Statistical Association. 1995 Mar 1;90(429):7-18.															
Silber JH, Rosenbaum PR, Schwartz JS, Ross RN, Williams SV. Evaluation of the complication rate as a measure of quality of care in coronary artery bypass graft surgery. Jama. 1995 Jul 26;274(4):317-23.	No	No	No	No	No	No	No	No	No	No	No	No	Yes	No	1
Sochalski J, Konetzka RT, Zhu J, Volpp K. Will	No	No	No	Yes	No	No	No	No	No	Yes	No	No	No	No	2

For peer review only

mandated minimum nurse staffing ratios lead to better patient outcomes?. Medical care. 2008 Jun 1:606-13.															
Sovie M. Hospital Restructuring's Impact on Outcomes: Report to Participating Hospitals.	No	No	No	No	No	N o	No	No	No	No	No	No	Yes	No	1
Sovie MD, Jawad AF. Hospital restructuring and its impact on outcomes: nursing staff regulations are premature. JONA: The Journal of	No	No	No	No	No	N o	No	No	Yes	No	No	No	No	No	1

For peer review only

Nursing Administration. 2001 Dec 1;31(12):588-600.															
Spetz J, Harless DW, Herrera CN, Mark BA. Using minimum nurse staffing regulations to measure the relationship between nursing and hospital quality of care. Medical Care Research and Review. 2013 Aug;70(4):380-99.	No	Yes	No	No	No	No	No	No	No	No	No	No	No	No	1
Staggs VS, Knight JE, Dunton N. Understandi	No	No	No	No	Yes	No	No	No	No	No	No	No	No	No	1

For peer review only

ng unassisted falls: effects of nurse staffing level and nursing staff characteristics. Journal of Nursing Care Quality. 2012 Jul 1;27(3):194-9.															
Stegenga J, Bell E, Matlow A. The role of nurse understaffing in nosocomial viral gastrointestinal infections on a general pediatrics ward. Infection Control & Hospital Epidemiolog	No	No	No	No	No	Y es	No	No	No	No	No	Yes	No	No	2

For peer review only

y. 2002 Mar;23(3):133-6.														
Stratton KM. Pediatric nurse staffing and quality of care in the hospital setting. Journal of nursing care quality. 2008 Apr 1;23(2):105-14.	No	No	No	No	No	Yes	No	No	No	No	No	Yes	No	No	2
Sujijantararat R, Booth RZ, Davis LL. Nosocomial urinary tract infection: nursing-sensitive quality indicator in a Thai hospital. Journal of nursing care quality. 2005 Apr	No	No	No	No	No	No	No	No	No	No	No	Yes	No	No	1

For peer review only

1;20(2):134-9.														
Sujijantararat R. Nursing-sensitive quality indicators: Nosocomial urinary tract infection in a Thai hospital. The University of Alabama at Birmingham; 2001.	No	No	Yes	No	No	No	No	No	No	No	No	No	No	No	1
Taunton RL, Kleinbeck SV, Stafford R, Woods CQ, Bott MJ. Patient outcomes. Are they linked to registered nurse absenteeism, separation, or work load?. The	No	No	No	No	No	No	No	No	No	No	No	No	Yes	No	1

For peer review only

Journal of nursing administration. 1994 Apr 1;24(4 Suppl):48-55.														
Taylor JA, Dominici F, Agnew J, Gerwin D, Morlock L, Miller MR. Do nurse and patient injuries share common antecedents? An analysis of associations with safety climate and working conditions. BMJ quality & safety. 2012 Feb 1;21(2):101-11.	No	No	No	No	No	No	No	No	No	No	Yes	No	No	No	1

For peer review only

Titler MG, Shever LL, Kanak MF, Picone DM, Qin R. Factors associated with falls during hospitalization in an older adult population. Research and Theory for Nursing Practice. 2011 Jan 1;25(2):127-52.	No	No	No	No	No	No	No	No	No	No	Yes	No	No	No	1
Tourangeau AE, Giovannetti P, Tu JV, Wood M. Nursing-related determinants of 30-day mortality for hospitalized patients. Canadian Journal of	No	No	No	No	No	No	No	No	Yes	No	No	No	No	No	1

For peer review only

Nursing Research Archive. 2002.															
Trinkoff AM, Johantgen M, Storr CL, Gurses AP, Liang Y, Han K. Nurses' work schedule characteristics, nurse staffing, and patient mortality. Nursing research. 2011 Jan 1;60(1):1-8.	No	No	No	No	No	No	No	No	No	Yes	No	No	No	No	1
Twigg D, Duffield C, Bremner A, Rapley P, Finn J. The impact of the nursing hours per patient day (NHPPD) staffing method on patient	No	Yes	No	Yes	No	No	No	No	No	No	Yes	Yes	No	No	4

For peer review only

outcomes: a retrospective analysis of patient and staffing data. International journal of nursing studies. 2011 May 1;48(5):540-8.															
Unruh L. Licensed nurse staffing and adverse events in hospitals. Medical care. 2003 Jan 1:142-52.	No	No	No	No	Yes	No	No	No	Yes	No	No	Yes	Yes	No	4
Unruh LY, Zhang NJ. Nurse staffing and patient safety in hospitals: new variable and longitudinal	No	No	No	No	No	No	No	No	No	No	Yes	Yes	No	No	2

For peer review only

approaches. Nursing research. 2012 Jan 1;61(1):3-12.															
Van den Heede K, Lesaffre E, Diya L, Vleugels A, Clarke SP, Aiken LH, Sermeus W. The relationship between inpatient cardiac surgery mortality and nurse numbers and educational level: analysis of administrative data. International journal of nursing studies. 2009 Jun	No	No	No	Yes	No	N o	No	No	No	No	No	No	No	No	1

For peer review only

1;46(6):796-803.															
Van den Heede K, Sermeus W, Diya L, Clarke SP, Lesaffre E, Vleugels A, Aiken LH. Nurse staffing and patient outcomes in Belgian acute hospitals: cross-sectional analysis of administrative data. International journal of nursing studies. 2009 Jul 1;46(7):928-39.	No	No	No	Yes	No	No	No	No	No	No	Yes	No	No	No	2
Van den Heede, K., Sermeus, W., Diya, L.,	No	No	No	No	Yes	No	No	No	No	No	No	No	No	No	1

For peer review only

Clarke, S. P., Lesaffre, E., Vleugels, A., & Aiken, L. H. (2009). Nurse staffing and patient out-comes in Belgian acute hospitals: Cross-sectional analysis of administrative data.														
Wan TT, Shukla RK. Contextual and organizational correlates of the quality of hospital nursing care. QRB-Quality Review Bulletin. 1987 Feb 1;13(2):61-4.	No	No	No	No	No	No	No	No	No	No	No	No	Yes	No	1

For peer review only

Weiss ME, Yakusheva O, Bobay KL. Quality and cost analysis of nurse staffing, discharge preparation, and postdischarge utilization. Health services research. 2011 Oct;46(5):1473-94.	No	No	No	No	No	No	No	Yes	No	No	No	No	No	No	1
Whitman GR, Kim Y, Davidson LJ, Wolf GA, Wang SL. The impact of staffing on patient outcomes across specialty units. JONA: the Journal of Nursing Administration. 2002	No	No	No	No	No	No	No	No	Yes	No	Yes	No	No	No	2

For peer review only

Dec 1;32(12):633-9.															
Wiltse Nicely KL, Sloane DM, Aiken LH. Lower mortality for abdominal aortic aneurysm repair in high-volume hospitals is contingent upon nurse staffing. Health services research. 2013 Jun;48(3):972-91.	No	No	No	Yes	No	No	No	No	No	No	No	No	No	No	1
Yang KP. Relationships between nurse staffing and patient outcomes. Journal of Nursing	No	No	No	No	No	No	No	No	No	No	No	Yes	No	No	1

For peer review only

Research. 2003 Sep 1;11(3):149-58.															
Yasunaga H, Hashimoto H, Horiguchi H, Miyata H, Matsuda S. Variation in cancer surgical outcomes associated with physician and nurse staffing: a retrospective observational study using the Japanese Diagnosis Procedure Combination Database. BMC health services research. 2012 Dec;12(1):1-7.	No	No	No	Yes	No	No	No	No	No	No	No	No	No	No	1

For peer review only

Yu D, Ma Y, Sun Q, Lu G, Xu P. A nursing care classification system for assessing workload and determining optimal nurse staffing in a teaching hospital in China: A pre-post intervention study. International journal of nursing practice. 2015 Aug;21(4):339-49.	No	No	Yes	No	No	No	No	No	No	No	No	No	No	No	1
Zhu, X.-W., You, L.-M., Zheng, J., Liu, K. E., Fang, J.-B., Hou, S.-X., … Zhang, L.-F. (2012).	No	No	No	No	Yes	No	No	No	No	No	No	No	No	No	1

For peer review only

Nurse staffing levels make a difference on patient outcomes: A multisite study in Chinese hospitals. Journal of Nursing Scholarship, 44(3), 266–273.															

For peer review only

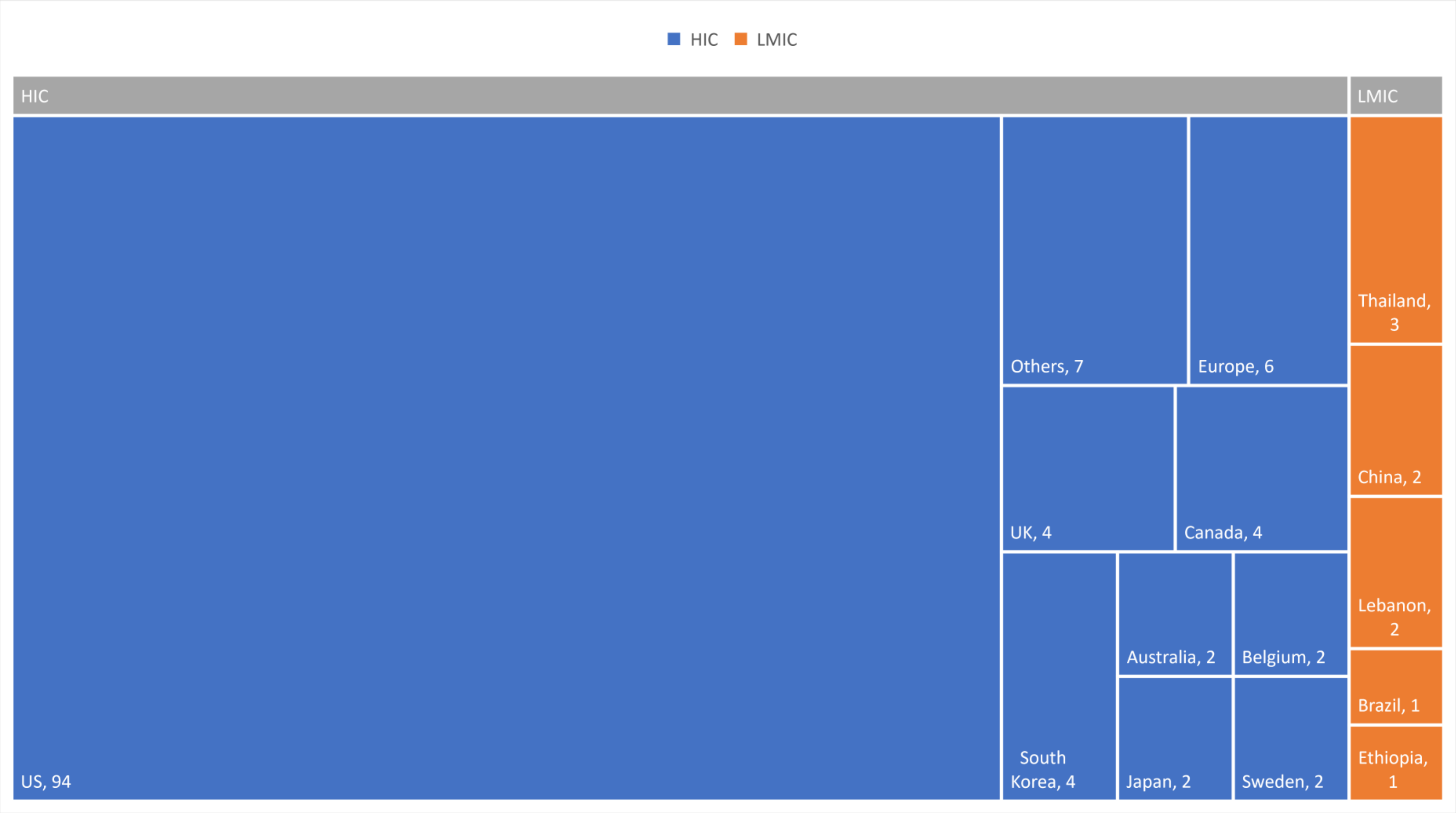

Online supplementary material 5 : Tree Map showing countries primary studies were conducted (n = 136).

Others group are HIC countries that had only 1 study – Kuwait, Taiwan, France, Finland + Netherlands (Dual country study), Multi-country, Italy, New Zealand.

*Studies conducted in Europe were conducted in multiple European countries. Two studies conducted in Lebanon and Sweden were also conducted in the US

For peer review only

Online supplemental material 6 – Summary of nursing metrics reported across papers

Nursing Metrics	Various definitions of individual metrics	Number of studies reporting a particular metric	Total number of studies reporting metrics
nursing hour per patient day	Total productive hours (care hours provided to patients) worked by registered nurses in a defined period/ Total number of patients admitted for care in the same defined period	33	45
	Total number of all nursing hours provided to patients in a defined period (includes any combination of registered nursing hours, licensed practice nursing hours and nurse assistant hours)/ Total number of patients admitted for care in the same defined period	11	
	Average number of nursing hours provided by registered nursing and licensed practice nursing in a defined period/average number of patients admitted for care in the same defined period	1	
Patient to nurse ratio per shift	Average reported number of patients by nurses on their last shift. Nurses' answers are averaged across a hospital to get a value.	32	32
Bed to nurse ratio/ Nurse per bed ratio	Total number of beds divided by the total number of full-time equivalent registered	2	10

For peer review only

	nurses (RNs) working in the general ward		
	Number of FTE nurses divided by the number of hospital beds	6	
	Number of inpatient nurses per 100 occupied beds	1	
	Infection control nurse to bed ratio	1	
Nurse to patient ratio/ patient to nurse ratio	The ratio of nurse productive hours worked to "patient census days/24	1	7
	The ratio of total nurse staff to the total number of patients in a defined period	2	
	The measure of the number of patients assigned to each nurse who has a direct patient care assignment	2	
	Undefined	2	
RN FTE per 1000 patient days	Number of Full-time registered nurses per 1000 patient-days	5	6
	The number of registered nurse full-time equivalents (FTEs) per 1000 in-patient days.	1	
RN per adjusted admission	Number of registered nurses/ adjusted admissions	1	2
	Number of full-time equivalent registered nurses per 100 adjusted admissions	1	
Others*		18	

*Others – Includes metrics that have frequencies of 1. This group includes - nursing hour per patient per shift, Inpatient clinical nursing worked hours per OCW), Mean difference between actual and expected nurse to patient ratio, RN hours per adjusted patient day, RN FTE, Total nursing hours, FTE RN to total hospital adjusted patient day, RN FTE per hospital, Daily average hours of care, All hours of direct care per patient day, RN per average daily census, The ratio of required to actual patient care hours, Required nursing personnel per shift, Number of licensed nurses, FTE RNs per adjusted average daily census, labour efficiency ratio, Daily average utilisation of RNs, Acuity-adjusted nursing hour per patient day

For peer review only

PRISMA 2020 Checklist

Section and Topic	Item #	Checklist item	Location where item is reported
**TITLE**			
Title	1	Identify the report as a systematic review.	Title page
**ABSTRACT**			
Abstract	2	See the PRISMA 2020 for Abstracts checklist.	Abstract (pg 2)
**INTRODUCTION**			
Rationale	3	Describe the rationale for the review in the context of existing knowledge.	Background (pg 5)
Objectives	4	Provide an explicit statement of the objective(s) or question(s) the review addresses.	Aims and Objectives (pg 6)
**METHODS**			
Eligibility criteria	5	Specify the inclusion and exclusion criteria for the review and how studies were grouped for the syntheses.	Selection of systematic reviews (pg 7)
Information sources	6	Specify all databases, registers, websites, organisations, reference lists and other sources searched or consulted to identify studies. Specify the date when each source was last searched or consulted.	Data sources and search strategy (pg 6 & 7)
Search strategy	7	Present the full search strategies for all databases, registers and websites, including any filters and limits used.	Data sources and search strategy (pg 6 & 7)
Selection process	8	Specify the methods used to decide whether a study met the inclusion criteria of the review, including how many reviewers screened each record and each report retrieved, whether they worked independently, and if applicable, details of automation tools used in the process.	Selection of systematic reviews (pg 7)
Data collection process	9	Specify the methods used to collect data from reports, including how many reviewers collected data from each report, whether they worked independently, any processes for obtaining or confirming data from study investigators, and if applicable, details of automation tools used in the process.	Data extraction (pg 8)
Data items	10a	List and define all outcomes for which data were sought. Specify whether all results that were compatible with each outcome domain in each study were sought (e.g. for all measures, time points, analyses), and if not, the methods used to decide which results to collect.	Selection of systematic reviews (pg 7)
	10b	List and define all other variables for which data were sought (e.g. participant and intervention characteristics, funding sources). Describe any assumptions made about any missing or unclear information.	Selection of systematic reviews (pg 7)
Study risk of bias assessment	11	Specify the methods used to assess risk of bias in the included studies, including details of the tool(s) used, how many reviewers assessed each study and whether they worked independently, and if applicable, details of automation tools used in the process.	Quality assessment (pg 8)
Effect measures	12	Specify for each outcome the effect measure(s) (e.g. risk ratio, mean difference) used in the synthesis or presentation of results.	Selection of systematic reviews, Data

For peer review only

PRISMA 2020 Checklist

Section and Topic	Item #	Checklist item	Location where item is reported
			synthesis (pg 7 and pg 9)
Synthesis methods	13a	Describe the processes used to decide which studies were eligible for each synthesis (e.g. tabulating the study intervention characteristics and comparing against the planned groups for each synthesis (item #5)).	Data synthesis (pg 9)
	13b	Describe any methods required to prepare the data for presentation or synthesis, such as handling of missing summary statistics, or data conversions.	Data synthesis (pg 9)
	13c	Describe any methods used to tabulate or visually display results of individual studies and syntheses.	Data synthesis (pg 9)
	13d	Describe any methods used to synthesize results and provide a rationale for the choice(s). If meta-analysis was performed, describe the model(s), method(s) to identify the presence and extent of statistical heterogeneity, and software package(s) used.	Data synthesis (pg 9)
	13e	Describe any methods used to explore possible causes of heterogeneity among study results (e.g. subgroup analysis, meta-regression).	Not applicable
	13f	Describe any sensitivity analyses conducted to assess robustness of the synthesized results.	Not applicable
Reporting bias assessment	14	Describe any methods used to assess risk of bias due to missing results in a synthesis (arising from reporting biases).	Quality assessment (pg 8)
Certainty assessment	15	Describe any methods used to assess certainty (or confidence) in the body of evidence for an outcome.	Not applicable
**RESULTS**			
Study selection	16a	Describe the results of the search and selection process, from the number of records identified in the search to the number of studies included in the review, ideally using a flow diagram.	Search results (figure 1)
	16b	Cite studies that might appear to meet the inclusion criteria, but which were excluded, and explain why they were excluded.	Supplementary file 2
Study characteristics	17	Cite each included study and present its characteristics.	Table 1 (pg 11 & 12)
Risk of bias in studies	18	Present assessments of risk of bias for each included study.	Figure 2
Results of individual studies	19	For all outcomes, present, for each study: (a) summary statistics for each group (where appropriate) and (b) an effect estimate and its precision (e.g. confidence/credible interval), ideally using structured tables or plots.	Not applicable
Results of syntheses	20a	For each synthesis, briefly summarise the characteristics and risk of bias among contributing studies.	Quality assessment of included studies (pg 13), Figure 2
	20b	Present results of all statistical syntheses conducted. If meta-analysis was done, present for each the summary estimate and its precision (e.g. confidence/credible interval) and measures of statistical heterogeneity. If comparing groups, describe the direction of the effect.	Figure 3, Figure 4, Supplementary file 5, 6 and 7

For peer review only

PRISMA 2020 Checklist

Section and Topic	Item #	Checklist item	Location where item is reported
	20c	Present results of all investigations of possible causes of heterogeneity among study results.	Not applicable
	20d	Present results of all sensitivity analyses conducted to assess the robustness of the synthesized results.	Not applicable
Reporting biases	21	Present assessments of risk of bias due to missing results (arising from reporting biases) for each synthesis assessed.	Not applicable
Certainty of evidence	22	Present assessments of certainty (or confidence) in the body of evidence for each outcome assessed.	Not applicable
**DISCUSSION**			
Discussion	23a	Provide a general interpretation of the results in the context of other evidence.	Discussion (pg 15 – 17)
	23b	Discuss any limitations of the evidence included in the review.	Strength and limitations (pg 17 & 18)
	23c	Discuss any limitations of the review processes used.	Strength and limitations (pg 17 & 18)
	23d	Discuss implications of the results for practice, policy, and future research.	Discussion (pg 15 – 17)
**OTHER INFORMATION**			
Registration and protocol	24a	Provide registration information for the review, including register name and registration number, or state that the review was not registered.	Abstract (pg 2), Research design (pg 6)
	24b	Indicate where the review protocol can be accessed, or state that a protocol was not prepared.	Research design (pg 6)
	24c	Describe and explain any amendments to information provided at registration or in the protocol.	Research design (pg 6)
Support	25	Describe sources of financial or non-financial support for the review, and the role of the funders or sponsors in the review.	Funding (pg 20)
Competing interests	26	Declare any competing interests of review authors.	Competing interests (pg 20)
Availability of data, code and other materials	27	Report which of the following are publicly available and where they can be found: template data collection forms; data extracted from included studies; data used for all analyses; analytic code; any other materials used in the review.	Data availability statement (pg 20)

From: Page MJ, McKenzie JE, Bossuyt PM, Boutron I, Hoffmann TC, Mulrow CD, et al. The PRISMA 2020 statement: an updated guideline for reporting systematic reviews. BMJ 2021;372:n71. doi: 10.1136/bmj.n71

For more information, visit: http://www.prisma-statement.org/

For peer review only
